# A Polypharmacology-Driven Approach to Alzheimer’s Disease and Tauopathies: Rational Design, Synthesis and Characterization of Amino-Pyrazole-Based Multikinase (GSK-3β/FYN-α/DYRK1A) Inhibitors

**DOI:** 10.1021/acs.jmedchem.5c01810

**Published:** 2026-04-29

**Authors:** Stefania Demuro, Debora Russo, Ilaria Penna, Siranuysh Grabska, Hovakim Grabski, Andrea Dalle Vedove, Aurora Valeri, Conall Sauvey, Giuliana Ottonello, Maria Summa, Sine Mandrup Bertozzi, Jose Ortega, Rosalia Bertorelli, Paola Storici, Stefania Girotto, Gabriele Cruciani, Rita M. C. Di Martino, Ruben Abagyan, Andrea Cavalli

**Affiliations:** † Computational and Chemical Biology, 121451 Istituto Italiano di Tecnologia, via Morego 30, 16163 Genova, Italy; ‡ Department of Pharmacy and Biotechnology, University of Bologna, via Belmeloro 6, 40126 Bologna, Italy; § D3 PharmaChemistry, 121451 Istituto Italiano di Tecnologia, via Morego 30, 16163 Genova, Italy; ∥ Skaggs School of Pharmacy and Pharmaceutical Sciences, 8784 University of California, La Jolla, San Diego, California 92093, United States; ⊥ L.A. Orbeli Institute of Physiology, National Academy of Sciences, Yerevan 0028, Armenia; # 18469 AREA Science Park, Padriciano 99, 34149 Trieste, Italy; ∇ Protein Targets for Drug Discovery Lab, Elettra Sincrotrone Trieste S.C.p.A., SS 14 - km 163,5 in AREA Science Park, 34149 Trieste, Italy; ○ Molecular Horizon srl, via Montelino 20, 06084 Bettona, Italy; ◆ Analytical Chemistry Facility, 121451 Istituto Italiano di Tecnologia, via Morego 30, 16163 Genova, Italy; ¶ Translational Pharmacology Facility, 121451 Istituto Italiano di Tecnologia, via Morego 30, 16163 Genova, Italy; & Department of Chemistry, Biology and Biotechnology, University of Perugia, via dell’Elce di Sotto 8, 06123 Perugia, Italy

## Abstract

Accumulation of microtubule-associated protein tau is a neurotoxic hallmark in Alzheimer’s disease (AD) and related tauopathies. To date, no small molecule disease-modifying therapy exists, underscoring an urgent unmet need. In this context, the multitarget-directed ligand (MTDL) approach offers a viable polypharmacological option for modulating key pathways/targets involved in tau pathology. Leveraging the interconnected roles of GSK-3β, FYN, and DYRK1A in tau hyperphosphorylation, we conducted a computational and X-ray crystallography-driven SAR exploration around our previously disclosed GSK-3β/FYN/DYRK1A inhibitor ARN25068 (**1**). Modification of the thieno­[3,2-*d*]­pyrimidine central core of **1** led to the discovery of quite well-balanced GSK-3β/FYN/DYRK1A triple-targeting analogs (**27**, **28** (ARN25699) and **31** (ARN26646)). Among these, **28** displayed a favorable ADME profile, acceptable pharmacokinetic properties, and efficacy in an in vitro tau phosphorylation assay, outperforming three single-target inhibitors tested individually or in combination. These compounds represent promising MTDL leads poised to advance therapeutic innovation in AD and related tauopathies.

## Graphical abstract

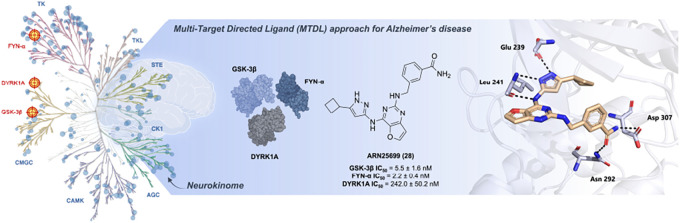

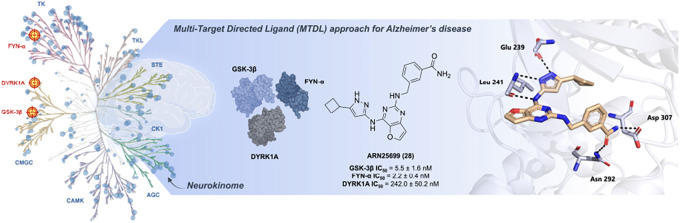

## Introduction

Tauopathies encompass a group of complex adult-onset neurodegenerative disorders characterized by intricate pathophysiological mechanisms. A defining neuropathologic hallmark of these disorders is the abnormal deposition of post-translationally hyperphosphorylated tau-positive intracellular inclusions, also known as neurofibrillary tangles (NFTs).^1^ In Alzheimer’s disease (AD) and related tauopathies, the aberrant hyperphosphorylation of tau protein arises from an imbalance in the activity of various intracellular regulators, including protein kinases (PKs) and phosphatases (PPs), which triggers a cascade of toxic events leading to neuronal death.^2,3^ The multifactorial etiology of these conditions poses a significant challenge in the development of effective therapeutic options. Furthermore, the clinical failures in developing single-target agents for AD underscore the urgent need to explore novel polypharmacological strategies.^4^

Despite the approval of disease-modifying biologics targeting amyloid-beta (Aβ) plaques, such as lecanemab (Leqembi), aducanumab (Aduhelm), and donanemab (Kisunla), no disease-modifying small molecules have been approved for AD.^5,6^ Additionally, 9% of clinical trials target tau protein, with 3% of Phase 3 trials and 7% of Phase 2 trials involving tau-directed agents.^7^

Given this landscape, a thorough evaluation of tau-targeting therapeutics in AD and related tauopathies is needed. Effective tau-targeting agents should meet multiple criteria, including robust effects in appropriate preclinical models, adequate brain penetration, and minimal peripheral side effects.^8^ Moreover, since tau is phosphorylated at more than 40 different sites, a drug combination of protein kinase inhibitors (PKIs) or multitarget PKIs holds promise for selectively targeting relevant kinases while minimizing off-target effects.

From this perspective, the neurokinome holds a great deal of promise owing to two key factors: (1) PKs play a central role in regulating multiple intracellular pathways and serve as critical regulators of divergent signaling cascades, and (2) their high degree of sequence conservation makes them adaptable targets for multitarget-based drug discovery programs.^4,9,10^

Among kinases implicated in tauopathies, glycogen synthase kinase-3β (GSK-3β), FYN proto-oncogene (FYN), and dual-specificity tyrosine-phosphorylation-regulated kinase 1A (DYRK1A) deserve particular attention given their elevated expression in distinct brain regions, their associations with both the onset and progression of neurodegenerative conditions, and evidence of functional crosstalk between the three proteins.^11^ GSK-3β phosphorylates tau protein mainly at Ser199, Ser396, and Ser413,^12^ whereas FYN, which interacts with the amino-terminal domain of tau, targets Tyr18.^13^ DYRK1A phosphorylates tau at multiple Ser/Thr residues, with Thr212 being the most prominent; this modification event acts as a priming step for subsequent GSK-3β-mediated phosphorylation.^14^ Additionally, during endoplasmic reticulum (ER) stress in AD pathogenesis, activation of FYN protein may contribute to GSK-3β up-regulation through increased phosphorylation at Tyr216.^15,16^

GSK-3β is a widely expressed serine/threonine kinase that regulates key neuronal processes, including development, metabolic homeostasis, and cell fate. In AD, GSK-3β is hyperactivated, and substantial evidence indicates that this protein is a major kinase responsible for pathological tau phosphorylation. It also represents a central link between Aβ and tau: tau overexpression promotes GSK-3β activation and contributes to its downstream toxicity, whereas GSK-3β pathological activation by Aβ further increases tau phosphorylation. Furthermore, inhibition of GSK-3β has been shown to reduce Aβ production and Aβ-mediated neurotoxicity by limiting the BACE-1-driven cleavage of the amyloid precursor protein (APP).^17–19^

Similar to GSK-3β, FYN is involved in APP phosphorylation, thereby promoting amyloidogenic processing and contributing to tau hyperphosphorylation and NFT formation. FYN also amplifies Aβ- and α-synuclein–mediated neurotoxicity. In addition, FYN acts as a critical mediator of inflammatory signaling in AD, owing to its sustained upregulation during neuroinflammation and constitutive expression in microglia and astrocytes.^20,21^

The proline-directed protein kinase DYRK1A is markedly dysregulated in AD and other tauopathies and facilitates tau alternative splicing, influencing cellular processes such as apoptosis and neurodegeneration. In addition to tau, DYRK1A phosphorylates several other substrates relevant to AD pathophysiology, such as APP and the transcription factor CREB, a key regulator of learning and memory.^22,23^

Taken together, these findings indicate that effective therapeutic strategies will likely require direct modulation of GSK-3β in addition to targeting FYN and DYRK1A in this interconnected kinase network.^11^

Despite substantial preclinical evidence, few kinase inhibitors targeting GSK-3β, FYN, and DYRK1A have advanced into clinical trials (Figure 1). Tideglusib, a well-known GSK-3β inhibitor, entered Phase IIa but failed to demonstrate significant improvement in cognitive function or other secondary end points compared to placebo.^24,25^ Other GSK-3 inhibitors, such as AR-A014418,^26^ showed efficacy in preclinical models of AD. Saracatinib, targeting FYN, represents a promising repurposed drug for inhibiting tau hyperphosphorylation together with other inhibitors of the Src family, including masitinib and dasatinib, which have progressed to Phase III and II clinical trials for AD, respectively.^27–31^ Similarly, CX-40945, a casein kinase 2 (CK2) inhibitor FDA approved for the treatment of solid tumors and under clinical investigation for a variety of diseases in the field of cancer and virology, showed nanomolar activity against DYRK1A and GSK-3β, and the capability to suppress tau hyperphosphorylation in the hippocampus of a Down syndrome (DS)-like mouse model after oral administration.^32,33^ The dual DYRK1A/GSK-3β nanomolar inhibitor SM07883 entered Phase I in Australia and New Zealand for AD in 2019,^34^ and the DYRK1A inhibitor leucettinib-21 recently reached Phase I for the management of DS and AD.^35^

**Figure 1:**
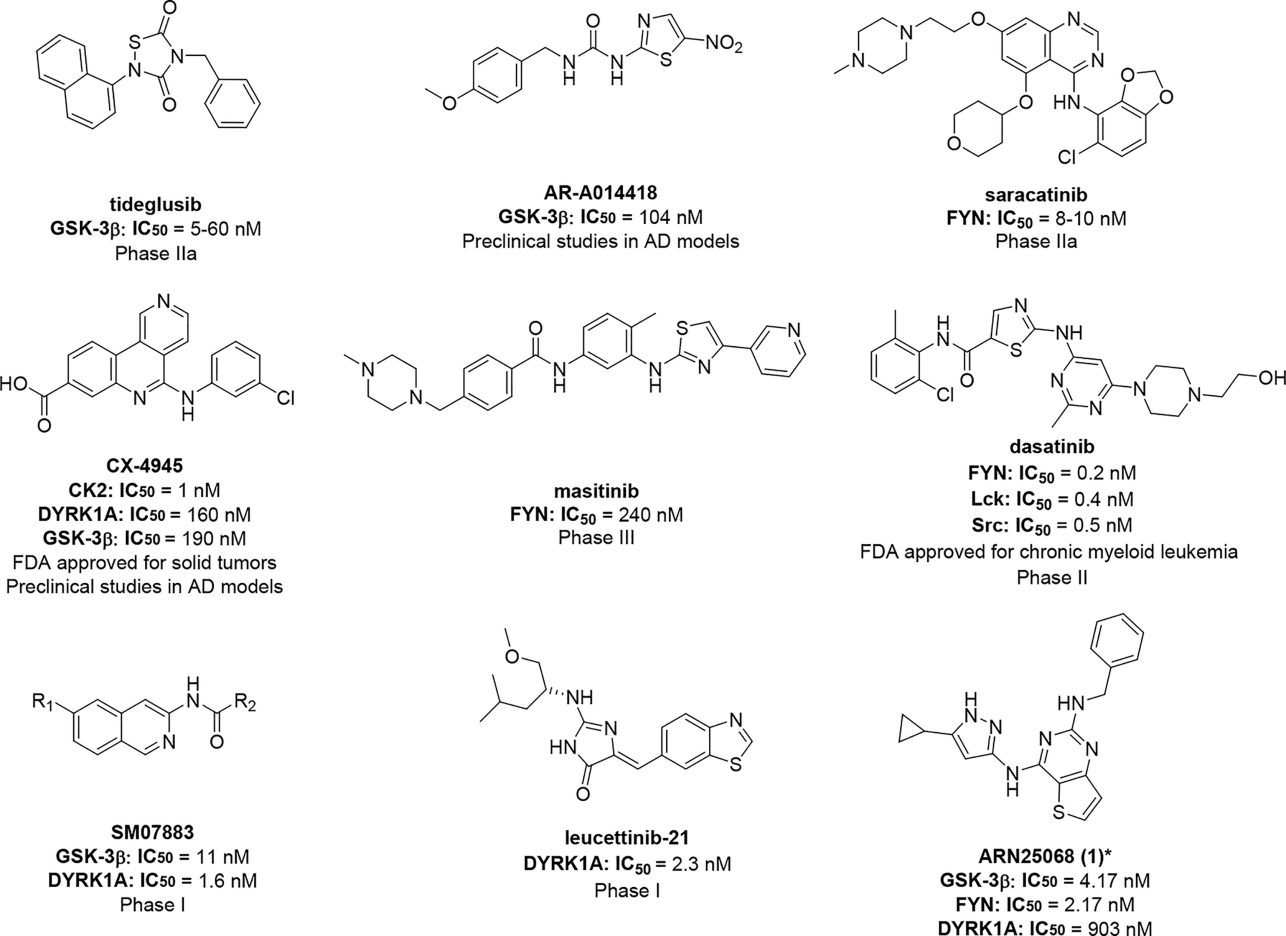
Structure and biological activity of representative single-target and multitarget GSK-3β/FYN/DYRK1A inhibitors evaluated under clinical and preclinical investigation for AD. The most advanced clinical trial phase for AD is reported based on the latest data from www.clinicaltrials.gov. Study status information as of February 2026. *Compound **1** was tested in a human CMGC kinase enzymatic radiometric assay [km ATP].^39^

Notably, none of the disease-modifying small molecules currently in clinical trials have been rationally designed as multitarget agents; rather, their multitarget activity often emerges serendipitously or because of the difficulty in achieving selectivity, particularly due to the high sequence homology within the ATP-binding pockets of kinases. This gap underlines both the inherent challenges and the significant potential of exploring a strategic multitarget approach.

Moreover, no rationally designed inhibitors targeting GSK-3β, FYN, and DYRK1A have been reported to date, offering an opportunity to harness the promiscuity of PKs while selectively modulating key players in tau hyperphosphorylation and potentially amplifying therapeutic efficacy through synergistic action.

In this context, designed polypharmacology, defined as the rational modulation of multiple disease-relevant targets through a single molecular entity, offers a rational framework to address the complexity underlying multifactorial disorders, including neurodegenerative diseases. Unlike unintended polypharmacology or multitarget activity arising from limited selectivity, this strategy enables the rational and controlled incorporation of complementary mechanisms of action within a single molecular framework. Such a rational design paradigm has the potential not only to enhance the overall therapeutic efficacy and overcome resistance but also to reduce polypharmacy by streamlining treatment into a single, well-optimized compound.^36–38^

Within this rational multitarget-design perspective, we previously disclosed ARN25068 (**1**, Figure 1) as a versatile starting point, featuring well-balanced low nanomolar potency against GSK-3β and FYN and low micromolar activity against DYRK1A.^39^

Considering the versatility of **1** (ARN25068), we embarked on a rational optimization campaign to develop more balanced GSK-3β/FYN/DYRK1A triple inhibitors as potential multitarget options for the treatment of AD and related disorders. Our goal was to achieve a balanced activity profile against all three targets while ensuring acceptable drug-likeness properties. This would provide proof of principle for the effectiveness of synergistic inhibition of the selected PKs, a crucial prerequisite for multitarget drug design.^37^ To better elucidate the pharmacophoric traits required for maintaining nanomolar potency against GSK-3β and FYN, while improving binding affinity against DYRK1A, we conducted a computationally driven SAR study. This exploration leveraged insights from new crystallographic structures of both DYRK1A and GSK-3β bound to different ligands.

## Results and Discussion

Our SAR investigation was driven by the high homology among the ATP-binding cavity of GSK-3β/FYN-α and DYRK1A, despite their differences in size and shape. Previous computational findings enabled us to identify DYRK1A as the primary kinase to optimize for inhibitory potency as an initial strategy to balance activity across all three targets.^39^

Thus, to validate our predictive computational model, the chemical space around the 2,4-disubstituted pyrimidine central core was first investigated by placing single, properly addressed modifications at three different positions while maintaining the aminopyrazole moiety, which engages in key hydrogen-bond (HB) interactions with the hinge region of the three PKs. To this end, we designed and synthesized three different series (I-II-III) of novel derivatives, each incorporating a single modification (Figure 2).

**Figure 2:**
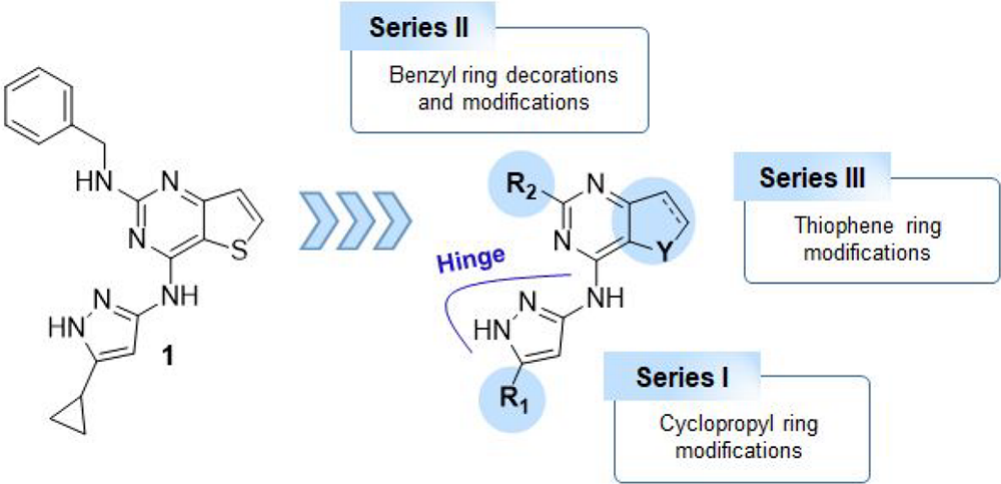
Schematic representation of the SAR exploration starting from compound **1**.

To explore the aminopyrazole moiety of **1**, the cyclopropyl ring was exchanged with substituents of diverse steric hindrance and electronic properties (R_1_, Figure 2 and Table 1), including a hydrogen atom (**2**), small lipophilic substituents such as methyl, isopropyl, and cyclobutyl groups (**3**–**5**), and aromatic rings such as phenyl (**6**) and 3-pyridine (**7**).

**Table 1:**
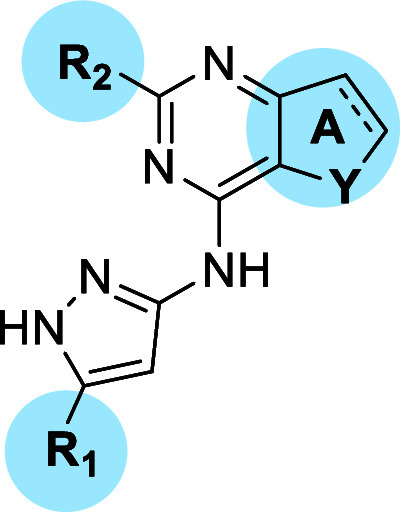

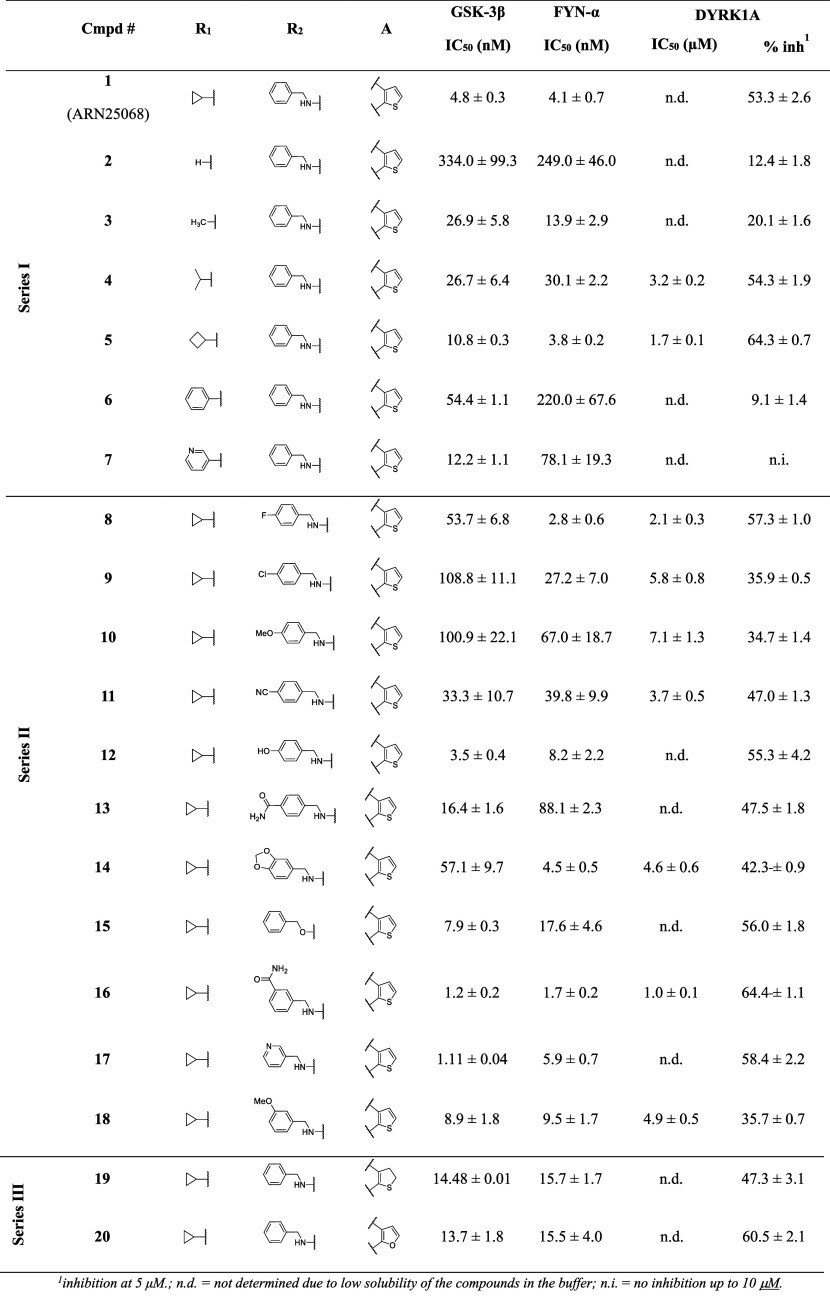
GSK-3β, FYN-α, and DYRK1A Inhibition Data of Series I–II–III Derivatives^a^

a To improve readability, IC_50_ values are reported in either nanomolar (nM; GSK-3β and FYN-α) or micromolar (μM; DYRK1A) units, to reflect the different potency ranges observed for the tested compounds across the three targets.

b Inhibition at 5 μM.; n.d. = not determined due to low solubility of the compounds in the buffer; n.i. = no inhibition up to 10 μM.

In series II, the benzylic function of **1** was decorated at *the para* and *meta* positions (Figure 2 and Table 1), as predicted, docking scores indicated a consistent improvement in binding affinity compared to *ortho* substitutions. A variety of electron-withdrawing groups (e.g., −F and −Cl: **8** and **9**, respectively; CN: **11**; CONH_2_: **13** and **16**; and 3-pyr: **17**) and electron-donating groups with different steric hindrance (OCH_3_: **10**; OH: **12**; and methandiol: **14**) were inserted on the benzyl moiety. In analog **15**, the benzylamine moiety of hit compound **1** was replaced with a benzyloxy one. The choice of the nature and position of these substituents was aimed at improving the selectivity and binding affinity of **1** by forming additional HBs with hydrophilic residues present in the large lipophilic niche of DYRK1A (Supporting Information, Figure S1A).

Given the high degree of rigidity of the pyrimidine central core and its indirect but essential role in stabilizing the binding pose of **1** at the ATP binding pockets of all three targets (Supporting Information, Figure S1A–C), a preliminary exploration around the thiophene ring was also performed (Figure 2 and Table 1). In this series, the sulfur atom of the 2,4-substituted-pyrimidinthiophene central core was first replaced with an HB-accepting oxygen atom (**20**) in order to assess whether additional HB interactions within the DYRK1A pocket would enhance ligand affinity for the enzyme while retaining potent and well-balanced nanomolar activity against GSK-3β and FYN-α. Additionally, the corresponding dihydrothiophene-based derivative **19** was synthesized to improve the flexibility of the pyrimidine-thiophene ring and evaluate the contribution of the aromaticity of the central core to the inhibition of all three PKs.

All synthesized compounds were screened in an *in-house* optimized LANCE Ultra time-resolved fluorescence energy transfer (TR-FRET) assay against GSK-3β, FYN-α, and DYRK1A (Table 1). Fluorescence-based kinase assay formats (e.g., LANCE Ultra and TR-FRET) are among the most widely used methods in kinase drug discovery because they are automation-friendly, straightforward to implement, and relatively cost-effective. During assay development, each target (GSK-3β, FYN, and DYRK1A) was individually optimized to establish a robust and reliable platform under target-specific conditions.

The removal of the cyclopropyl moiety (**2**) proved to be detrimental for the activity against all three PKs, with an IC_50_ of 334 nM for GSK-3β and 249 nM for FYN-α and only 12% of inhibitory activity against DYRK1A. This trend was corroborated by docking simulations, which yielded lower binding scores for all three targets (Supporting Information, Figure S1D–F). Derivative **2** lacked hydrophobic interaction with several residues within the DYRK1A binding pocket, including I1e165, the gatekeeper Phe238, Gly166 of the glycine-rich loop, and Phe170 (Supporting Information, Figure S1D). This observation confirmed that, despite differences in the binding pockets of GSK-3β, FYN- α, and DYRK1A, a single chemical entity attached to the aminopyrazole moiety is essential for establishing hydrophobic interactions at the ATP-binding sites of all three kinases.

The importance of maintaining the substituent directly attached to the aminopyrazole was additionally confirmed by testing the derivatives featuring alkyl and cycloalkyl groups (**3**–**5**) (Supporting Information, Figure S1G–O), which were better tolerated than aryl and heteroaryl groups (**6** and **7**) (Supporting Information, Figure S1P–U). Regarding FYN-α, **6** and **7** exhibited the weakest activities in the series, similar to compound **2**. The in silico analysis revealed that both compounds were unable to retain strong hydrophobic interactions with Val25 and Ala147, which are characteristic of compound **1** (Supporting Information, Figure S1C,R,U).

Regarding GSK-3β, compounds **1**, **6**, and **7** exhibited similar binding poses. However, unlike compound **1**, both compounds **6** and **7** lack van der Waals interactions with Asn186 and hydrophobic interactions with Thr138 (Supporting Information, Figure S1B,Q,T).

The best DYRK1A performing inhibitor of this series featured a cyclobutyl moiety (**5**, DYRK1A IC_50_ = 1.7 μM; % inh @ 5 μM = 64%) instead of a cyclopropyl group. Although both the cyclopropyl group in **1** and the cyclobutyl moiety in **5** interact with Phe238 of DYRK1A, the larger cyclobutyl moiety provides an increased hydrophobic interaction surface compared to the cyclopropyl group (Supporting Information, Figures S1A,M and S2). Moreover, compound **5** retained a well-balanced low nanomolar activity against GSK-3β and FYN-α (IC_50_ = 10.8 and 3.8 nM, respectively, Table 1). Very good results were also achieved with 5-isopropyl-1*H*-pyrazol-3-yl derivative **4** (Table 1), which demonstrated an IC_50_ value of 3.2 μM against DYRK1A and well-balanced inhibition of GSK-3β and FYN-α in the midnanomolar range (IC_50_ = 26.7 nM for GSK-3β and IC_50_ = 30.1 for FYN-α).

In vitro assessment of inhibitory activity was essential for a comprehensive, rational understanding of the effects of small modifications to the benzyl moiety of **1** in Series II derivatives. All the replacements and decorations were well tolerated against all three enzymes (Table 1).

Computational studies indicate that compound **8** interacts with DYRK1A and FYN-α in a similar fashion to what was observed for **1** (Supporting Information, Figure S3A,C), whereas the *para*-substituted fluorobenzyl group in compound **8** slightly deviates from the pose observed for **1** in the GSK-3β binding pocket (Supporting Information, Figure S3B). Overall, compound **8** engages with the same amino acid residues as compound **1** across all three kinases. The packing into the FYN-α binding pocket appears to be tight initially for compound **8**, but a small refinement restored a good fit (Supporting Information, Figure S3C). Similarly, the large chlorine substituent of compound **9** does not fit well in the protein pockets of DYRK1A and FYN-α (Supporting Information, Figure S3D,F). In further detail, compound **8** (Table 1) demonstrated an 11-fold decrease in the inhibitory activity against GSK-3β compared to **1**, while the insertion of a larger chlorine atom showed a more detrimental effect (23-fold decrease) on the enzyme inhibition (**9**, Table 1, Supporting Information Figure S3E). The same modifications were better tolerated by the other two targets, which retained IC_50_ values in the nanomolar range (2.8 nM for **8** and 27.2 nM for **9**) for FYN-α and low micromolar range (2.1 μM for **8** and 5.8 μM for **9**) for DYRK1A. The insertion of the bulky *p*-methoxy moiety (**10**) produced a similar effect to **9** for GSK-3β and DYRK1A, but led to a 16-fold decrease in FYN-α affinity compared to **1** (Table 1). Furthermore, the installation of a *para*-carbamoyl function on the benzylamine group (**13**) slightly reduced the potency against GSK-3β and DYRK1A compared to **1**, while a more marked decrease in FYN-α inhibition was observed, although the activity remained in the two-digit nanomolar range (IC_50_ = 88.1 nM) (Table 1). Interestingly, shifting the same substituent to the *meta* position (**16**) led to a remarkable improvement in GSK-3β and FYNα inhibition (IC_50_ values around 1 nM), along with an IC_50_ value of 1.0 μM against DYRK1A, suggesting a key role for this modification in GSK-3β/FYNα and DYRK1A inhibition. The favorable outcomes obtained with this compound were further corroborated by X-ray crystallography (Figure 3).

**Figure 3:**
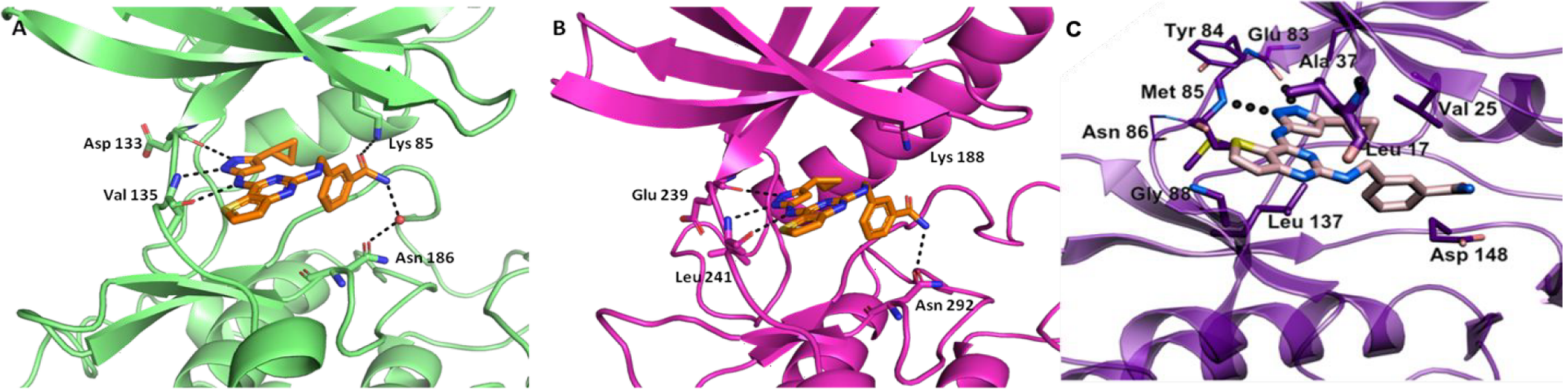
Cartoon models of the X-ray crystal structures of compound **16** in complex with GSK-3β, PDB ID: 9FR6 (A), and with DYRK1A, PDB ID: 9FUF (B). (C) Best docking pose of compound **16** in the FYN binding pocket. HBs are represented by the dotted lines.

The X-ray crystal structure of **16** in complex with GSK-3β (PDB ID: 9FR6, Figure 3A) shared a similar binding pose with compound **1**. This pose is characterized by three HBs within the hinge region of the enzyme, a water-mediated contact with the Asn186 side-chain, and a novel predicted interaction between Lys85 of this latter kinase and the *m*-carbamoyl function of the ligand. A comparable binding mode was observed for DYRK1A in complex with **16** (PDB ID: 9FUF, Figure 3B), in which the three HBs between the 3-aminopyrazole moiety and the protein backbone were conserved, and a direct interaction of the *m*-carbamoyl group with the side-chain of Asn292 was identified. Due to the poorer resolution of this structure, we cannot appreciate water-mediated interactions; however, the 4.4 Å O-N distance between the *m*-carbamoyl moiety of **16** and the side-chain of Lys188 suggests the possibility of such an interaction, which may contribute to the higher binding affinity measured for this target. Finally, the high conformational and sequence similarity of the ATP binding pockets among the selected kinases, as well as the known cocrystal structure of FYN (kinase domain) with staurosporine (PDB ID: 2DQ7), helped the docking prediction of the pose of compound **16**. In this model, **16** exhibited a hydrogen bonding pattern similar to that observed for GSK-3β and DYRK1A (Figure 3A, B), maintaining interactions between the aminopyrazole moiety and the backbone atoms of Glu83, Tyr84, Met85, and Asn86 of FYN-α (Figure 3C).

Similar to what was observed with compound **16**, the 3-picolylamine derivative (**17**) exhibited a slight improvement in DYRK1A inhibition, reaching 58% at 5 μM, while retaining low nanomolar activity against GSK-3β and FYN-α (IC_50_ = 1.1 and 5.9 nM, respectively, Table 1).

These preliminary results, which align well with the predictive computational models, illustrated how small differences in the shape of the binding pockets of the three targets influence the benzyl accommodation. Furthermore, the predicted binding poses suggest greater tolerance for *meta* substitutions in the DYRK1A pocket than for *para* substitutions.

The inhibitory activity of compounds **19** and **20** (Series III) showed no significant differences in binding with FYN-α and GSK-3β (Table 1). However, a notable conformational change was observed for furo­[3,2-*d*]­pyrimidine-based derivative **20** in the X-ray structure solved in complex with DYRK1A (Supporting Information, Figure S4), which can explain the slightly enhanced activity toward DYRK1A (61% inhibition at 5 μM).

In contrast, the removal of aromaticity in compound **19** altered molecule planarity and resulted in a slight decrease of DYRK1A inhibitory potency (47% inhibition at 5 μM). In **20**, the 2,4-substituted furopyrimidine central core shifted to the top of the DYRK1A pocket, thus facilitating interactions with different residues compared to compounds **1** and **19**. This shift also promoted the proximity of the polar furanyl moiety to the backbone nitrogen of Leu241, as confirmed by X-ray crystallographic data (PDB ID: 9FT4, Supporting Information, Figure S4).

These findings suggested the importance of having a planar molecular frame bridging the two extremities of the molecule and, therefore, the suitability of an aromatic central core to confer a rigid conformation for optimal targets’ interaction.

### Hybrid Derivatives

Prior chemical modifications within Series I–II–III provided valuable insights into optimal substitution patterns, thereby guiding the rational design of a novel set of improved hybrid molecules that incorporate merged pharmacophoric traits. Specifically, six interesting derivatives (**4**, **5**, **15**, **16**, **17**, and **20**) from the first-generation series were selected as a starting platform for a merging strategy to rationally design hybrid inhibitors. This approach involved combining two modifications (**21**–**26**) or three modifications (**27** and **28**) at once onto the compound **1** framework.

Although solubility issues in the assay buffer prevented the determination of IC_50_ values against DYRK1A of some derivatives, the percentage of inhibition at 5 μM was measured (Table 2), showing results consistent with docking predictions.

**Table 2:**
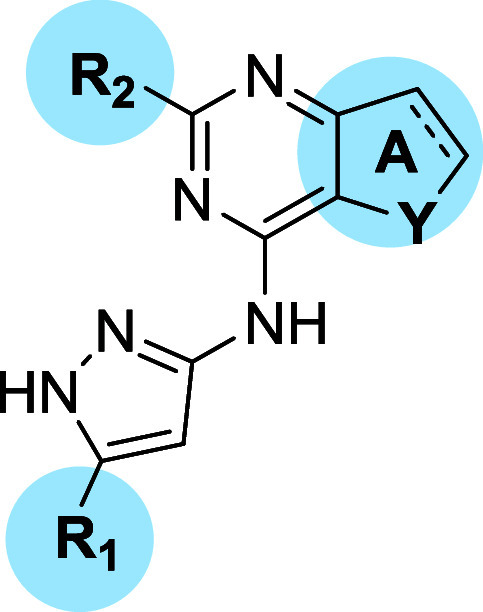

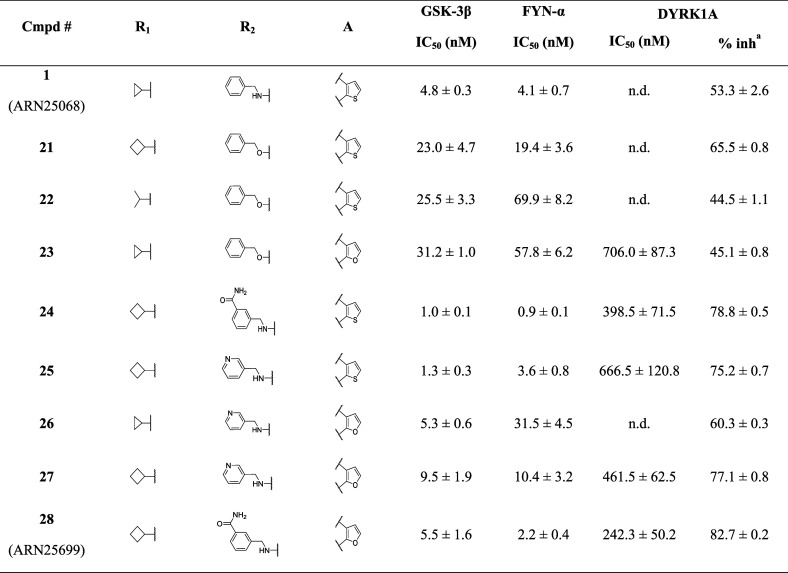
GSK-3β, FYN-α and DYRK1A Inhibition Data of Series IV-Hybrid Derivatives

a Inhibition at 5 μM; n.d. = not determined due to low solubility of the compounds in the buffer.

A decrease in binding affinity was observed for FYN-α and GSK-3β with compounds **21**, **22**, and **23**, while their overall binding pose remained similar to that of compound **1** (data not shown). With the only exception of benzyloxy derivatives **22** and **23**, for which a decrease of DYRK1A inhibition was observed (45% inhibition) in comparison to **1**, the combination of two modifications at a time provoked a significant improvement in DYRK1A affinity with IC_50_ values in the high nanomolar range (particularly in **24** and **25**), while keeping nanomolar activity on FYN-α and GSK-3β.

Compounds **27** and **28** (ARN25699), featuring three merged substitutions, showed the greatest improvement, with a well-balanced and low nanomolar inhibitory activity against GSK-3β and FYN-α and a notable increase in potency toward DYRK1A (IC_50_ = 462 and 242 nM, respectively, Table 2).

In light of these results, and with the goal of selecting the best candidate for further in vivo pharmacokinetic profile evaluation, compounds **27** and **28** were chosen for an in vitro ADME profiling, in a comparative fashion alongside hit compound **1** (Table 3). Compound **1** showed good phase I metabolic stability in both mouse and human liver microsomes (MLMs and HLMs, respectively), as well as good plasma stability in both species, making it an optimal starting point. However, it suffers from low kinetic solubility. Hybrids **27** and **28** preserved the favorable plasma stability of **1**, showing a decrease in phase I metabolic stability in MLMs compared to **1**, with half-life values of approximately 20 min. Of note, *m*-carbamoyl analog **28** not only exhibited greater stability than the 3-pyridine substituted derivative **27** in HLMs but also exhibited good kinetic solubility (*S*_k_ = 85 ± 30 μM) compared to both hit compounds **1** and hybrid **27**. These properties position **28** as the most promising GSK-3β/FYN-α/DYRK1A inhibitor for further investigations (Figure 4).

**Table 3:** Stability Data in Phase I MLMs and HLMs, m- and h-Plasma, and *S*_k_ of Hit Compound **1** and Hybrid Derivatives **27** and **28** ^a^

**cmpd #**	**MLM-Ph1,** ***t*_1/2_** **(min)** ^b^	**m-plasma,** ***t*_1/2_** **(min)** ^c^	**HLM-Ph1,** ***t*_1/2_** **(min)** ^b^	**h-plasma,** ***t*_1/2_** **(min)** ^c^	***S*** **_kinetic_ (**μM)
**1** (ARN25068)	>60	>120	>60^d^	>60^d^	<1
**27**	20 ± 1	>120	46 ± 4	>120	1 ± 0
**28** (ARN25699)	22 ± 1	>120	>60	>120	85 ± 30

a Results obtained from three independent experiments unless otherwise specified.

b Final cmpd conc.: 5 μM in microsomes + 0.1% DMSO.

c Final cmpd conc.: 2 μM in plasma + 0.5% DMSO.

d Obtained from two independent experiments.

**Figure 4:**
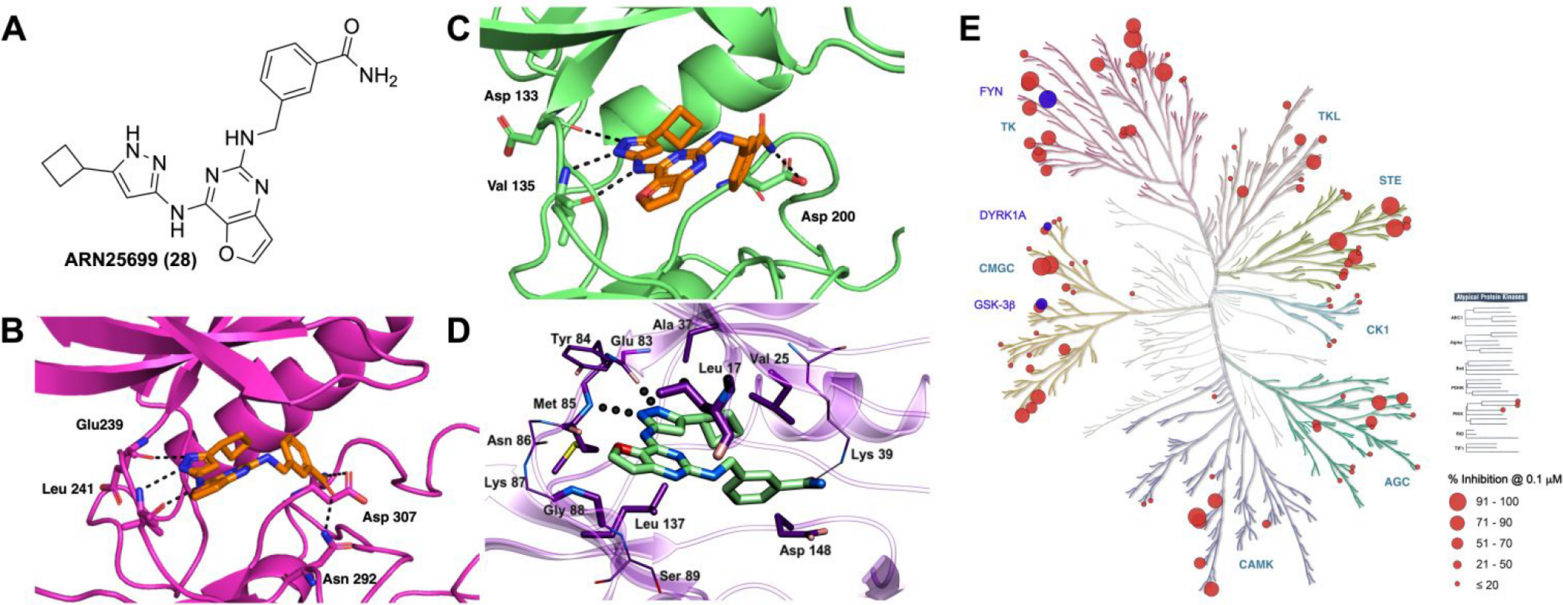
Structural and selectivity insights of compound **28**. (A) 2D structure of **28**. (B) X-ray crystal structure of **28** in complex with DYRK1A protein (PDB ID: 9FT2), black dotted lines indicate HB interactions. (C) X-ray crystal structure of **28** in complex with GSK-3β protein (PDB ID: 9FR9). (D) Docking pose of compound **28** in complex with FYN-α. (E) Kinome tree representation showing the percentage (%) inhibition against a panel of 100 selected PKs. The selectivity profile of compound **28** was determined using a radiometric assay (at 0.1 μM). Dots size reflects the level of inhibition as indicated in the legend. Raw data and comparison with compound **1** are provided in the Supporting Information (Tables S1 and S2, respectively). Illustration reproduced courtesy of Cell Signaling Technology, Inc. (www.cellsignal.com).

Co-crystal structures of **28** with DYRK1A and GSK-3β (PDB ID: 9FT2 and 9FR9, respectively, Figure 4B, C) highlighted the important role of the *m*-carbamoyl function in establishing additional HB interactions within the protein pockets. In the refined structure of **28** in complex with DYRK1A, the *m*-carbamoyl group engages in two additional HBs with the side-chains of Asn292 and Asp307 (Figure 4B). However, two alternative poses, with lower refinement statistics and poor electron densities, show interactions of the same moiety with either Lys188 or Asn244 side-chain and Glu291 main chain (Supporting Information, Figure S5). In the GSK-3β-**28** complex, rather than interacting with Lys85 (as shown for **16** in Figure 3A), which remains nearby (ca. 4.4 Å) and engaged in a salt bridge with Glu97 (not shown), the *m*-carbamoyl group forms an HB with the side-chain of Asp200 (Figure 4C). The predicted HBs formed by the aminopyrazole moiety of **28** within the hinge region of FYN-α (Glu83 and Met85) occur similarly to those in GSK-3β and DYRK1A (Figure 4D). Additionally, the *m*-carbamoyl group forms a HB with the amine of Lys39. The side chains of Leu17, Val25, Ala37, and Leu137 contribute significantly to favorable hydrophobic interactions.

Following the approach used to characterize compound **1**,^39^ the kinome selectivity of **28** was assessed at 0.1 and 10 μM using the KinaseProfiler platform based on a radiometric assay (Figure 4E and Supporting Information, Table S1). In this work, we expanded the profiling panel to 100 representative PKs. The selected kinases included proteins involved in tau protein phosphorylation (e.g., CDK5, CK1δ, MAPK1, CDK1, PKA) and kinases phylogenetically related to GSK-3β, FYN-α, and DYRK1A (e.g., GSK-3α, Yes, cSrc, DYRK1B, DYRK2, DYRK3), as well as a broader set of PKs not closely related to these families. Specifically, the panel was designed to cover kinases implicated in CNS disorders, particularly AD and neurodegeneration, beyond tau hyperphosphorylation, as well as kinases relevant to cancer and immune/inflammatory-related pathways. At 0.1 μM concentration, compound **28** displayed measurable activity across multiple kinase families, inhibiting 35 kinases by >50% and an additional 12 kinases by >30%, reflecting the broader inhibition spectrum often associated with ATP-competitive kinase inhibitors. Notably, a substantial fraction of the kinases inhibited by 50% have been implicated in AD-relevant pathogenic mechanisms, including tau phosphorylation and cell-cycle-related pathways (CDK5, CDK1/CDC2, CDK2, and MARK3),^40,41^ synaptic dysfunction and excitotoxic signaling (SRC-family kinases FYN/SRC/YES/LCK, and CaMK2α),^42,43^ neuroinflammation (SYK and ITK),^44,45^ and cellular stress responses (MST1, AMPKα1/α2, and PAK1).^46–48^ Inhibition of additional kinases linked to neuronal signaling and cytoskeletal regulation (LRRK2 and TNIK)^49,50^ further supports the engagement of multiple AD-associated pathways. Conversely, 44 kinases showed no detectable inhibition or only minimal inhibition (≤20%), including members of the CK1, MAPK (CMGC), AGC, STE, DMPK, and PIKK groups.

Within the 20-kinase subpanel shared with the profiling performed for compound **1**, compound **28** showed a generally more selective profile compared to compound **1** at 0.1 μM (Supporting Information, Table S2).

### In-Cell Tau Phosphorylation Assay

The microtubule (MT)-associated protein tau is the main component of the neurofibrillary tangles, aberrant structures that appear in the brains of AD patients and other tauopathies. Tau protein physiologically binds to and stabilizes MTs; however, in pathological conditions, it aggregates and loses its important functions. These tau aggregates are composed mainly of hyperphosphorylated and truncated forms of tau.^51^ To model this pathogenic process, the in vitro tau phosphorylation assay exploits the U2OS cell line stably expressing human triple mutant Tau-tGFP, which biochemically behaves as hyperphosphorylated tau, decreasing its ability to interact and stabilize MTs. On the other hand, kinase inhibitors or phosphatase activators promote tau binding to MTs and MT bundle formation.^52^

Based on their reported ability to reduce tau phosphorylation in different cell-based models, we selected SB216763,^53^ saracatinib,^54^ and harmine,^55^ three selective ATP-competitive inhibitors of GSK-3β, FYN, and DYRK1A, respectively. All three inhibitors were shown to modulate this end point, although their EC_50_ values vary as a function of assay conditions, cell type, and detection method.^32,56–62^

Considering the variability among previously reported cell-based models, we designed our experiments to evaluate these compounds, both individually and in combination, along with compound **28**, across a concentration range of 1–100 μM, to gain a more comprehensive assessment within our model system (Figure 5). In the combination experiment, for each tested dose, the ratio of each compound was 1:1:1, meaning that at the lowest concentration of 1 μM, each inhibitor was added at around 300 nM, allowing us to also explore the effect of a submicromolar concentration.

**Figure 5:**
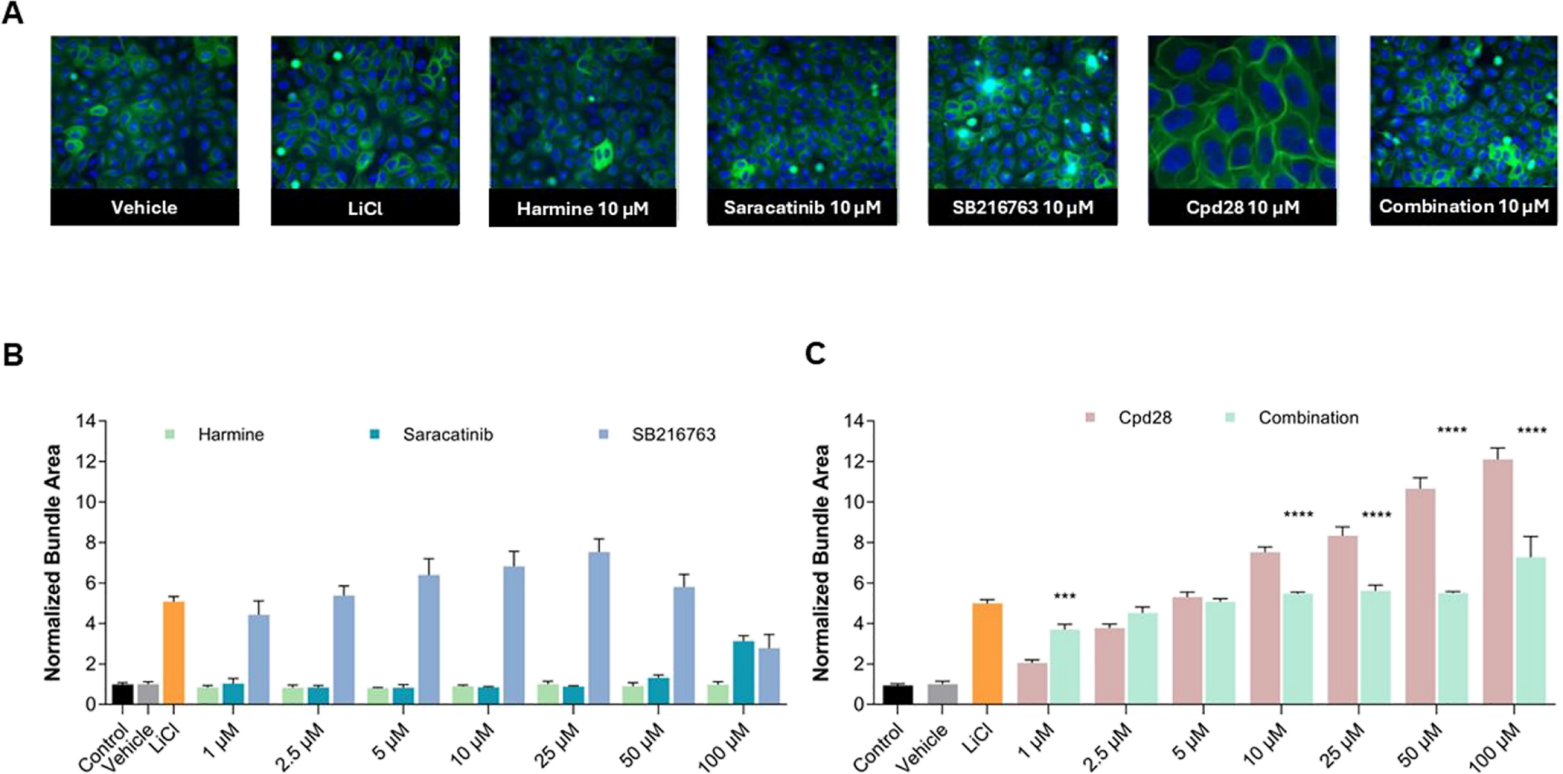
Tau phosphorylation assay on Tau0N4R-TM-tGFP U2OS cell line. (A) Representative images of cells treated with DMSO (vehicle control), LiCl – (positive control), and the tested compounds at 10 μM (20× magnification). (B) Dose–response relationship for harmine, saracatinib and SB216763 (1–100 μM). (C) Dose–response relationship for **28** and harmine/saracatinib/SB216763 combination (1–100 μM). Cells were treated with vehicle or compound at the indicated concentrations for 6 h. Data points represent the mean ± SD for each condition for a single experiment performed in technical triplicate. Results are expressed as the average of the total area of bundles per cell after treatment with the tested compounds, normalized respect to vehicle. Two-way ANOVA followed by the Bonferroni’s posthoc test (****p < 0.0001; ***p < 0.001; **28** vs combination).

All compounds and the SB216763/saracatinib/harmine combination were evaluated in the Tau0N4R-TM-tGFP U2OS stable cell line at seven concentrations over 6 h, using DMSO as the vehicle and LiCl as a positive control. The rationale for evaluating the combination was to explore potential synergistic effects and benchmark them against those elicited by compound **28**.

Treatment with harmine showed no detectable activity in the tau phosphorylation assay, whereas saracatinib was effective only at 100 μM, producing an approximately 3.1-fold increase in bundles compared to the vehicle (Figure 5B); however, at this concentration, the morphology of some nuclei indicates a certain degree of toxicity (data not shown).

The selective GSK-3β inhibitor SB216763 displayed a dose-dependent increase in tau bundle formation (up to 25 μM; Figure 5B) but caused a marked reduction in the number of nuclei at the three highest concentrations, indicating a certain degree of toxicity (data not shown). The combination of harmine, saracatinib, and SB216763 induced an effect comparable to SB216763 alone, albeit with lower intensity, and did not produce any synergistic activity (Figure 5C).

Compound **28** showed a clear dose-dependent increase in MT bundle formation, reaching a maximum 7.5-fold enhancement at 10 μM relative to the vehicle (Figure 5C). At 25, 50, and 100 μM concentrations, bundle formation further increased, although alterations in cell morphology suggested toxicity (data not shown). Notably, at 10 μM, compound **28** demonstrated superior efficacy, 1.4-fold higher, compared to the harmine/saracatinib/SB216763 combination.

### MetaID Studies

To obtain a comprehensive understanding of the metabolic pathways of compounds **1** and **28**, including both cytochrome P450- and noncytochrome P450-mediated processes, we carried out metabolite identification (MetID) studies in MLMs (Figure 6). Samples were analyzed by LC-MS/MS, and raw data were processed using MassMetaSite 4.5.0–2 in the WebMetabase application, encapsulated in the web platform Oniro version 1.4.2 (Mass-Analytica).^63,64^ Each compound was tested at 5 μM, and the percentage of metabolites formed was monitored at different incubation times (i.e., 0.5, 15, 30, and 60 min, see Supporting Information, Figure S9 for additional details).

**Figure 6:**
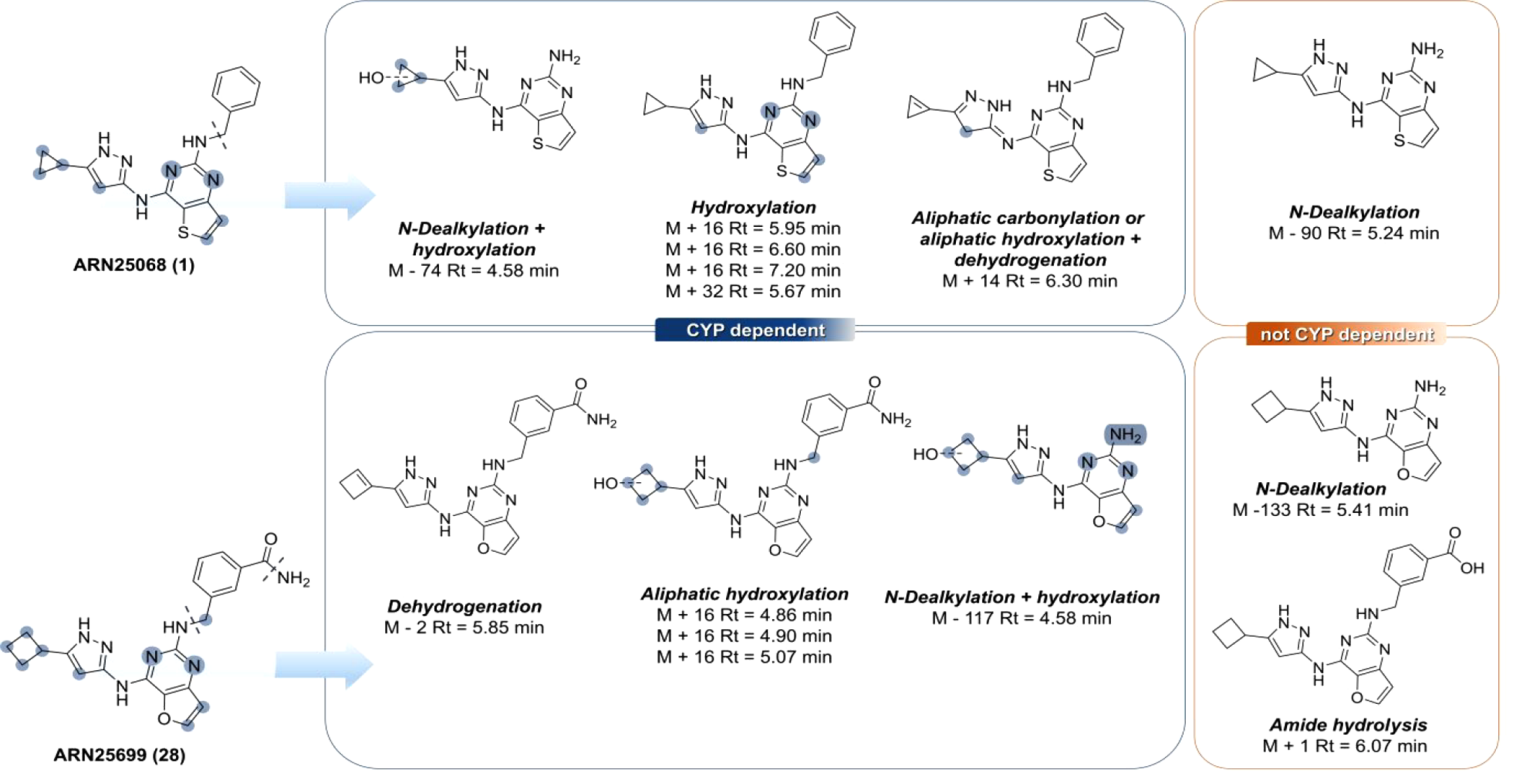
Experimental sites of metabolism for compounds **1** and **28**.

In contrast to the in vitro ADME profiling results, both compounds showed similar half-lives (*t*_1/2_ = 17 ± 4 min for **1** and *t*_1/2_ = 24 ± 1 min for **28**), with compound **28** showing slightly higher metabolic stability. Hydroxylation at both aromatic and aliphatic positions, either alone or in combination with *N*-dealkylation, dehydrogenation, or aliphatic carbonylation, represented the main cytochrome P450-mediated biotransformations (Figure 6). Notably, an aromatic hydroxylated metabolite (M + 16, Rt = 5.95 min, Figure 6) was identified as the most abundant product for compound **1** (Supporting Information, Figure S9A,B), whereas an aliphatic hydroxylated metabolite (M + 16, Rt = 5.07 min, Figure 6) was the predominant product observed for compound **28** (Figure S9D,E).

Remarkably, when both **1** and **28** were incubated in MLMs without NADPH to investigate non-CYP-mediated metabolism, we observed the formation of *N*-dealkylated metabolites (M – 90, Rt = 5.24 min for **1**, and M – 133, Rt = 5.41 min for **28**, Figure 6 and Supporting Information, Figure S9C), together with the more polar carboxylic acid metabolite of **28** (M + 1, Rt = 6.07 min, Figure 6 and Supporting Information, Figure S9F).

### In Vivo Evaluation of **1** and **28**

Encouraged by the favorable selectivity profile and the positive outcomes obtained in the in vitro tau phosphorylation assay, the in vivo pharmacokinetic properties of compound **28** were evaluated in CD1 mice following intravenous (i.v.) and oral *(*p.o.) administration at 3 and 10 mg/kg, respectively, in comparison to compound **1** (Figure 7).

**Figure 7:**
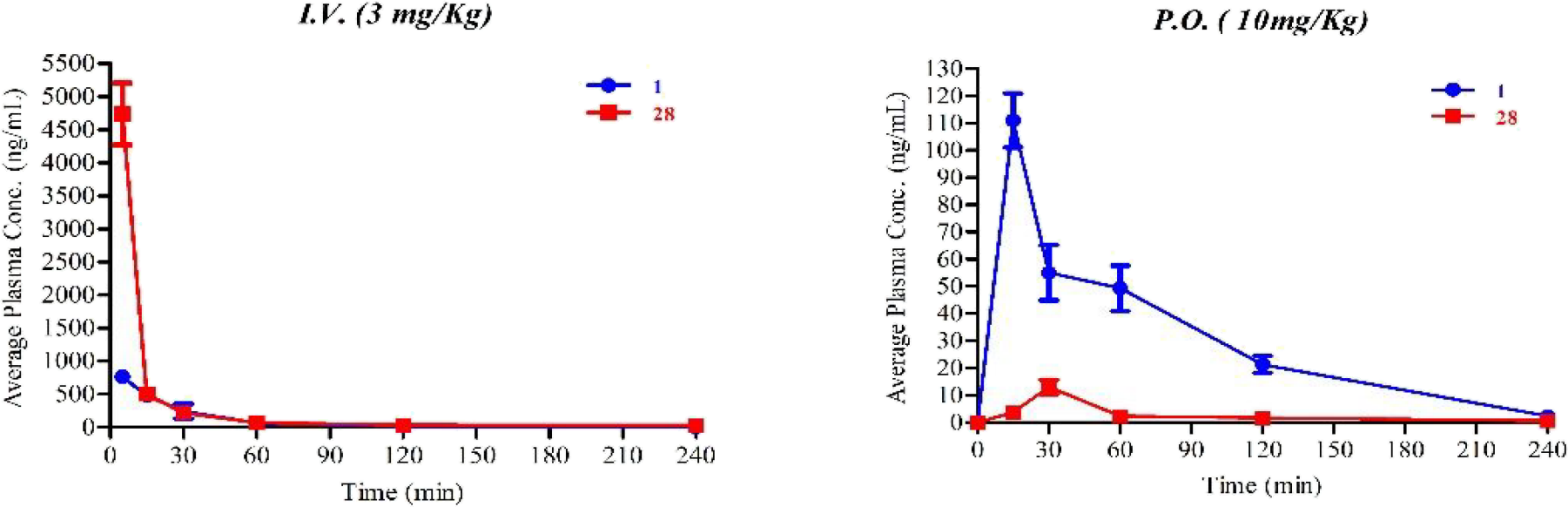
Pharmacokinetic profiles in mouse plasma of **1** and **28**. Route of administration: *i.v.* (3 mg/kg); *p.o.* (10 mg/kg).

Compound **1** showed a *C*_max_ = 762 ng/mL in plasma after 5 min by i.v. administration, still present after 4 h (4 ng/mL). However, via the same route, the compound also showed a steep decline within the first hour, with a half-life (*t*_1/2_) of 1 h. Following p.o. administration, a *C*_max_ of 111 ng/mL was reached after 15 min, and the compound remained detectable at 8 h (2 ng/mL). Overall, it exhibited very high clearance (CL = 1365 mL/min/kg). Oral bioavailability was calculated to be *F* = 9%, and low brain exposure was observed after i.v. administration, while negligible exposure was measured following *p.o.* administration (Tables 4 and 5).

**Table 4:** Pharmacokinetic Parameters of **1** (ARN25068) and **28** (ARN25699)

	**1** (ARN25068)		**28** (ARN25699)	
**pharmacokinetic parameters**	**i.v.**	**p.o.**	**i.v.**	**p.o.**
*C*_max_ (ng/mL)	762	111	4737	13
*T*_max_ (min)	5	15	5	30
AUC (min ng/mL)^a^	24,414	7177	54,854	623
*t*_1/2_ (min)	63	40	141	n.c.
*V*_D_ (L/kg)	11.0	79.0	10.0	n.c.
CL (mL/min/kg)	121	1365	50	n.c.
*F* (%)	9		n.c.	

a AUC was calculated based on *t* = 240 min. n.c. = not calculated

**Table 5:** Mouse PK Data of **1** and **28** in the Brain Following i.v. and p.o. Administration at 3 and 10 mg/kg, Respectively

	**1** (ARN25068)		**28** (ARN25699)	
	**i.v.**	**p.o.**	**i.v.**	**p.o.**
**time points** (min)	**ng/mg brain** ^a^			
0		0.00 ± 0.00		<LOQ^b^
5	0.71 ± 0.47		0.25 ± 0.31	
15	0.3 ± 0.08	0.04 ± 0.02	0.03 ± 0.01	<LOQ^b^
30	0.15 ± 0.13	0.02 ± 0.01	0.02 ± 0.01	<LOQ^b^
60	0.03 ± 0.01	0.02 ± 0.01	<LOQ^b^	<LOQ^b^
120	0.00 ± 0.00	<LOQ^b^	<LOQ^b^	<LOQ^b^
240	0.00 ± 0.00	<LOQ^b^	<LOQ^b^	<LOQ^b^
480		<LOQ^b^		<LOQ^b^

a Normalized by 100 mg brain/mL.

b Limit of quantification (LOQ) – 5 nM.

On the other hand, **28** showed a relatively higher *C*_max_ = 4737 ng/mL in plasma 5 min after *i.v.* administration, and it was still detectable after 4 h (28 ng/mL). Although its i.v. profile declined rapidly within the first 15 min, a longer *t*_1/2_ (>2 h) was observed. However, the compound showed very poor exposure in plasma following p.o. administration, with *C*_max_ of 13 ng/mL observed after 30 min, and only low levels of **28** were detected in the brain after both i.v. and p.o. administration (Tables 4 and 5).

Overall, the in vivo PK studies confirmed suboptimal drug-like properties for both compounds: (i) low oral bioavailability and (ii) low brain exposure after oral administration, with compound **1** exhibiting a better profile, although with reduced water solubility. The limited stability against metabolic reactions of phase 1 when incubated with MLMs has likely reduced the oral bioavailability of both compounds. Moreover, the low brain exposure after oral administration was consistent with the low BBB scores calculated for each compound according to the method described by Gupta et al.^65^ (3.36 for **1** and 1.44 for **28**), considering that a BBB score of ∼4 is generally regarded as the lower threshold for acceptable BBB permeability. The largest difference between the two molecules lies in their polar surface area (PSA) values, with compound **28** exhibiting a substantially higher polar surface area than compound **1** (188.67 vs 98.83 Å^2^). Such high polarity is expected to significantly hinder passive diffusion across the BBB, as compounds with PSA values below 70–80 Å^2^ are typically considered within the acceptable range for CNS penetration. These data suggest the need for further chemical refinement of compound **28** to enhance brain exposure and PK properties of this chemotype.

### Design of **31** (ARN26646)

The research of ligands with improved affinity for DYRK1A was the initial driving force toward the development of balanced inhibitors with concurrent activity against GSK-3β/FYN-α and DYRK1A. For this reason, the focus was on a literature review of known ligands that selectively interact with DYRK1A. Notable examples include the selective inhibitor **29**,^66^ and the dual GSK-3β/DYRK1A inhibitor **30**,^67^ an antidiabetic drug candidate developed by the Novartis research foundation, which showed 83% inhibitory activity at 10 μM against FYN-α (Figure 8A).

**Figure 8:**
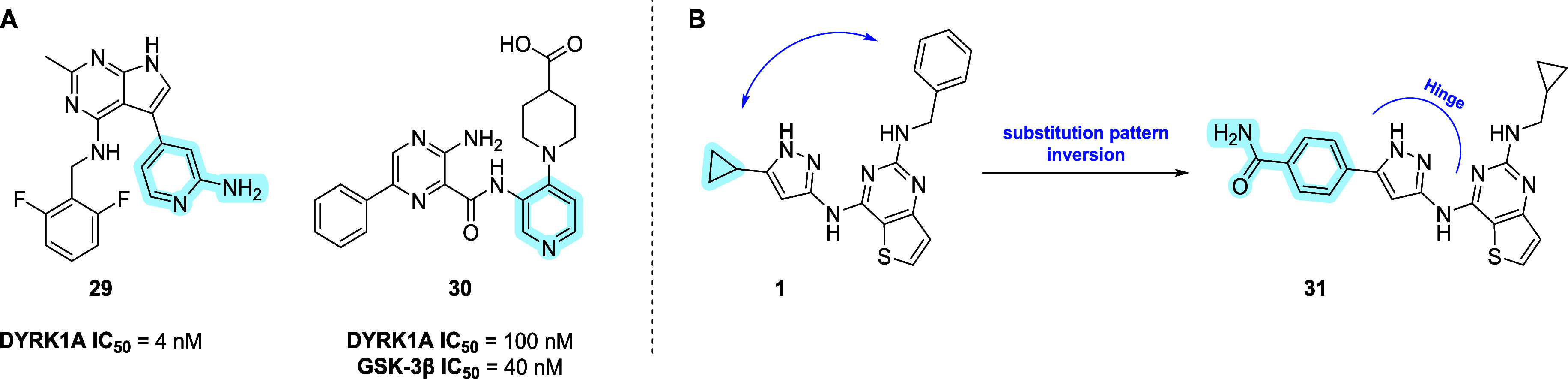
(A) Structure and inhibitory activity of **29** and **30**. (B) Chemical substitution pattern inversion and design of compound **31**.

Our attention focused on the design of compound **31** (ARN26646, Figure 8B), characterized by an inversion of the chemical substitution pattern on the pyrazole and at the C2 position of the 2,4-dichloropyrimidine core.

Compounds **1**, **29**, **30**, and **31** were docked in the DYRK1A binding pocket and superimposed (Supporting Information, Figure S6) as the ultimate goal to identify common pharmacophoric features and gain insights into possible more drastic modifications of compound **1**. Notably, an additional HB interaction with Lys188 within the binding pocket of the enzyme was confirmed not only for **29** and **30** as reported in the literature but also predicted for **31**. A strong hydrophobic interaction with the gatekeeper Phe238 was also observed for compounds **29**, **30**, and **31**, along with HBs with Glu239 and Leu241.

Remarkably, compound **31** showed a quite well-balanced nanomolar activity against all three targets, exhibiting the highest inhibitory potency against DYRK1A (IC_50_ = 199 nM, Table 6), likely due to two additional HB interactions engaged with Glu291 and Lys188 of this target (Supporting Information, Figure S6D). Furthermore, although compound **31** exhibited lower kinetic solubility than compound **1**, it displayed favorable in vitro ADME properties (Table 6), including greater stability in MLMs than compound **28**. However, its calculated BBB score^65^ (1.36), together with its high PSA (182.58 Å^2^), suggests poor BBB permeability. These results make compound **31** an optimal starting point for further optimization campaigns aimed at assessing the effects of more radical structural variations on the original scaffold, thereby opening opportunities for the design of novel balanced inhibitors with improved drug-likeness.

**Table 6:** GSK-3β, FYN-α and DYRK1A Inhibition and Stability Data in MLMs and HLMs (Ph1), m- and h-Plasma, and *S*_k_ of Compound **31**

	**31** (ARN26646)^a^
GSK-3β IC_50_ (nM)	125.5 ± 5.0
FYN-α IC_50_ (nM)	83.0 ± 11.8
DYRK1A IC_50_ (nM)	198.6 ± 10.7
MLM-Ph1 *t*_1/2_ (min)^b^	54 ± 3
m-plasma *t*_1/2_ (min)^c^	>120
HLM-Ph1 *t*_1/2_ (min)^b^	45 ± 7
h-plasma *t*_1/2_ (min)^c^	>120
*S*_kinetic_ (μM)	<1

a Results obtained from three or more independent experiments.

b Final cmpd conc.: 5 μM in microsomes + 0.1% DMSO.

c Final cmpd conc.: 2 μM in plasma + 0.5% DMSO.

## Chemistry

Compounds described in this article were prepared in good yields and at a low cost of the process, by applying the mild and scalable chemical route previously employed for the synthesis of **1** consisting of two sequential SN_Ar_ reactions on the 2,4-dichloro­[3,2-*d*]­pyrimidin-fused heterocycle (Scheme 1).

**Scheme 1:**
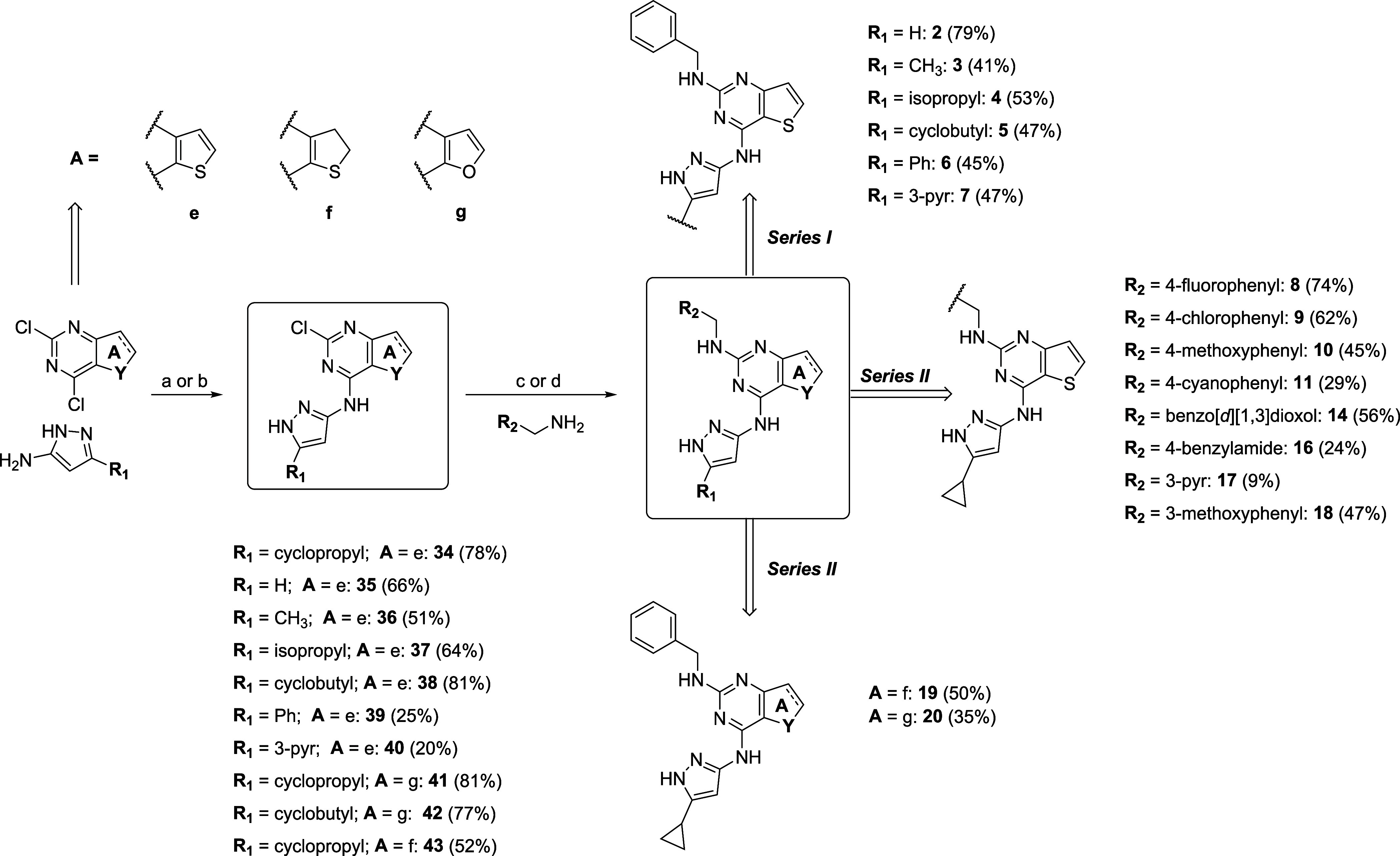
Synthesis of Compounds **2–11**, **14**, and **16–20** (Series I–II–III)^a^

a Reagents and conditions. (a) Et_3_N, rt, 2–5 days; (b) 2-propanol, Et_3_N, rt or 50 °C, 2–5 days; (c) *n*-BuOH, DIPEA, 180 °C, 4–6 h, MW, Ar; (d) *n*-BuOH, 110 °C, 2 days, Ar.

In all cases, the general chemical procedure involved an initial chemoselective amination at the 4-position of the 2,4-dichloropyrimidine ring by employing the commercially available aminopyrazole derivatives and triethylamine as a base reaction, either under neat conditions or in the presence of 2-propanol as a solvating agent, at room temperature. The less reactive 3-pyridine-substituted pyrazole required an increase in the reaction temperature up to 50 °C. Successively, the resultant 2-chloro-*N*-(5-alkyl/cycloalkyl/aryl/heteroaryl-1*H*-pyrazol-3-yl)­heteroaryl­[3,2-*d*]­pyrimidin-4-amines **34–43** were treated through harsher experimental conditions under conventional heating (110 °C) with a large excess of the appropriate benzylamine (5 equiv), affording compounds **2–10** in moderate to good yields. Optimization of the same chemical protocol under MW irradiation significantly shortened the reaction times from 72 to 6–8 h and reduced the amount of the appropriate benzylamine from 5 to 1.5 equivalents. The use of DIPEA as a non-nucleophilic base also facilitated the purification process (compounds **11–28**; Schemes 1–4). Finally, displacement of the chloro-substituent at C2 of the appropriate 4-substituted intermediates **34**, **37**, **38**, and **4** **1** in the presence of sodium benzyloxide under MW irradiation (Scheme 2) yielded the corresponding benzyloxy analogs **15**, and **21–23**.

**Scheme 2:**
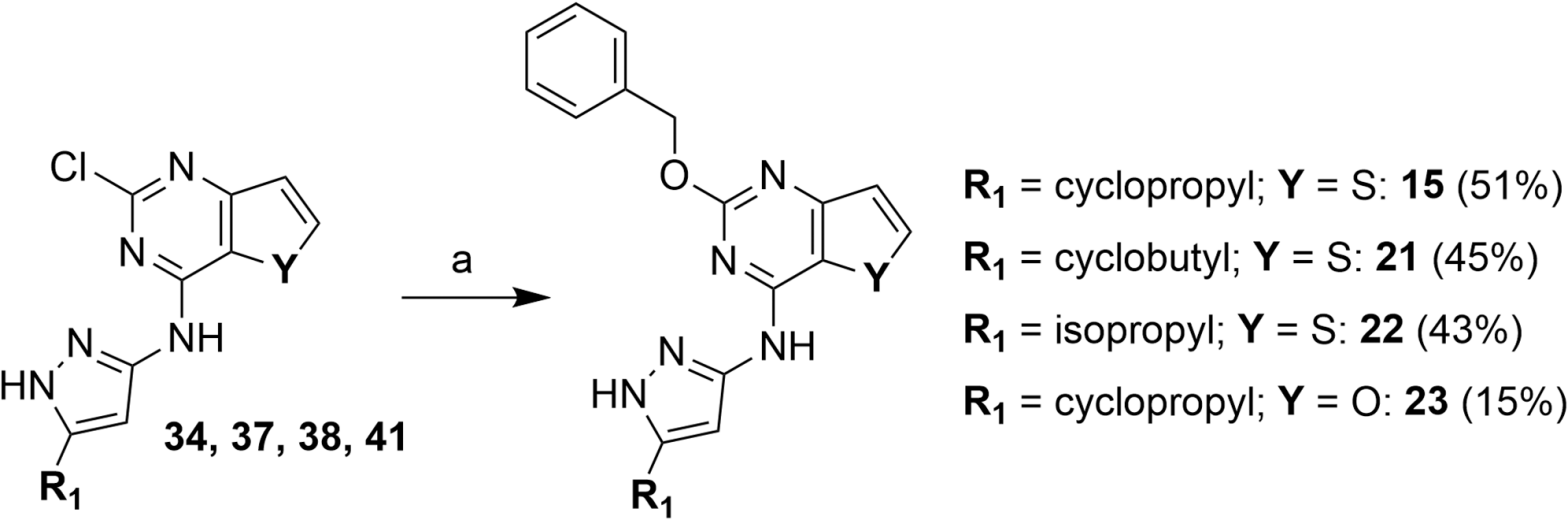
Synthesis of Benzyloxy Derivatives^a^

a Reagent and conditions. (a) Sodium benzyloxide, THF, 150 °C, 7 h, MW, Ar.

**3:**
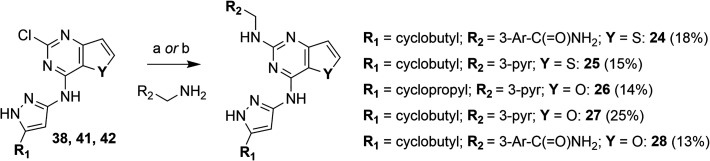
Preparation of Benzylamino Hybrid Derivatives^a^

a Reagents and conditions. *n*-BuOH, DIPEA, 180 °C, 4–8 h, MW, Ar.

Chemoselective protection of the commercially available 4-hydroxybenzylamine at the hydroxyl function with *tert*-butyl­(chloro)­diphenylsilane, followed by an SN_Ar_ reaction between the corresponding TBDMS-protected benzylamine **44** and **34**, allowed access to intermediate **45**. A final cleavage of the TBDPS group under mild conditions using KHF_2_ ^68^ at room temperature afforded the title derivative **12** (Scheme 4) in good yield. The 4-(aminomethyl)­benzamide (**47**) to prepare derivative **13** was, in turn, synthesized *via* a two-step procedure as depicted in Scheme 4: (1) treatment of the corresponding carboxylic acid with di-*tert*-butyl dicarbonate in the presence of NH_4_HCO_3_; and (2) cleavage of the *N*-Boc protecting group under acid conditions using TFA. Lastly, a SN_Ar_ reaction between **47** and **34** provided compound **13** in good yield.

**Scheme 4:**
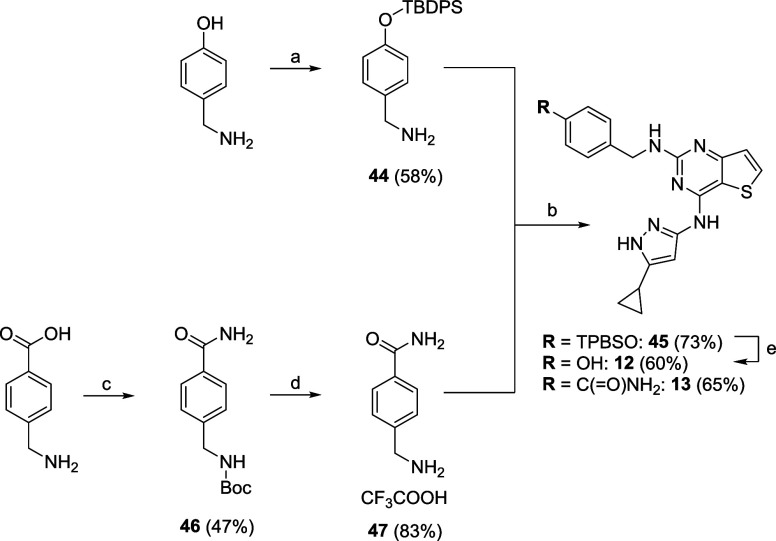
Multi-Step Synthesis of Derivatives **12** and **13** ^a^

a Reagent and conditions. (a) TBDPS-Cl, imidazole, dry THF, 10 h, Ar; (b) *n*-BuOH, DIPEA, 180 °C, 4–6 h, MW, Ar; (c) (Boc)_2_O/NH_4_HCO_3_, pyridine, dry 1,4-dioxane, 105 °C, 3 h, MW, Ar; (d) TFA, CH_2_Cl_2_, 0 °C to rt, 1.5 h, Ar; (e) KHF_2_, MeOH, rt, on.

The preparation of compound **31** was performed via a four-step synthetic procedure reported in Scheme 5. The gram scale insertion of a hydroxyl moiety at the more reactive C4 position of the pyrimidine ring in 2,4-dichlorothieno­[3,2-*d*]­pyrimidine proceeded in quantitative yield (**48**), opening the possibility to direct the first SN_Ar_ reaction toward the less reactive C2 position of **48**. Reaction with the commercially available 1-cyclopropanemethylamine under MW irradiation at 160 °C afforded **49**, which was then subjected to a chlorination reaction in the presence of phosphorus oxychloride to give **50**. A subsequent SN_Ar_ reaction with the in-house prepared 5-substituted pyrazole **51**, in turn synthetized via a Suzuki cross-coupling reaction between the 3-bromo-1*H*-pyrazol-5-amine and the (4-carbamoylphenyl)­boronic acid, afforded the desired product as the formic acid salt (**52**) in good yield after HPLC purification. Final purification of the same compound via strong cation exchange (SCX) chromatography gave **31** as the free base.

**Scheme 5:**
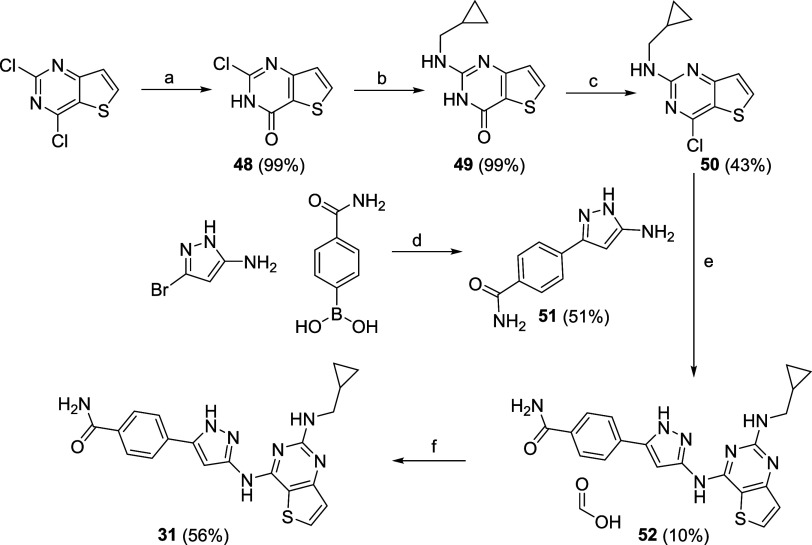
Multi-Step Procedure To Access to Compound **31** ^a^

a Reagents and conditions. (a) (i) NaOH, THF/water, 60 °C on; (ii) acetic acid, 35 °C, 2 h; (b) 1-cyclopropylmethanamine, DIPEA, *n*-BuOH, 6 h, 160 °C, MW, Ar; (c) POCl_3_, DMSO (cat.), 105 °C, 3 h, Ar; (d) 3-bromo-1*H*-pyrazol-5-amine, (4-carbamoylphenyl)­boronic acid, Pd­(PPh_3_)_4_, dry 1,4-dioxane, K_2_CO_3_ aq (2 M), MW, 130 °C, 2 h, Ar; (e) *n*-BuOH, DIPEA, 180 °C, 8 h, Ar; (f) SCX separation.

## Conclusions

The multifactorial etiology of tau-related disorders remains a significant challenge in developing disease-modifying therapies. By leveraging the MTDL approach and the high versatility of our previously reported GSK-3β/FYN/DYRK1A triple-targeting inhibitor **1** (ARN25068), we demonstrated, through a combination of computational modeling and X-ray crystallography-assisted SAR studies, that the initial unbalanced activity of **1** against GSK-3β, FYN-α, and DYRK1A can be optimized. This led to the development of compounds **27** and **28** (ARN25699) with significantly improved potency toward DYRK1A (IC_50_ values of 462 and 242 nM, respectively), while maintaining low nanomolar potency against GSK-3β and FYN-α. Among these, compound **28** exhibited a favorable ADME profile and acceptable pharmacokinetic properties, albeit with limited brain exposure following intravenous and oral administration. Furthermore, consistent with the behavior of ATP-competitive kinase inhibitors, compound **28** displayed a broader kinome inhibition profile at 0.1 μM, inhibiting a subset of kinases implicated in AD-relevant pathogenic mechanisms beyond tau phosphorylation.

Notably, the same compound showed a dose-dependent effect in the in vitro tau phosphorylation assay, with a maximum 7.5-fold increase in tau bundle formation at 10 μM compared to the negative control. At this concentration, its efficacy was also superior to that of the SB216763/saracatinib/harmine combination, with a 1.4-fold greater effect.

Structural modifications inspired by our earlier analogs enabled the rational design of compound **31** (ARN26646), which achieved a well-balanced nanomolar inhibition against three PKs, GSK-3β, FYN-α, and DYRK1A (IC_50_ = 126, 83, and 199 nM, respectively), alongside good ADME properties.

These findings provide a strong foundation for further medicinal chemistry efforts to refine our lead compounds, particularly by enhancing drug-like properties, BBB permeability, and kinase selectivity profile, while preserving the desired potency. Given the central role of GSK-3β, FYN-α, and DYRK1A in tau-related pathologies, our approach offers a compelling and high-potential avenue for the development of disease-modifying therapies for AD and other tauopathies. The optimization strategies outlined in this study, which led to disclosing multikinase inhibitors with concurrent activity against GSK-3β, FYN, and DYRK1A, a previously unexplored kinase combination, pave the way for a new generation of molecules with enhanced efficacy and translational potential for the treatment of neurological disorders.

## Experimental Section

### Materials and Methods

Solvents and reagents were purchased from commercial suppliers (Acros, Aldrich, Merck, Fluorochem, TCI, or Alfa Aesar) and used without further purification. Dry solvents were purchased from Sigma-Aldrich. Thin-layer chromatography analyses were performed using precoated Supelco silica gel on TLC Al foils, 0.2 mm, and visualized by UV (254 nm). Automated column chromatography purifications were done using a Teledyne ISCO apparatus (CombiFlash Rf) with prepacked silica gel or basic alumina columns of different sizes (from 4 g up to 48 g) and mixtures of increasing polarity of cyclohexane and ethyl acetate (EtOAc), chloroform (CHCl_3_) or dicloromethane (CH_2_Cl_2_) and methanol (MeOH) or Ethanol (EtOH) enriched with NH_3_ 1 M solution, were specified.

NMR experiments were run on a Bruker Avance III 400 system, equipped with a BBI probe and Z-gradients, and on a Bruker UltrashieldTM Plus FT-NMR 600 MHz Avance III, equipped with a CryoProbeTM QCI 1H/19F/13C/15N and with a SampleJet autosampler and temperature control. Spectra were acquired at 300 K, using deuterated dimethyl sulfoxide (DMSO-*d*_*6*_), deuterated chloroform (CDCl_3_), or acetone (Acetone-*d*_6_) as solvents. Chemical shifts for ^1^H and ^13^C spectra were recorded in parts per million using the residual nondeuterated solvent as the internal standard (for DMSO-*d*_*6*_: 2.50 ppm, ^1^H; 39.52 ppm, ^13^C). Data are reported as follows: chemical shift (ppm), multiplicity (indicated as: br., broad signal; s, singlet; d, doublet; t, triplet; dd, doublet of doublets, dq, doublet of quartets; m, multiplet and combinations thereof), coupling constants (*J*) in Hertz (Hz), and integrated intensity.

Not all ^13^C signals were detectable for compounds **2**–**7**, **8–11**, **12–18**, **19**–**27**, **28** (ARN25699), **31** (ARN26646), and **52,** despite multiple attempts with exhaustive signal averaging. The missing signals are likely attributable to the long relaxation times of tertiary and quaternary carbons in systems where two tautomeric species are present in different abundances.

UPLC-MS analyses were run on a Waters ACQUITY UPLC-MS system consisting of a single quadrupole detector (SQD) mass spectrometer equipped with an electrospray ionization interface (ESI) and a photodiode array detector (PDA) from Waters Inc. (Milford, MA, USA). Electrospray ionization in positive and negative mode was applied in the mass scan range 100–750 Da. The PDA range was 210–400 nm. The analyses were performed on an ACQUITY UPLC BEH C_18_ (50 × 2.1 mm ID, particle size 1.7 μm) with a VanGuard BEH C_18_ precolumn (5 × 2.1 mm ID, particle size 1.7 μm). The mobile phase was 10 mM NH_4_OAc in H_2_O at pH 5 adjusted with AcOH (A) and 10 mM NH_4_OAc in MeCN-H_2_O (95:5) at pH 5 (B). Three types of gradients were employed depending on the polarity of the compounds: an a polar method where the mobile phase B was increased from 50 to 100% in 2.5 min, a generic method where the mobile phase B was increased from 5 to 95% in 2.5 min and a polar method where the mobile phase B was increased from 0 to 50% in 2.5 min.

The quality control (QC) analysis of the final compound was performed on a Waters ACQUITY UPLC-MS system as defined above, starting from a 10 mM stock solution in DMSO-*d*_6_ and further diluted 20-fold with MeCN-H_2_O (1:1) for analysis. The analysis was run on an ACQUITY UPLC BEH C_18_ (100 × 2.1 mm ID, particle size 1.7 μm) with a VanGuard BEH C_18_ precolumn (5 × 2.1 mm ID, particle size 1.7 μm), using 10 mM NH_4_OAc in H_2_O at pH 5 adjusted with AcOH (A) and 10 mM NH_4_OAc in MeCN-H_2_O (95:5) at pH 5 (B) as mobile phase. A linear gradient was applied starting from 10% of the mobile phase B, holding for 0.20 min. Then increasing B from 10 to 90% in 6 min 90–100% in 0.10 min and 100% hold for 0.70 min with a total run time of 7 min. All final compounds were found to be >95% pure by UPLC-MS purity (UV at 215 nm).

Compounds were named according to IUPAC nomenclature using the naming algorithm developed by CambridgeSoft Corporation and used in ChemDraw professional 20.0.

#### General Procedure A: SN_Ar_ at the C4 of the Pyrimidine Core (**34–37**, **39**)

Triethylamine (2.2 equiv) was added to a mixture of 2,4-dichlorosubstituted [3,2-*d*]­pyrimidine (1 equiv) and the appropriate 5-substituted aminopyrazole (2.5 equiv). The resulting mixture was allowed to react at room temperature for 2 days under an inert atmosphere (Ar). After complete conversion of the starting material, the reaction mixture was precipitated with water (5 mL) and filtered under *vacuum* to afford the desired products. The synthesis and characterization of intermediate **34** were previously reported by Demuro et al.^39^

#### General Procedure B: SN_Ar_ at the C4 of the Pyrimidine Core (**38**, **40**, **41**–**43**)

2,4-Dichloro-substituted­[3,2-*d*]­pyrimidine (1 equiv), the appropriate 5-substituted aminopyrazole (2.5 equiv), and triethylamine (2.2 equiv) were suspended in anhydrous 2-propanol (4 mL). The reaction mixture was stirred for 2–3 days at room temperature or, where otherwise specified, at 80 °C, under Ar. After complete conversion of the starting material, the reaction mixture was precipitated with water (5 mL), filtered under *vacuum,* and the obtained solid was either used in the following step without any further purification or purified *via* normal phase flash column chromatography, as specified.

#### General Procedure C: SN_Ar_ at the C2 of the Pyrimidine Core (**2–4**, **6, 8**–**10**)

To intermediates **34–37**, **39** (1 equiv), the appropriate benzylamine (5 equiv) was added. *N*-butanol (0.7 mL) was added, and the reaction mixture was heated at 110 °C for 2 days under Ar. After observing the complete conversion of the starting material, the reaction mixture was cooled down to room temperature and concentrated to dryness. The resulting crude was purified by normal phase flash chromatography to obtain the desired products.

#### General Procedure D: SN_Ar_ at the C2 of the Pyrimidine Core (**5**, **7**, **11**, **45**, **13**, **14**, **16–19**, **20**, **24**)

To intermediates **34**, **38**, **40**–**43** (1 equiv), the appropriate benzylamine (1.5 equiv), DIPEA (2.5 equiv), and *n*-butanol (1.37 mL) were added. The resulting suspension was sonicated and heated for 6–8 h under MW irradiation at 180 °C, under Ar. After observing complete conversion of the starting material, the reaction mixture was cooled down to room temperature and concentrated to dryness. The resulting crude was purified by normal-phase flash chromatography.

#### General Procedure E: Synthesis of Benzyloxy Derivatives (**15**, **21–23**, 25-28)

To the appropriate 4-substituted bicyclic-pyrimidine (**34**, **37**, **38**, **41**) (1 equiv), sodium benzyloxide solution (1 M, 1.1 equiv) was added. The resulting mixture was suspended in anhydrous THF (2.7 mL) and heated under MW irradiation for 7 h at 150 °C, under Ar. After completion, the reaction was concentrated in vacuo, and the resulting solid was washed with water (10 mL × 3) and extracted with EtOAc (10 mL × 3). The organic layers were combined and dried over Na_2_SO_4_, filtered and concentrated under *vacuum*. The resulting crude products were purified by normal phase flash chromatography to yield the title compounds.

##### *N*2-Benzyl-*N*4-(1*H*-pyrazol-3-yl)­thieno­[3,2-*d*]­pyrimidine-2,4-diamine (**2**)

Compound **2** was synthesized following the general procedure C, starting from 80.0 mg (0.318 mmol) of **35** and 1.589 mmol of benzylamine (0.174 mL) dissolved in *n*-butanol (0.060 mL). Purification by normal phase flash chromatography employing a 12 g gold silica cartridge (Solvent A: CH_2_Cl_2_ – Solvent B: CH_2_Cl_2_/MeOH 9:1 – Detection: 260/286 nm – Gradient: 5–20% Solvent B) and a final trituration with CH_2_Cl_2_ (7 mL) yielded the desired product as a white solid (81.1 mg, 79%). UPLC-MS (generic method): Rt = 1.66 min, MS (ESI) *m*/*z*: 323.1 [M + H]^+^, C_16_H_15_N_6_S^+^ [M + H]^+^ calculated: 323.1. QC analysis: Rt = 3.09 min, UPLC-MS purity (UV at 215 nm): 99.5%. Both ^1^H and ^13^C NMR spectra in DMSO-*d*_6_ were consistent with the isolation of two different tautomeric forms, a and b, in a dynamic equilibrium, with a corresponding to the major and b to the minor in abundance. ^1^H NMR (600 MHz, DMSO-*d*_6_) δ 13.05 (br. s, 1Hb), 12.35 (br. s, 1Ha), 10.42 (br. s, 1Hb), 9.72 (br. s, 1Ha), 7.91 (br. s, 1Hb and 1Ha), 7.61 (br. s, 1Hb and 1Ha), 7.59–6.49 (m, 7Hb and 7Ha), 6.70 (br. s, 1Ha), 5.95 (br. s, 1Hb), 4.54 (d, *J* = 6.1 Hz, 2Ha and 2Hb). ^13^C NMR (151 MHz, DMSO-*d*_6_) δ 162.45, 160.73, 160.60, 154.97, 147.50, 141.13, 140.32, 138.00, 133.31, 128.16, 127.16, 127.11, 127.06, 126.43, 123.04, 105.55, 98.81, 91.81, 44.32.

##### *N*2-Benzyl-*N*4-(5-methyl-1*H*-pyrazol-3-yl)­thieno­[3,2-*d*]­pyrimidine-2,4-diamine (**3**)

Compound **3** was synthesized following the general procedure C, starting from 66 mg (0.248 mmol) of **36** and 1.242 mmol of benzylamine (0.14 mL) dissolved in 0.81 mL of *n*-butanol. Purification by normal phase flash chromatography employing a 12 g gold silica cartridge (Solvent A: CH_2_Cl_2_ – Solvent B: CH_2_Cl_2_/MeOH 9:1 – Detection: 240/260 nm – Gradient: 0–40% Solvent B) followed by a sequential trituration with cold diethyl ether (7 mL) yielded the desired product as a white solid (34.1 mg, 41%). UPLC-MS (generic method): Rt = 1.79 min, MS (ESI) *m*/*z*: 337.1 [M + H]^+^, C_17_H_17_N_6_S^+^ [M + H]^+^ calculated: 337.1. QC analysis: Rt = 3.21 min, UPLC-MS purity (UV at 215 nm): 99.5%. ^1^H spectrum in DMSO-*d*_6_ was consistent with the isolation of two different tautomeric forms, a and b, in a dynamic equilibrium, with a corresponding to the major and b to the minor in abundance. ^1^H NMR (600 MHz, DMSO-*d*_6_) δ 12.62 (br. s, 1Hb), 12.00 (br. s, 1Ha), 10.29 (br. s, 1Hb), 9.59 (br. s, 1Ha), 7.88 (br. s, 1Ha), 7.68–6.94 (m, 7Ha and 8Hb), 6.41 (br. s, 1Ha), 5.75 (s, 1Hb), 4.53 (d, *J* = 6.2 Hz, 2Ha and 2Hb), 2.18 (br. s, 3Ha and 3 Hb). ^13^C NMR (151 MHz, DMSO-*d*_6_) δ 162.40, 160.71, 154.91, 147.66, 141.21, 137.87, 137.82, 133.20, 128.11, 126.97, 126.90, 126.30, 123.49, 123.01, 122.94, 105.57, 98.15, 44.30, 10.80.

##### *N*2-Benzyl-*N*4-(5-isopropyl-1*H*-pyrazol-3-yl)­thieno­[3,2-*d*]­pyrimidine-2,4-diamine (**4**)

Compound **4** was synthesized following the general procedure C, starting from 74.3 mg (0.253 mmol) of **37** and 1.265 mmol of benzylamine (0.14 mL) dissolved in 0.843 mL of *n*-butanol. Purification by normal phase flash chromatography employing a 12 g gold silica cartridge (Solvent A: CH_2_Cl_2_ – Solvent B: CH_2_Cl_2_/1N NH_3_ in MeOH 9:1 – Detection: 240/260 nm – Gradient: 0.25–35% Solvent B) and a final trituration by using cold diethyl ether (7 mL) yielded the desired product as a white solid (49.1 mg, 53%). UPLC-MS (generic method): Rt = 1.96 min, MS (ESI) *m*/*z*: 365.2 [M + H]^+^, C_19_H_21_N_6_S^+^ [M + H]^+^ calculated: 365.2. QC analysis: Rt = 3.93 min, UPLC-MS purity (UV at 215 nm): 99.5%. ^1^H NMR (600 MHz, DMSO-*d*_6_) δ 12.04 (br. s, 1H), 9.59 (br. s, 1H), 7.88 (br. s, 1H), 7.48–6.91 (m, 6H), 6.45 (br. s, 1H), 4.56 (d, *J* = 6.3 Hz, 2H), 2.87 (br. s, 1H), 1.19 (d, *J* = 6.9 Hz, 6H). ^13^C NMR (151 MHz, DMSO-*d*_6_) δ 162.44, 160.78, 154.94, 149.06, 147.29, 141.24, 140.30, 133.25, 128.12, 126.92, 126.33, 122.91, 105.48, 95.50, 44.25, 25.46, 22.36.

##### *N*2-Benzyl-*N*4-(5-cyclobutyl-1*H*-pyrazol-3-yl)­thieno­[3,2-*d*]­pyrimidine-2,4-diamine (**5**)

Compound **5** was synthesized following the general procedure D, starting from 100 mg (0.327 mmol) of intermediate **38**, benzylamine (0.053 mL, 0.490 mmol), and DIPEA (0.142 mL, 0.817 mmol) dissolved in 1.63 mL of *n*-butanol and stirred at 180 °C for 8 h. After completion, the reaction mixture was concentrated in vacuo, and the resulting crude product was purified by normal phase flash chromatography employing a 12 g gold silica cartridge (Solvent A: CHCl_3_ – Solvent B: CHCl_3_/EtOH 8:2 – Detection: 240/260 nm – Gradient: 5–10% Solvent B). The isolated fraction was concentrated under high *vacuum* and lyophilized to yield the desired product as a white solid (57.51 mg, 47%). UPLC-MS (generic method): Rt = 2.04 min, MS (ESI) *m*/*z*: 377.0 [M + H]^+^, C_20_H_21_N_6_S^+^ [M + H]^+^ calculated: 377.1. QC analysis: Rt = 4.03 min, UPLC-MS purity (UV at 215 nm): 99.5%. Both ^1^H and ^13^C NMR spectra in DMSO-*d*_6_ were consistent with the isolation of two different tautomeric forms, a and b, in a dynamic equilibrium, with a corresponding to the major and b to the minor in abundance. ^1^H NMR (400 MHz, DMSO-*d*_6_) δ 12.66 (br. s, 1Hb), 12.06 (br. s, 1Ha), 10.30 (br. s, 1Hb), 9.64 (br. s, 1Ha), 7.90 (br. s, 1Ha and 1Hb), 7.42–6.87 (m, 7Ha and 7Hb), 6.51 (br. s, 1Ha), 5.79 (br. s, 1Hb), 4.57 (d, *J* = 6.2 Hz, 2Ha and 2Hb), 3.43 (p, *J* = 8.6 Hz, 1Ha and 1Hb), 2.31–1.75 (m, 6Ha and 6Hb). ^13^C NMR (151 MHz, DMSO-*d*_6_) δ 162.44, 160.80, 154.87, 147.52, 146.92, 141.25, 140.36, 133.24, 128.12, 126.86, 126.31, 122.93, 105.51, 96.07, 44.24, 28.99, 18.13.

##### *N*2-Benzyl-*N*4-(5-phenyl-1*H*-pyrazol-3-yl)­thieno­[3,2-*d*]­pyrimidine-2,4-diamine (**6**)

Compound **6** was synthesized following the general procedure C, starting from 68.8 mg (0.209 mmol) of **39** and benzylamine (0.114 mL, 1.046 mmol) dissolved in 0.7 mL of *n*-butanol. Purification by normal phase flash chromatography employing a 12 g gold silica cartridge (Solvent A: CH_2_Cl_2_ – Solvent B: CH_2_Cl_2_/EtOH 9:1 – Detection: 240/260 nm – Gradient: 10–30% of Solvent B) a final trituration by diethyl ether (7 mL), and solvent removal via lyophilization and high *vacuum*, yielded the desired product as a white solid (37 mg, 45%). UPLC-MS (generic method): Rt = 2.09 min, MS (ESI) *m*/*z*: 399.1 [M + H]^+^, C_22_H_19_N_6_S^+^ [M + H]^+^ calculated: 399.1. QC analysis: Rt = 4.31 min, UPLC-MS purity (UV at 215 nm): 99.5%. ^1^H spectrum in DMSO-*d*_6_ was consistent with the isolation of two different tautomeric forms, a and b, in a dynamic equilibrium, with a corresponding to the major and b to the minor in abundance. ^1^H NMR (400 MHz, DMSO-*d*_6_) δ 12.97 (br. s, 1Ha and 1Hb), 10.50 (br. s, 1Hb), 9.91 (br. s, 1Ha), 7.96 (br. s, 1Ha and 1Hb), 7.75 (br. s, 2Ha and 2Hb), 7.44–7.37 (m, 5Ha and 5Hb), 7.34–7.29 (m, 3Ha and 3Hb), 7.25 (m, 1Ha and 1Hb), 7.11 (br. s, 1Ha and 1Hb), 6.39 (br. s, 1Ha and 1Hb), 4.59 (d, *J* = 6.1 Hz, 2Ha and 2Hb). ^13^C NMR (151 MHz, DMSO-*d*_6_) δ 162.94, 162.57, 160.68, 160.43, 148.79, 141.34, 133.99, 133.49, 128.74, 128.24, 127.38, 126.54, 124.92, 123.52, 123.17, 105.61.

##### *N*2-Benzyl-*N*4-(5-(pyridin-3-yl)-1*H*-pyrazol-3-yl)­thieno­[3,2-*d*]­pyrimidine-2,4-diamine (**7**)

Compound **7** was synthesized following the general procedure D, starting from 62 mg (0.189 mmol) of **40**, benzylamine (0.031 mL, 0.282 mmol), and DIPEA (0.082 mL, 0.471 mmol) dissolved in 0.941 mL of *n*-butanol and stirred for 6 h. Purification by normal phase flash chromatography employing a 24 g alumina (Al_2_O_3_ pH = 7) cartridge (Solvent A: CH_2_Cl_2_ – Solvent B: CH_2_Cl_2_/EtOH 9:1 – Detection: 240/260 nm – Gradient: 0–60% of Solvent B) and a final trituration by cold diethyl ether (7 mL × 2) yielded, after freeze-drying, the desired product as a pale yellow solid (29.3 mg, 47%). UPLC-MS (generic method): Rt = 1.93 min, MS (ESI) *m*/*z*: 398.0 [M – H]^−^, C_21_H_16_N_7_S^–^ [M – H]^−^ calculated: 398.13. QC analysis: Rt = 3.54 min, UPLC-MS purity (UV at 215 nm): 99.5%. ^1^H NMR (400 MHz, DMSO-*d*_6_) δ 13.24 (br. s, 1H), 10.59 (br. s, 1H), 9.01 (br. s, 1H), 8.51 (dd, *J* = 4.7, 1.5 Hz, 1H), 8.12 (br. s, 1H), 7.98 (br. s, 1H), 7.70–7.12 (m, 8H), 6.58 (br. s, 1H), 4.59 (d, *J* = 6.0 Hz, 2H). ^13^C NMR (151 MHz, DMSO-*d*_6_) δ 162.58, 160.42, 153.41, 148.50, 146.27, 140.37, 133.59, 132.05, 129.60, 128.26, 127.32, 126.59, 123.81, 123.43, 105.61, 89.37, 44.36.

##### *N*4-(5-Cyclopropyl-1*H*-pyrazol-3-yl)-*N*2-(4-fluorobenzyl)­thieno­[3,2-*d*]­pyrimidine-2,4-diamine (**8**)

Compound **8** was synthesized following the general procedure C, starting from 80 mg (0.275 mmol) of **34** and 1.242 mmol of 4-fluorobenzylamine (0.157 mL, 1.37 mmol) dissolved in 0.92 mL of *n*-butanol. Purification by normal phase flash chromatography employing a 12 g gold silica cartridge (Solvent A: CH_2_Cl_2_ – Solvent B: CH_2_Cl_2_/EtOH 9:1 – Detection: 240/260 nm – Gradient: 10–40% Solvent B) and a final trituration with CH_2_Cl_2_ (7 mL) yielded, after solvent removal under *vacuum* at 80 °C, the desired product as a white solid (85 mg, 74%). UPLC-MS (generic method): Rt = 2.07 min, MS (ESI) *m*/*z*: 381.0 [M + H]^+^, C_19_H_18_FN_6_S^+^ [M + H]^+^ calculated: 381.1. QC analysis: Rt = 3.83 min, UPLC-MS purity (UV at 215 nm): 99.5%. ^1^H NMR (400 MHz, DMSO-*d*_6_) δ 12.14 (br. s, 1H), 9.70 (br. s, 1H), 7.90 (d, *J* = 5.4 Hz, 1H), 7.37 (dd, *J* = 8.3, 5.6 Hz, 2H), 7.11 (t, *J* = 8.9 Hz, 2H), 7.05 (d, *J* = 5.4 Hz, 1H), 6.12 (br. s, 1H), 4.51 (d, *J* = 6.2 Hz, 2H), 1.85-1.81 (m, 1H), 0.87 (dd, *J* = 8.4, 2.4 Hz, 2H), 0.62 (br. s, 2H). ^13^C NMR (151 MHz, DMSO-*d*_6_) δ 162.38, 161.21 (C-F *J* = 239.5 Hz), 160.19, 154.88, 152.38, 147.51, 145.21, 137.33, 136.53, 133.27, 129.22 (C-F *J* = 62.1 Hz), 123.46, 122.92, 114.88 (C-F *J* = 20.8 Hz), 105.58, 95.07, 43.55, 7.71.

##### *N*2-(4-Chlorobenzyl)-*N*4-(5-cyclopropyl-1*H*-pyrazol-3-yl)­thieno­[3,2-*d*]­pyrimidine-2,4-diamine (**9**)

Compound **9** was synthesized following the general procedure C, starting from 80 mg (0.275 mmol) of **34** and 4-chlorobenzylamine (0.167 mL, 1.37 mmol) dissolved in 0.92 mL of *n*-butanol. Purification by normal phase flash chromatography employing a 12 g gold silica cartridge (Solvent A: CH_2_Cl_2_ – Solvent B: CH_2_Cl_2_/EtOH 9:1 – Detection: 240/260 nm – Gradient: 0–40% of Solvent B) and a final trituration with CH_2_Cl_2_ (7 mL) and water (7 mL) to remove a basic impurity yielded, after freeze-drying, the desired product as a white solid (62.7 mg, 62%). UPLC-MS (generic method): Rt = 2.19 min, MS (ESI) *m*/*z*: 397.0/399.0 [M + H]^+^ and 395.0/397.0 [M – H]^−^ C_19_H_18_ClN_6_S^+^ [M + H]^+^ calculated: 397.1. QC analysis: Rt = 4.15 min, UPLC-MS purity (UV at 215 nm): 98%. ^1^H NMR (400 MHz, DMSO-*d*_6_) δ 12.07 (br. s, 1H), 9.69 (br. s, 1H), 7.91 (d, *J* = 4.4 Hz, 1H), 7.36 (br. s, 5H), 7.04 (d, *J* = 4.4 Hz, 1H), 6.13 (br. s, 1H), 4.52 (d, *J* = 6.2 Hz, 2H), 1.85-1.83 (m, 1H), 0.87 (d, *J* = 7.17 Hz, 2H), 0.62 (br. s, 2H). ^13^C NMR (151 MHz, DMSO-*d*_*6*_) δ 162.36, 160.63, 160.22, 154.90, 152.42, 147.50, 145.20, 140.33, 133.24, 129.11, 128.71, 128.65, 128.62, 128.05, 122.89, 105.63, 95.08, 43.59, 7.71, 6.84.

##### *N*4-(5-Cyclopropyl-1*H-*pyrazol-3-yl)-*N*2-(4-methoxybenzyl)­thieno­[3,2-*d*]­pyrimidine-2,4-diamine (**10**)

Compound **10** was synthesized following the general procedure C, starting from 80 mg (0.275 mmol) of **34** and 4-methoxybenzylamine (0.179 mL, 1.37 mmol) dissolved in 0.92 mL of *n*-butanol. Purification by normal phase flash chromatography employing a 12 g gold silica cartridge (Solvent A: CH_2_Cl_2_ – Solvent B: CH_2_Cl_2_/EtOH 9:1 – Detection: 240/260 nm – Gradient: 10–30% Solvent B) and a final trituration by CH_2_Cl_2_ (7 mL) yielded the desired product as a white solid (48 mg, 45%). UPLC-MS (generic method): Rt = 1.99 min, MS (ESI) *m*/*z*: 393.1 [M + H]^+^, C_20_H_21_N_6_OS^+^ [M + H]^+^ calculated: 393.1. QC analysis: Rt = 3.57 min, UPLC-MS purity (UV at 215 nm): 99%. ^1^H NMR (400 MHz, DMSO-*d*_6_) δ 12.07 (br. s, 1H), 9.60 (br. s, 1H), 7.91 (br. s, 1H), 7.27 (d, *J* = 8.22 Hz, 2H), 7.05 (br. s, 1H), 6.87 (d, *J* = 8.22 Hz, 2H), 6.32 (br. s, 1H), 4.46 (d, *J* = 6.1 Hz, 2H), 3.71 (s, 3H), 1.84 (tt, *J* = 8.7, 5.1 Hz, 1H), 0.87 (d, *J* = 5.5 Hz, 2H), 0.64 (br. s, 2H). ^13^C NMR (151 MHz, DMSO-*d*_6_) δ 162.41, 160.69, 158.00, 157.93, 154.81, 147.58, 145.20, 133.13, 128.65, 128.18, 128.09, 123.47, 122.93, 113.57, 105.41, 95.05, 55.02, 43.72, 7.72.

##### 4-(((4-((5-Cyclopropyl-1*H*-pyrazol-3-yl)­amino)­thieno­[3,2-*d*]­pyrimidin-2-yl)­amino)­methyl)­benzonitrile (**11**)

Compound **11** was synthesized following the general procedure D, starting from 110 mg (0.377 mmol) of **34**, 4-cyanobenzylamine (74.75 mg, 0.566 mmol) and DIPEA (0.164 mL, 0.942 mmol) dissolved in 1.5 mL of *n*-butanol and stirred for 6 h. After completion, the reaction mixture was concentrated in vacuo, dissolved in H_2_O and the pH was adjusted to 7. The aqueous phase was extracted with EtOAc (10 mL × 3), the organic layers were collected and dried over Na_2_SO_4_ and concentrated under *vacuum*. The resulting crude product was purified by normal phase flash chromatography employing a 12 g gold silica cartridge (Solvent A: CH_2_Cl_2_ – Solvent B: CH_2_Cl_2_/1N NH_3_ in MeOH 9:1 – Detection: 240/260 nm – Gradient: 10–20% of Solvent B). The isolated fraction was concentrated under high *vacuum* and lyophilized to yield the desired product as a white solid (42.5 mg, 29%). UPLC-MS (generic method): Rt = 1.90 min, MS (ESI) *m*/*z*: 388.1 [M + H]^+^, C_20_H_18_N_7_S^+^ [M + H]^+^ calculated: 388.1. QC analysis: Rt = 3.42 min, UPLC-MS purity (UV at 215 nm): 98%. Both ^1^H and ^13^C NMR spectra in DMSO-*d*_6_ were consistent with the isolation of two different tautomeric forms a and b in a dynamic equilibrium, with a corresponding to the major and b to the minor in abundance. ^1^H NMR (400 MHz, DMSO-*d*_6_) δ 12.55 (br. s, 1Hb), 12.08 (br. s, 1Ha), 10.28 (br. s, 1Hb), 9.64 (br. s, 1Ha), 7.90 (br. s, 1Ha and 1Hb), 7.77 (d, *J* = 8.0 Hz, 2Ha and 2Hb), 7.52 (d, *J* = 8.0 Hz, 2Ha and 2Hb), 7.26 (br. s, 1Ha and 1Hb), 7.03 (br. s, 1Ha and 1Hb), 6.27 (br. s, 1Hb), 5.68 (br. s, 1Ha), 4.62 (d, *J* = 6.3 Hz, 2Ha and 2Hb), 1.83 (br. s, 1Ha and 1Hb), 0.88 (br. s, 2Ha and 2Hb), 0.62 (br. s, 2Ha and 2Hb). ^13^C NMR (151 MHz, DMSO-*d*_6_) δ 162.31, 160.57, 154.96, 153.26, 152.47, 147.47, 146.69, 145.20, 133.35, 133.21, 132.14, 127.99, 127.97, 127.93, 127.72, 122.89, 119.04, 109.03, 105.82, 95.14, 44.08, 44.05, 9.37, 7.70, 6.83.

##### 4-(((4-((5-Cyclopropyl-1*H*-pyrazol-3-yl)­amino)­thieno­[3,2-*d*]­pyrimidin-2-yl)­amino)­methyl)­phenol (12)

In a 25 mL round flask with a stir bar, intermediate **45** (220 mg, 0.357 mmol) and KHF_2_ (69.70 mg, 0.892 mmol) were stirred in anhydrous MeOH (3.57 mL) at room temperature overnight. TLC analysis in CH_2_Cl_2_/MeOH 9.5/0.5 and UPLC-MS analysis revealed the presence of the desired deprotected product **12**. Evaporation of the reaction solvent in vacuo gave the corresponding crude product, which was directly purified by normal-flash column chromatography by employing a 12 g gold silica cartridge (Solvent A: CHCl_3_ – Solvent B: CH_2_Cl_2_/MeOH 9:1 – Detection: 240/260 nm – Gradient: 20–100% of Solvent B), a sequential change of the elution phase during the course of the separation into 8:2 CH_2_Cl_2_/MeOH, yielded, after trituration in CH_2_Cl_2_ (10 mL) and 3 h under direct high *vacuum* at 80 °C, the desired product as a white solid (80 mg, 60%). UPLC-MS (generic method): Rt = 1.58 min, MS (ESI) *m*/*z*: 379.0 [M + H]^+^, C_19_H_19_N_6_OS^+^ [M + H]^+^ calculated: 379.1. QC analysis: Rt = 2.81 min, UPLC-MS purity (UV at 215 nm): 96%. Both ^1^H and ^13^C NMR spectra in DMSO-*d*_6_ were consistent with the isolation of two different tautomeric forms, a and b, in a dynamic equilibrium, with a corresponding to the major and b to the minor in abundance. ^1^H NMR (600 MHz, DMSO-*d*_6_) δ 12.56 (br. s, 1Hb), 12.08 (br. s, 1Ha), 10.23 (br. s, 1Hb), 9.60 (br. s, 1Ha), 9.24 (s, 1Ha and 1Hb), 7.90 (s, 2Ha and 2Hb), 7.53 (br. s, 1Hb), 7.15 (d, *J* = 7.3 Hz, 1Ha and 1Hb), 7.04 (br. s, 2Ha and 2Hb), 6.69 (d, *J* = 7.3 Hz, 1Ha and 1Hb), 6.34 (br. s, 1Hb), 5.59 (br. s, 1Ha), 4.41 (d, *J* = 6.1 Hz, 2Ha and 2Hb), 1.85-1.82 (m, 1Ha and 1Hb), 0.87 (br. s, 2Ha and 2Hb), 0.64 (br. s, 2Ha and 2Hb). ^13^C NMR (151 MHz, DMSO-*d*_6_) δ 162.49, 160.61, 156.06, 134.54, 133.28, 128.65, 128.47, 123.18, 114.99, 105.40, 43.92, 7.82.

##### 4-(((4-((5-Cyclopropyl-1*H*-pyrazol-3-yl)­amino)­thieno­[3,2-*d*]­pyrimidin-2-yl)­amino)­methyl)­benzamide (**13**)

Compound **13** was synthesized adapting the general procedure D starting from 108.24 mg (0.371 mmol) of **34**, 4-(aminomethyl)­benzamide, trifluoroacetic salt **47** (147 mg, 0.556 mmol) and DIPEA (0.151 mL, 0.866 mmol 3.5 equiv) dissolved in 1.85 mLof *n*-butanol and stirred for 8 h. After completion, the reaction mixture was cooled down to room temperature and filtrated under *vacuum*. A final trituration with EtOH (5 mL × 2) of the obtained solid yielded the pure product as a pale-yellow solid (98.3 mg, 65%). UPLC-MS (generic method): Rt = 1.43 min, MS (ESI) *m*/*z*: 406.0 [M + H]^+^, C_20_H_20_N_7_OS^+^ [M + H]^+^ calculated: 406.1. QC analysis: Rt = 2.45 min, UPLC-MS purity (UV at 215 nm): 96%. Both ^1^H and ^13^C NMR spectra in DMSO-*d*_6_ were consistent with the isolation of two different tautomeric forms a and b in a dynamic equilibrium, with a corresponding to the major and b to the minor in abundance. ^1^H NMR (400 MHz, DMSO-*d*_6_) δ 12.57 (br. s, 1Hb), 12.06 (br. s, 1Ha), 10.26 (br. s, 1Hb), 9.61 (br. s, 1Ha), 7.88 (br. s, 2Ha and 2Hb), 7.81 (d, *J* = 7.3 Hz, 2Ha and 2Hb), 7.40–7.03 (m, 5Ha and 5Hb), 6.31 (br. s, 1Ha), 5.66 (br. s, 1Hb), 4.59 (d, *J* = 6.0 Hz, 2Ha and 2Hb), 1.86–1.80 (m, 1Ha and 1Hb), 0.92–0.82 (m, 2Ha and 2Hb), 0.63 (br. s, 2Ha and 2Hb). ^13^C NMR (151 MHz, DMSO-*d*_6_) δ 167.77, 162.39, 160.65, 154.86, 147.52, 145.22, 144.68, 143.08, 133.32, 132.45, 132.41, 127.43, 126.81, 126.56, 122.95, 105.59, 95.06, 60.36, 44.04, 13.86, 13.60, 7.81, 7.72, 6.86.

##### *N*2-(Benzo­[*d*]­[1,3]­dioxol-5-ylmethyl)-*N*4-(5-cyclopropyl-1*H*-pyrazol-3-yl)­thieno­[3,2-d]­pyrimidine-2,4-diamine (**14**)

Compound **14** was synthesized following the general procedure D, starting from 150 mg (0.369 mmol) of **34**, 1,3-benzodioxole-5-methylamine (0.096 mL, 0.771 mmol) and DIPEA (0.223 mL, 1.284 mmol) dissolved in 2.6 mL of *n*-butanol and stirred for 6 h. After completion, the reaction mixture was concentrated in vacuo, and the resulting crude product was purified by normal phase flash chromatography employing a 12 g gold silica cartridge (Solvent A: CH_2_Cl_2_ – Solvent B: CH_2_Cl_2_/1N NH_3_ in MeOH 9:1 – Detection: 240/260 nm – Gradient: 10–30% of Solvent B). The isolated fraction was precipitated in CH_2_Cl_2_ (10 mL) and filtered under *vacuum* to yield the desired product as a white solid (83.6 mg, 56%). UPLC-MS (generic method): Rt = 2.00 min, MS (ESI) *m*/*z*: 407.0 [M + H]^+^, C_20_H_19_N_6_O_2_S^+^ [M + H]^+^ calculated: 407.1. QC analysis: Rt = 3.52 min, UPLC-MS purity (UV at 215 nm): 97%. ^1^H spectrum in DMSO-*d*_6_ was consistent with the isolation of two different tautomeric forms, a and b, in a dynamic equilibrium, with a corresponding to the major and b to the minor in abundance. ^1^H NMR (400 MHz, DMSO-*d*_6_) δ 12.54 (br. s, 1Hb), 12.08 (br. s, 1Ha), 10.24 (br. s, 1Hb), 9.59 (br. s, 1Ha), 7.90 (br. s, 1Ha), 7.59 (br. s, 1Hb), 7.04–6.65 (m, 5Ha and 5Hb), 6.35 (br. s, 1Ha), 5.95 (s, 2Ha and 2Hb), 5.67 (br. s, 1Hb), 4.44 (d, *J* = 6.2 Hz, 2Ha and 2Hb), 1.86–1.82 (m, 1Ha and 1Hb), 0.88 (br. s, 2Ha and 2Hb), 0.64 (br. s, 2Ha and 2Hb). ^13^C NMR (151 MHz, DMSO-*d*_6_) δ 162.46, 160.71, 160.26, 154.88, 153.21, 152.43, 147.60, 147.15, 145.73, 145.27, 140.30, 135.21, 134.21, 133.25, 122.94, 120.51, 119.96, 108.00, 107.95, 107.67, 107.61, 100.69, 95.09, 9.39, 7.76, 7.72, 6.88, 6.83.

##### 2-(Benzyloxy)-*N*-(5-cyclopropyl-1*H*-pyrazol-3-yl)­thieno­[3,2-*d*]­pyrimidin-4-amine (**15**)

Compound **15** was synthesized starting from intermediate **34** (125 mg, 0.428 mmol), according to the general procedure E. The resulting crude product was purified by normal phase flash chromatography employing a 12 g gold silica cartridge (Solvent A: CH_2_Cl_2_ – Solvent B: CH_2_Cl_2_/1N NH_3_ in MeOH 9:1 – Detection: 240/260 nm – Gradient: 10–20% Solvent B) to yield the desired product as a white solid (79.1 mg, 51%). UPLC-MS (generic method): Rt = 2.14 min, MS (ESI) *m*/*z*: 364.0 [M + H]^+^ C_19_H_18_N_5_OS^+^ [M + H]^+^ calculated: 364.1. QC analysis: Rt = 4.12 min, UPLC-MS purity (UV at 215 nm): 99%. ^1^H NMR (400 MHz, DMSO-*d*_6_) δ 12.24 (br. s, 1H), 10.04 (br. s, 1H), 8.07 (d, *J* = 5.4 Hz, 1H), 7.46–7.29 (m, 5H), 7.23 (d, *J* = 5.4 Hz, 1H), 6.26 (br. s, 1H), 5.39 (s, 2H), 1.99–1.87 (m, 1H), 0.92 (d, *J* = 7.2 Hz, 2H), 0.79–0.51 (m, 2H). ^13^C NMR (151 MHz, DMSO-*d*_6_) δ 162.95, 162.08, 156.52, 146.63, 145.70, 137.43, 135.06, 128.35, 127.71, 127.66, 123.13, 109.48, 96.06, 67.65, 7.76, 6.84.

##### 3-(((4-((5-Cyclopropyl-1*H*-pyrazol-3-yl)­amino)­thieno­[3,2-*d*]­pyrimidin-2-yl)­amino) methyl)­benzamide (**16**)

Compound **16** was synthesized following the general procedure D, starting from 80 mg (0.274 mmol) of intermediate **34**, 3-(aminomethyl)­benzamide (61.72 mg, 0.411 mmol), and DIPEA (0.119 mL, 0.685 mmol) dissolved in 1.37 mL of *n*-butanol and stirred for 8 h. After completion, the reaction mixture was concentrated in vacuo and the resulting crude product was purified by normal phase flash chromatography employing a 12 g gold silica cartridge (Solvent A: CH_2_Cl_2_ – Solvent B: CH_2_Cl_2_/MeOH 9:1 – Detection: 240/260 nm – Gradient: 10–60% of Solvent B) followed by a trituration in CH_2_Cl_2_ (3 mL × 2) to give 38 mg of the desired product with impurities. A sequential purification employing a 4 g gold silica cartridge (Solvent A: CH_2_Cl_2_ – Solvent B: CH_2_Cl_2_/1N NH_3_ in MeOH 9:1 – Detection: 240/260 nm – Gradient: 0–50% of Solvent B) yielded the desired product as a white solid (27 mg, 24%). UPLC-MS (generic method): Rt = 1.46 min, MS (ESI) *m*/*z*: 406.2 [M + H]^+^, C_20_H_20_N_7_OS^+^ [M + H]^+^ calculated: 406.1. QC analysis: Rt = 2.56 min, UPLC-MS purity (UV at 215 nm): 99%. Both ^1^H and ^13^C NMR spectra in DMSO-*d*_6_ were consistent with the isolation of two different tautomeric forms, a and b, in a dynamic equilibrium, with a corresponding to the major and b to the minor in abundance. ^1^H NMR (400 MHz, DMSO-*d*_6_) δ 12.58 (br. s, 1Hb), 12.06 (br. s, 1Ha), 10.30 (br. s, 1Hb), 9.62 (br. s, 1Ha), 7.93 (s, 1Ha and 1Hb), 7.88 (s, 1Ha and 1Hb), 7.72 (d, *J* = 7.7 Hz, 1Ha and 1Hb), 7.49 (d, *J* = 7.0 Hz, 1Ha and 1Hb), 7.37 (t, *J* = 7.4 Hz, 1Ha and 1Hb), 7.31 (br. s, 1Ha and 1Hb), 7.17 (br. s, 1Hb), 7.04 (br. s, 1Ha), 6.32 (br. s, 1Hb), 5.67 (br. s, 1Ha), 4.59 (d, *J* = 6.2 Hz, 2Ha and 2Hb), 1.86–1.80 (m, 1Ha and 1Hb), 0.87 (br. s, 2Ha and 2Hb), 0.63 (br. s, 2Ha and 2Hb). ^13^C NMR (151 MHz, DMSO-*d*_6_) δ 168.20, 162.45, 160.63, 141.17, 134.28, 133.44, 130.01, 128.17, 126.46, 125.60, 123.21, 105.72, 44.26, 7.85.

##### *N*4-(5-Cyclopropyl-1*H*-pyrazol-3-yl)-*N*2-(pyridin-3-ylmethyl)­thieno­[3,2-*d*]­pyrimidine-2,4-diamine (**17**)

Compound **17** was synthesized following the general procedure D, starting from 125 mg (0.539 mmol) of intermediate **34**, 3-picolylamine (0.082 mL, 0.809 mmol), and DIPEA (0.235 mL, 1.348 mmol) dissolved in 2.7 mL of *n*-butanol and stirred for 8 h. After completion, the reaction mixture was concentrated in vacuo, and the resulting crude product was purified by normal phase flash chromatography employing a 48 g alumina (Al_2_O_3_ pH = 7) cartridge (Solvent A: CHCl_3_ – Solvent B: CHCl_3_/EtOH 9:1 – Detection: 240/260 nm – Gradient: 5–50% of Solvent B). A second purification via normal phase chromatography employing a 4 g gold silica cartridge (Solvent A: CH_2_Cl_2_ – Solvent B: CH_2_Cl_2_/MeOH 9:1 in NH_3_ 1 N – detection: 240/260 nm gradient: 10–40% of Solvent B) yielded the pure product which fraction was concentrated under high *vacuum* and lyophilized to yield the desired product as a white solid (17 mg, 9%). UPLC-MS (generic method): Rt = 1.49 min, MS (ESI) *m*/*z*: 364.0 [M + H]^+^, C_18_H_18_N_7_S ^+^ [M + H]^+^ calculated: 364.1. QC analysis: Rt = 2.63 min, UPLC-MS purity (UV at 215 nm): 98%. Both ^1^H and ^13^C NMR spectra in DMSO-*d*_6_ were consistent with the isolation of two different tautomeric forms, a and b, in a dynamic equilibrium, with a corresponding to the major and b to the minor in abundance. ^1^H NMR (400 MHz, DMSO-*d*_6_) δ 12.48 (br. s, 1Hb), 12.11 (br. s, 1Ha), 10.20 (br. s, 1Hb), 9.69 (br. s, 1Ha), 8.57 (br. s, 1Ha and 1Hb), 8.42 (d, *J* = 4.4 Hz, 1Ha and 1Hb), 7.92 (br. s, 1Ha and 1Hb), 7.74 (d, *J* = 7.6 Hz, 1Ha and 1Hb), 7.32 (dd, *J* = 7.6, 5.2 Hz, 1Ha and 1Hb), 7.25 (br. s, 1Ha and 1Hb), 7.06 (br. s, 1Ha and 1Hb), 6.27 (br. s, 1Ha and 1Hb), 4.55 (d, *J* = 6.2 Hz, 2Ha and 2Hb), 1.86–1.81 (m, 1Ha and 1Hb), 1.03–0.77 (d, *J* = 7.12 Hz, 2Ha and 2Hb), 0.64 (br. s, 2Ha and 2Hb). ^13^C NMR (151 MHz, DMSO-*d*_6_) δ 162.34, 160.53, 155.00, 148.79, 147.75, 145.33, 136.49, 134.94, 133.42, 123.40, 122.97, 105.75, 95.20, 42.03, 7.75, 6.88.

##### *N*4-(5-Cyclopropyl-1*H*-pyrazol-3-yl)-*N*2-(3-methoxybenzyl)­thieno­[3,2-*d*]­pyrimidine-2,4-diamine (**18**)

Compound **18** was synthesized following the general procedure D starting from 80 mg (0.274 mmol) of intermediate **34**, 3-methoxybenzylamine (0.052 mL, 0.411 mmol) and DIPEA (0.119 mL, 0.685 mmol) dissolved in 1.37 mL of *n*-butanol and stirred for 8 h. After completion, the reaction mixture was concentrated in vacuo and the resulting crude product was purified by normal phase flash chromatography employing a 12 g gold silica cartridge (Solvent A: CHCl_3_ – Solvent B: CHCl_3_/MeOH 9:1 – Detection: 240/260 nm – Gradient: 5–20% of Solvent B). A sequential trituration in cold CH_2_Cl_2_ (3 mL × 2) yielded the desired product as a white solid (50 mg, 47%). UPLC-MS (generic method): Rt = 1.90 min, MS (ESI) *m*/*z*: 393.1 [M + H]^+^, C_20_H_21_N_6_OS^+^ [M + H]^+^ calculated: 393.1. QC analysis: Rt = 3.66 min, UPLC-MS purity (UV at 215 nm): 99%. ^1^H NMR (400 MHz, DMSO-*d*_6_) δ 12.21 (br. s, 1H), 9.74 (br. s, 1H), 7.90 (d, *J* = 5.3 Hz, 1H), 7.21 (t, *J* = 8.0 Hz, 1H), 7.05 (d, *J* = 5.4 Hz, 1H), 6.92 (s, 1H), 6.91 (s, 1H), 6.77 (dd, *J* = 8.2, 2.5 Hz, 1H), 6.12 (br. s, 1H), 4.52 (d, *J* = 6.2 Hz, 2H), 3.70 (s, 3H), 1.88–1.79 (m, 1H), 0.92–0.84 (m, 2H), 0.63 (br. s, 2H). ^13^C NMR (151 MHz, DMSO-*d*_6_) δ 162.42, 160.68, 159.32, 154.42, 142.64, 133.34, 129.30, 123.16, 119.34, 112.87, 111.79, 105.64, 54.97, 44.32, 7.82.

##### *N*2-Benzyl-*N*4-(5-cyclopropyl-1*H*-pyrazol-3-yl)-6,7-dihydrothieno­[3,2-*d*]­pyrimidine-2,4-diamine (**19**)

Compound **19** was synthesized following the general procedure D, starting from 125 mg (0.425 mmol) of **43**, benzylamine (0.069 mL, 0.637 mmol), and DIPEA (0.184 mL, 1.062 mmol) dissolved in 2.12 mL of *n*-butanol and stirred for 6 h. Purification by normal phase flash chromatography employing a 12 g gold silica cartridge (Solvent A: CH_2_Cl_2_ – Solvent B: CH_2_Cl_2_/EtOH 9:1 – Detection: 240/260 nm – Gradient: 0–40% of Solvent B) and a final trituration by diethyl ether (7 mL) yielded the desired product as a white solid (77.1 mg, 50%). UPLC-MS (generic method): Rt = 1.87 min, MS (ESI) *m*/*z*: 365.0 [M + H]^+^ C_19_H_21_N_6_S^+^ [M + H]^+^ calculated: 365.1. QC analysis: Rt = 3.85 min, UPLC-MS purity (UV at 215 nm): 99%. ^1^H spectrum in DMSO-*d*_6_ was consistent with the isolation of two different tautomeric forms, a and b, in a dynamic equilibrium, with a corresponding to the major and b to the minor in abundance. ^1^H NMR (400 MHz, DMSO-*d*_6_) δ 12.32 (br. s, 1Hb), 11.94 (br. s, 1Ha), 9.27 (br. s, 1Hb), 8.31 (br. s, 1Ha), 7.30 (d, *J* = 4.20 Hz, 4Ha and 4Hb), 7.20 (m, 1Hb and 1Ha), 5.88 (br. s, 1Hb and 1Ha), 4.47 (d, *J* = 6.4 Hz, 2Ha and 2Hb), 3.22 (t, *J* = 7.3 Hz, 2Ha and 2Hb), 3.00 (t, *J* = 7.3 Hz, 2Ha and 2Hb), 1.78 (m, 1Ha and 1Hb), 0.83 (d, *J* = 5.7 Hz, 2Ha and 2Hb), 0.56 (br. s, 2Ha and 2Hb). ^13^C NMR (151 MHz, DMSO-*d*_6_) δ 168.90, 160.67, 153.81, 147.66, 145.16, 140.73, 128.14, 126.93, 126.41, 126.11, 101.50, 94.12, 44.29, 36.85, 29.13, 7.57.

##### *N*2-Benzyl-*N*4-(5-cyclopropyl-1*H*-pyrazol-3-yl)­furo­[3,2-*d*]­pyrimidine-2,4-diamine (**20**)

Compound **20** was synthesized following the general procedure D, starting from 70 mg (0.254 mmol) of intermediate **41**, benzylamine (0.042 mL, 0.380 mmol), and DIPEA (0.111 mL, 0.635 mmol) dissolved in *n*-butanol (1.3 mL) and stirred for 8 h. After completion, the reaction mixture was concentrated in vacuo and the resulting crude product was purified by normal phase flash chromatography employing 24 g alumina (Al_2_O_3_ pH = 7) cartridge (Solvent A: CH_2_Cl_2_ – Solvent B: CH_2_Cl_2_/MeOH 9:1 – Detection: 240/260 nm – Gradient: 0–30% of Solvent B) followed by a final trituration in cold CH_2_Cl_2_ (5 mL), furnished the pure product, which fraction was concentrated under high *vacuum* and lyophilized to yield 31.1 mg (35%) of a white solid. UPLC-MS (generic method): Rt = 1.75 min, MS (ESI) *m*/*z*: 347.0 [M + H]^+^, C_19_H_19_N_6_O^+^ [M + H]^+^ calculated: 347.1. QC analysis: Rt = 3.47 min, UPLC-MS purity (UV at 215 nm): 99%. Both ^1^H and ^13^C NMR spectra in DMSO-*d*_6_ were consistent with the isolation of two different tautomeric forms a and b in a dynamic equilibrium, with a corresponding to the major and b to the minor in abundance. ^1^H NMR (400 MHz, DMSO-*d*_6_) δ 12.45 (br. s, 1Hb), 11.98 (br. s, 1Ha), 10.48 (br. s, 1Hb), 9.65 (br. s, 1Ha), 8.01 (br. s, 1Ha and 1Hb), 7.35–7.19 (m, 5Ha and 5Hb), 7.07 (br. s, 1Ha and 1Hb), 6.68 (br. s, 1Ha and 1Hb), 6.35 (br. s, 1Ha), 5.68 (br. s, 1Hb), 4.51 (d, *J* = 6.0 Hz, 2Ha and 2Hb), 1.85–1.79 (m, 1Ha and 1Hb), 0.86 (d, *J* = 6.3 Hz, 2Ha and 2Hb), 0.62 (br. s, 2Ha and 2Hb). ^13^C NMR (101 MHz, DMSO-*d*_6_) δ 159.41, 151.74, 149.39, 144.78, 140.99, 128.76, 128.19, 127.07, 126.44, 106.87, 44.62, 7.73.

##### 2-(Benzyloxy)-*N*-(5-cyclobutyl-1*H*-pyrazol-3-yl)­thieno­[3,2-*d*]­pyrimidin-4-amine (**21**)

Compound **21** was synthesized starting from intermediate **38** (80 mg, 0.262 mmol) and sodium benzyloxide (1 M sol., 0.288 mL) according to the general procedure E. The resulting crude product was purified by normal phase flash chromatography employing a 12 g gold silica cartridge (Solvent A: CH_2_Cl_2_ – Solvent B: CH_2_Cl_2_/1N NH_3_ in MeOH 9:1 – Detection: 240/260 nm – Gradient: 10–50% of Solvent B) to yield the desired product as a white solid (44.3 mg, 45%). UPLC-MS (generic method): Rt = 2.09 min, MS (ESI) *m*/*z*: 378.0 [M + H]^+^ C_20_H_20_N_5_OS^+^ [M + H]^+^ calculated: 378.1. QC analysis: Rt = 4.53 min, UPLC-MS purity (UV at 215 nm): 98%. ^1^H NMR (400 MHz, DMSO-*d*_6_) δ 12.29 (s, 1H), 10.08 (s, 1H), 8.07 (d, *J* = 5.4 Hz, 1H), 7.45 (d, *J* = 7.5 Hz, 2H), 7.37 (t, *J* = 7.1 Hz, 2H), 7.35–7.27 (m, 1H), 7.24 (d, *J* = 5.4 Hz, 1H), 6.40 (s, 1H), 5.41 (s, 2H), 3.54–3.43 (m, 1H), 2.28 (qt, *J* = 8.3, 2.9 Hz, 2H), 2.12 (pd, *J* = 9.1, 2.6 Hz, 2H), 2.00–1.78 (m, 2H). ^13^C NMR (151 MHz, DMSO-*d*_*6*_) δ 163.06, 162.14, 156.57, 137.51, 135.12, 128.44, 127.76, 123.25, 67.75, 29.06, 18.19.

##### 2-(Benzyloxy)-*N*-(5-isopropyl-1*H*-pyrazol-3-yl)­thieno­[3,2-*d*]­pyrimidin-4-amine (**22**)

Compound **22** was synthesized starting from intermediate **37** (80 mg, 0.272 mmol) and sodium benzyloxide 1 M solution (0.408 mL) according to the general procedure E. The resulting crude product was purified by normal phase flash chromatography employing a 12 g gold silica cartridge (Solvent A: CH_2_Cl_2_ – Solvent B: CH_2_Cl_2_/1N NH_3_ in MeOH 9.9:0.1 – Detection: 240/260 nm – Gradient: 60–100% of Solvent B). A sequential trituration in CH_2_Cl_2_ (5 mL × 2) yielded the desired product as a white solid (43 mg, 43%). UPLC-MS (generic method): Rt = 2.18 min, MS (ESI) *m*/*z*: 366.1 [M + H]^+^ C_19_H_20_N_5_OS^+^ [M + H]^+^ calculated: 366.1. QC analysis: Rt = 4.34 min, UPLC-MS purity (UV at 215 nm): 99.5%. ^1^H NMR (400 MHz, DMSO-*d*_6_) δ 12.26 (br. s, 1H), 10.07 (br. s, 1H), 8.07 (d, *J* = 5.4 Hz, 1H), 7.45 (d, *J* = 6.9 Hz, 2H), 7.37 (t, *J* = 7.4 Hz, 2H), 7.32 (d, *J* = 7.2 Hz, 1H), 7.23 (d, *J* = 5.4 Hz, 1H), 6.36 (s, 1H), 5.41 (s, 2H), 2.97–2.92 (m, 1H), 1.22 (d, *J* = 7.0 Hz, 6H). ^13^C NMR (151 MHz, DMSO-*d*_6_) δ 163.01, 162.10, 156.56, 149.67, 146.41, 137.47, 135.06, 128.39, 127.73, 127.69, 123.19, 109.53, 96.15, 67.68, 25.52, 22.35.

##### 2-(Benzyloxy)-*N*-(5-cyclopropyl-1*H*-pyrazol-3-yl)­furo­[3,2-*d*]­pyrimidin-4-amine (**23**)

Compound **23** was synthesized starting from intermediate **41** (80 mg, 0.290 mmol) and sodium benzyloxide 1 M solution (0.319 mL) according to the general procedure E. The resulting crude product was purified by normal phase flash chromatography employing a 12 g gold silica cartridge (Solvent A: CH_2_Cl_2_ – Solvent B: CH_2_Cl_2_/1N NH_3_ in MeOH 9:1 – Detection: 240/260 nm – Gradient: 5–20% of Solvent B) to give 27 mg of the desired product with impurities. A sequential purification employing an 8 g alumina (Al_2_O_3_ pH = 7) cartridge (Solvent A: CH_2_Cl_2_ – Solvent B: CH_2_Cl_2_/MeOH 99:1 – detection: 240/260 nm – Gradient: 10–100% of Solvent B) yielded the desired product as a white solid (15 mg, 15%). UPLC-MS (generic method): Rt = 1.99 min, MS (ESI) *m*/*z*: 347.9 [M + H]^+^ C_19_H_18_N_5_O_2_^+^ [M + H]^+^ calculated: 348.1. QC analysis: Rt = 3.85 min, UPLC-MS purity (UV at 215 nm): 96%. ^1^H NMR (400 MHz, DMSO-*d*_6_) δ 12.12 (br. s, 1H), 10.13 (br. s, 1H), 8.18 (d, *J* = 1.5 Hz, 1H), 7.44–7.29 (m, 5H), 6.87 (d, *J* = 2.0 Hz, 1H), 6.28 (br. s, 1H), 5.35 (s, 2H), 1.91–1.87 (m, 1H), 0.91 (qd, *J* = 4.1, 2.0 Hz, 2H), 0.66 (qd, *J* = 4.1, 2.0 Hz, 2H). ^13^C NMR (151 MHz, DMSO-*d*_6_) δ 160.96, 151.73, 150.66, 146.32, 137.49, 130.68, 128.41, 127.73, 127.71, 107.22, 94.51, 67.99, 7.77.

##### 3-(((4-((5-Cyclobutyl-1*H*-pyrazol-3-yl)­amino)­thieno­[3,2-*d*]­pyrimidin-2-yl)­amino) methyl)­benzamide (**24**)

Compound **24** was synthesized following the general procedure D starting from 100 mg (0.327 mmol) of intermediate **38**, 3-(aminomethyl)­benzamide (73.6 mg, 0.490 mmol) and DIPEA (0.142 mL, 0.817 mmol) dissolved in 1.63 mL of *n*-butanol and stirred for 8 h. After completion, the reaction mixture was concentrated in vacuo and the resulting crude product was purified by normal phase flash chromatography employing a 12 g gold silica cartridge (Solvent A: CH_2_Cl_2_ – Solvent B: CH_2_Cl_2_/MeOH 9:1 – Detection: 240/260 nm – Gradient: 10–60% of Solvent B). A sequential purification using a 4 gold silica cartridge (Solvent A: CH_2_Cl_2_ – Solvent B: CH_2_Cl_2_/1N NH_3_ in MeOH 9:1 – Detection: 240/260 nm – Gradient: 10–40% of Solvent B) followed by trituration with cold CH_2_Cl_2_ and subsequently in water (3 mL), yielded the desired product as a white solid (25 mg, 18%). UPLC-MS (generic method): Rt = 1.61 min, MS (ESI) *m*/*z*: 420.0 [M + H]^+^, C_21_H_22_N_7_OS^+^ [M + H]^+^ calculated: 420.1. QC analysis: Rt = 2.86 min, UPLC-MS purity (UV at 215 nm): 99.5%. ^1^H NMR (400 MHz, DMSO-*d*_6_) δ 12.09 (br. s, 1H), 9.69 (br. s, 1H), 7.91 (d, *J* = 6.55 Hz, 1H), 7.89 (s, 1H), 7.72 (d, *J* = 8.0 Hz, 1H), 7.50 (d, *J* = 7.28 Hz, 1H), 7.38 (t, *J* = 7.5 Hz, 1H), 7.31 (br. s, 1H), 7.05 (br. s, 1H), 6.34 (br. s, 1H), 4.60 (d, *J* = 5.7 Hz, 2H), 2.24–1.81 (m, 6H). ^13^C NMR (151 MHz, DMSO-*d*_6_) δ 167.97, 162.41, 160.71, 141.39, 134.20, 133.32, 129.70, 127.97, 126.26, 125.38, 122.95, 105.60, 44.18, 31.18, 28.99.

##### *N*4-(5-Cyclobutyl-1*H*-pyrazol-3-yl)-*N*2-(pyridin-3-ylmethyl)­thieno­[3,2-*d*]­pyrimidine-2,4-diamine (**25**)

Compound **25** was synthesized following the general procedure D starting from 100 mg (0.327 mmol) of intermediate **38**, 3-picolylamine (0.050 mL, 0.490 mmol) and DIPEA (0.142 mL, 0.817 mmol) dissolved in 1.6 mL of *n*-butanol and stirred for 8 h. After completion, the reaction mixture was concentrated in vacuo and the resulting crude product was purified by normal phase flash chromatography employing a 4 g gold silica cartridge (Solvent A: CH_2_Cl_2_ – Solvent B: CH_2_Cl_2_/MeOH 8:2 – Detection: 240/260 nm – Gradient: 10–20% of Solvent B) followed by a final trituration with cold CH_2_Cl_2_ (3 mL × 2), drying under high *vacuum* and lyophilization to yield the desired product as a white solid (18 mg, 15%). UPLC-MS (generic method): Rt = 1.60 min, MS (ESI) *m*/*z*: 378.1 [M + H]^+^, C_19_H_20_N_7_S^+^ [M + H]^+^ calculated: 378.1. QC analysis: Rt = 2.97 min, UPLC-MS purity (UV at 215 nm): 99.5%. ^1^H NMR (600 MHz, DMSO-*d*_6_) δ 12.11 (br. s, 1H), 9.70 (br. s, 1H), 8.57 (s, 1H), 8.41 (d, *J* = 5 Hz, 1H), 7.91 (d, *J* = 3.5 Hz, 1H), 7.74 (d, *J* = 7.2 Hz, 1H), 7.32 (dd, *J* = 7.2, 5.0 Hz, 1H), 7.24 (br. s, 1H), 7.05 (br. s, 1H), 4.56 (d, *J* = 6.2 Hz, 2H), 2.27–2.22 (m, 2H), 2.09 (br. s, 2H), 1.99–1.87 (m, 2H), 1.81 (br. s, 2H). ^13^C NMR (151 MHz, DMSO-*d*_6_) δ 162.39, 160.59, 154.94, 148.78, 147.78, 136.45, 134.97, 133.46, 123.44, 123.10, 105.85, 96.08, 42.09, 31.32, 18.19.

##### *N*4-(5-Cyclopropyl-1*H*-pyrazol-3-yl)-*N*2-(pyridin-3-ylmethyl)­furo­[3,2-*d*]­pyrimidine-2,4-diamine (**26**)

Compound **26** was synthesized following the general procedure D starting from 100 mg (0.363 mmol) of intermediate **41**, 3-picolylamine (0.055 mL, 0.544 mmol) and DIPEA (0.158 mL, 0.907 mmol) dissolved in 1.81 mL of *n*-butanol and stirred for 8 h. After completion, the reaction mixture was concentrated in vacuo and the resulting crude product was purified by normal phase flash chromatography employing a 12 g gold silica cartridge (Solvent A: CH_2_Cl_2_ – Solvent B: CH_2_Cl_2_/MeOH 9:1 – Detection: 240/260 nm – Gradient: 5–45% of Solvent B). Trituration in cold CH_2_Cl_2_ (2 mL) and cold MeOH (2 mL) followed by solvent removal under high *vacuum* and lyophilization, yielded the desired product as a pale-yellow solid (17 mg, 14%). UPLC-MS (generic method): Rt = 1.41 min, MS (ESI) *m*/*z*: 348.1 [M + H]^+^, C_18_H_18_N_7_O^+^ [M + H]^+^ calculated: 348.1. QC analysis: Rt = 2.40 min, UPLC-MS purity (UV at 215 nm): 99% ^1^H NMR (600 MHz, DMSO-*d*_6_) δ 12.03 (br. s, 1H), 9.77 (br. s, 1H), 8.55 (s, 1H), 8.42 (d, *J* = 4.3 Hz, 1H), 8.02 (s, 1H), 7.73 (d, *J* = 7.8 Hz, 1H), 7.32 (dd, *J* = 7.7, 5.1 Hz, 1H), 7.19 (br. s, 1H), 6.69 (s, 1H), 6.19 (br. s, 1H), 4.51 (d, *J* = 6.1 Hz, 2H), 1.85–1.81 (m, 1H), 0.87 (d, *J* = 7.22 Hz, 2H), 0.62 (br. s, 2H). ^13^C NMR (151 MHz, DMSO-*d*_6_) δ 159.24, 151.70, 149.48, 148.77, 147.79, 144.78, 136.38, 134.97, 128.88, 123.44, 106.88, 93.58, 42.35, 7.75.

##### *N*4-(5-Cyclobutyl-1*H*-pyrazol-3-yl)-*N*2-(pyridin-3-ylmethyl)­furo­[3,2-*d*]­pyrimidine-2,4-diamine (**27**)

Compound **27** was synthesized following the general procedure D starting from 100 mg (0.345 mmol) of intermediate **42**, 3-picolylamine (0.053 mL, 0.518 mmol) and DIPEA (0.150 mL, 0.863 mmol) dissolved in 1.73 mL of *n*-butanol and stirred for 10 h. After completion, the reaction mixture was concentrated in vacuo and the resulting crude product was purified by normal phase flash chromatography employing a 12 g gold silica cartridge (Solvent A: CH_2_Cl_2_ – Solvent B: CH_2_Cl_2_/1N NH_3_ in MeOH 9:1 – Detection: 240/260 nm – Gradient: 10–30% of Solvent B). Trituration in cold CH_2_Cl_2_ (3 mL × 2) yielded the desired product as a pale-yellow solid (31 mg, 25%). UPLC-MS (generic method): Rt = 1.57 min, MS (ESI) *m*/*z*: 362.0 [M + H]^+^, C_19_H_20_N_7_O^+^ [M + H]^+^ calculated: 362.2. QC analysis: Rt = 2.78 min, UPLC-MS purity (UV at 215 nm): 99.5%. Both ^1^H and ^13^C NMR spectra in DMSO-*d*_6_ were consistent with the isolation of two different tautomeric forms a and b in a dynamic equilibrium, with a corresponding to the major and b to the minor in abundance. ^1^H NMR (600 MHz, DMSO-*d*_6_) δ 12.50 (br. s, 1Hb), 12.00 (br. s, 1Ha), 10.53 (br. s, 1Hb), 9.71 (br. s, 1Ha), 8.56 (s, 1Ha and 1Hb), 8.41 (d, *J* = 7.4 Hz, 1Ha and 1Hb), 8.02 (br. s, 1Ha and 1Hb), 7.73 (d, *J* = 7.8 Hz, 1Ha and 1Hb), 7.32 (t, *J* = 6.3 Hz, 1Ha and 1Hb), 7.13 (br. s, 1Ha and 1Hb), 6.68 (s, 1Ha and 1Hb), 6.50 (br. s, 1Hb), 5.80 (br. s, 1Ha), 4.53 (d, *J* = 6.4 Hz, 2Ha and 2Hb), 3.45–3.41 (m, 1Ha and 1Hb) 2.27–2.21 (m, 2Ha and 2Hb), 2.09 (br. s, 2Ha and 2Hb), 1.97–1.89 (m, 1Ha and 1Hb), 1.84–1.79 (m, 1Ha and 1Hb). ^13^C NMR (151 MHz, DMSO-*d*_6_) δ 159.29, 149.41, 148.77, 148.71, 147.76, 147.70, 147.67, 136.53, 134.82, 128.97, 123.34, 106.85, 106.79, 94.96, 42.34, 28.97, 18.13.

##### 3-(((4-((5-Cyclobutyl-1*H*-pyrazol-3-yl)­amino)­furo­[3,2-*d*]­pyrimidin-2-yl)­amino)­methyl)­benzamide (**28**, ARN25699)

Compound **28** (ARN25699) was synthesized following the general procedure D starting from 100 mg (0.345 mmol) of intermediate **42**, 3-(aminomethyl)­benzamide (77.79 mg, 0.518 mmol) and DIPEA (0.150 mL, 0.863 mmol) dissolved in 1.73 mL of *n*-butanol and stirred for 8 h. After good conversion of the starting material into the desired product, the reaction mixture was concentrated in vacuo. The resulting crude was purified by normal phase flash chromatography employing a 12 g gold silica cartridge (Solvent A: CH_2_Cl_2_ – Solvent B: CH_2_Cl_2_/MeOH 9:1 – Detection: 240/260 nm – Gradient: 10–40% of Solvent B). Trituration in cold CH_2_Cl_2_ (2 mL), and a sequential trituration in water (2 mL) yielded the desired product as a pale-yellow solid (18.5 mg, 13%). UPLC-MS (generic method): Rt = 1.54 min, MS (ESI) *m*/*z*: 402.2 [M – H]^−^, C_21_H_20_N_7_O_2_^–^ [M – H]^−^ calculated: 402.2. QC analysis: Rt = 2.71 min, UPLC-MS purity (UV at 215 nm): 99.5%. ^1^H NMR (400 MHz, DMSO-*d*_6_) δ 12.03 (br. s, 1H), 9.85 (br. s, 1H), 8.03 (br. s, 1H), 7.93 (br. s, 1H), 7.88 (s, 1H), 7.82 (br. s 1H), 7.72 (d, *J* = 7.5 Hz, 1H), 7.48 (d, *J* = 7.5 Hz, 1H), 7.37 (t, *J* = 7.5 Hz, 1H), 7.31 (br. s, 1H), 6.69 (s, 1H), 6.43 (br. s, 1H), 4.57 (d, *J* = 6.0 Hz, 2H), 2.28–2.17 (m, 2H), 2.07 (br. s, 2H), 1.97–1.88 (m, 1H), 1.80 (br. s, 1H). ^13^C NMR (151 MHz, DMSO-*d*_6_) δ 168.10, 159.16, 151.30, 149.52, 145.05, 141.21, 134.25, 129.82, 128.07, 126.30, 125.50, 106.72, 44.56, 29.04, 18.21.

##### 4-(5-((2-((Cyclopropylmethyl)­amino)­thieno­[3,2-*d*]­pyrimidin-4-yl)­amino)-1*H*-pyrazol-3-yl)­benzamide (**31**, ARN26646)

The salt **52** (0.016 g, 39 μmol) suspension in MeOH/MeCN 1:0.5 (2 mL) was gently heated at 40 °C to aid solubility, and purified by a cation exchange column SCX previously packed with MeOH. Once adsorbed to the resin, the product was eluted with 1N NH_3_ in MeOH obtaining the free base of the desired product 7 mg (56%). UPLC-MS (generic method): Rt = 1.65 min, MS (ESI) *m*/*z*: 404.1 [M – H]^−^, C_20_H_18_N_7_OS^–^ [M – H]^−^ calculated: 404.1. QC analysis: Rt = 2.85 min, UPLC-MS purity (UV at 215 nm): 95%. ^1^H NMR (400 MHz, DMSO-*d*_6_) δ 13.21 (br. s, 1Hb), 13.10 (br. s, 1Ha), 10.48 (br. s, 1Ha), 10.20 (br. s, 1Hb), 8.01–7.96 (m, 2H), 7.96–7.86 (m, 4H), 7.82 (d, *J* = 8.3 Hz, 1H), 7.38 (br. s, 2H), 7.12 (br. s, 1H), 6.43 (br. s, 1H), 3.22 (t, *J* = 6.2 Hz, 2H), 1.12 (s, 1H), 0.47 (d, *J* = 7.0 Hz, 2H), 0.26 (q, *J* = 4.8 Hz, 2H).^13^C NMR (151 MHz, DMSO-*d*_6_) δ 167.48, 141.78, 133.58, 124.60, 54.93, 45.49, 10.95, 3.39.

##### 2-Chloro-*N*-(1*H*-pyrazol-3-yl)­thieno­[3,2-*d*]­pyrimidin-4-amine (**35**)

Compound **35** was synthesized following the general procedure A by reaction of 2,4-dichlorothieno­[3,2-*d*]­pyrimidine (100 mg, 0.487 mmol) and 1*H*-pyrazol-5-amine (101.29 mg, 1.219 mmol) in presence of triethylamine 0.149 mL (1.073 mmol). The crude product was purified by normal phase flash chromatography employing a 12 g gold silica cartridge (Solvent A: CH_2_Cl_2_ – Solvent B: CH_2_Cl_2_/MeOH 9:1 – Detection: 240/260 nm – Gradient: 0–40% of Solvent B) to yield the desired product as a yellow solid (80.6 mg, 66%). UPLC-MS (generic method): Rt = 1.31 min; MS (ESI) *m*/*z*: 250/252 [M – H]^−^ and 252.0/254.0 [M + H]^+^, C_9_H_7_ClN_5_S^+^ [M + H]^+^ calculated: 252.0/254.0. ^1^H NMR (400 MHz, DMSO-*d*_6_) δ 12.66 (s, 1H), 10.61 (s, 1H), 8.21 (d, *J* = 5.4 Hz, 1H), 7.76 (s, 1H), 7.36 (d, *J* = 5.4 Hz, 1H), 6.59 (s, 1H).

##### 2-Chloro-*N*-(5-methyl-1*H*-pyrazol-3-yl)­thieno­[3,2-*d*]­pyrimidin-4-amine (**36**)

Compound **36** was synthesized following the general procedure A by reaction of 2,4-dichlorothieno­[3,2-*d*]­pyrimidine (100 mg, 0.487 mmol) and 3-methyl-1*H*-pyrazol-5-amine (118.39 mg, 1.219 mmol) in presence of triethylamine 0.149 mL (1.073 mmol). The crude product was purified by normal phase chromatography employing a 12 g gold silica cartridge (Solvent A: CHCl_3_ – Solvent B: CHCl_3_/MeOH 9:1 – Detection: 260/286 nm – Gradient: 0–40% of Solvent B) to yield the desired product as a white solid (66.1 mg, 51%). UPLC-MS (generic method): Rt = Rt.1.54 min; MS (ESI) *m*/*z*: 266.0/268.0 [M + H] ^+^, C_10_H_9_ClN_5_S^+^ [M + H]^+^ calculated: 266.0/268.0. ^1^H NMR (400 MHz, DMSO-*d*_6_) δ 12.34 (br. s, 1H), 10.51 (br. s, 1H), 8.20 (d, *J* = 5.4 Hz, 1H), 7.34 (d, *J* = 5.4 Hz, 1H), 6.34 (br. s, 1H), 2.27 (s, 3H).

##### 2-Chloro-*N*-(5-isopropyl-1*H*-pyrazol-3-yl)­thieno­[3,2-*d*]­pyrimidin-4-amine (**37**)

Compound **37** was synthesized following the general procedure A by reaction of 2,4-dichlorothieno­[3,2-*d*]­pyrimidine (100 mg, 0.487 mmol) and 3-isopropyl-1*H*-pyrazol-5-amine (152.4 mg, 1.2175 mmol) in presence of triethylamine 0.149 mL (1.073 mmol). The crude product was purified by normal phase chromatography employing a 12 g gold silica cartridge (Solvent A: CHCl_3_ – Solvent B: CHCl_3_/MeOH 9:1 – Detection: 240/260 nm – Gradient: 0–35% of Solvent B) to yield the desired product as a pink solid (92.2 mg, 64%). UPLC-MS (generic method): Rt = 1.82 min; MS (ESI) *m*/*z*: 294.0/296.0 [M + H]^+^, C_12_H_13_ClN_5_S^+^ [M + H]^+^ calculated: 294.0/296.0. ^1^H NMR (400 MHz, DMSO-*d*_6_) δ 12.41 (s, 1H), 10.48 (s, 1H), 8.20 (d, *J* = 5.4 Hz, 1H), 7.34 (d, *J* = 5.4 Hz, 1H), 6.33 (s, 1H), 3.15–2.75 (m, 1H), 1.25 (d, *J* = 6.9 Hz, 6H).

##### 2-Chloro-*N*-(5-cyclobutyl-1*H*-pyrazol-3-yl)­thieno­[3,2-*d*]­pyrimidin-4-amine (**38**)

Compound **38** was synthesized following the general procedure B using 2,4-dichlorothieno­[3,2-*d*]­pyrimidine 175.36 mg (0.855 mmol) and 293.2 mg (2.137 mmol) of 3-amino-5-cyclobutyl-1*H*-pyrazole in presence of triethylamine 0.262 mL (1.880 mmol) and 2.85 mL of anhydrous 2-propanol for 3 days. Precipitation in water (10 mL), filtration under *vacuum* afforded intermediate **38** as a white solid (212 mg, 81%). UPLC-MS (generic method): Rt = 1.90 min, MS (ESI) *m*/*z*: 306.0/308.0 [M + H]^+^, C_13_H_13_ClN_5_S^+^ [M – H]^+^ calculated: 306.0/308.0. ^1^H NMR (400 MHz, DMSO-*d*_6_) δ 12.45 (s, 1H), 10.46 (br. s, 1H), 8.20 (d, *J* = 5.5 Hz, 1H), 7.35 (d, *J* = 5.5 Hz, 1H), 6.40 (s, 1H), 3.53 (q, *J* = 8.6 Hz, 1H), 2.44–2.09 (m, 4H), 2.07–1.90 (m, 2H).

##### 2-Chloro-*N*-(5-phenyl-1*H*-pyrazol-3-yl)­thieno­[3,2-*d*]­pyrimidin-4-amine (**39**)

Compound **39** was synthesized following the general procedure A by reaction of 2,4-dichlorothieno­[3,2-*d*]­pyrimidine (200 mg, 0.975 mmol) and 3-phenyl-1*H*-pyrazol-5-amine (388.1 mg, 2.438 mmol) in presence of triethylamine 0.299 mL (2.145 mmol) and stirred for 3 days. The reaction mixture was precipitated in water (7 mL) and the resulting crude was purified by normal phase chromatography employing a 12 g gold silica cartridge (Solvent A: CH_2_Cl_2_ – Solvent B: CH_2_Cl_2_/MeOH 9:1 – Detection: 260/286 nm – Gradient: 10–30% of Solvent B) to yield, after precipitation of the product in CH_2_Cl_2_/cyclohexane 9:1 (10 mL), the desired product as a white solid (77 mg, 25%). UPLC-MS (generic method): Rt = Rt.1.95 min; MS (ESI) *m*/*z*: 328.0/330.0 [M + H]^+^, C_15_H_11_ClN_5_S^+^ [M + H]^+^ calculated: 328.0/330.0. ^1^H NMR (600 MHz, DMSO-*d*_6_) δ 13.22 (s, 1H), 10.68 (s, 1H), 8.23 (d, *J* = 5.0 Hz, 1H), 7.77 (d, *J* = 7.1 Hz, 2H), 7.49 (t, *J* = 7.1 Hz, 2H), 7.38 (m, 2H), 6.99 (s, 1H).

##### 2-Chloro-*N*-(5-(pyridin-3-yl)-1*H*-pyrazol-3-yl)­thieno­[3,2-*d*]­pyrimidin-4-amine (**40**)

Compound **40** was synthesized following the general procedure B using 2,4-dichlorothieno­[3,2-*d*]­pyrimidine (0.976 mmol) and 0.392 g (2.438 mmol) of 3-(pyridin-3-yl)-1*H*-pyrazol-5-amine in presence of triethylamine (0.296 mL, 2.14 mmol) and 4.0 mL of anhydrous 2-propanol for 4 days. Precipitation in water (10 mL), filtration under *vacuum* and sequential trituration in EtOH (7 mL) afforded intermediate **40** as a white solid (64 mg, 20%). UPLC-MS (generic method): Rt = 1.56 min, MS (ESI) *m*/*z*: 327/329 [M – H]^−^, C_14_H_8_ClN_6_S^–^ [M – H]^−^ calculated: 327.02/329.02. ^1^H NMR (400 MHz, DMSO-*d*_6_) δ 13.39 (br. s, 1H), 10.73 (br. s, 1H), 9.01 (br. s, 1H), 8.57 (d, *J* = 3.5 Hz, 1H), 8.24 (d, *J* = 5.4 Hz, 1H), 8.15 (d, *J* = 8.0 Hz, 1H), 7.52 (br. s, 1H), 7.39 (d, *J* = 5.4 Hz, 1H), 7.11 (br. s, 1H).

##### 2-Chloro-*N*-(5-cyclopropyl-1*H*-pyrazol-3-yl)­furo­[3,2-*d*]­pyrimidin-4-amine (**41**)

Intermediate **41** was obtained starting from 2,4-dichlorofuro­[3,2-*d*]­pyrimidine 150 mg (0.794 mmol) and 243.86 mg (1.98 mmol) of 3-cyclopropyl-1*H*-pyrazol-5-amine in presence of triethylamine (0.243 mL, 1.75 mmol) and 2.65 mL of 2-propanol at room temperature under Ar for 3 days according to the general procedure B. Precipitation in water (10 mL) afforded intermediate **41** as a bright yellow solid (170 mg, 81%). UPLC-MS (generic method): Rt = 1.57 min, MS (ESI) *m*/*z*: 276.0/278.0 [M + H]^+^, C_12_H_11_ClN_5_O^+^ [M + H]^+^ calculated: 276.0/278.0. ^1^H NMR (400 MHz, DMSO-*d*_6_) δ 12.23 (s, 1H), 10.57 (s, 1H), 8.33 (d, *J* = 2.0 Hz, 1H), 7.00 (d, *J* = 2.0 Hz, 1H), 6.29 (s, 1H), 1.96–1.89 (m, 1H), 0.94 (dq, *J* = 4.5, 1.7 Hz, 2H), 0.70 (dq, *J* = 4.5, 1.7 Hz, 2H). ^13^C NMR (101 MHz, DMSO-*d*_*6*_) δ 153.18, 152.17, 151.60, 146.29, 132.55, 107.21, 94.83, 7.73, 7.53, 6.87.

##### 2-Chloro-*N*-(5-cyclobutyl-1*H*-pyrazol-3-yl)­furo­[3,2-*d*]­pyrimidin-4-amine (**42**)

Intermediate **42** was obtained starting from 2,4-dichlorofuro­[3,2-*d*]­pyrimidine 200 mg (1.058 mmol) and 362.9 mg (2.645 mmol) of 3-amino-5-cyclobutyl-1*H*-pyrazole in presence of triethylamine (0.324 mL, 2.328 mmol) and 3.53 mL of 2-propanol at room temperature under an inert atmosphere, for 3 days according to the general procedure B. Precipitation in water (10 mL) afforded intermediate **42** as a yellow solid (235.6 mg, 77%). The product was employed in the next steps without any further purification. UPLC-MS: Rt = 1.87 min, MS (ESI) *m*/*z*: 290.0/292.0 [M + H]^+^, C_13_H_13_ClN_5_O^+^ [M + H]^+^ calculated: 290.1/292.1. ^1^H NMR (400 MHz, DMSO-*d*_6_) δ 12.29 (s, 1H), 10.61 (s, 1H), 8.32 (d, *J* = 2.2 Hz, 1H), 7.00 (d, *J* = 2.2 Hz, 1H), 6.44 (s, 1H), 3.62–3.43 (m, 1H), 2.34–2.26 (m, 2H), 2.19–2.20 (m, 2H), 2.02– 2.91 (m, 1H), 1.89–1.80 (m, 1H).

##### 2-Chloro-*N*-(5-cyclopropyl-1*H*-pyrazol-3-yl)-6,7-dihydrothieno­[3,2-*d*]­pyrimidin-4-amine (**43**)

Compound **43** was synthesized following the general procedure B using 2,4-dichloro-6,7-dihydrothieno­[3,2-*d*]­pyrimidine 0.200 g (0.966 mmol) and 0.297 g (2.415 mmol) of 3-cyclopropyl-1*H*-pyrazol-5-amine in presence of triethylamine (0.296 mL, 2.125 mmol) and 3.22 mL of anhydrous 2-propanol at 80 °C under Ar, for 3 days. Precipitation in water (10 mL) afforded intermediate **43** as a white solid (145.8 mg, 52%). UPLC-MS (generic method): Rt = 1.65 min, MS (ESI) *m*/*z*: 294.0/296.0 [M + H]^+^, C_12_H_13_ClN_5_S^+^ [M + H]^+^ calculated: 294.1/296.1. ^1^H NMR (400 MHz, DMSO-*d*_6_) δ 12.21 (s, 1H), 9.45 (s, 1H), 6.10 (s, 1H), 3.17 (t, *J* = 8.7, 7.1 Hz, 2H), 1.88 (m, 1H), 0.92 (d, *J* = 7.24 Hz, 2H), 0.68 (m, 2H).

##### (4-((*tert*-Butyldiphenylsilyl)­oxy)­phenyl)­methanamine (**44**)

*tert*-Butyldiphenylsilyl chloride (0.158 mL, 0.609 mmol) was added dropwise to a stirred suspension of 4-hydroxybenzylamine (50 mg, 0.406 mmol) in THF (1.624 mL) at room temperature under Ar. Imidazole (55.28 mg, 0.812 mmol) was subsequently added and the reaction mixture was stirred for 10 h. Subsequently, water (5 mL) was added and the resulting mixture was extracted with EtOAc (5 mL × 3). Combined organic layers were dried over Na_2_SO_4_ and concentrated under high *vacuum*. The crude product was purified by normal phase flash chromatography employing a 12 g gold silica cartridge (Solvent A: CHCl_3_ – Solvent B: CHCl_3_/MeOH 8:2 – Detection: 240/260 nm – Gradient: 10–20% of Solvent B) to yield the pure product as a white dense solid (78.2 mg, 58%). UPLC-MS (apolar method): Rt = 1.44 min, C_23_H_28_NOSi^+^ [M + H]^+^ calculated: 362.2 (no ionization observed).^1^H NMR (600 MHz, DMSO-*d*_6_) δ 7.69–7.65 (m, 4H), 7.63 (br. s, 2H), 7.51–7.45 (m, 2H), 7.45–7.41 (m, 4H), 7.08–7.05 (m, 2H), 6.66–6.64 (m, 2H), 4.12 (br. s, 2H), 1.03 (s, 9H).

##### *N*2-(4-((*tert*-Butyldiphenylsilyl)­oxy)­benzyl)-*N*4-(5-cyclopropyl-1*H*-pyrazol-3-yl)­thieno­[3,2-*d*]­pyrimidine-2,4-diamine (**45**)

Compound **45** was synthesized following the general procedure D, starting from 140.92 mg (0.483 mmol) of intermediate **34**, TBDPS-protected intermediate **44** (262 mg, 0.725 mmol) and DIPEA (0.210 mL, 1.2077 mmol) dissolved in 2.41 mL of *n*-butanol and stirred for 6 h. After completion, the reaction mixture was concentrated in vacuo. Purification of the resulting crude by normal phase flash chromatography employing a 12 g gold silica cartridge (Solvent A: CHCl_3_ – Solvent B: CHCl_3_/MeOH 9:1 – Detection: 240/260 nm – Gradient: 0–30% of Solvent B), to yield the desired product as a white solid (220 mg, 73%). UPLC-MS apolar method: Rt = 2.43 min, MS (ESI) *m*/*z*: 615.4 [M – H]^−^, C_35_H_35_N_6_OSSi^–^ [M – H]^−^ calculated: 615.2. Both ^1^H and ^13^C NMR spectra in DMSO-*d*_6_ were consistent with the isolation of two different tautomeric forms a and b in a dynamic equilibrium, with a corresponding to the major and b to the minor in abundance. ^1^H NMR (600 MHz, DMSO-*d*_6_) δ 12.55 (br. s, 1Hb), 12.05 (br. s, 1Ha), 10.26 (br. s, 1Hb), 9.67 (br. s, 1Ha), 7.87 (br. s, 1Ha and 1Hb), 7.59–6.63 (m, 4Ha and 4Hb), 7.49–7.40 (m, 6Ha and 6Hb), 7.10 (br. s, 1Ha and 1Hb), 7.07 (br. s, 2Ha and 2Hb) 6.67 (br. s, 1Ha and 1Hb), 6.66 (br. s, 2Hb and 2Ha) 6.32 (br. s, 1Hb), 5.65 (br. s, 1Ha), 4.40 (d, *J* = 6.1 Hz, 2Ha and 2Hb), 1.80 (br. s, 1Ha and 1Hb), 1.03 (br. s, 9Ha and 9Hb), 0.85 (br. s, 2Ha and 2Hb), 0.62 (br. s, 2Ha and 2Hb).

##### *tert*-Butyl (4-carbamoylbenzyl)­carbamate (**46**)

In a 50 mL round flask under Ar, 4-(aminomethyl) benzoic acid (0.2 g, 1.323 mmol) in anhydrous 1,4-dioxane (13.23 mL) was stirred at room temperature. Di-*tert*-butyl dicarbonate (4.33 g, 19.84 mmol) and pyridine (0.534 mL, 6.615 mmol) were added to the suspension followed by addition of ammonium bicarbonate (1.57 g, 19.84 mmol). The resulting mixture was heated under MW irradiation at 105 °C for 3 h and once completed concentrated under *vacuum* and purified by normal phase chromatography employing a 24 g gold silica cartridge (Solvent A: CH_2_Cl_2_ – Solvent B: CH_2_Cl_2_/ EtOH 9:1 – Detection: 240/280 nm – Gradient: 10–40% of Solvent B) to yield the desired product as a white solid (156 mg, 47%). UPLC-MS (generic method): Rt = 1.51 min, MS (ESI) *m*/*z*: 251.1 [M + H]^+^, C_13_H_19_N_2_O_3_^+^ [M + H]^+^ calculated: 251.1. ^1^H NMR (400 MHz, DMSO-*d*_6_) δ 7.90 (br. s, 1H), 7.81 (d, *J* = 8.2 Hz, 2H), 7.42 (t, *J* = 6.1 Hz, 1H), 7.28 (d, *J* = 8.2 Hz, 3H), 4.16 (d, *J* = 6.1 Hz, 2H), 1.39 (s, 9H).

##### 4-(Aminomethyl)­benzamide, Trifluoroacetic Salt (**47**)

In a round flask, intermediate **46** (200 mg, 0.799 mmol) was dissolved in 7.7 mL of anhydrous CH_2_Cl_2_ at 0 °C. Trifluoroacetic acid (0.9 mL, 11.7 mmol) was added dropwise while stirring and the reaction was stirred at room temperature for 1 h. Once completed, 7.7 mL of cold diethyl ether were added to the solution and the resulting white precipitate was filtered under *vacuum* yielding 175.9 mg (83%) of the pure product as a white solid, which was employed in the following step without any further purification. ^1^H NMR (400 MHz, DMSO-*d*_6_) δ 8.30 (br. s, 3H), 8.01 (br. s, 1H), 7.91 (d, *J* = 8.3 Hz, 2H), 7.52 (d, *J* = 8.3 Hz, 2H), 7.41 (br. s, 1H), 4.09 (s, 2H).

##### 2-Chlorothieno­[3,2-*d*]­pyrimidin-4­(3*H*)-one (**48**)

2,4-Dichlorothieno­[3,2-*d*]­pyrimidine (1 g, 5 mmol) was dissolved in a mixture of anhydrous tetrahydrofuran (8 mL) and water (2 mL), sodium hydroxide 6 M solution (2 mL, 10 mmol) was added dropwise to the reaction mixture, which was heated up to 50 °C and stirred at the same temperature overnight. The reaction was monitored by TLC in CH_2_Cl_2_/MeOH 9.5:0.5, noticing complete conversion of the starting material. Thus, the temperature was cooled down to 35 °C and AcOH (0.6 mL, 10 mmol) was added dropwise and the resulting mixture was stirred for 2 h. After 2 h, it was quenched with water (10 mL) and extracted three times with EtOAc (30 mL), washed with brine and dried over Na_2_SO_4_. The resulting organic layer was concentrated under *vacuum* to obtain **48** (0.9 g, 99%), which was used in the next step without any further purification. UPLC-MS (generic method): Rt = 1.04 min, MS (ESI) *m*/*z*: 186.8/188.7 [M + H]^+^, C_6_H_4_ClN_2_OS^+^ [M + H]^+^ calculated: 187.0/189.0. ^1^H NMR (600 MHz, DMSO-*d*_6_) δ 8.22 (d, *J* = 4.6 Hz, 1H), 7.37 (d, *J* = 4.6 Hz, 1H).

##### 2-((Cyclopropylmethyl)­amino)­thieno­[3,2-*d*]­pyrimidin-4­(3*H*)-one (**49**)

In a MW vial, **48** (150 mg, 0.80 mmol) was dissolved in anhydrous *n*-butanol (4.03 mL). DIPEA (0.35 mL, 2.01 mmol) and 1-cyclopropylmethanamine (0.07 mL, 0.80 mmol) were sequentially added and the reaction was left stir for 6 h at 160 °C under MW irradiation. The reaction was set up three times in parallel in the same experimental conditions. When LC-MS and TLC (CH_2_Cl_2_/MeOH 9.5:0.5) confirmed complete conversion of the starting material, the reaction mixtures were mixed together and concentrated under *vacuum*. Purification via flash chromatography 100% CH_2_Cl_2_ gave **49** (0.53 g, 99%) as a yellow powder. UPLC-MS (generic method): Rt = 1.47 min, MS (ESI) *m*/*z*: 221.8 [M + H]^+^, C_10_H_12_N_3_OS^+^ [M + H]^+^ calculated: 222.1. ^1^H NMR (600 MHz, DMSO-*d*_6_) δ 10.79 (s, 1H), 7.95 (d, *J* = 5.2 Hz, 1H), 7.05 (d, *J* = 5.2 Hz, 1H), 6.32 (t, *J* = 5.4 Hz, 1H), 3.15 (t, *J* = 6.2 Hz, 2H), 1.08–1.04 (m, 1H), 0.47–0.44 (m, 2H), 0.25–0.22 (m, 2H).

##### 4-Chloro-*N*-(cyclopropylmethyl)­thieno­[3,2-*d*]­pyrimidin-2-amine (**50**)

In an oven-dried pressure tube under N_2_ atmosphere, a catalytic amount of dimethyl sulfoxide (1 μL, 14.08 μL) was added to a suspension of **49** (200 mg, 0.904 mmol) in phosphoryl trichloride (1.77 mL, 19.0 mmol). The resulting mixture was stirred for 3 h at 110 °C. When complete conversion of the starting material was observed by TLC (CH_2_Cl_2_/MeOH 9.5:0.5), the reaction was cooled down to 0 °C and quenched by addition of ice. The aqueous layer was extracted with EtOAc (20 mL × 3). The combined organic layers were washed with brine and dried over Na_2_SO_4_. The crude was purified by silica gel chromatography employing 100% CH_2_Cl_2_ as a solvent to yield the desired product as a white solid (94 mg, 43%). UPLC-MS (generic method): Rt = 2.40 min, MS (ESI) *m*/*z*: 239.9/241.9 [M + H]^+^, C_10_H_11_ClN_3_S^+^ [M + H]^+^ calculated: 240.0/242.0. ^1^H NMR (600 MHz, CDCl_3_) δ 7.81 (d, *J* = 5.2 Hz, 1H), 7.19 (d, *J* = 5.2 Hz, 1H), 5.38 (br. s, 1H), 3.32 (t, *J* = 5.2 Hz, 2H), 1.12–1.05 (m, 1H), 0.54 (d, *J* = 7.4 Hz, 2H), 0.27 (d, *J* = 5.0 Hz, 2H).

##### 4-(5-Amino-1*H-*pyrazol-3-yl)­benzamide (**51**)

In a oven-dried MW vial, 3-bromo-1*H*-pyrazol-5-amine (150 mg, 0.926 mmol), (4-carbamoylphenyl)­boronic acid (305 mg, 1.85 mmol), tetrakis­(triphenylphosphine)­palladium(0) (161 mg, 0.139 mmol) were added. The solid mixture was suspended in anhydrous 1,4-dioxane (1.85 mL) and a 2 M solution of potassium carbonate (2.31 mL, 4.63 mmol) was added. The reaction mixture was degassed and backfilled with N_2_ and stirred for 2 h at 130 °C under MW irradiation. The reaction was quenched with water (5 mL) and washed with EtOAc (10 mL × 3, to remove apolar impurities). The aqueous phase was concentrated under *vacuum* and purified by silica gel chromatography (Solvent A: CH_2_Cl_2_ – Solvent B: MeOH – Gradient 10–20% of MeOH) followed by a sequential purification (Solvent A: EtOH – Solvent B: MeOH – Gradient: 0–20% of Solvent B) to yield the desired product as a pale-yellow solid (95 mg, 51%). UPLC-MS (generic method): Rt = 0.84 min, MS (ESI) *m*/*z*: 202.9 [M + H]^+^, C_10_H_11_N_4_O^+^ [M + H]^+^ calculated: 201.1. ^1^H NMR (600 MHz, DMSO-*d*_6_) δ 7.86 (d, *J* = 8.3 Hz, 2H), 7.71 (d, *J* = 8.3 Hz, 2H), 7.32 (br. s, 1H), 5.82 (s, 1H), 4.87 (br. s, 2H).

##### 4-(5-((2-((Cyclopropylmethyl)­amino)­thieno­[3,2-*d*]­pyrimidin-4-yl)­amino)-1*H*-pyrazol-3-yl)­benzamide, Formic Acid Salt (**52**)

In a MW vial, 4-chloro-*N*-(cyclopropylmethyl)­thieno­[3,2-*d*]­pyrimidin-2-amine **50** (94 mg, 0.39 mmol) and 4-(5-amino-1*H*-pyrazol-3-yl)­benzamide (**51**) (0.12 g, 0.59 mmol) were dissolved in anhydrous *n*-butanol (2.0 mL) under inert atmosphere (N_2_). DIPEA (51 mg, 68 μL, 0.39 mmol) was added to the solution and the reaction was left go for 8 h at 180 °C under MW irradiation according to the general chemical procedure D. When TLC (CH_2_Cl_2_/MeOH 9.5:0.5) confirmed complete conversion of the starting material, the reaction mixture was concentrated under vacuum. Purification via flash chromatography (Solvent A: CH_2_Cl_2_ – Solvent B: MeOH – Gradient: 0–10% of Solvent B) followed by purification via HPLC-prep (H_2_O/MeCN 0.1% HCOOH from 5 to 95% of MeCN in 18 min, flow rate: 30 min/mL) yielded the formic acid salt of the desired product as a pale yellow solid (16 mg, 10%). UPLC-MS (generic method): Rt = 1.60 min, MS (ESI) *m*/*z*: 406.0 [M + H]^+^, C_20_H_20_N_7_OS^+^ [M + H]^+^ calculated: 406.1. ^1^H NMR (400 MHz, DMSO-*d*_6_) δ 8.13 (s, 1H), 8.06 (br. s, 1H), 8.05 (br. s, 1H), 7.99–7.90 (m, 2H), 7.86-7.88 (m, 2H), 7.81 (d, *J* = 8.4 Hz, 1H), 7.41 (br. s, 1H), 7.20 (d, *J* = 5.4 Hz, 1H), 1.16 (br. s, 1H), 0.52–0.48 (m, 2H), 0.30–0.26 (m, 2H).

#### Human CMGC Kinase Enzymatic Radiometric Assay [Km ATP], KinaseProfiler (Performed at Eurofins Cerep, Poitiers, France)

h-FYN is incubated with 50 mM Tris pH 7.5, 0.1 mM EGTA, 0.1 mM Na_3_VO_4_, 250 μM KVEKIGEGTYGVVYK (Cdc2 peptide), 10 mM magnesium acetate, and [gamma-33P]-ATP (specific activity and concentration as required). The reaction is initiated by the addition of the Mg/ATP mix. After incubation for 40 min at room temperature, the reaction is stopped by the addition of phosphoric acid to a concentration of 0.5%. An aliquot of the reaction is then spotted onto a filter and washed four times for 4 min in 0.425% phosphoric acid and once in methanol prior to drying and scintillation counting.

h-GSK-3β is incubated with 8 mM MOPS pH 7.0, 0.2 mM EDTA, 20 μM YRRAAVPPSPSLSRHSSPHQS­(p) EDEEE (phospho GS2 peptide), 10 mM magnesium acetate, and [gamma-33P]-ATP (specific activity and concentration as required). The reaction is initiated by the addition of the Mg/ATP mix. After incubation for 40 min at room temperature, the reaction is stopped by the addition of phosphoric acid to a concentration of 0.5%. An aliquot of the reaction is then spotted onto a filter and washed four times for 4 min in 0.425% phosphoric acid and once in methanol prior to drying and scintillation counting.

h-DYRK1A is incubated with 8 mM MOPS pH 7.0, 0.2 mM EDTA, 50 μM RRRFRPASPLRGPPK, 10 mM magnesium acetate, and [gamma-33P]-ATP (specific activity and concentration as required). The reaction is initiated by the addition of the Mg/ATP mix. After incubation for 40 min at room temperature, the reaction is stopped by the addition of phosphoric acid to 21 a concentration of 0.5%. An aliquot of the reaction is then spotted onto a filter and washed four times for 4 min in 0.425% phosphoric acid and once in methanol prior to drying and scintillation counting.

h-CDK5/p25 is incubated with 8 mM MOPS pH 7.0, 0.2 mM EDTA, 0.1 mg/mL histone H1, 10 mM magnesium acetate, and [g-33P]-ATP (specific activity and concentration as required). The reaction is initiated by the addition of the Mg/ATP mix. After incubation for 40 min at room temperature, the reaction is stopped by the addition of phosphoric acid to a concentration of 0.5%. An aliquot of the reaction is then spotted onto a filter and washed four times for 4 min in 0.425% phosphoric acid and once in methanol prior to drying and scintillation counting.

For all tested PKs the Pan-kinase inhibitor staurosporine was used as a reference compound.

##### GSK-3β, FYN-α, and DYRK-1A Kinase Assays

GSK-3β, FYN-α, and DYRK1A kinase assays were run in 384-well microplates (OptiPlateTM-384, White, Perkin-Elmer) in a total reaction volume of 20 μL. The inhibitory potency against human recombinant GSK-3β, FYN-α, and DYRK1A (Carna Biosciences) was evaluated using the LANCE Ultra (Perkin-Elmer) time-resolved fluorescence resonance energy transfer (TR-FRET) by measuring the phosphorylation of the ULight-labeled substrate, according to the manufacturer’s instructions. The synthetic peptide surrounding Ser641 of human Muscle Glycogen Synthase (ULight-GS (Ser641/pSer657)) was used as the substrate for GSK-3β and DYRK1A, while the synthetic 28-amino acid peptide containing eight Tyr residues placed in different amino acid contexts (ULightTM-TK (PT66)) was selected as the substrate for FYN-α. Briefly, test compounds, staurosporine or harmine (reference compounds for GSK-3β/Fyn-α and DYRK1A, respectively) or DMSO (control) are mixed with the enzyme (GSK-3β: 2 nM, DYRK1A: 4 nM and FYN-α: 1 nM) in a buffer containing 50 mM Hepes (pH 7.5), 1 mM EGTA, 10 mM MgCl_2_, 2 mM DTT and 0.01% Tween-20. The reaction is initiated by adding 50 nM of the substrate and ATP at a final concentration, determined experimentally for each new ATP stock solution prepared, near the K_m_ value of the enzyme for ATP (e.g., GSK-3β: 1.2 μM; DYRK1A: 1.7 μM or FYN-α: 8 μM), and the mixture is incubated for 60 (GSK-3β and DYRK1A) or 90 min (FYN-α), at 23 °C. Following incubation, the reaction is stopped by adding 8 mM EDTA. After 5 min, the antiphospho antibody labeled with europium chelate is added. After 1 more hour, the kinase reaction is monitored by irradiation at 320 nm, and the fluorescence is measured at 615 and 665 nm, using EnVision 2014 Multilabel Reader (PerkinElmer). The calculated signal ratio at 665/615 nm is proportional to the extent of ULight-substrate phosphorylation. The compounds were tested at 11 different concentrations (range 100 pM–10 μM for GSK-3β and FYN-α and 1 nM–100 μM for DYRK1A), in technical triplicates. For a few compounds with poor solubility or potency, the percentage of inhibition at one concentration (5 μM) was determined. The results were expressed as a percent inhibition of the control enzyme activity.

**Data.** Dose–response curves were run at least in three independent experiments, performed in three technical replicates. IC_50_ values (concentrations causing half-maximal enzyme inhibition) were determined by nonlinear regression analysis of the Log [concentration]/response curves generated with mean replicate values using a four-parameter Hill equation curve fitting with GraphPad Prism 8 (GraphPad Software Inc., CA-USA).

#### Tau Phosphorylation Assay in Human Recombinant Tau0N4R-TM-tGFP U2OS Cells, Performed at Innoprot (Spain)

Dose–response assays were performed using a cellular fluorescence bundle formation assay after compound addition in a human recombinant Tau0N4R-TM-tGFP U2OS stable cell line. The formation of tau and MT bundles after treatment was measured in triplicate. Medium (OptiMem) and Vehicle (DMSO) were used as negative controls, and 10 or 30 mM LiCl as a positive control (depending on the experimental setting). This bundle increase is detected and quantified by fluorescence using automated image analysis (Cell Insight CX7 from ThermoFisher). Results are expressed as the bundle average area per cell normalized with respect to the vehicle. Data points represent the mean ± SD for each condition for a single experiment performed in triplicate. Two-way ANOVA was used to evaluate statistical significance, followed by Bonferroni’s post hoc test. GraphPad Prism 8 was used for statistical analysis (GraphPad Software Inc., San Diego, CA, USA). P values less than 0.05 were considered significant.

#### In Vitro ADME-PK Evaluation

##### Aqueous Kinetic Solubility

The aqueous kinetic solubility was determined from a 10 mM DMSO stock solution of the test compound in Phosphate Buffered Saline (PBS) at pH 7.4. The study was performed by incubation of an aliquot of 10 mM DMSO stock solution in PBS (pH 7.4) at a target concentration of 250 μM (2.5% DMSO). The incubation was carried out under shaking at 25 °C for 24 h, followed by centrifugation at 21,100 *g* for 30 min. The supernatant was further diluted (4:1) with MeCN and analyzed by UPLC-MS for the quantification of dissolved compound (in μM) by UV at a specific wavelength (215 nm). The aqueous kinetic solubility (in μM) was calculated by dividing the peak area of the dissolved test compound (supernatant) by the peak area of the test compound in the reference (250 μM in MeCN) and further multiplying by the target concentration and dilution factor. The UPLC-MS analyses were performed on a Waters ACQUITY UPLC-MS system consisting of a single quadrupole detector (SQD) mass spectrometer equipped with an electrospray ionization (ESI) interface and a photodiode array detector (PDA) from Waters Inc. (Milford, MA, USA). The PDA range was 210–400 nm. ESI in positive mode was used in the mass scan range 100–650 Da. The analyses were run on an ACQUITY UPLC BEH C_18_ column (50 × 2.1 mm ID, particle size 1.7 μm) with a VanGuard BEH C_18_ precolumn (5 × 2.1 mm ID, particle size 1.7 μm), using 10 mM NH_4_OAc in H_2_O at pH 5 adjusted with AcOH (A) and 10 mM NH_4_OAc in MeCN-H_2_O (95:5) at pH 5 (B) as mobile phase.

##### In Vitro Mouse Plasma Stability

A ten mM DMSO stock solution of the test compound was diluted 50-fold with DMSO-H_2_O (1:1) and incubated at 37 °C for 2 h with mouse plasma containing 5% DMSO (preheated at 37 °C for 10 min). The final compound concentration was 2 μM (0.5% DMSO). At each time point (0, 5, 15, 30, 60, 120 min), an aliquot of the incubation mixture was diluted (1:3) with cold MeCN spiked with 200 nM of an appropriate internal standard, followed by centrifugation at 3270 *g* for 20 min. The supernatant was further diluted (1:1) with H_2_O and analyzed by LC-MS/MS on a Waters ACQUITY UPLC-MS/MS system consisting of a triple quadrupole detector (TQD) mass spectrometer equipped with an electrospray ionization interface (ESI) and a photodiode array detector (PDA) from Waters Inc. (Milford, MA, USA). Electrospray ionization was applied in positive mode. Compound-dependent parameters, such as MRM transitions and collision energy, were developed for each compound. The analyses were run on an ACQUITY UPLC BEH C_18_ (50 × 2.1 mm ID, particle size 1.7 μm) with a VanGuard BEH C_18_ precolumn (5 × 2.1 mm ID, particle size 1.7 μm) at 40 °C, using H_2_O + 0.1% HCOOH (A) and MeCN + 0.1% HCOOH (B) as mobile phase. The percentage of test compound remaining at each time point relative to *t* = 0 was calculated by the response factor based on the internal standard peak area. The percentage of test compound versus time was plotted and fitted by GraphPad Prism (GraphPad Software, Version 5 for Windows, CA, USA, www.graphpad.com) to estimate the compound’s half-life (*t*_1/2_), which was reported as a mean value along with the standard deviation (*n* = 3).

##### In Vitro Mouse Liver Microsomal Stability

Ten mM DMSO stock solution of the test compound was preincubated at 37 °C for 15 min with mouse liver microsomes in 0.1 M Tris-HCl buffer (pH 7.5) containing 10% DMSO. The final compound concentration was 5 μM (0.1% DMSO). After preincubation, the cofactors (NADPH, G6P, G6PDH and MgCl_2_ predissolved in 0.1 M Tris-HCl) were added to the incubation mixture, and the incubation was continued at 37 °C for 1 h. At each time point (0, 5, 15, 30, 60 min), 30 μL of incubation mixture was diluted with 200 μL cold CH_3_CN spiked with 200 nM of an appropriate internal standard, followed by centrifugation at 3,270 *g* for 15 min. The supernatant was further diluted (1:1) with H_2_O for analysis. A reference incubation mixture (microsomes *without* cofactors) was prepared for each test compound and analyzed at *t* = 0 and 60 min in order to verify the compound’s stability in the matrix. The two time points were diluted as for the time points of the incubation mixture above. The supernatants were analyzed by LC-MS/MS on a Waters ACQUITY UPLC-MS/MS system as defined above. The percentage of test compound remaining at each time point relative to *t* = 0 was calculated by the response factor based on the internal standard peak area. The percentage of test compound versus time was plotted and fitted by GraphPad Prism (GraphPad Software, Version 5 for Windows, CA, USA, www.graphpad.com) to estimate the compound’s half-life (*t*_1/2_), which was reported as a mean value along with the standard deviation (*n* = 3).

##### In Vitro Human Liver Microsomal Stability

A ten mM DMSO stock solution of the test compound was preincubated at 37 °C for 15 min with human liver microsomes in 0.1 M Tris-HCl buffer (pH 7.5) containing 10% DMSO. The final compound concentration was 5 μM (0.1% DMSO). After preincubation, the cofactors (NADPH, G6P, G6PDH and MgCl_2_ predissolved in 0.1 M Tris-HCl) were added to the incubation mixture, and the incubation was continued at 37 °C for 1 h. At each time point (0, 5, 15, 30, 60 min), 30 μL of incubation mixture was diluted with 200 μL cold CH_3_CN spiked with 200 nM of an appropriate internal standard, followed by centrifugation at 3270 *g* for 15 min. The supernatant was further diluted (1:1) with H_2_O for analysis. A reference incubation mixture (microsomes *without* cofactors) was prepared for each test compound and analyzed at *t* = 0 and 60 min in order to verify the compound’s stability in the matrix. The two time points were diluted as for the time points of the incubation mixture above. The supernatants were analyzed by LC-MS/MS on a Waters ACQUITY UPLC-MS/MS system as defined above. The percentage of test compound remaining at each time point relative to *t* = 0 was calculated by the response factor on the basis of the internal standard peak area. The percentage of test compound versus time was plotted and fitted by GraphPad Prism (GraphPad Software, Version 5 for Windows, CA, USA, www.graphpad.com) to estimate the compound’s half-life (*t*_1/2_), which was reported as a mean value along with the standard deviation (*n* = 3).

##### MetID and MLM Stability

Samples of test compound at 5 μM were incubated for 0, 5, 15, 30, and 60 min at 37 °C in a 0.1 M phosphate buffer (pH 7.4) containing MLM (0.5 mg/mL, pool of 10 donors; Gibco cat. MSMCPL, Lot #MS065). The reactions were started by the addition of 1 mM NADPH. At each time point, an aliquot of the reaction mixture was taken and quenched with ice-cold acetonitrile 3:1 (containing 1 μM labetalol as an internal standard). Proteins were precipitated by centrifugation at 21,000 *g* for 10 min at 4 °C. The supernatant was further diluted 1:1 with H_2_O for analysis. Blank sample was prepared by incubating the cell solution without any compound for 60 min. No CYP enzymatic activity was evaluated by incubating compounds in the same reaction mixture without NADPH. Metabolite identification was performed by LC/MS-MS. For the data set, chromatographic separation of metabolites was performed using a Thermo Ultimate 3000 UPLC system with a Phenomenex Luna Omega C18 column (1.6 μm, 2.1 × 150 mm) in positive polarity. The mobile phases consisted of 0.1% formic acid in water (A) and acetonitrile + 0.1% formic acid (B), respectively. The LC gradient was as follows: 0–10 min 0–95% B, 10–12 min 95%, and (post time) 12–14 min 95–5% B, at a flow rate of 0.4 mL/min. Full MS scans were acquired in the Orbitrap Q-Exactive mass over the *m*/*z* 100–800 range with a resolution of 70000, with an automatic gain control (ACG) setting of 1e6 and maximum injection time of 300 ms. Peaks were fragmented in the HCD collision cell with a normalized collision energy of 30%, and a tandem mass spectrum was acquired in the Orbitrap mass analyzer with a resolution of 17500, ACG of 5e5, and a max injection time of 80 ms. Full scan MS/MS was a data-dependent acquisition (DDA) using a specific inclusion list generated using the software Mass-MetaSite 5.1.9 (MolDiscovery). The DDA method setting employed a minimum ACG target of 4e2, intensity threshold of 5e3, apex trigger between 2 and 3 s, isotopes excluded, and dynamic exclusion of 2 s.

##### In Vivo Pharmacokinetic Measurements

Male CD1 mice, 8 weeks old, were used (Charles River). All procedures were performed in compliance with the Ethical Guidelines of the European Communities Council (Directive 2010/63/EU of 22 September 2010) and accepted by the Italian Ministry of Health (N. 769/2022-PR). All efforts were made to minimize animal suffering and to use the minimal number of animals required to produce reliable results, according to the “3Rs concept”. Animals were group-housed in ventilated cages and had free access to food and water. They were maintained under a 12-h light/dark cycle (lights on at 8:00 am) at controlled temperature (21 °C ± 1 °C) and relative humidity (55% ± 10%). **1** – ARN25068 and **28** – ARN25068 were administered p.o. and i.v. to CD1 male mice at 10 and 3 mg/kg. The vehicle used was PEG400/Tween 80/saline solution at 10/10/80% in volume, respectively. Three animals per time point were treated. Blood samples at 0, 15, 30, 60, 120, and 240 minutes after administration were collected for the P.O. arm. Blood samples at 0, 5, 15, 30, 60, 120, and 240 min after administration were collected for the I.V. arm. Plasma was separated from blood by centrifugation for 15 min at 1500 rpm a 4 °C, transferred to Eppendorf tubes, and frozen (−80 °C). Control animals treated with vehicle only were also included in the experimental protocol.

*Plasma:* Plasma samples were centrifuged at 21.100*g* for 15 min at 4 °C. An aliquot of each plasma sample was extracted (1:3) with cold MeCN containing 200 nM of an appropriate internal standard. A calibration curve was prepared in blank mouse plasma over a 1 nM–10 μM range. Three quality control samples were prepared by spiking the parent compound in blank mouse plasma to 20, 200, and 2000 nM as final concentrations. The calibrators and quality control samples were extracted (1:3) with the same extraction solution as the plasma samples. The plasma samples, calibrators, and quality control samples were centrifuged at 3270 *g* for 15 min at 4 °C.

*Brain:* Whole brains were homogenized in 10 volumes (w/v) homogenizing solution (phosphate buffer saline: protease inhibitor (100:1). An aliquot of each brain homogenate was extracted (1:3) with cold MeCN containing 200 nM of an appropriate internal standard. A calibration curve was prepared in naïve mouse brain homogenate over a 1 nM to 10 μM range. Three quality control samples were prepared by spiking the parent compound in naïve mouse brain homogenate to 20, 200, and 2000 nM as final concentrations. The calibrators and quality control samples were extracted (1:3) with the same extraction solution as the brain homogenates. The brain homogenates, calibrators, and quality control samples were centrifuged at 3270 *g* for 20 min at 4 °C.

*Quantification:* The supernatants of the extracted plasma samples, brain homogenates, and respective calibrators and quality controls were further diluted (1:1) with H_2_O, and analyzed by LC-MS/MS on a Waters ACQUITY UPLC-MS/MS system as defined above. For ARN25068 (**1**), the analyses were run on an ACQUITY UPLC BEH C_18_ (50 × 2.1 mm ID, particle size 1.7 μm) with a VanGuard BEH C_18_ precolumn (5 × 2.1 mm ID, particle size 1.7 μm) at 40 °C, using H_2_O + 0.1% HCOOH (A) and MeCN + 0.1% HCOOH (B) as mobile phase. For **28** (ARN25699), the analyses were run on a ACE Excel 2 C_18_ (150 × 2.1 mm ID) with a ACE Excel UHPLC Precolumn Filter at 40 °C, using 5 mM NH_4_HCO_3_ in H_2_O (A) and MeCN (B) as mobile phase. All samples were quantified by MRM peak area response factor in order to determine the levels of the parent compound in both plasma and brain. The plasma concentrations versus time were plotted, and the profiles were fitted using PK Solutions Excel Application (Summit Research Service, USA) in order to determine the pharmacokinetic parameters.

#### X-ray Studies, Protein Production, Crystallization, and X-ray Crystal Structure Determination

##### GSK-3β

Human full-length GSK3β sequence (1–420) was overexpressed in High 5 insect cells as previously described.^69^ For purification, a pellet of 900 × 10^6^ cells was thawed and resuspended in lysis buffer (20 mM Tris pH 8.0, 0.5 M NaCl, 10 mM Imidazole, 1 mM DTT, 5 mM MgCl2, 0.5× protease inhibitor EDTA free (*Roche*)*,* 5% glycerol and, 0.01% Tween20) and lysed by sonication (12′ pulse at 60–70% intensity). After sonication, the lysate solution was incubated for 20 min at 4 °C with DNase I (5 μg/mL final working concentration) and centrifuged for 1 h at 30,000 *g* at 4 °C. The supernatant solution, containing the protein of interest, was used for further purification. First, the clarified supernatant was incubated for 2 h with Ni-NTA agarose resin (Qiagen). The protein-bound resin was washed with binding buffer (20 mM TRIS pH 8.0, 0.5 M NaCl, 10 mM Imidazole, 5% glycerol, and 1 mM DTT). Protein elution was obtained by the addition of 300 mM Imidazole to the binding buffer. The eluted protein solution was further purified using a cationic exchange column *HiTrap HP SP,* equilibrated with buffer A (20 mM Hepes, pH 7.5, 40 mM NaCl, 5% glycerol, 1 mM DTT). Different isoforms, corresponding to different GSK-3β phosphorylation states, were separated by applying a linear gradient to Buffer B (20 mM Hepes pH 7.5, 1 M NaCl, 5% glycerol, 1 mM DTT). Only the first peak eluting from the HiTrap HP SP column at 100–130 mM NaCl, corresponding to the phosphorylated (pTyr216) and most active isoform of GSK-3β, was used for subsequent experiments. Protein aliquots were collected and stored at −80 °C.

##### DYRK1A

For DYRK1A, a pET28a vector, inserted with codon-optimized cDNA sequence (coding for DYRK1A kinase domain residue 127–485) between NcoI/XhoI sites was used. The expressed DYRK1A Kinase domain contained a N-terminal Hexa-His tag separated from the protein by a 3C protease cleavage tag. *E. coli* cells (BL21DE3) transfected with this vector were used to overexpress the protein. Briefly, an overnight culture was used to inoculate 1 L Luria–Bertani (LB) medium supplemented with 50 μM kanamycin. Protein overexpression was induced by the addition of 0.5 mM IPTG when OD_600_ reached 0.6. After overnight bacterial growth at 25 °C, cells were pelleted, and lysis was performed in 50 mM potassium phosphate, pH 7.4, 500 mM NaCl, 1 mM DTT, 5% glycerol, 0.5× protease inhibitor (EDTA-free) using sonication. The first purification step was a His-trap affinity chromatography using the equilibration buffer: 50 mM potassium phosphate, pH 7.4, 500 mM NaCl, 1 mM DTT, 5% glycerol, and 5 mM imidazole. After an initial wash with 25 mM imidazole buffer, elution was performed with 200 mM imidazole. His-tag was removed by overnight incubation of the purified protein with 3C protease at 4 °C. His-trap affinity chromatography was then run to remove the uncleaved protein and the His-Tag. The final purification step was performed using a Superdex 200 Increase column (GE Healthcare) with the following running buffer: 50 mM MES (pH 6.5), 100 mM KCl, 1 mM DTT, and 5% glycerol. The purified protein was finally stored at −80 °C.

##### Protein Crystallization

GSK-3β protein was concentrated to 4 mg/mL in the buffer 20 mM Hepes, pH 7.5, 150 mM NaCl, and 1 mM DTT. A compound solution was prepared (0.5–2 mM final concentration) in the buffer 20 mM Hepes, pH 7.5, and 150 mM NaCl. This buffer was mixed with protein solution to get a final protein concentration of 2 mg/mL and a compound concentration of 0.25 to 1 mM. Co-crystallization was performed using the hanging drop vapor diffusion method. Drops were prepared by mixing 1 μL of protein solution with 1 μL of reservoir solution (20 mM Hepes pH 7.5, 15–20% Polyethylene glycol 3350, and 100 mM NaCl). The same protocol was used for all the compounds and resulted in crystals the next day. Crystals were allowed to grow for one week at room temperature. Crystals were soaked in the reservoir buffer supplemented with 20% glycerol for cryoprotection before being frozen by plunging directly into liquid nitrogen.

DYRK1A crystallization was also performed by the hanging drop method. The final protein concentration was 4–5 mg/mL in 50 mM Mes pH 6.5, 100 mM KCl, 1 mM DTT, 5% glycerol. Reservoir buffer was 50 mM Mes pH 6.5, 150 mM KCl, and 15–20% polyethylene glycol 1000. The protein solution was mixed with the reservoir buffer in a 1:1 ratio and equilibrated against the reservoir buffer. Crystals appeared in one day and grew for a week at RT. Crystals were soaked for 5–6 h in 50 mM Mes pH 6.5, 150 mM KCl, 20% PEG 1000 buffer, and 1–2 mM compound concentrations. The same soaking protocol was used for all the compounds. Crystals were frozen in liquid nitrogen after scooping directly from the soaking solution.

##### X-ray Diffraction Data Collection and Crystal Structure Solutions

X-ray diffraction data were collected at the XRD2 beamline of Elettra Synchrotron, Trieste, Italy. For all the crystals, a total of 720 diffraction images were collected, each corresponding to a 0.5-degree rotation, finally covering the entire 360-degree reciprocal space. Data integration was performed using XDS.^70^ Data scaling is performed using AIMLESS.^71^ Structures were solved by molecular replacement using PHASER.^72^ For molecular replacement, the following available structures were used: PDB ID 6H0U (for GSK-3β) and 3ANQ (for DYRK1A). Structures were refined using PHENIX.^73^ Model modification, visualization, and evaluation were performed using Coot.^74^ Images were prepared using Pymol.^75^

#### Computational Modeling and Compound Docking

Multiple relevant published structures of each of the human proteins GSK-3β, DYRK1A, and FYN were obtained from the Protein Data Bank.^76^ Computational modeling, docking, and visualizations were performed using MolSoft ICM-Pro software, version 3.9-4a.^77,78^ Molsoft LLC. Available online: https://www.molsoft.com/ (accessed on 1 April 2025).

## Supplementary Material











## Notes

Selectivity studies (Tables S1 and S2); Docking poses representation of compounds **1**-**9**, **29**, **30**, **31** (ARN26646) in the GSK-3β/FYN and DYRK1A binding pockets (Figures S1–S3, and S6); X-ray of **16**, **20**, **25**, **27** and **28** (ARN25699) in complex with GSK-3β and DYRK1A (Figures S4, S7, and S8); X-ray low resolution poses of **28** (ARN25699) in complex with DYRK1A (Figure S5); X-ray crystallographic data processing and analysis (Tables S3 and S4); MetID experiments in MLMs (Figure S9); ^1^H NMR and ^13^C NMR spectra of all tested compounds and intermediates; UPLC-MS purity chromatograms of all tested compounds (PDF).
Molecular formula strings (CSV)
3D PDB docking compound 1 (PDB)
3D PDB docking compound 16 (PDB)
3D PDB docking compound 28 (PDB)

## ●

Structural Biophysics Facility, Istituto Italiano di Tecnologia, 16163 Genova, Italy

## ◊

Department of Chemical and Systems Biology, Stanford University, Stanford, California 94305, USA

## ▲

Department of Pharmaceutical Sciences, Università degli Studi del Piemonte Orientale, Largo Donegani 2, 28100 Novara, Italy

## □

CECAM, EPFL, Avenue Forel 3, CH-1015 Lausanne, Switzerland

## Abbreviations

Aβ: amyloid-beta
AD: Alzheimer’s disease
ADME: absorption, distribution, metabolism and excretion
BBB: blood-brain barrier
CL: clearance
CNS: central nervous system
DS: Down Syndrome
DYRK1A: dual-specificity tyrosine-regulated kinase 1A
ER: endoplasmic reticulum
F: fraction absorbed (bioavailability)
FDA: Food and Drug Administration
FYN: FYN proto-oncogene, Src family tyrosine kinase
GSK-3β: glycogen synthase kinase-3β
HB: hydrogen bond
HLM: human liver microsomes
HTRF: homogeneous time-resolved fluorescence
I.V.: intravenous
MLM: mouse liver microsomes
MT: microtubule
MTDL: multitarget directed ligand
NFTs: neurofibrillary tangles
PKIs: protein kinase inhibitors
PKs: protein kinases
P.O.: oral
PPs: protein phosphatases
PSA: polar surface area
SAR: structure–activity relationship
SCX: strong cation exchange
*S*_k_: kinetic solubility
SQD: single quadrupole detector
*t*_1/2_: half-life
TR-FRET: time-resolved fluorescence energy transfer
VD: volume of distribution
