# Supplementary material for: A Polypharmacology-Driven Approach to Alzheimer’s Disease and Tauopathies: Rational Design, Synthesis and Characterization of Amino-Pyrazole-Based Multikinase (GSK-3β/FYN-α/DYRK1A) Inhibitors
Source: J Med Chem. 2026 Apr 29;69(9):9991–10018. doi: 10.1021/acs.jmedchem.5c01810 (PMC13181777; doi:10.1021/acs.jmedchem.5c01810)

SUPPORTING INFORMATION

A Polypharmacology-Driven Approach to Alzheimer's Disease and Tauopathies: Rational Design, Synthesis and Characterization of Amino-Pyrazole-Based Multikinase (GSK-3β/FYN-α/DYRK1A) Inhibitors

Stefania Demuro[a,b,1], Debora Russo[c,2], Ilaria Penna[c,3], Siranuysh Grabska[d,e], Hovakim Grabski[d,e], Andrea Dalle Vedove[f,g], Aurora Valeri[h], Conall Sauvey[d], Giuliana Ottonello[i], Maria Summa[j], Sine Mandrup Bertozzi[i], Jose Ortega[a], Rosalia Bertorelli[j], Paola Storici[g], Stefania Girotto[a,2], Gabriele Cruciani[k], Rita M.C. Di Martino[a,4*], Ruben Abagyan[d^], Andrea Cavalli[a,b,5^*]

[a]Computational and Chemical Biology, Istituto Italiano di Tecnologia, via Morego 30, 16163 Genoa, Italy

[b]Department of Pharmacy and Biotechnology, University of Bologna, via Belmeloro 6, 40126 Bologna, Italy

[c]D3 PharmaChemistry, Istituto Italiano di Tecnologia, via Morego, 30, 16163 Genova, Italy.

[d]Skaggs School of Pharmacy and Pharmaceutical Sciences, University of California, San Diego, La Jolla, California 92093, United States

[e]L.A. Orbeli Institute of Physiology, National Academy of Sciences, Yerevan 0028, Armenia.

[f]AREA Science Park, Padriciano 99, 34149, Trieste, Italy

[g]Protein Targets for Drug Discovery Lab, Elettra Sincrotrone Trieste S.C.p.A., SS 14 - km 163,5 in AREA Science Park, 34149, Trieste, Italy

[h]Molecular Horizon srl, via Montelino 20, 06084 Bettona, Italy

[i]Analytical Chemistry Facility, Istituto Italiano di Tecnologia, via Morego 30, 16163 Genova, Italy

[j]Translational Pharmacology Facility, Istituto Italiano di Tecnologia, via Morego 30, 16163 Genova, Italy

[k]Department of Chemistry, Biology and Biotechnology, University of Perugia, via dell'Elce di Sotto 8, 06123 Perugia, Italy

Present addresses: [1]Department of Chemical and Systems Biology, Stanford University, Stanford, CA 94305, USA; [2]Structural Biophysics Facility, Istituto Italiano di Tecnologia, 16163, Genoa, Italy; [3]Medicinal Chemistry and Technologies for Drug Discovery and Delivery Facility, Istituto Italiano di Tecnologia, via Morego 30, 16163 Genova, Italy; [4]Department of Pharmaceutical Sciences, Università degli Studi del Piemonte Orientale, Largo Donegani 2, 28100 Novara, Italy; [5]CECAM, EPFL, Avenue Forel 3, CH - 1015 Lausanne, Switzerland.

^Equal contribution as senior author

*Correspondence should be addressed to:

Dr. Rita Maria Concetta Di Martino, email: rita.dimartino@uniupo.it (https://orcid.org/0000-0003-2287-3331)

Prof. Andrea Cavalli, email: andrea.cavalli@iit.it (https://orcid.org/0000-0002-6370-1176)

Table of Contents

1. Selectivity studies..S4

2. Docking and X-ray figures of selected compounds..S7

3. Details of crystallographic data processing and analysis……….............…...……..…......S11

4. MetaID studies in MLMs...………..............…...….S14

5. ^{1}H and ^{13}C NMR spectra.................…………………………………..……………………..… S16

6. UPLC- and UV-MS chromatograms ………......................………………….…………….…..…S53

Selectivity studies

Table S1. Percentage (%) of inhibition of compound **28** at 0.1 and 10 µM against a panel of 100 different PKs.

Kinase	Kinase Inhibition % Cpd 28 (ARN25699)	
	0.1 µM	10 µM
Abl(h)	53	100
ALK1(h)	81	100
AMPKα1(h)	85	100
AMPKα2(h)	92	99
ASK1(h)	4	13
ATM(h)	12	30
ATR/ATRIP(h)	0	4
Axl(h)	10	96
BMPR2(h)	37	99
BMX(h)	71	101
BTK(h)	45	100
c-Raf(h)	57	100
CaMK2α(h)	49	98
CDK1/cyclinB(h)	57	98
CDK2/cyclinA(h)	79	100
CDK4/cyclinD3(h)	30	99
CDK5/p25(h)	88	98
CDK7/cyclinH/MAT1(h)	58	99
CK1δ(h)	0	70
CK1ε(h)	0	71
CK2(h)	0	85
cKit(h)	9	98
CLK1(h)	99	101
CLK2(h)	100	100
CRIK(h)	4	12
cSRC(h)	89	99
DAPK1(h)	0	30
DAPK2(h)	0	47
DDR1(h)	90	100
DYRK1A(h)	32	98
DYRK1B(h)	37	95

**DYRK2(h)**	2	87
**DYRK3(h)**	15	94
**EGFR(h)**	0	87
**EphA5(h)**	35	98
**ErbB2(h)**	0	69
**FAK(h)**	41	99
**FGFR1(h)**	99	99
**FGFR2(h)**	88	98
**Flt3(h)**	97	98
**Fyn(h)**	100	99
**GSK3α(h)**	69	100
**GSK3β(h)**	52	95
**HIPK1(h)**	7	70
**HIPK4(h)**	18	93
**Itk(h)**	89	92
**JNK1α1(h)**	10	49
**LATS1(h)**	28	82
**LATS2(h)**	35	94
**Lck(h) activated**	86	100
**LKB1(h)**	12	96
**LRRK2(h)**	64	91
**MAP4K4(h)**	28	98
**MAPK1(h)**	4	91
**MARK3(h)**	80	102
**MEK1(h)**	45	99
**MEK2(h)**	23	99
**MELK(h)**	85	99
**Met(h)**	10	86
**MINK(h)**	26	95
**MKK3(h)**	0	30
**MLK2(h)**	45	97
**MSK1(h)**	11	88
**MST1(h)**	96	99
**mTOR/FKBP12(h)**	4	16
**NLK(h)**	11	89
**PAK1(h)**	74	98
**PAK2(h)**	45	96
**PKA(h)**	17	96
**PKCα(h)**	0	70

**PRK1(h)**	65	102
**PRK2(h)**	18	93
**PRP4(h)**	8	17
**Pyk2(h)**	41	99
**Ret(h)**	96	99
**RIPK1(h)**	0	48
**RIPK2(h)**	20	83
**ROCK1(h)**	2	29
**ROCK2(h)**	0	39
**Rsk1(h)**	72	99
**Rsk2(h)**	65	98
**SAPK2a(h)**	0	7
**SGK(h)**	1	12
**SIK(h)**	63	98
**SLK(h)**	100	100
**SNRK(h)**	6	84
**SRPK1(h)**	16	96
**SRPK2(h)**	23	97
**Syk(h)**	67	97
**TAK1(h)**	37	93
**TAO1(h)**	14	93
**TAO2(h)**	5	69
**Tec(h) activated**	63	98
**TGFBR1(h)**	26	97
**TGFBR2(h)**	13	89
**TNIK(h)**	91	98
**TTBK1(h)**	0	0
**TTBK2(h)**	2	4
**VRK1(h)**	4	36
**VRK2(h)**	0	69
**Yes(h)**	96	100

Table S2. Head-to-head comparison of compounds **1** and **28** showing the percentage (%) of inhibition at 0.1 and 10 μM against a panel of 20 selected PKs.

Kinase	Kinase Inhibition %			
	Cpd 1 (ARN25068)		Cpd 28 (ARN25699)	
	0.1 μM	10 μM	0.1 μM	10 μM
DYRK1A(h)	14	92	32	98
CDK5/p25(h)	86	97	88	98
Fyn(h)	100	100	100	99
GSK3β(h)	94	100	52	95
GSK3α(h)	96	100	69	100
SAPK2a(h)	13	27	0	7
MAPK1(h)	19	67	4	91
MAP4K4(h)	14	99	28	98
JNK1α1(h)	11	80	10	49
PKA(h)	9	88	17	96
cSRC(h)	96	96	89	99
CDK1/cyclinB(h)	62	99	57	98
Yes(h)	98	100	96	100
Lck(h) activated	58	97	86	100
CK1δ(h)	6	73	0	70
DYRK1B(h)	10	88	37	95
CK1ε(h)	0	83	0	71
CK2(h)	5	75	0	85
DYRK3(h)	19	90	15	94
DYRK2(h)	16	77	2	87

Docking and X-ray figures of key compounds

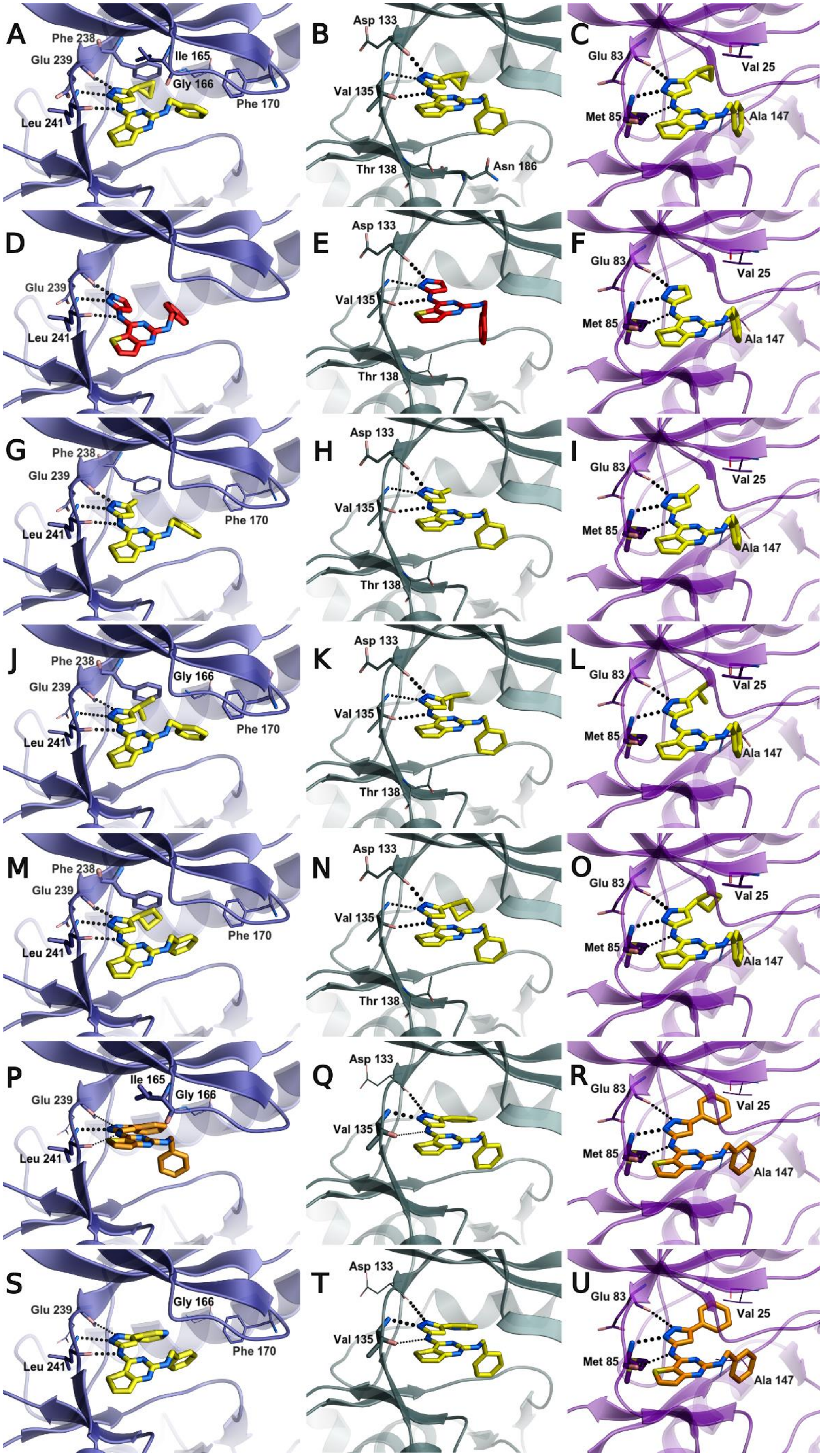

A
Phe 238
Glu 239
Ile 165
Gly 166
Phe 170
Leu 241
B
Asp 133
Val 135
Thr 138
Asn 186
C
Glu 83
Val 25
Met 85
Ala 147
D
Glu 239
Leu 241
E
Asp 133
Val 135
Thr 138
F
Glu 83
Val 25
Met 85
Ala 147
G
Phe 238
Glu 239
Phe 170
Leu 241
H
Asp 133
Val 135
Thr 138
I
Glu 83
Val 25
Met 85
Ala 147
J
Phe 238
Glu 239
Gly 166
Phe 170
Leu 241
K
Asp 133
Val 135
Thr 138
L
Glu 83
Val 25
Met 85
Ala 147
M
Phe 238
Glu 239
Phe 170
Leu 241
N
Asp 133
Val 135
Thr 138
O
Glu 83
Val 25
Met 85
Ala 147
P
Ile 165
Glu 239
Gly 166
Leu 241
Q
Asp 133
Val 135
R
Glu 83
Val 25
Met 85
Ala 147
S
Glu 239
Gly 166
Phe 170
Leu 241
T
Asp 133
Val 135
U
Glu 83
Val 25
Met 85
Ala 147

Figure S1. Predicted docking poses of the tested series I compounds: DYRK1A with compound **1** (A), **2** (D), **3** (G), **4** (J), **5** (M), **6** (P), **7** (S) (**2**-red, **6**-light orange, rest-yellow); GSK-3β with **1** (B), **2** (E), **3** (H), **4** (K), **5** (N), **6** (Q), **7** (T) (**2**-red, rest-yellow); FYN with **1** (C), **2** (F), **3** (I), **4** (L), **5** (O), **6** (R), **7** (U) (**6**-light orange, **7**-dark orange, rest-yellow). Hydrogen bonds are shown as dotted lines.

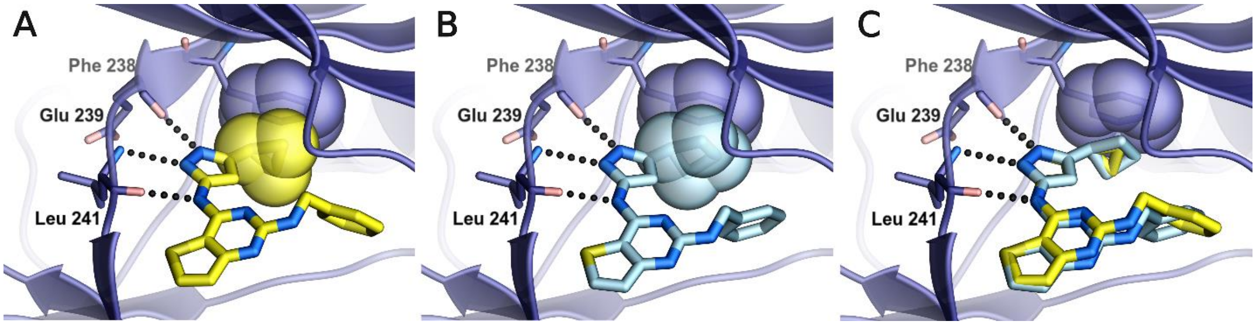

Figure S2. The docking poses of compounds **1** and **5** at the ATP-binding pocket of DYRK1A. Panel A: DYRK1A bound to **1**; Panel B: DYRK1A bound to **5**; Panel C: superimposition of compounds **1** (yellow) and **5** (skyblue).

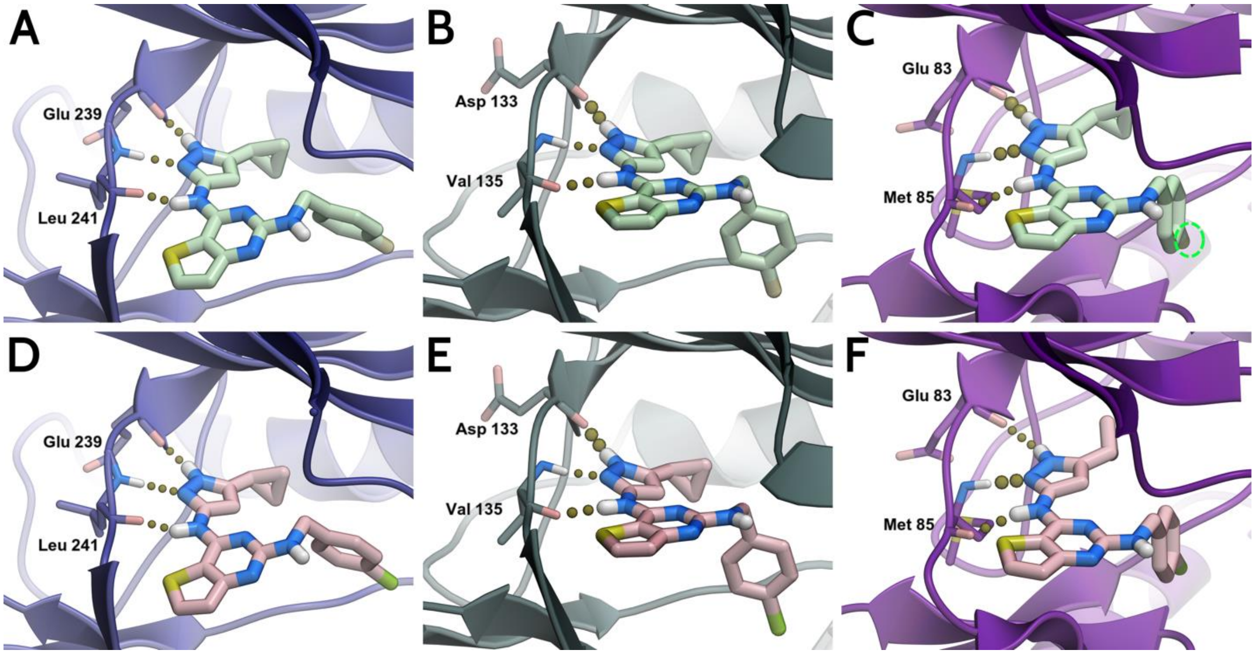

Figure S3. Docking poses of compound **8** in the binding pockets of DYRK1A (A), GSK-3β (B), and FYN (C). Docking poses of compound **9** docked into the binding pockets of DYRK1A (D), GSK-3β (E), and FYN (F) are also shown. The green dotted circle indicates the most favorable interaction with the pocket.

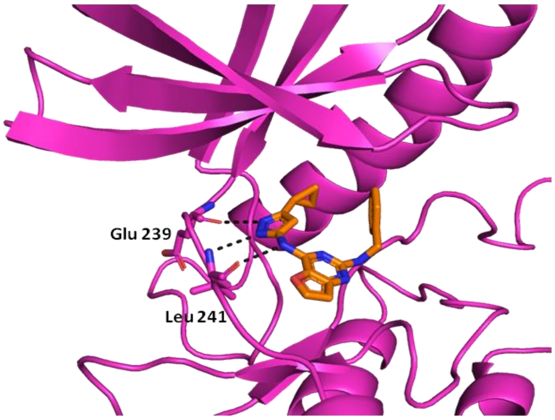

Figure S4. X-ray structure of compound **20** in complex with DYRK1A (PDB ID: 9FT4).

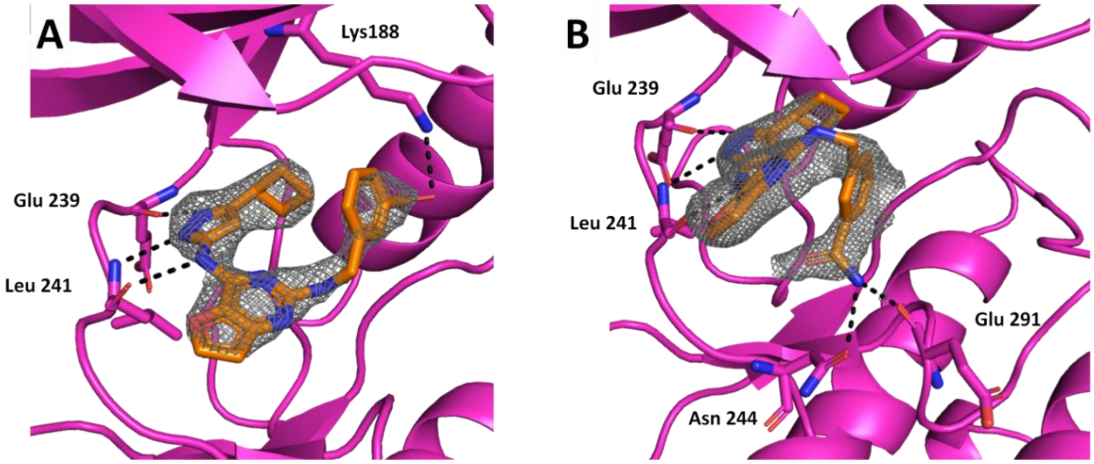

Figure S5. Additional poses, discarded due to poor refinement statistics, suggest a direct interaction of the *m*-carbamoyl moiety of **28** (ARN25699) with either Lys188 side-chain (A) or with both the side chain of Asn244 and the main chain of Glu291 (B).

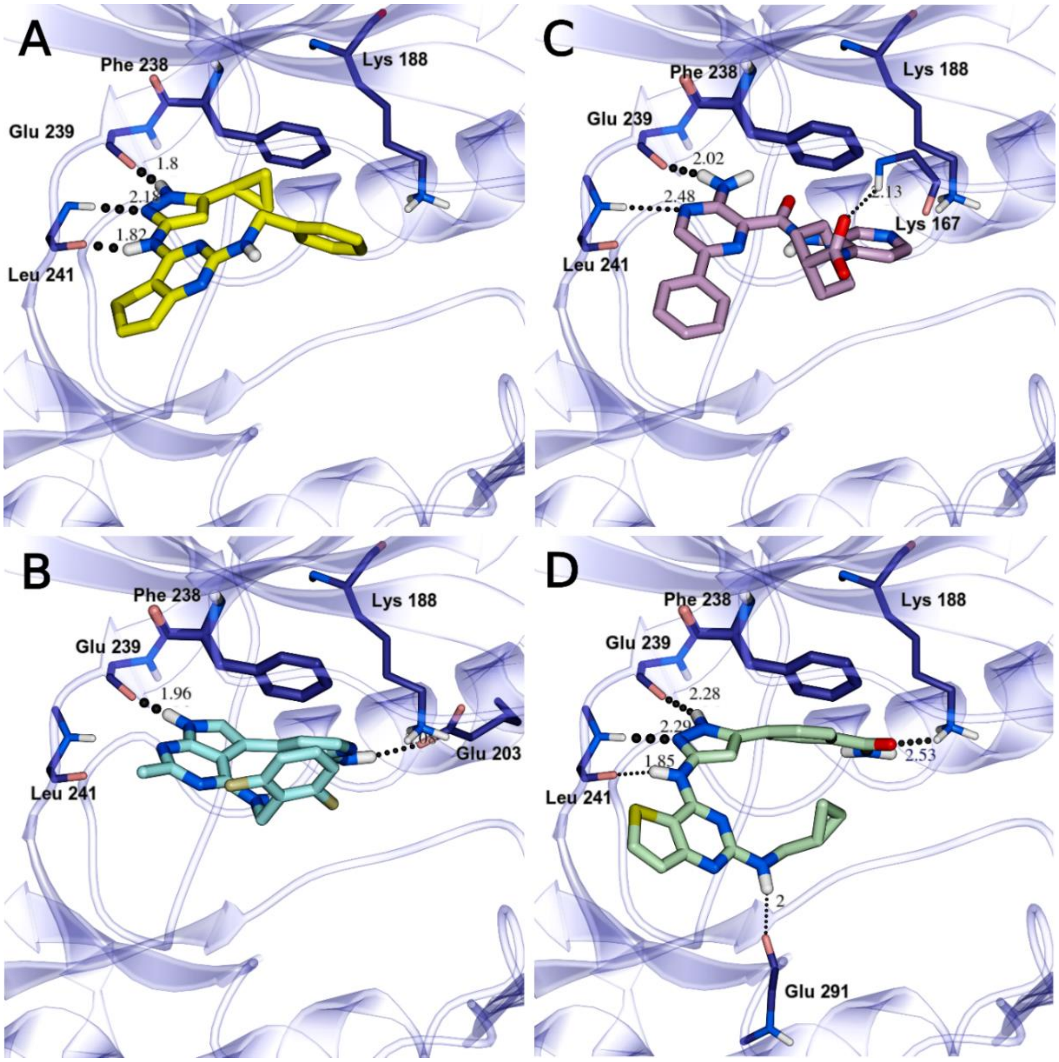

Figure S6. Predicted binding poses of compounds **1** (A), **29** (B), **30** (C), and **31** (ARN26646, D) in the DYRK1A binding pocket.

Details of crystallographic data processing and analysis

Table S3. Data Collection and Refinement Statistics for GSK-3β structures in complex with inhibitors.

	**27** (ARN25697)	**16** (ARN25641)	**20** (ARN25507)	**25** (ARN25565)	**28** (ARN25699)
**PDB entry**	9FR5	9FR6	9FR7	9FR8	9FR9
Data Collection					
**Beamline**	Elettra XRD2	Elettra XRD2	Elettra XRD2	Elettra XRD2	Elettra XRD2
**Space group**	$P2_12_12_1$	$P2_12_12_1$	$P2_12_12_1$	$P2_12_12_1$	$P2_12_12_1$
**Unit cell parameters (Å; °)**	A = 82.37<br>B = 85.35<br>C = 178.64<br>α = β = γ = 90	A = 82.31<br>B = 85.26<br>C = 178.44<br>α = β = γ = 90	A = 82.32<br>B = 85.25<br>C = 178.42<br>α = β = γ = 90	A = 82.57<br>B = 85.44<br>C = 178.35<br>α = β = γ = 90	A = 82.46<br>B = 84.62<br>C = 178.45<br>α = β = γ = 90

**R_{merge}**	0.02 (0.26)	0.03 (0.23)	0.03 (0.29)	0.03 (0.33)	0.03 (0.26)
**R_{meas}**	0.03 (0.37)	0.04 (0.33)	0.04 (0.41)	0.05 (0.46)	0.04 (0.36)
**R_{pim}**	0.02 (0.26)	0.03 (0.23)	0.03 (0.29)	0.03 (0.33)	0.03 (0.26)
**<I/σ(I)>**	25.57 (5.51)	19.41 (5.03)	24.77 (3.63)	19.65 (3.32)	22.89 (4.95)
**CC1/2**	0.999 (0.787)	0.999 (0.814)	0.999 (0.775)	0.998 (0.726)	0.998 (0.752)
**Completeness (%)**	99.76 (98.05)	96.81 (81.94)	97.58 (83.51)	94.90 (69.18)	97.15 (80.44)
Refinement					
**Resolution (Å)**	60.55 -2.30 (2.38 -2.30)	61.64-2.30 (2.38-2.30)	49.34-2.30 (2.38-2.30)	74.93-2.30 (2.38-2.30)	56.07-2.55 (2.64-2.55)
**R_{work}/R_{free} (%)**	20.3/23.5	19.8/23.2	19.8/22.8	19.1/22.2	19.4/23.3
r.m.s. deviations					
**Bond lengths (Å)**	0.003	0.004	0.002	0.08	0.09
**Bond angles (°)**	0.62	0.65	0.51	0.92	0.99

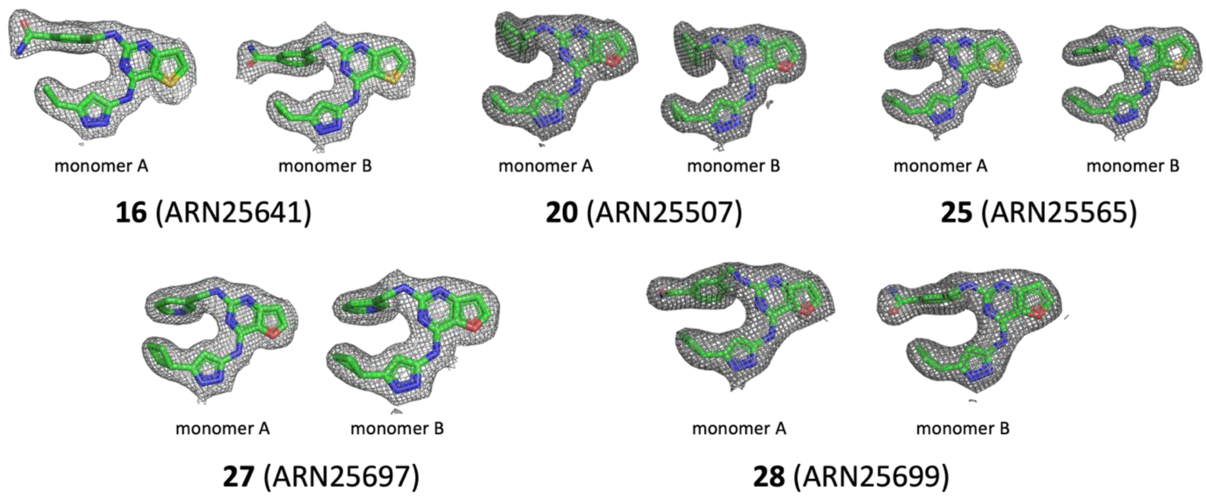

Figure S7. Electron density of selected compounds in complex with GSK-3β.

Table S4. Data Collection and Refinement Statistics for DYRK1A structures in complex with inhibitors.

	**27** (ARN25697)	**16** (ARN25641)	**20** (ARN25507)	**25** (ARN25565)	**28** (ARN25699)
**PDB entry**	9FT6	9FUF	9FT4	9FT3	9FT2
Data Collection					
**Beamline**	Elettra XRD2	Elettra XRD2	Elettra XRD2	Elettra XRD2	Elettra XRD2
**Space group**	$P4_2$	$P4_2$	$P4_2$	$P4_2$	$P4_2$
**Unit cell parameters (Å; °)**	A = 93.81<br>B = 93.81<br>C = 48.98<br>α = β = γ = 90	A = 94.08<br>B = 94.08<br>C = 49.05<br>α = β = γ = 90	A = 94.18<br>B = 94.18<br>C = 48.44<br>α = β = γ = 90	A = 94.27<br>B = 94.27<br>C = 48.70<br>α = β = γ = 90	A = 94.07<br>B = 94.07<br>C = 49.08<br>α = β = γ = 90
**R_{merge}**	0.03 (0.20)	0.02 (0.20)	0.01 (0.11)	0.02 (0.24)	0.02 (0.22)
**R_{meas}**	0.04 (0.28)	0.03 (0.29)	0.02 (0.15)	0.03(0.34)	0.04 (0.32)
**R_{pim}**	0.03 (0.20)	0.02 (0.20)	0.01 (0.11)	0.02 (0.24)	0.02 (0.22)
**<I/σ(I)>**	14.85 (3.74)	23.61 (4.20)	24.10 (5.70)	23.09 (3.49)	16.62 (2.92)

**CC1/2**	0.998 (0.892)	0.999 (0.874)	1.000 (0.972)	0.999 (0.833)	0.999 (0.886)
**Completeness (%)**	99.95 (100.0)	99.96 (100.0)	99.79 (99.53)	99.98 (100.0)	99.73 (100.0)
Refinement					
**Resolution (Å)**	46.90-3.00 (3.11-3.00)	47.04-2.70 (2.80-2.70)	43.08-3.00 (3.11-3.00)	66.66-2.70 (2.80-2.70)	43.52-2.90 (3.00-2.90)
**R_{work}/R_{free} (%)**	20.5/23.4	21.0/22.9	20.3/24.2	19.4/23.0	21.1/23.5
r.m.s. deviations					
**Bond lengths (Å)**	0.003	0.002	0.001	0.009	0.001
**Bond angles (°)**	0.51	0.48	0.43	1.01	0.41

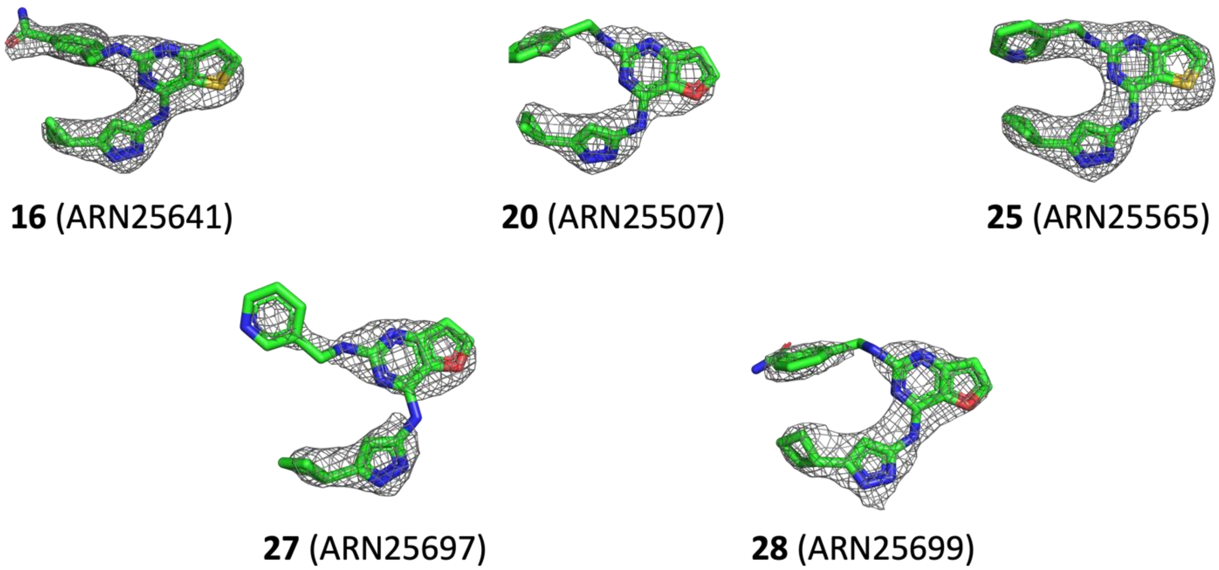

Figure S8. Electron density of selected compounds in complex with DYRK1A.

MetID studies in MLMs

A

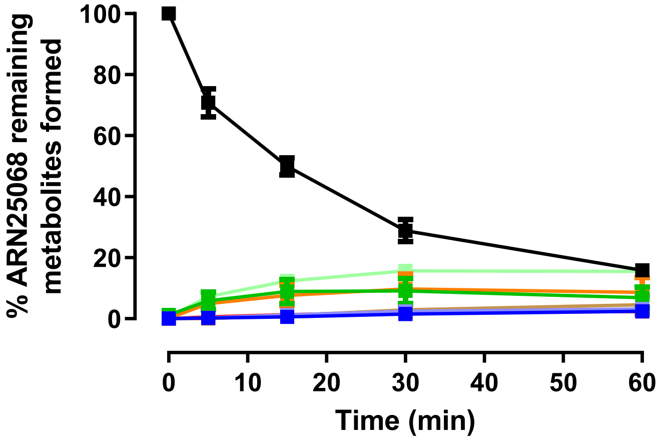

B

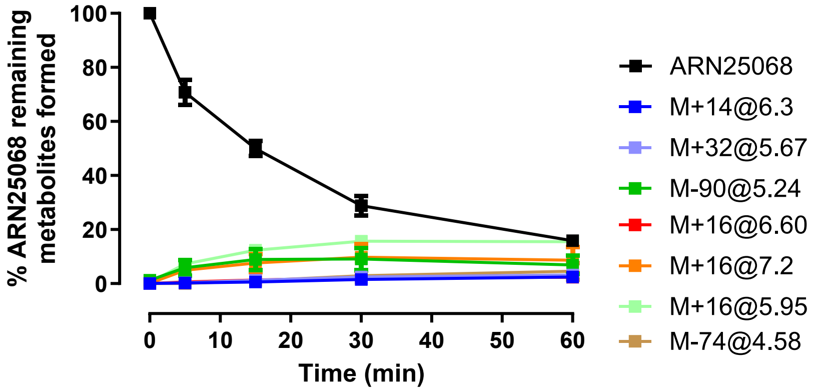

C

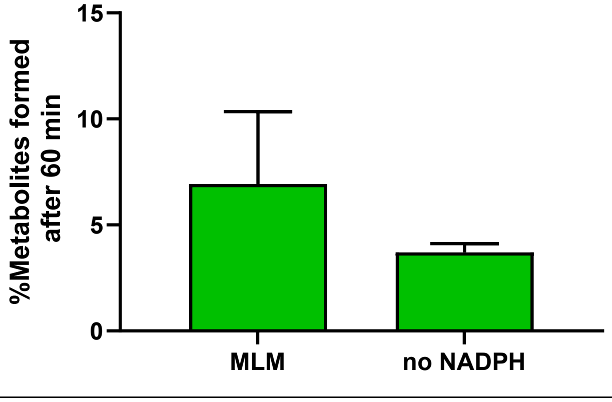

D

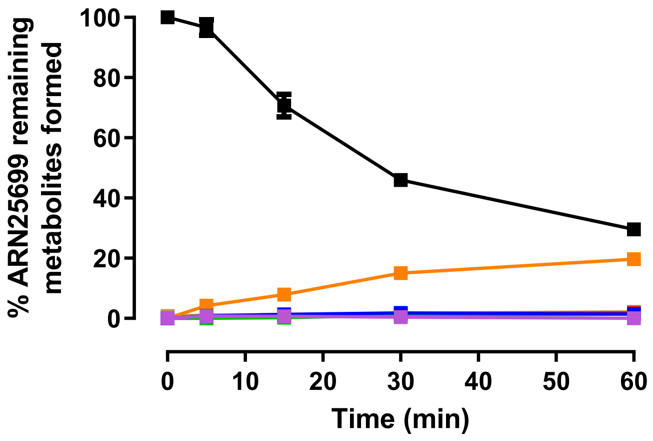

E

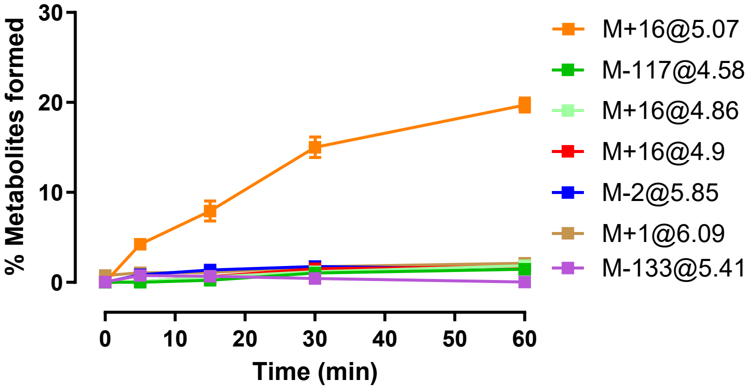

F

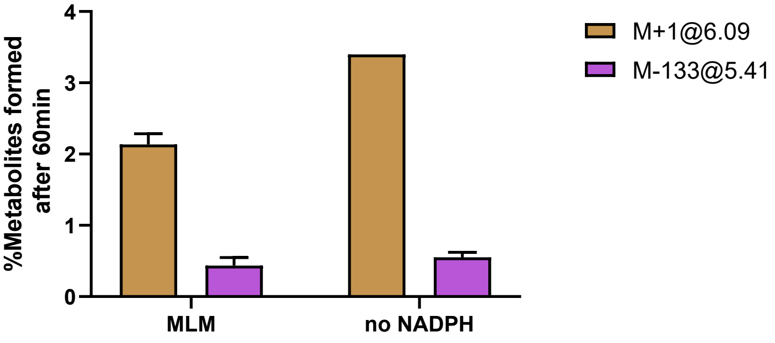

Figure S9. Metabolite profile analysis in MLMs of **1** (ARN25068, A, B, C) and **28** (ARN25699, D, E, F).

^{1}H NMR (400 MHz, DMSO-*d*$_6$) and ^{13}C NMR (101 MHz, DMSO-*d*$_6$) of compound 2

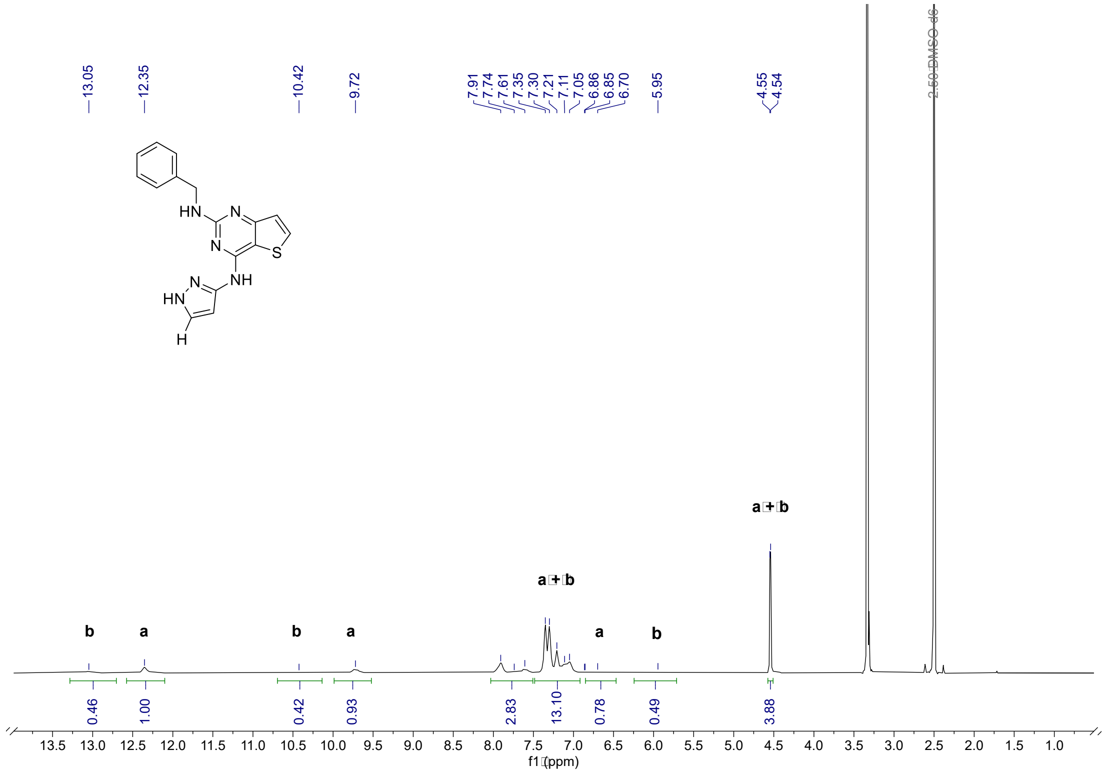

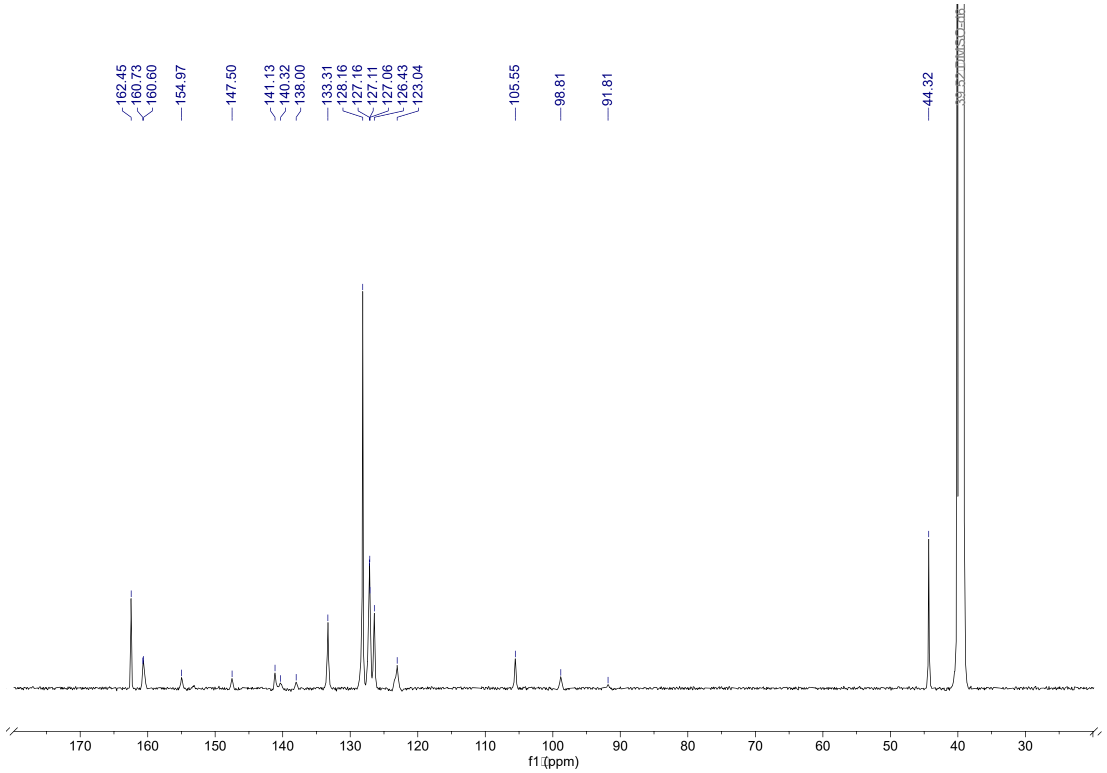

^{1}H NMR (400 MHz, DMSO-*d*$_6$) and ^{13}C NMR (101 MHz, DMSO-*d*$_6$) of compound 3

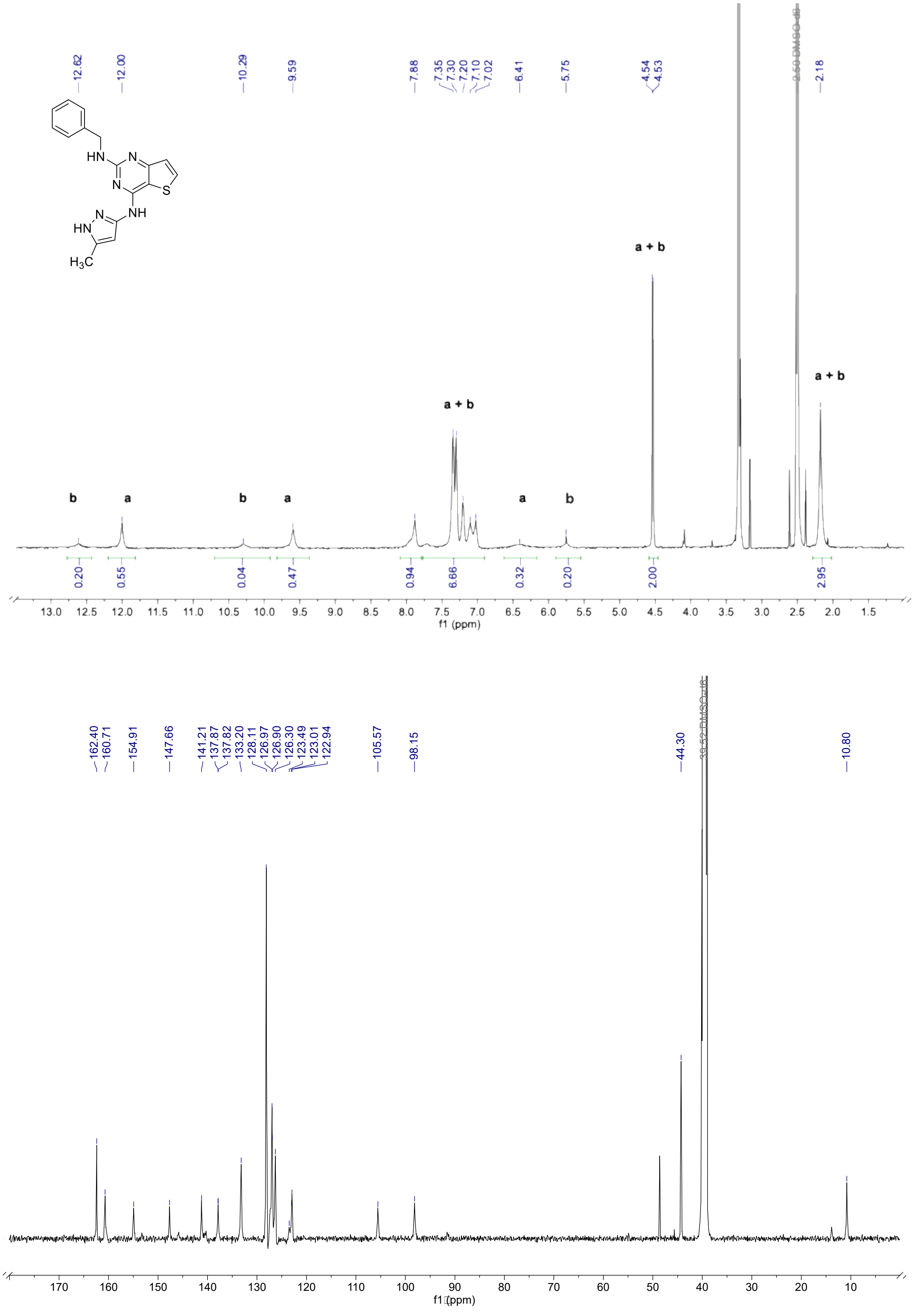

^{1}H NMR (400 MHz, DMSO-d_6) and ^{13}C NMR (101 MHz, DMSO-d_6) of compound 4

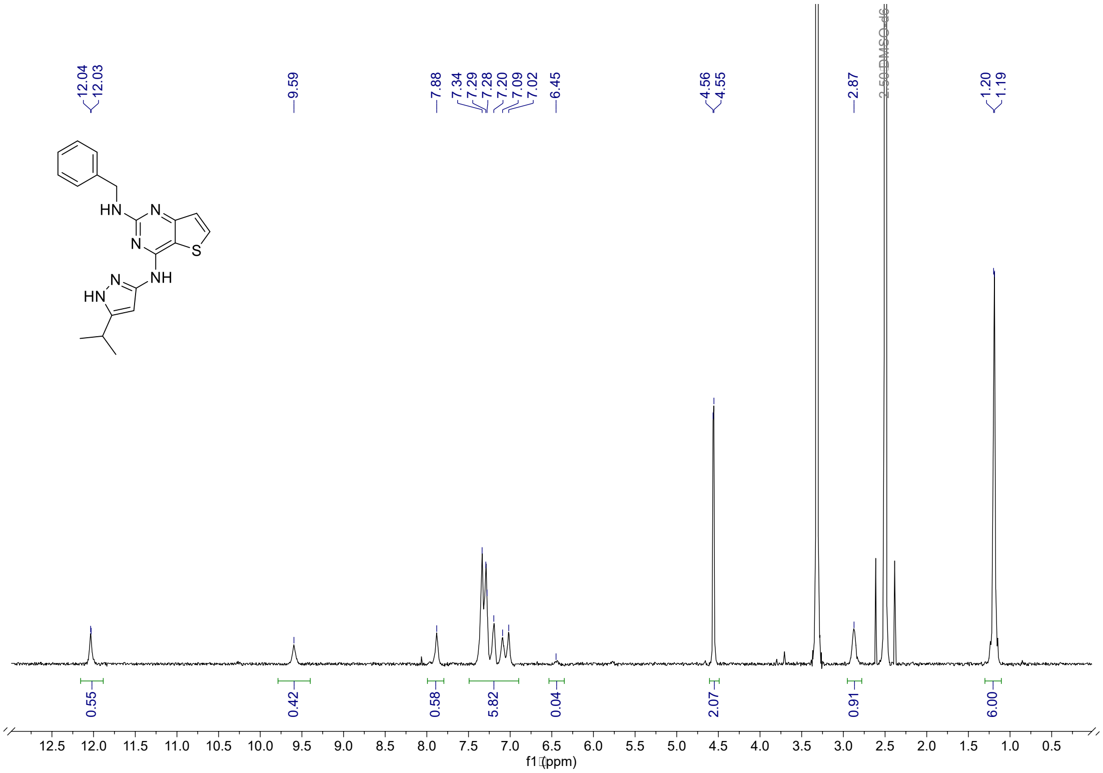
12.04
12.03
9.59
7.88
7.34
7.29
7.28
7.20
7.09
7.02
6.45
4.56
4.55
2.87
2.50 DMSO-d6
1.20
1.19
HN
N
N
S
NH
HN
N
0.55
0.42
0.58
5.82
0.04
2.07
0.91
6.00
12.5 12.0 11.5 11.0 10.5 10.0 9.5 9.0 8.5 8.0 7.5 7.0 6.5 6.0 5.5 5.0 4.5 4.0 3.5 3.0 2.5 2.0 1.5 1.0 0.5
f1 (ppm)

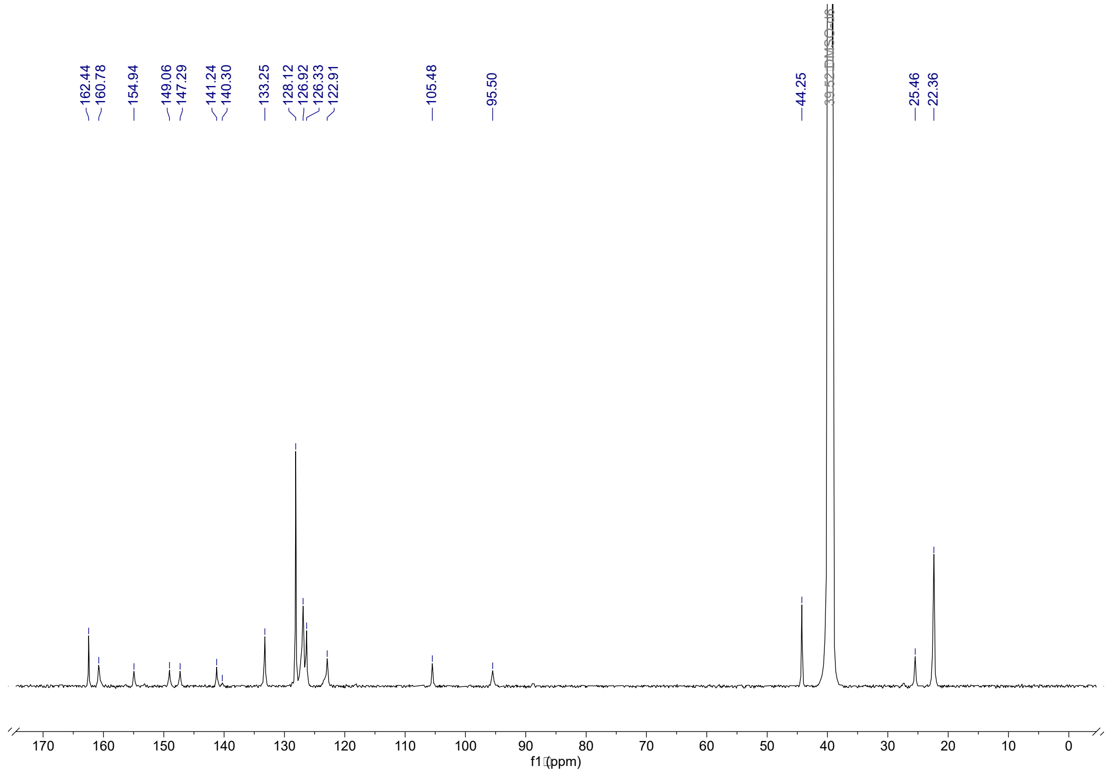
162.44
160.78
154.94
149.06
147.29
141.24
140.30
133.25
128.12
126.92
126.33
122.91
105.48
95.50
44.25
39.52 DMSO-d6
25.46
22.36
170 160 150 140 130 120 110 100 90 80 70 60 50 40 30 20 10 0
f1 (ppm)

^{1}H NMR (400 MHz, DMSO-d_6) and ^{13}C NMR (101 MHz, DMSO-d_6) of compound 5

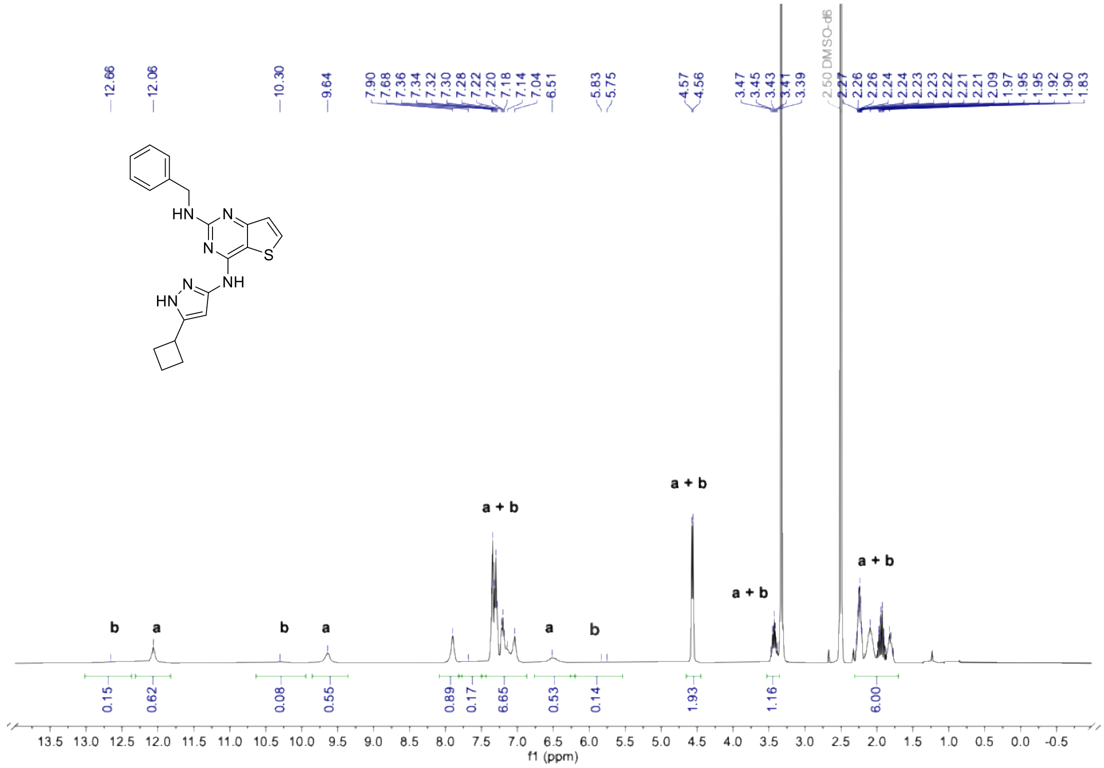

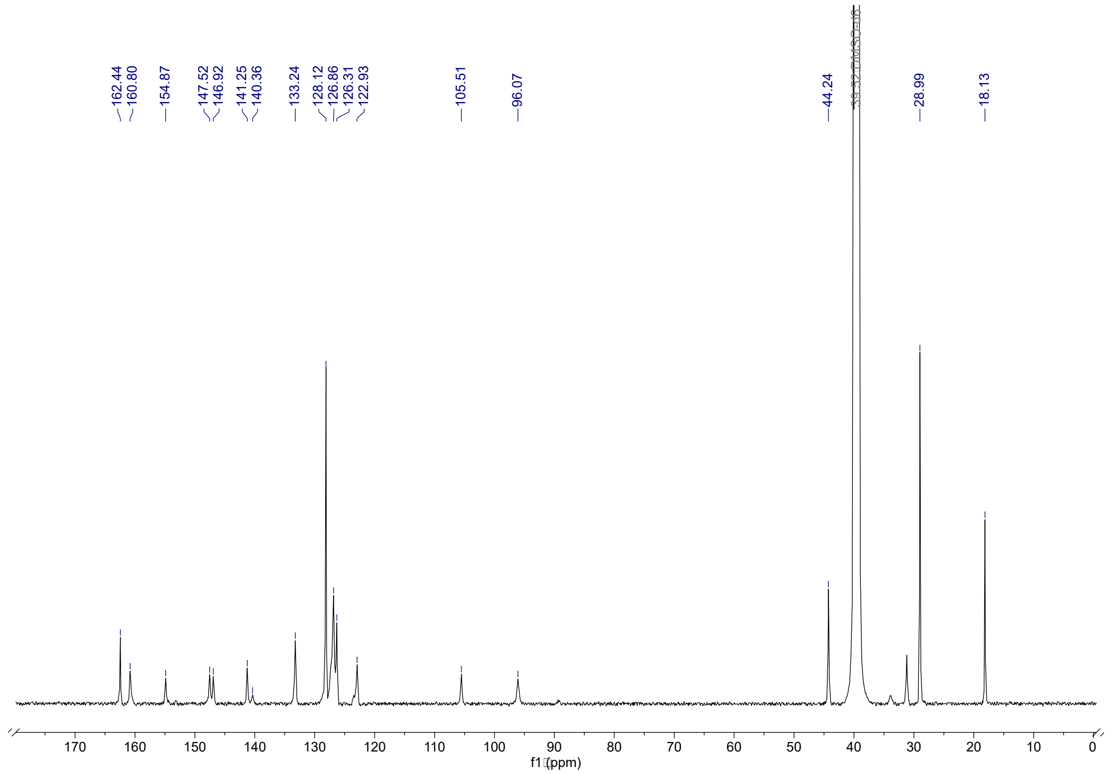

^{1}H NMR (400 MHz, DMSO-d_6) and ^{13}C NMR (101 MHz, DMSO-d_6) of compound 6

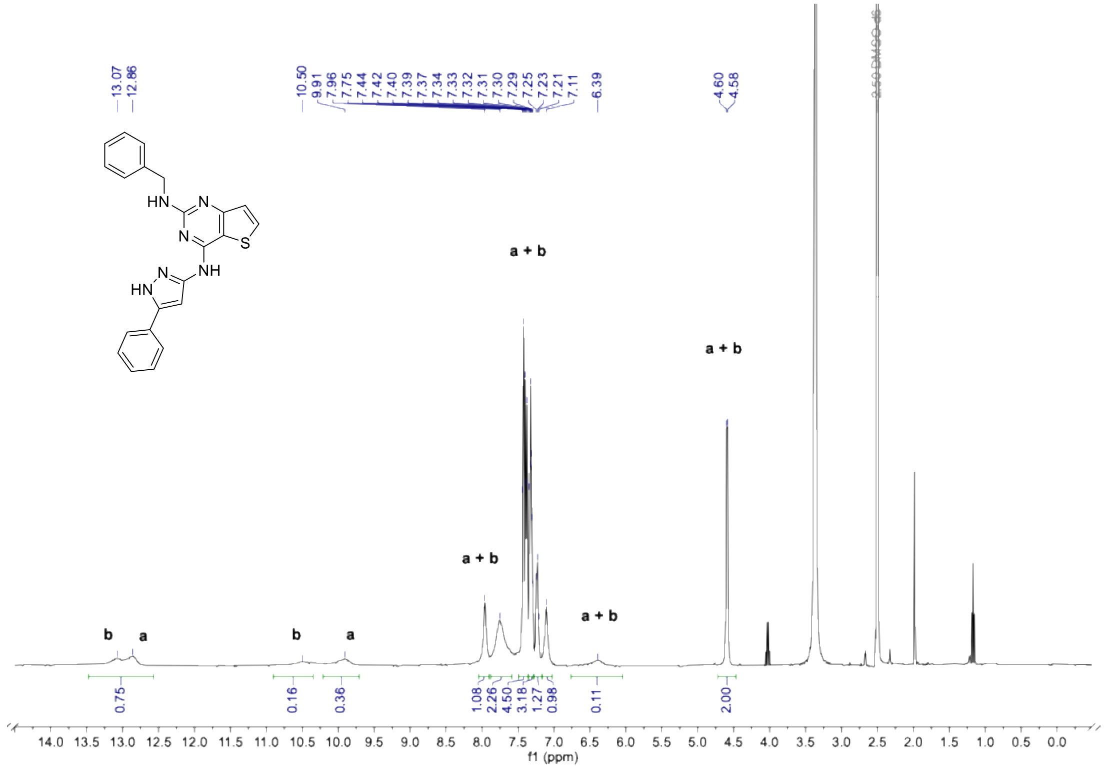

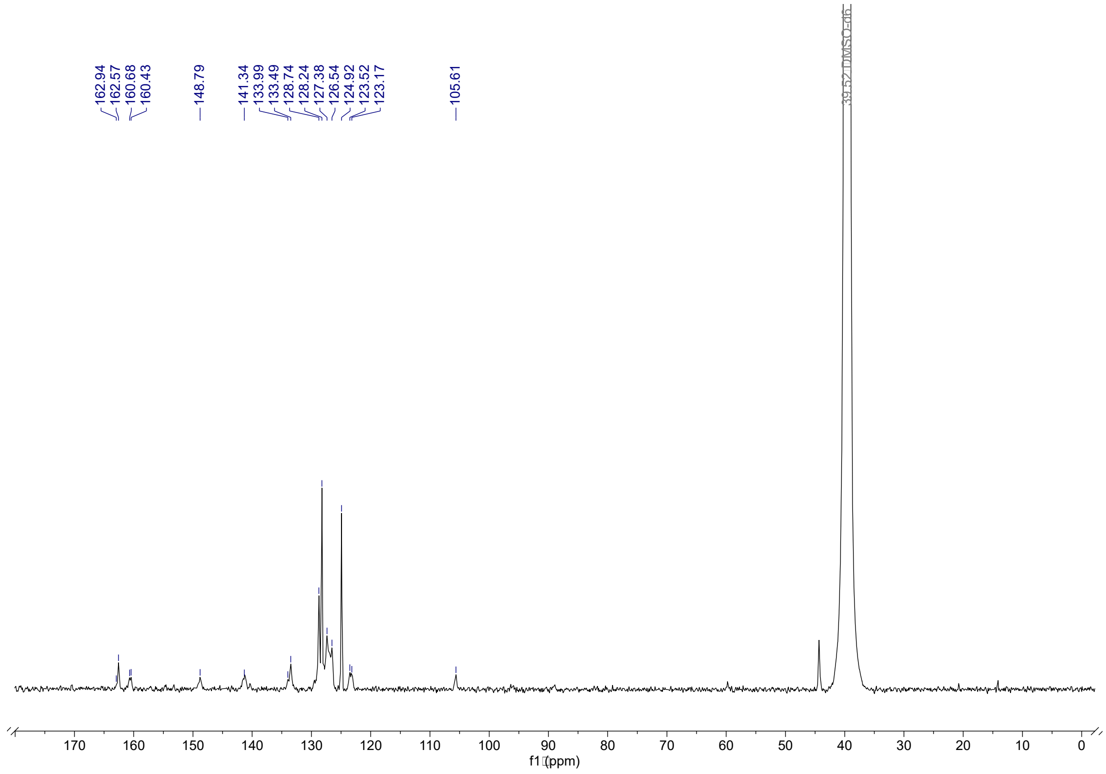

^{1}H NMR (400 MHz, DMSO-d_6) and ^{13}C NMR (101 MHz, DMSO-d_6) of compound 7

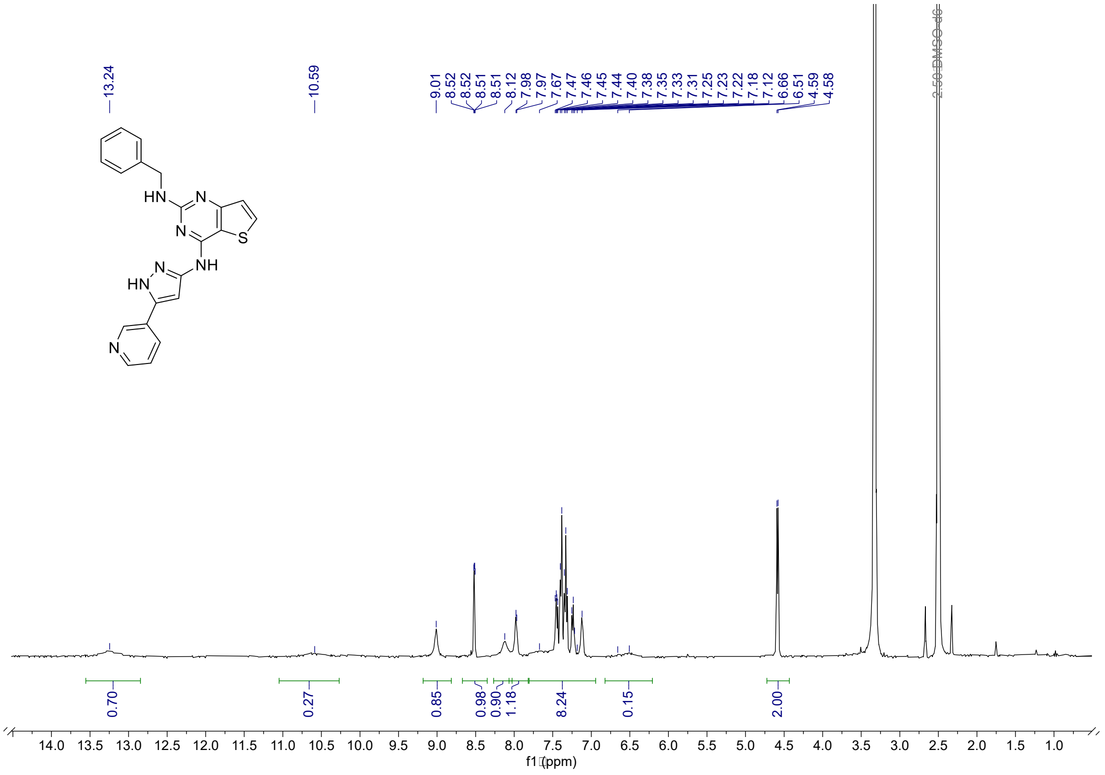

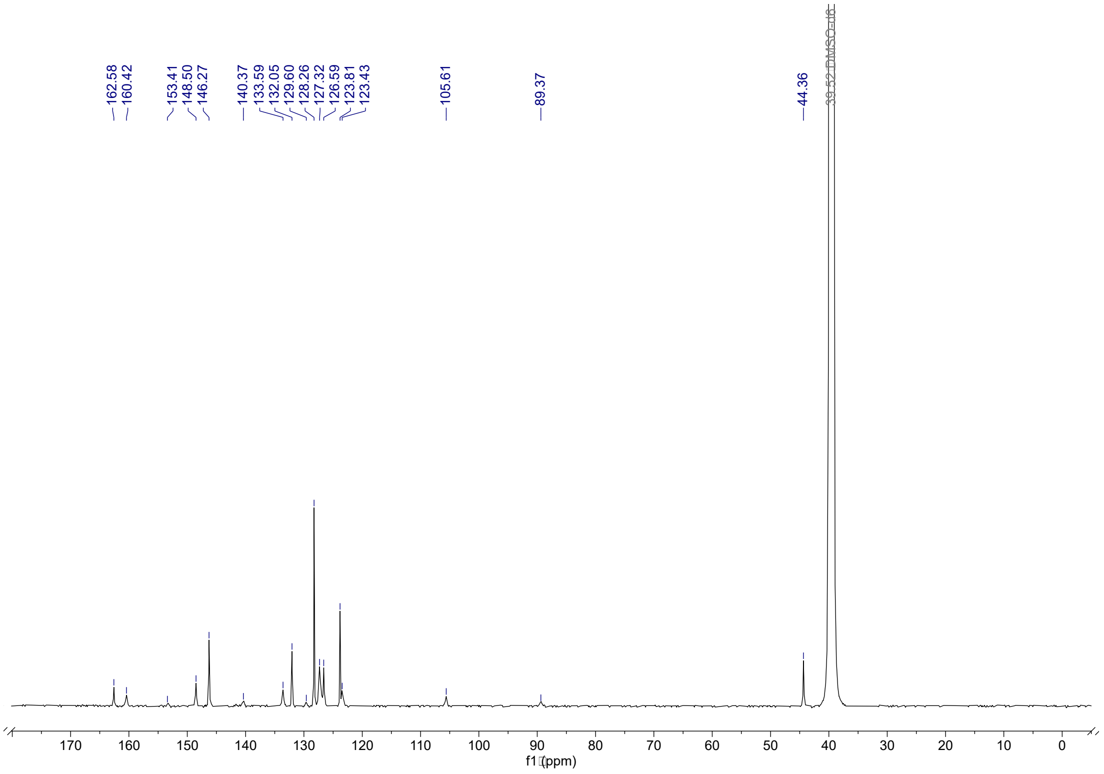

^{1}H NMR (400 MHz, DMSO-d_6) and ^{13}C NMR (101 MHz, DMSO-d_6) of compound 8

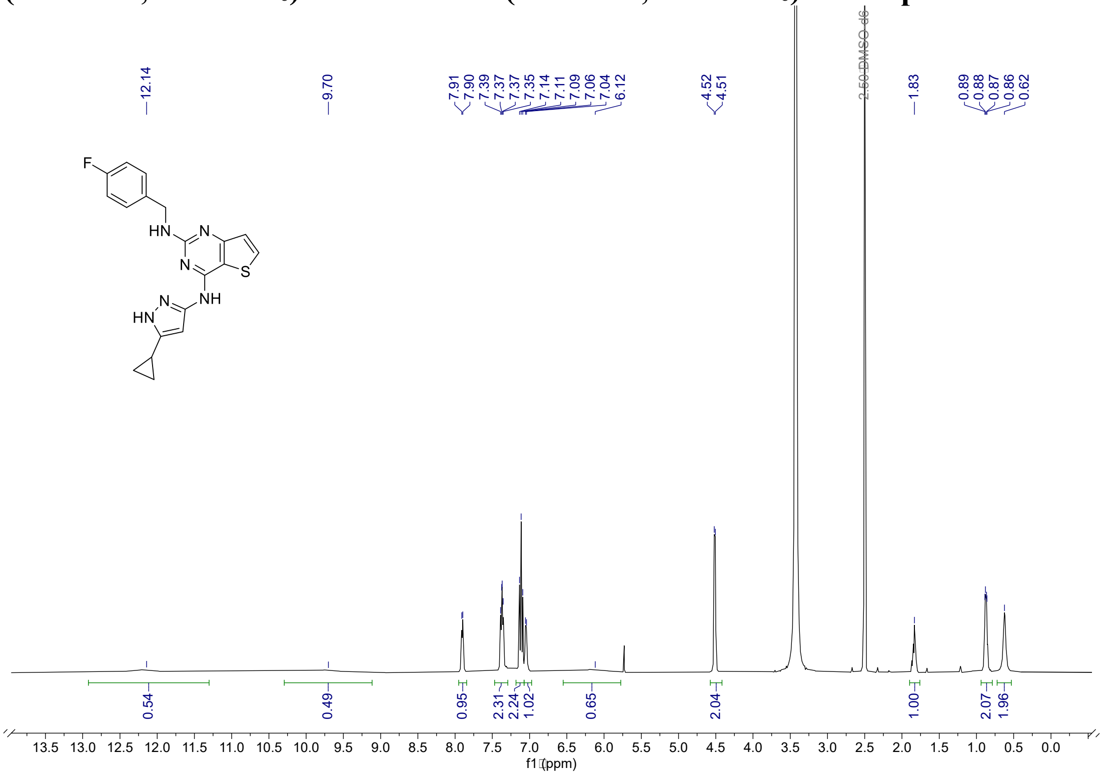

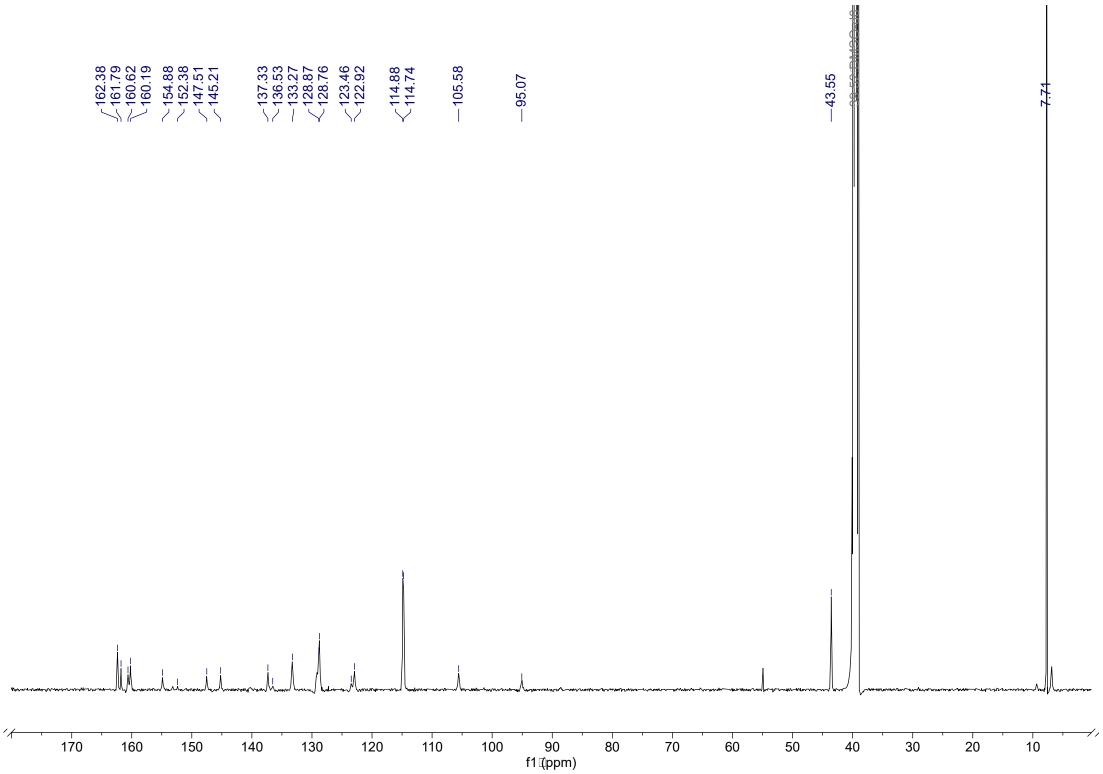

^{1}H NMR (400 MHz, DMSO-d_6) and ^{13}C NMR (101 MHz, DMSO-d_6) of compound 9

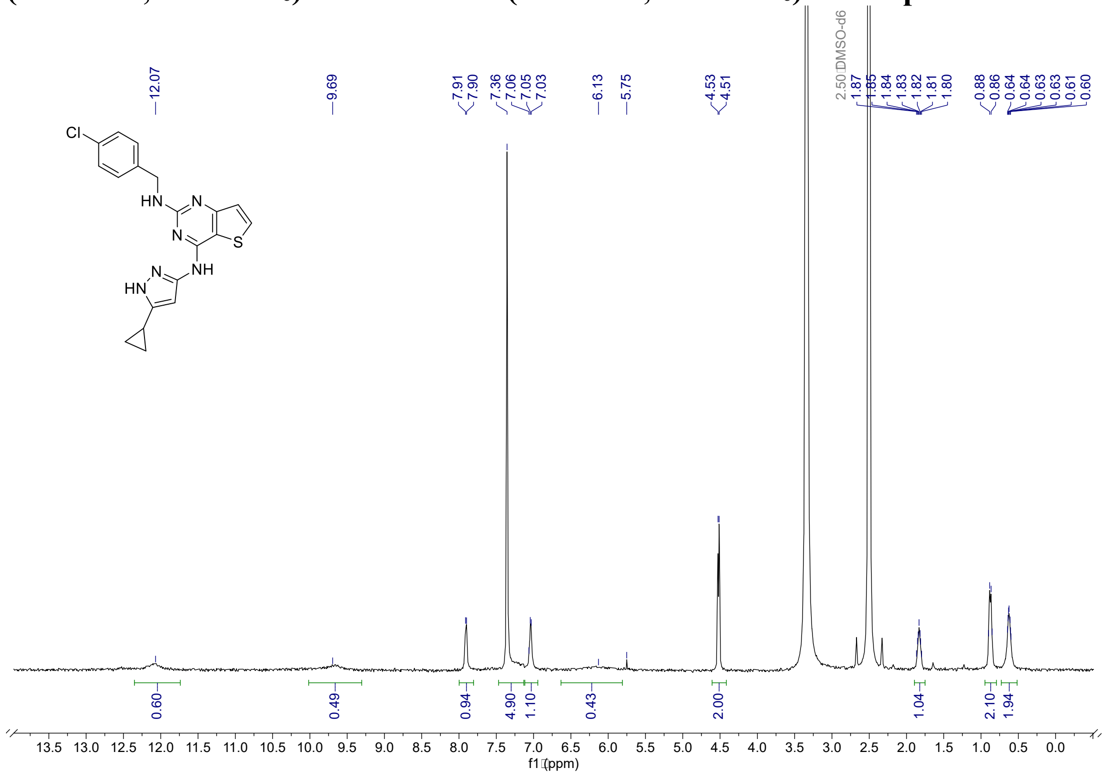

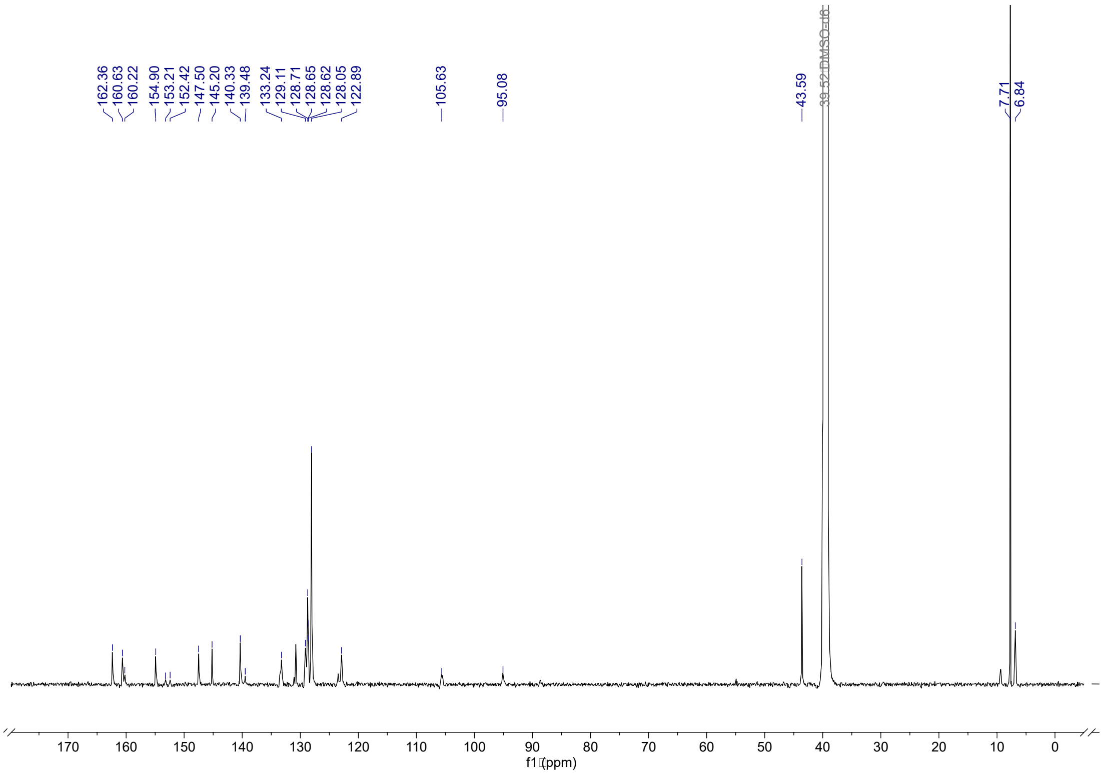

^{1}H NMR (400 MHz, DMSO-d_6) and ^{13}C NMR (101 MHz, DMSO-d_6) of compound 10

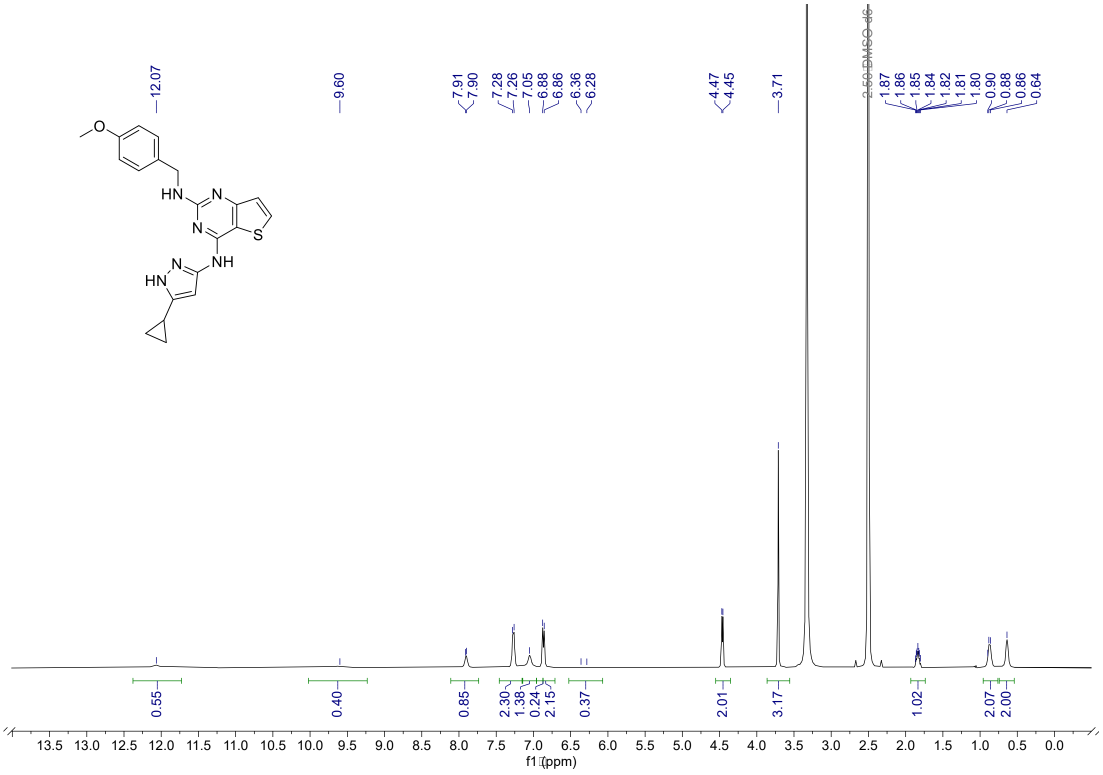

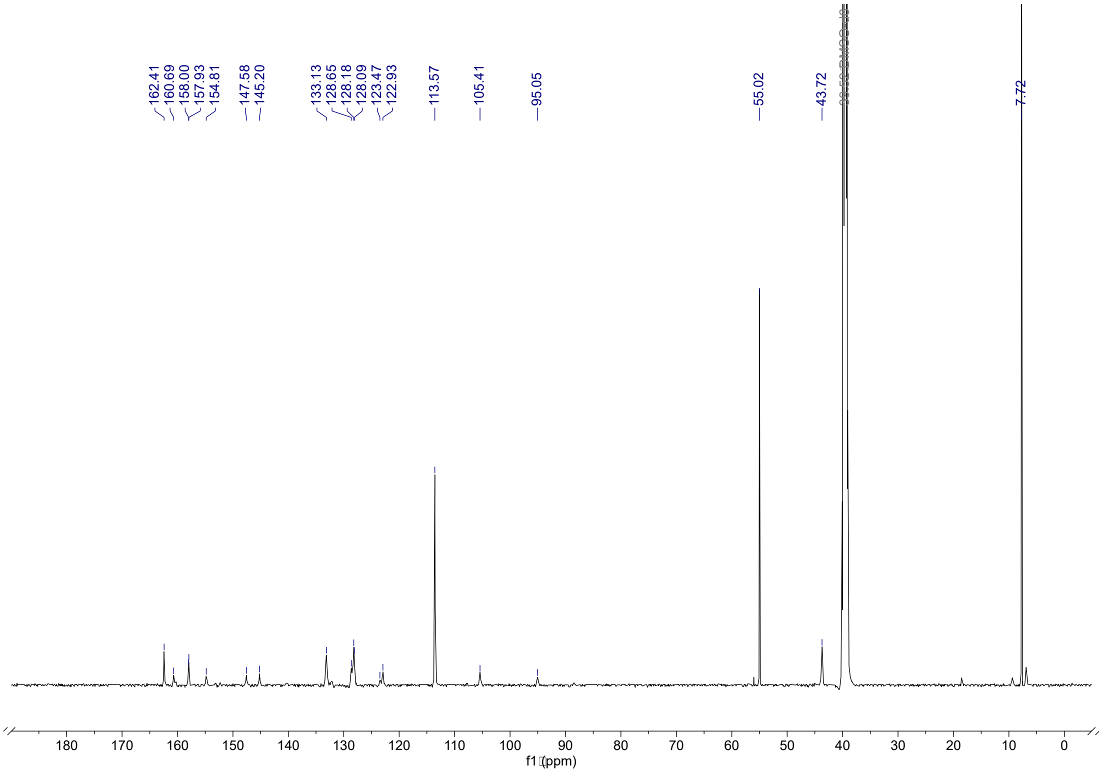

^{1}H NMR (400 MHz, DMSO-d_6) and ^{13}C NMR (101 MHz, DMSO-d_6) of compound 11

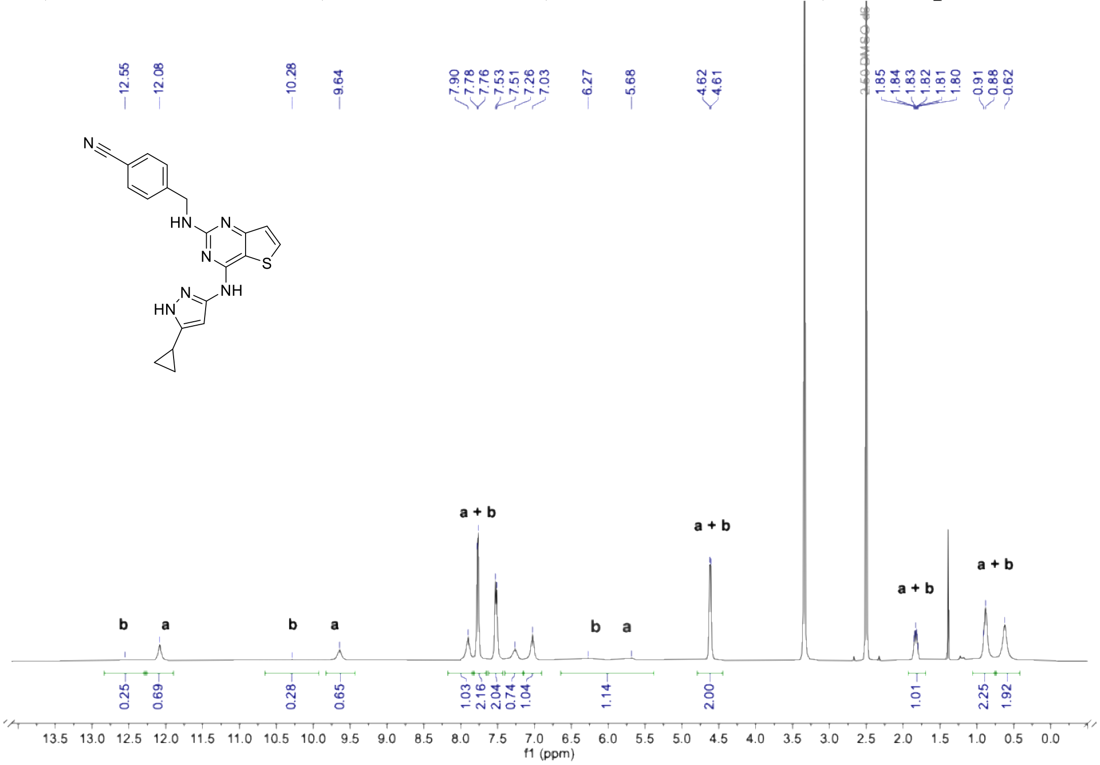

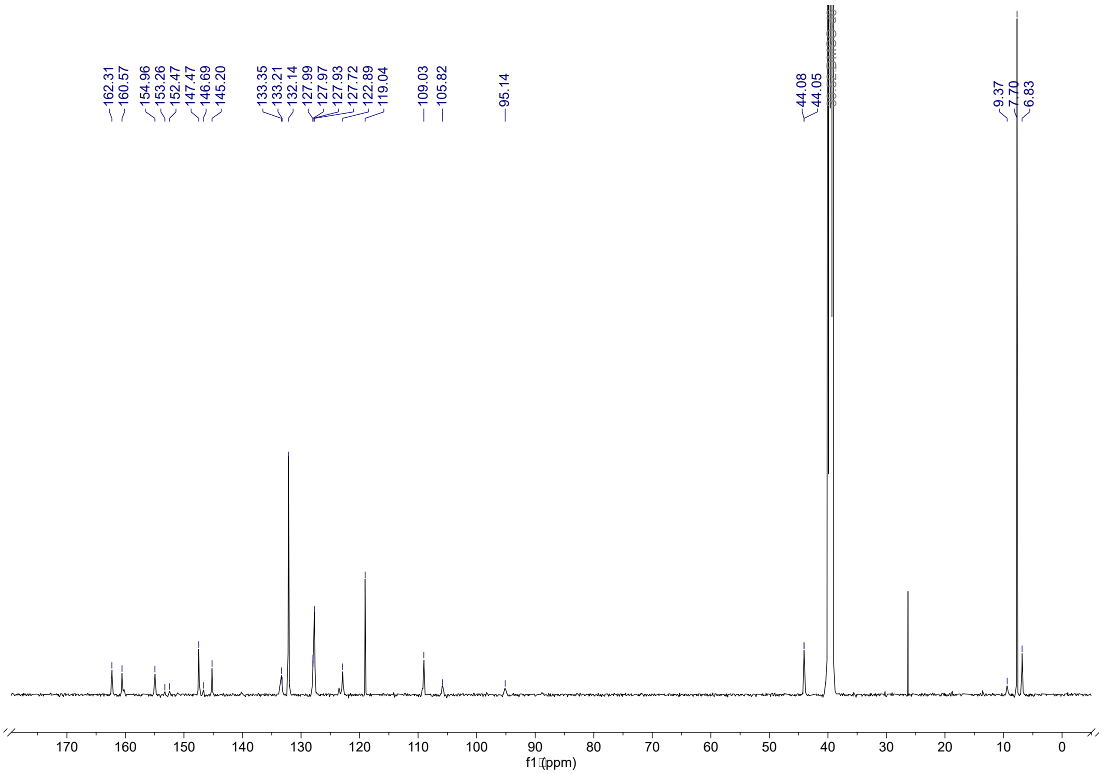

^{1}H NMR (400 MHz, DMSO-d_6) and ^{13}C NMR (101 MHz, DMSO-d_6) of compound 12

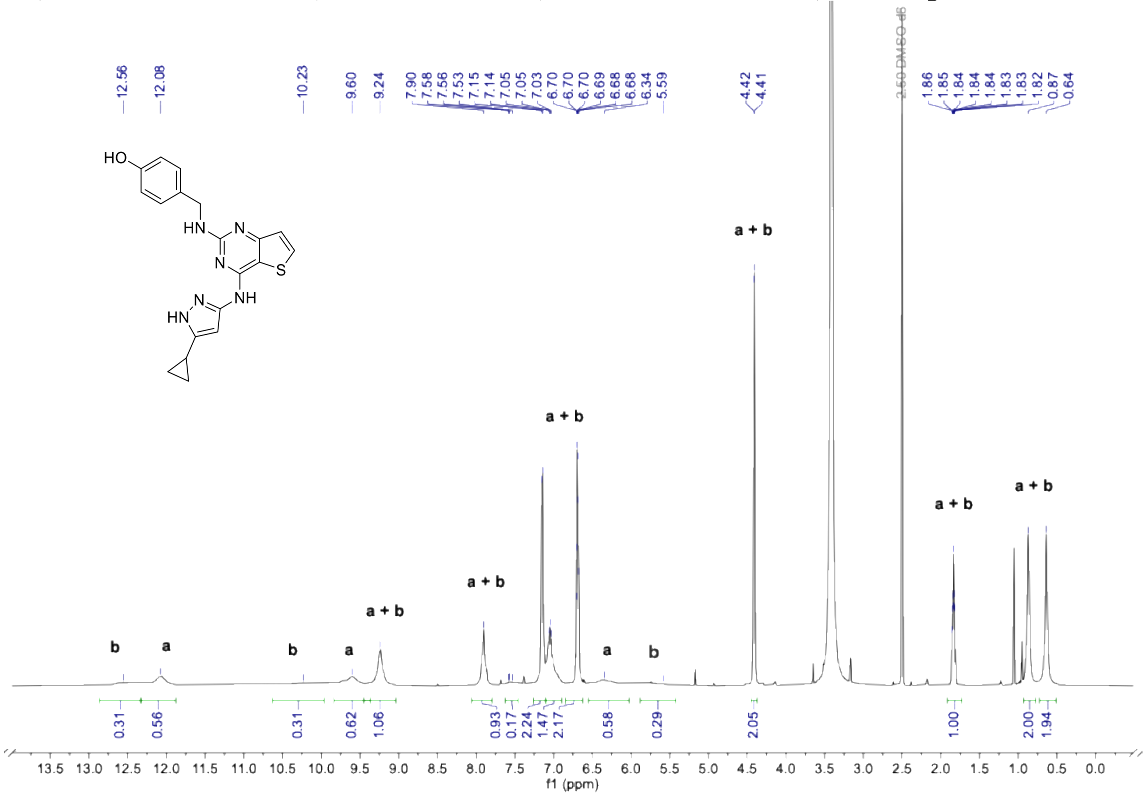

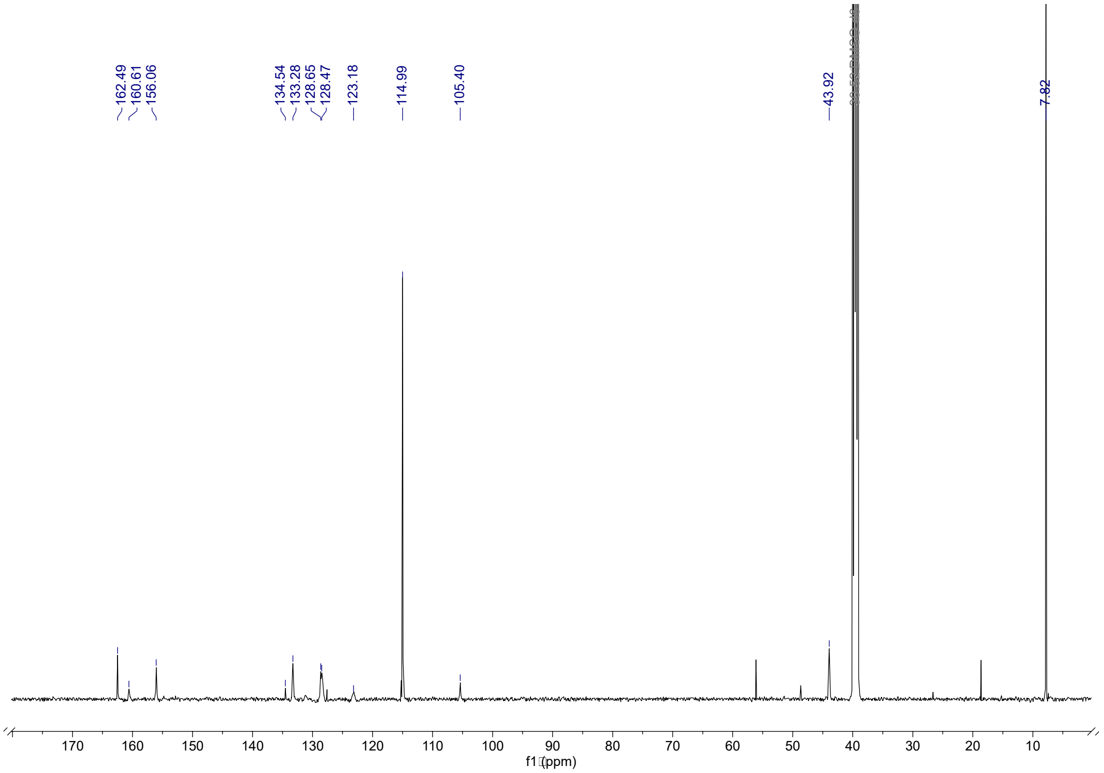

^{1}H NMR (400 MHz, DMSO-*d*$_6$) and ^{13}C NMR (101 MHz, DMSO-*d*$_6$) of compound 13

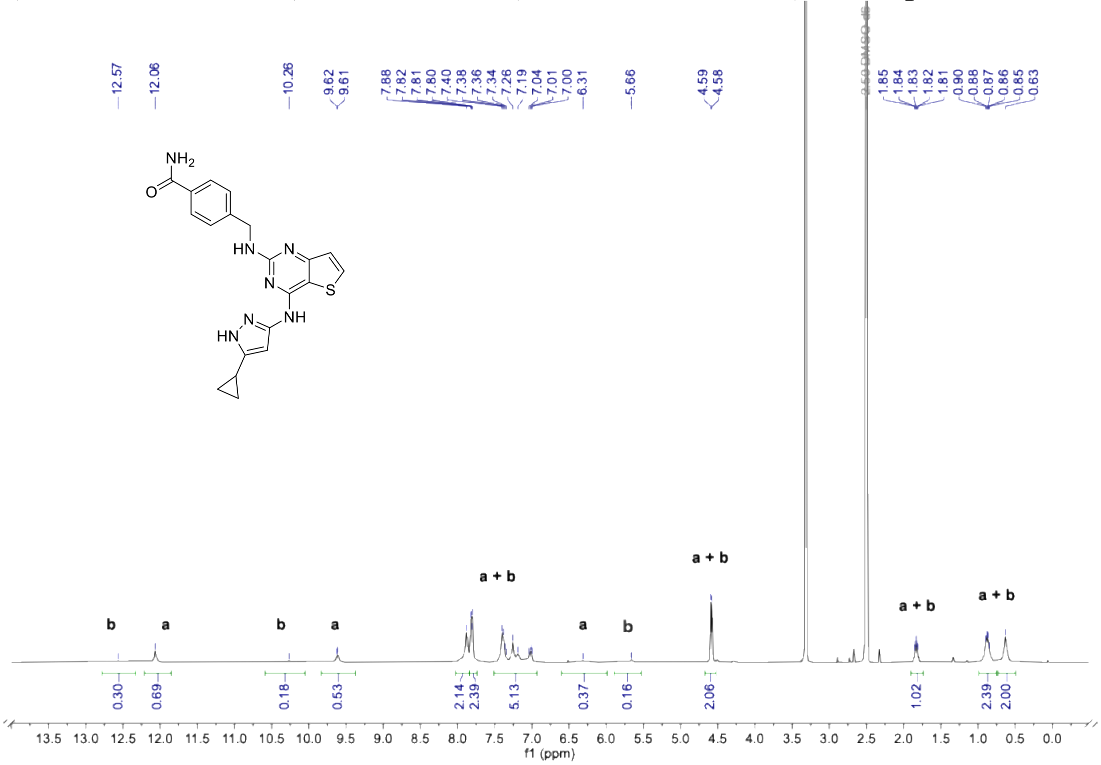

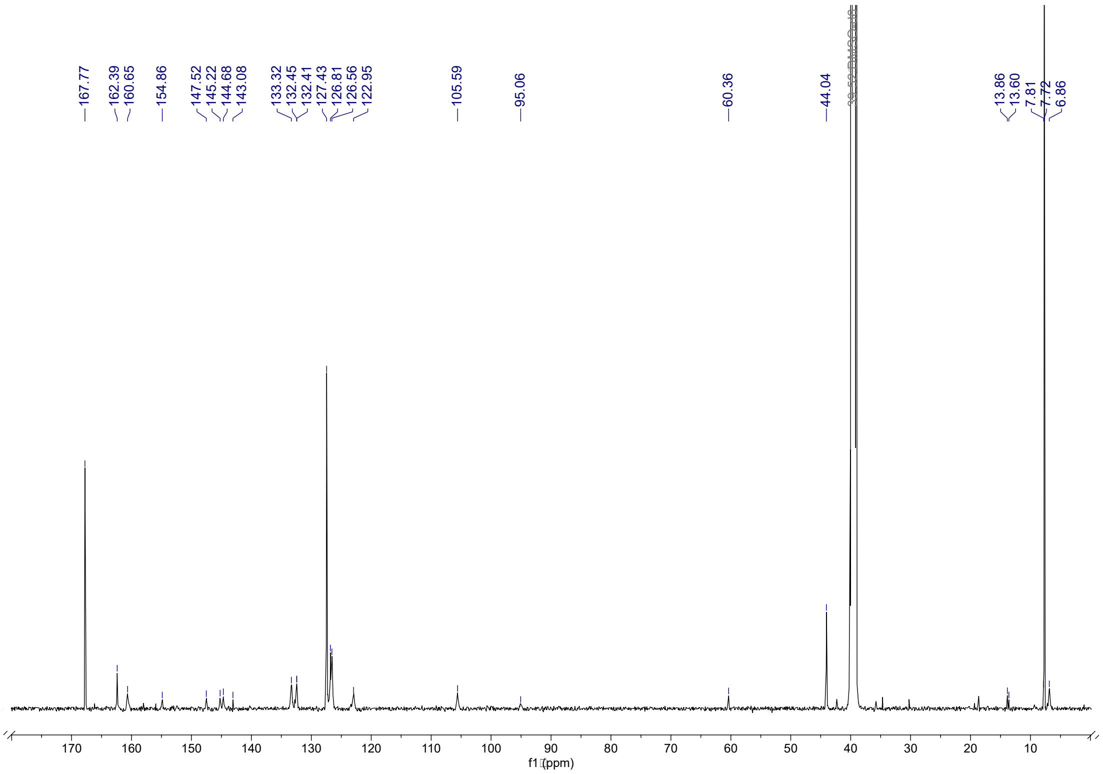

^{1}H NMR (400 MHz, DMSO-d_6) and ^{13}C NMR (101 MHz, DMSO-d_6) of compound 14

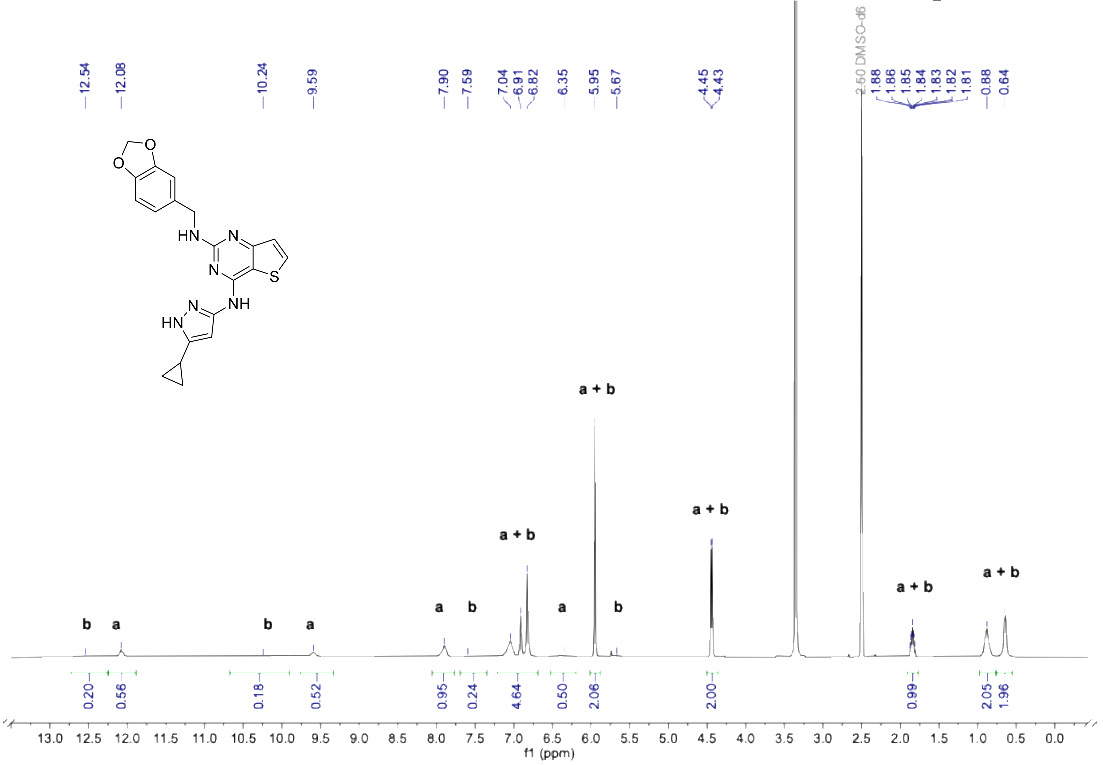

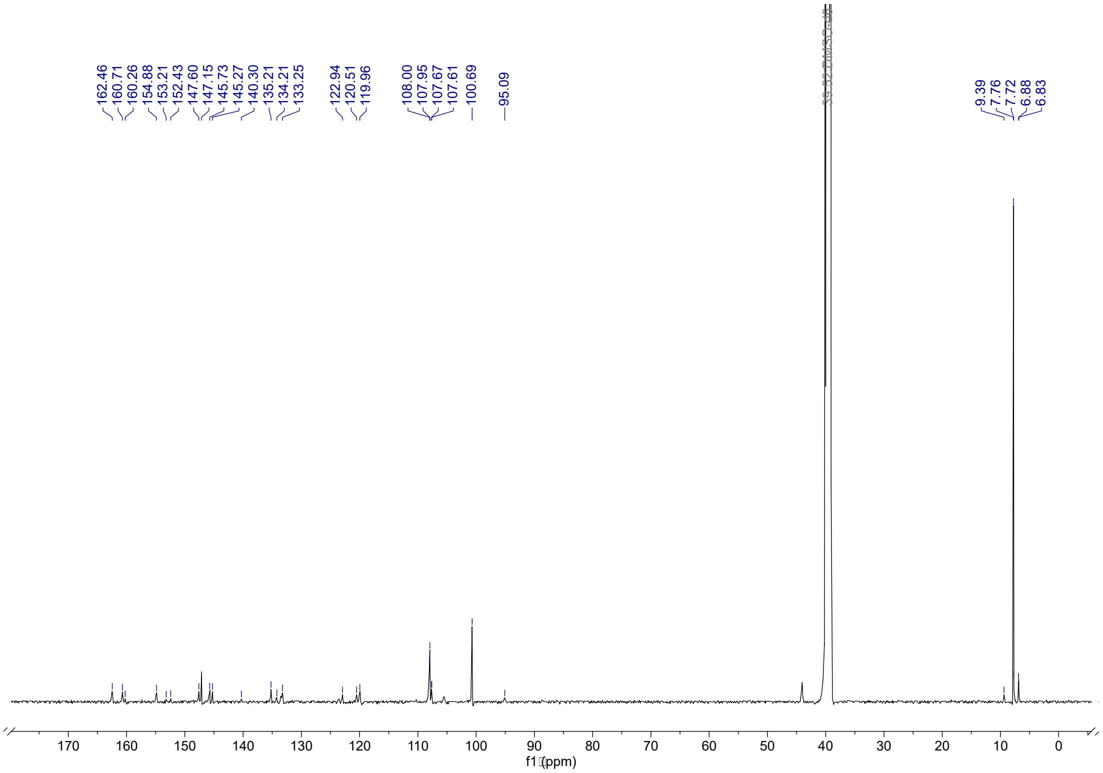

^{1}H NMR (400 MHz, DMSO-d_6) and ^{13}C NMR (101 MHz, DMSO-d_6) of compound 15

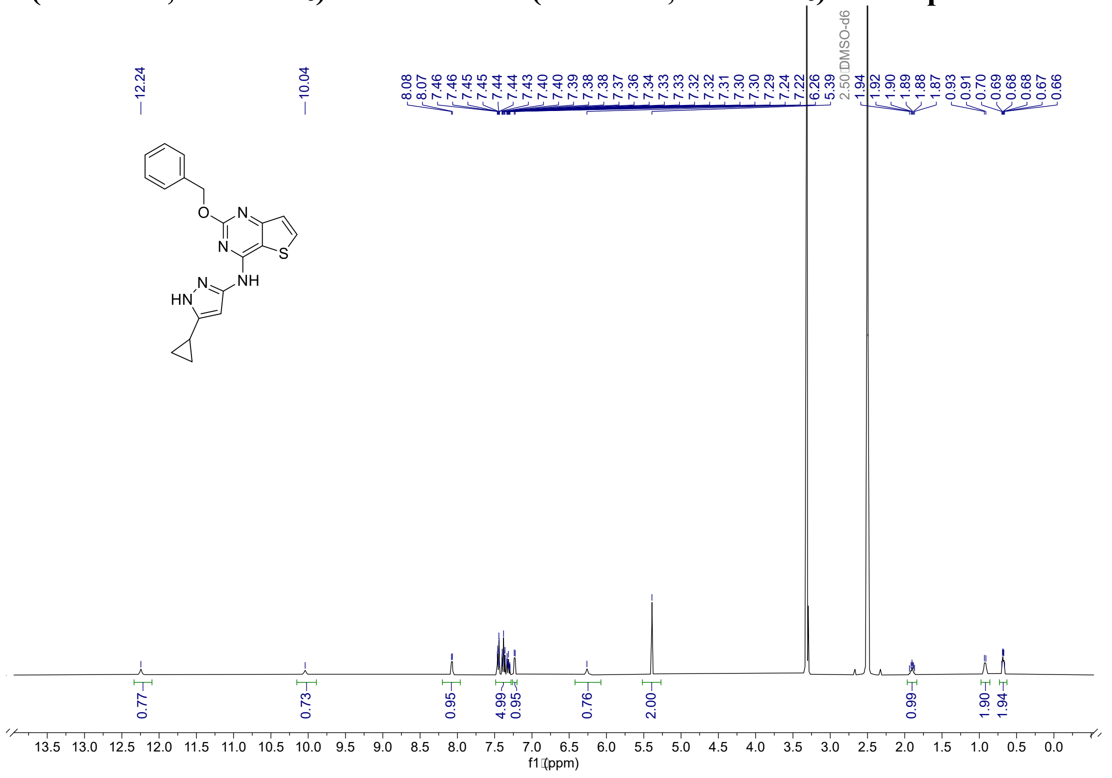

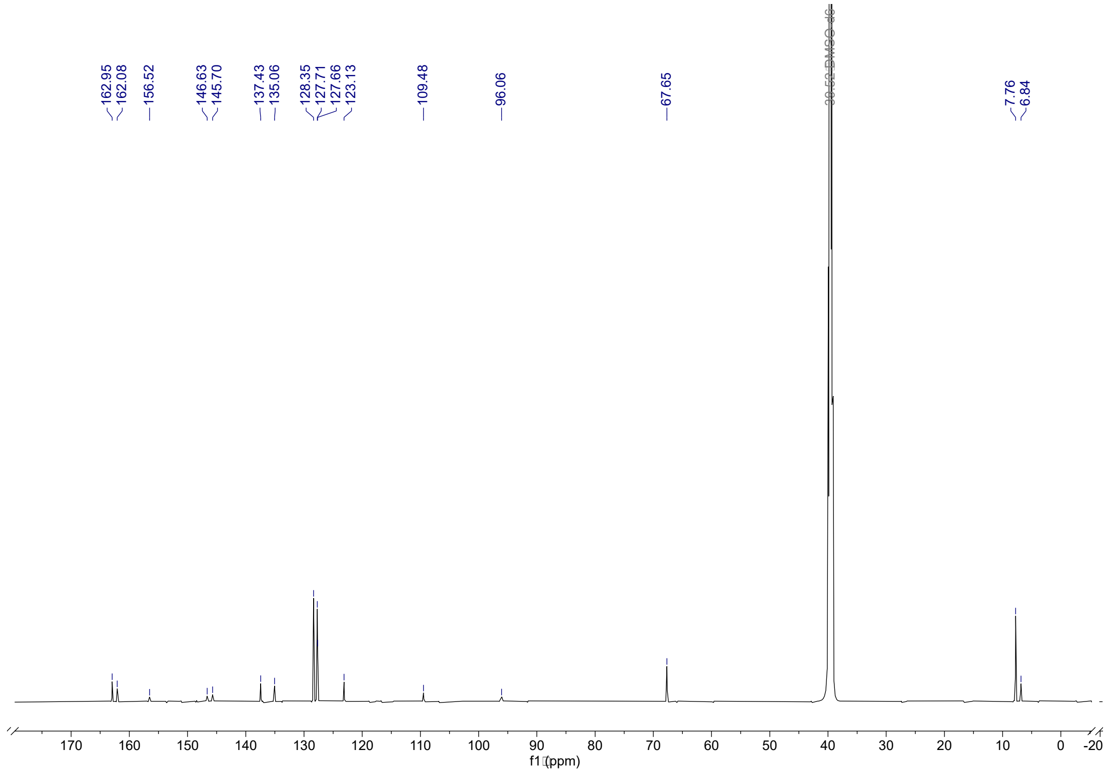

^{1}H NMR (400 MHz, DMSO-d_6) and ^{13}C NMR (101 MHz, DMSO-d_6) of compound 16

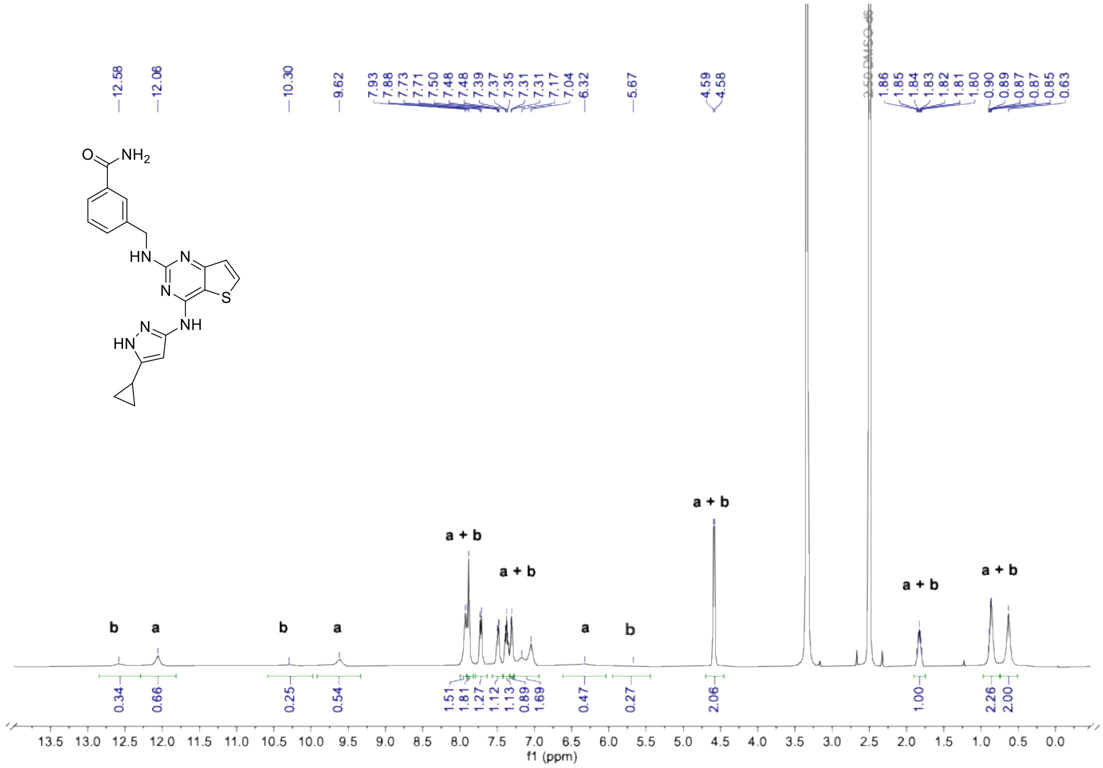

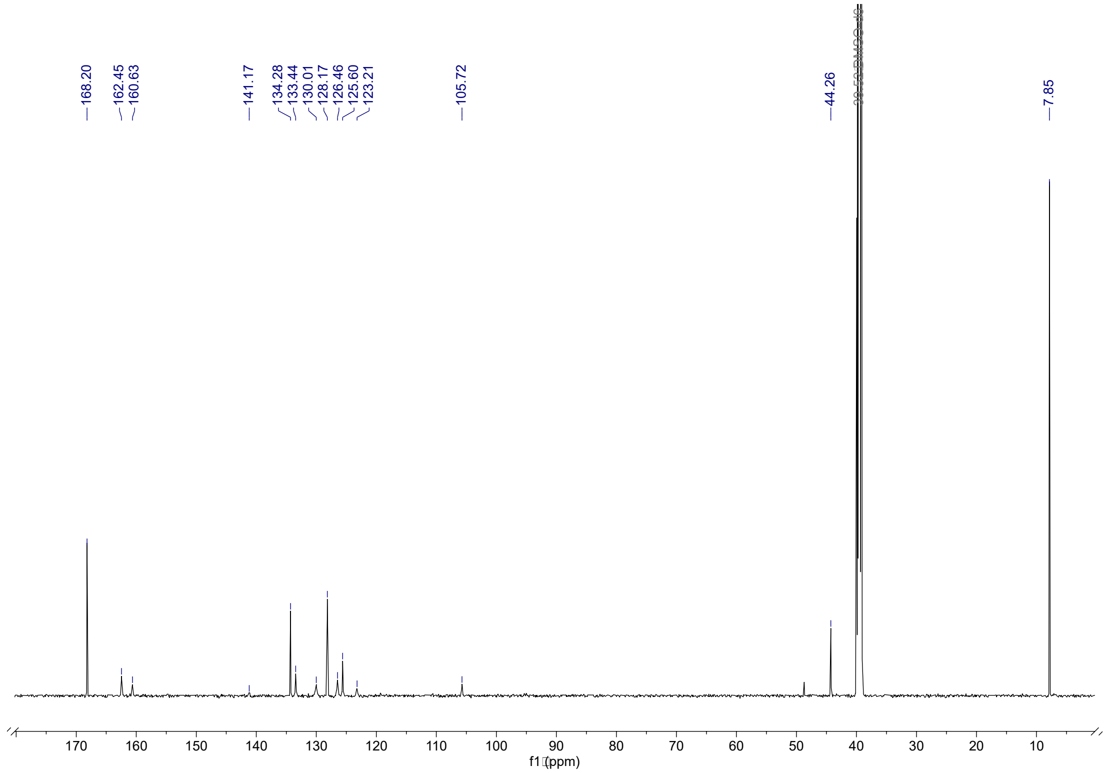

^{1}H NMR (400 MHz, DMSO-d_6) and ^{13}C NMR (101 MHz, DMSO-d_6) of compound 17

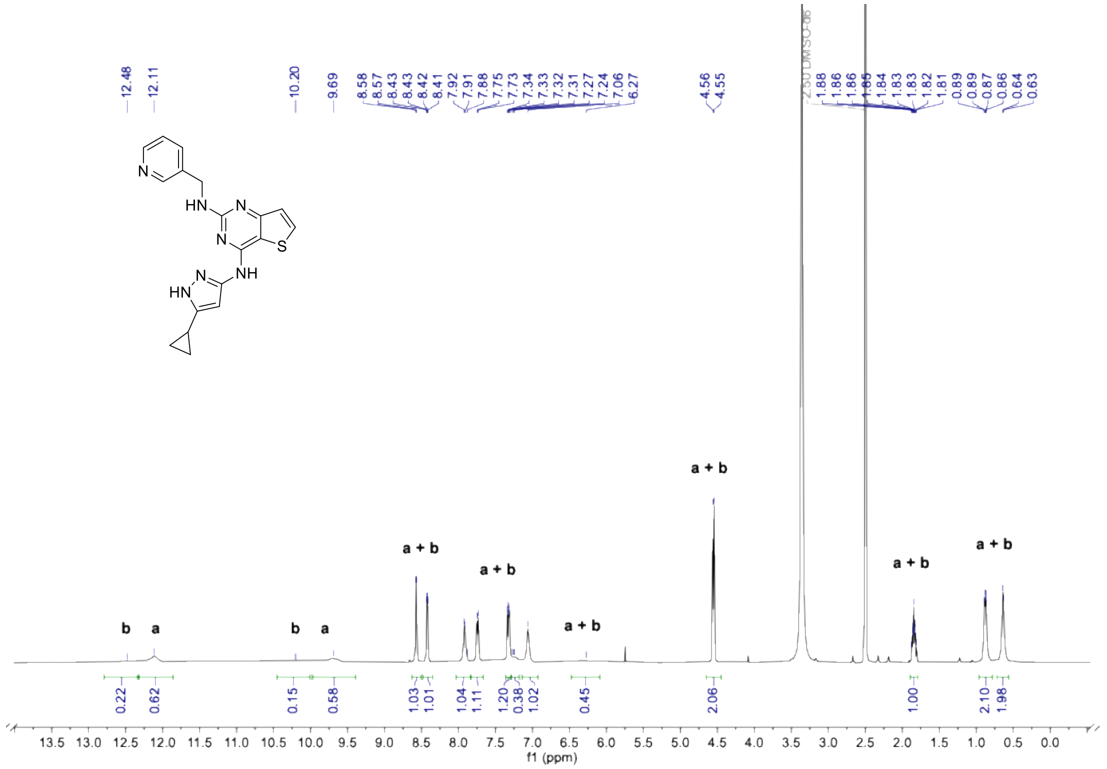

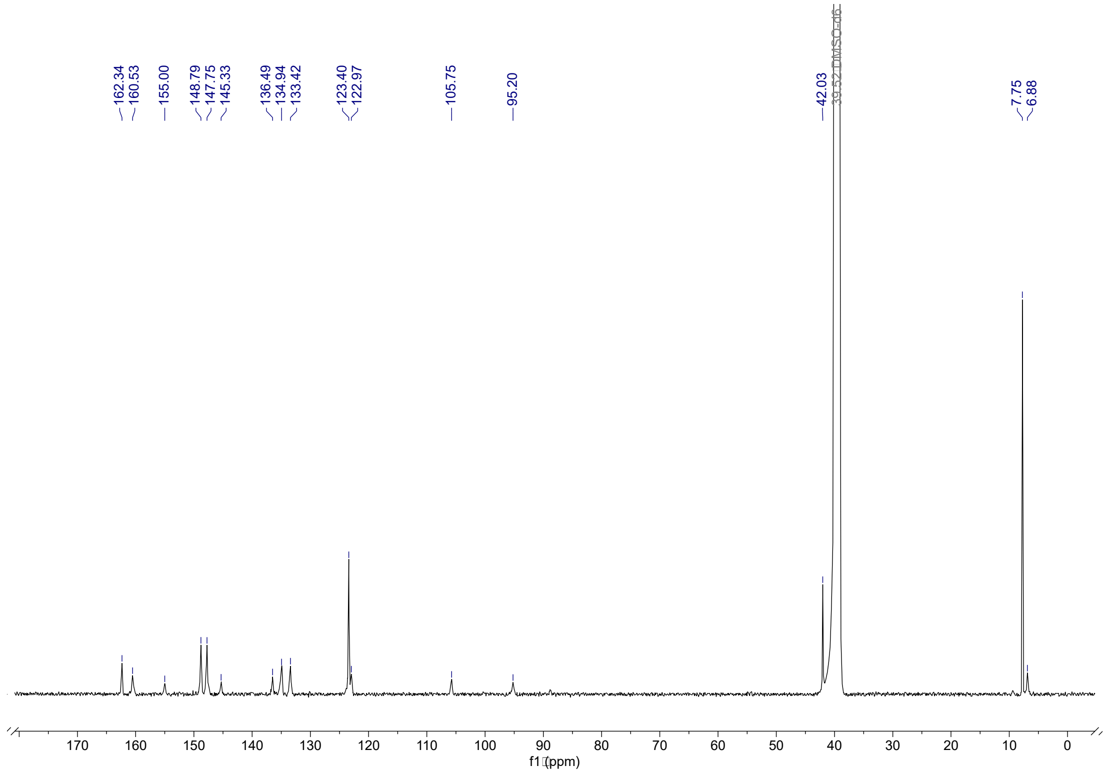

^{1}H NMR (400 MHz, DMSO-d_6) and ^{13}C NMR (101 MHz, DMSO-d_6) of compound 18

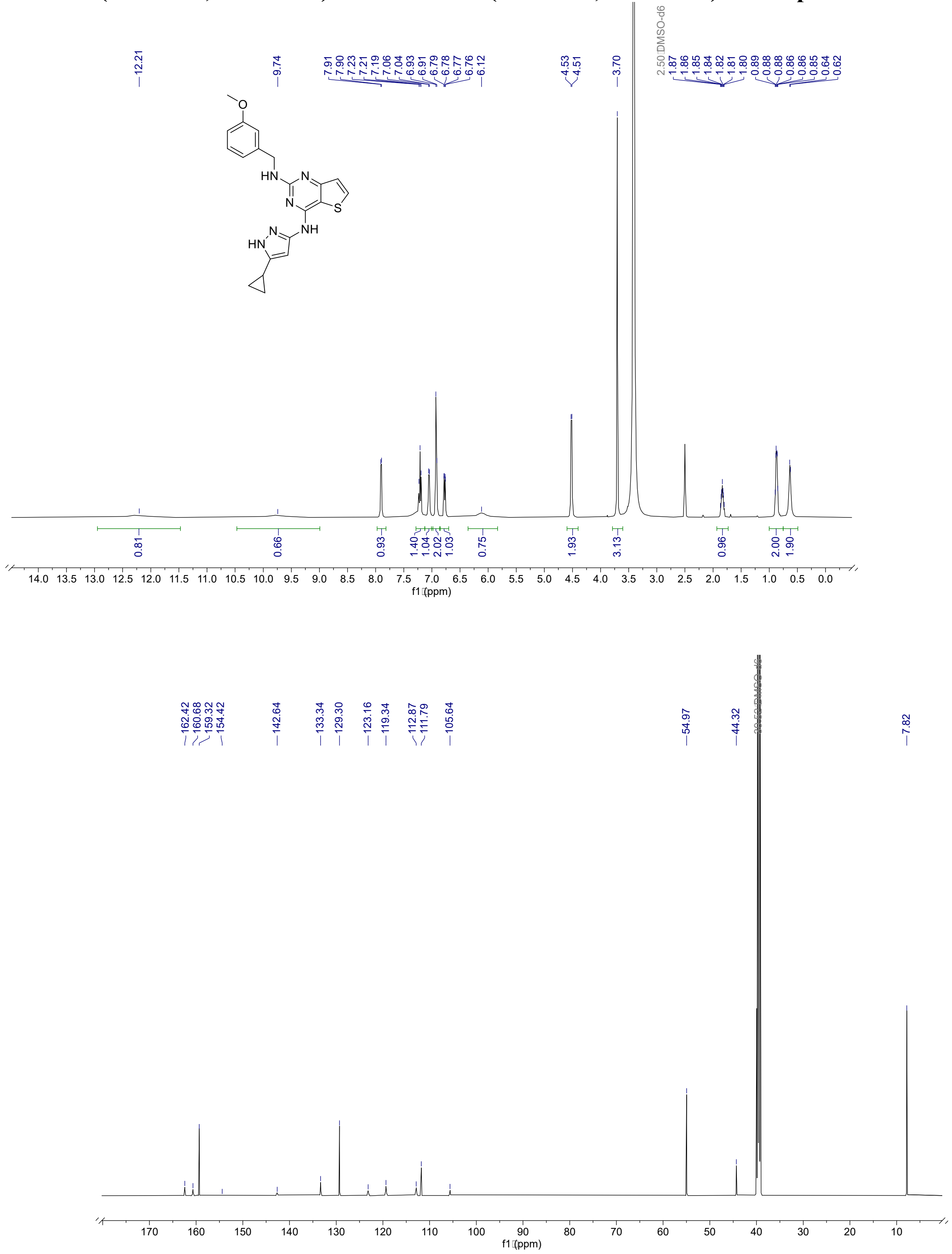

^{1}H NMR (400 MHz, DMSO-d_6) and ^{13}C NMR (101 MHz, DMSO-d_6) of compound 19

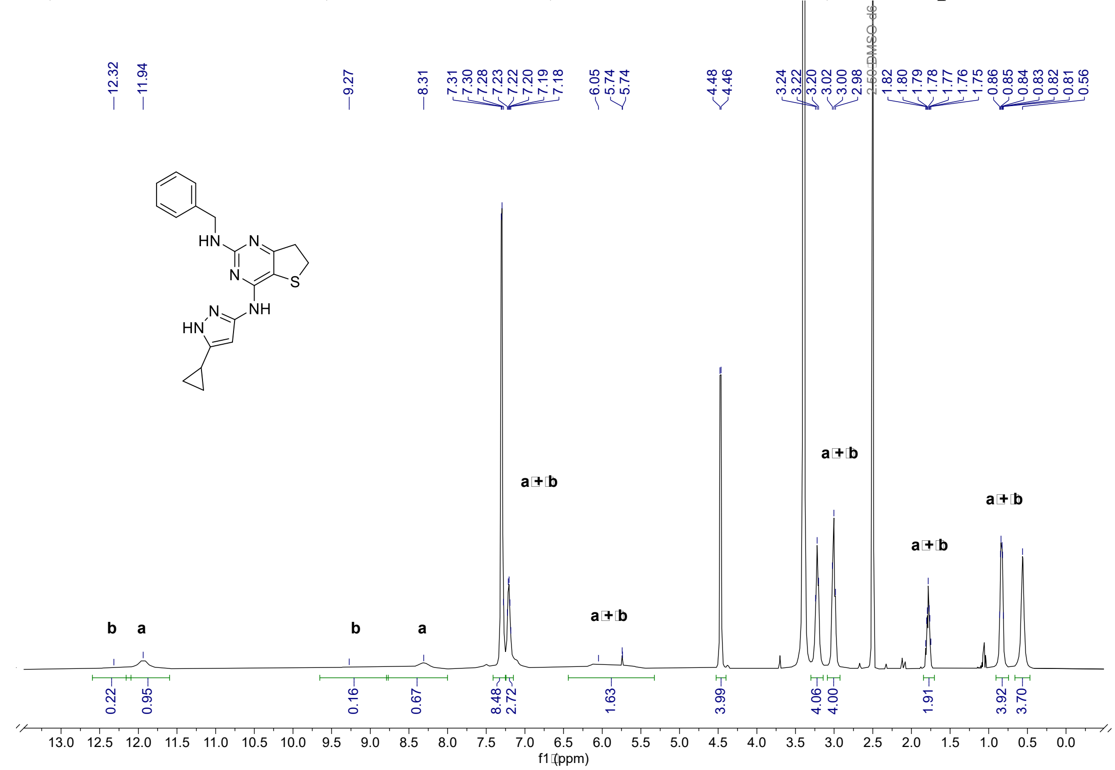

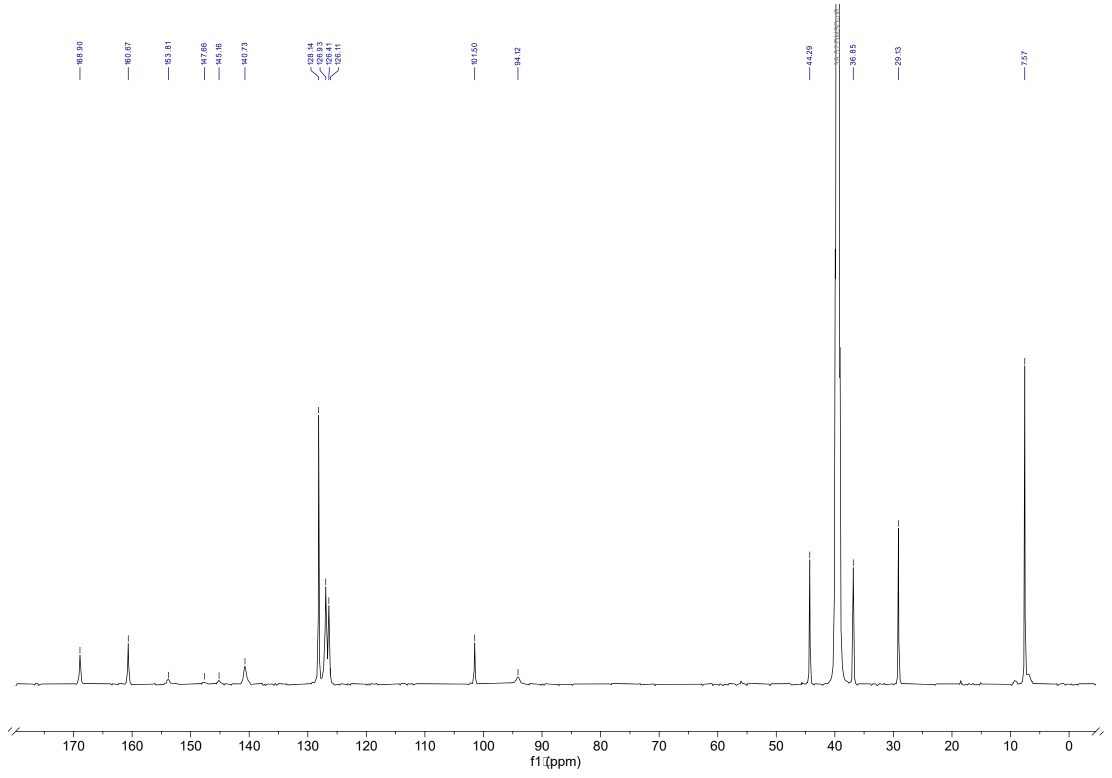

^{1}H NMR (400 MHz, DMSO-d_6) and ^{13}C NMR (101 MHz, DMSO-d_6) of compound 20

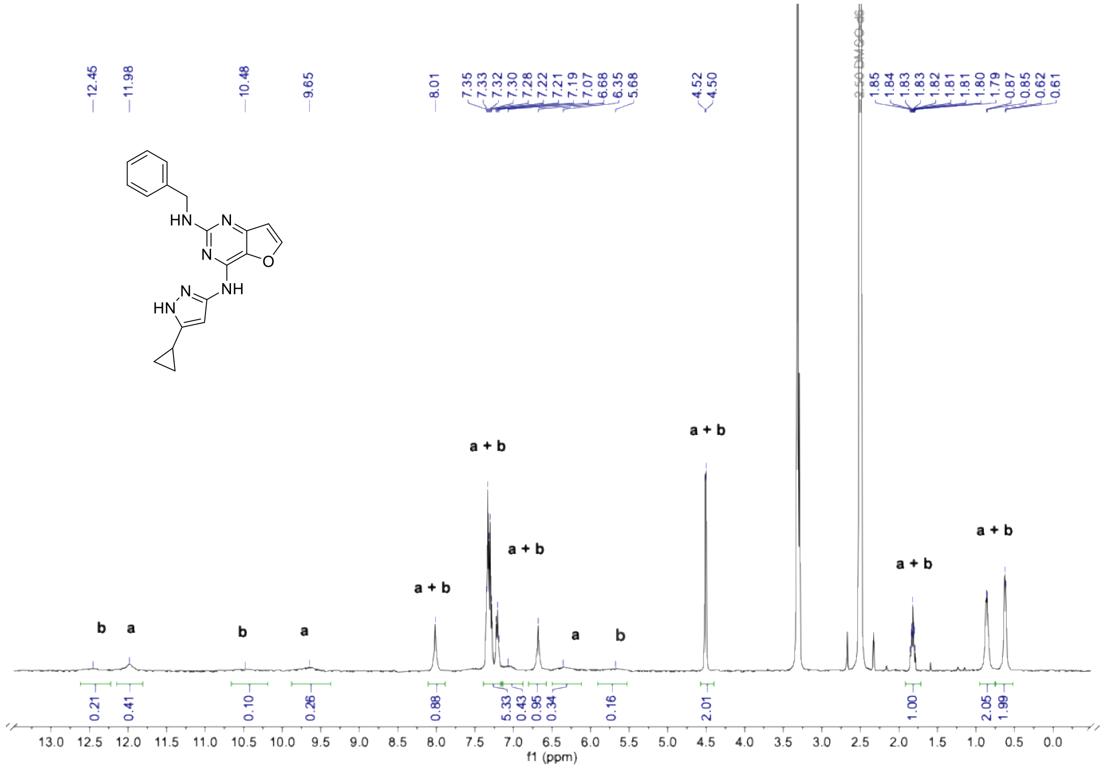

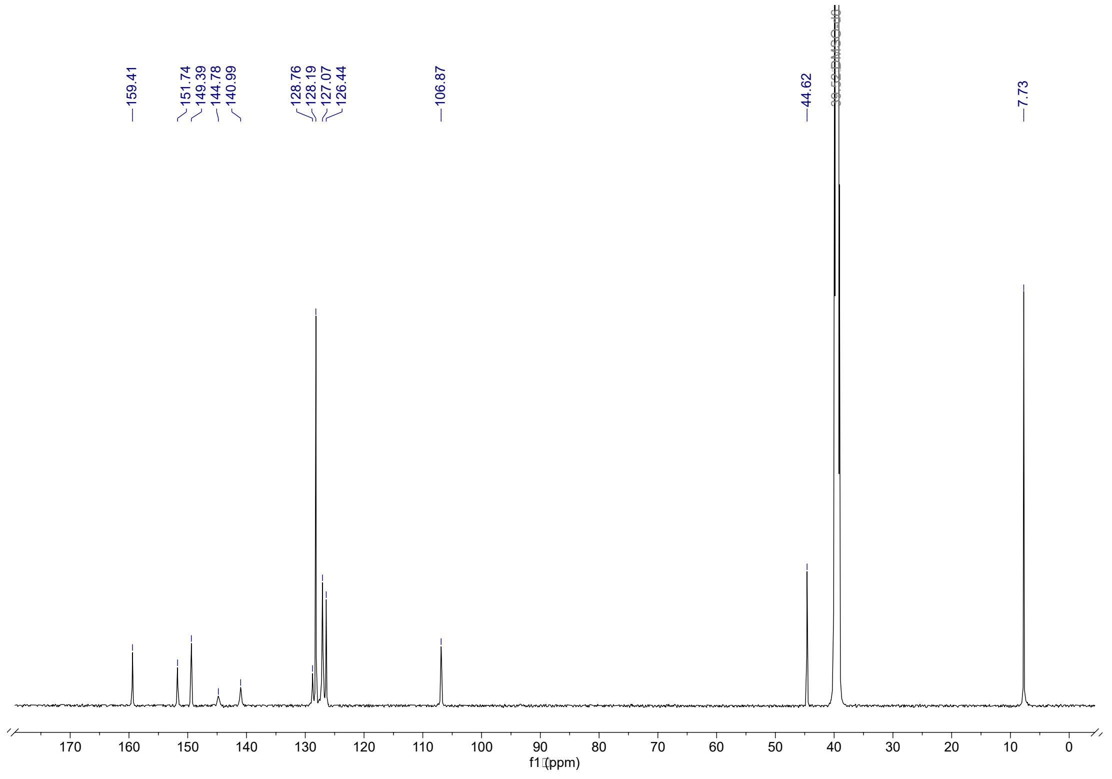

^{1}H NMR (400 MHz, DMSO-d_6) and ^{13}C NMR (101 MHz, DMSO-d_6) of compound 21

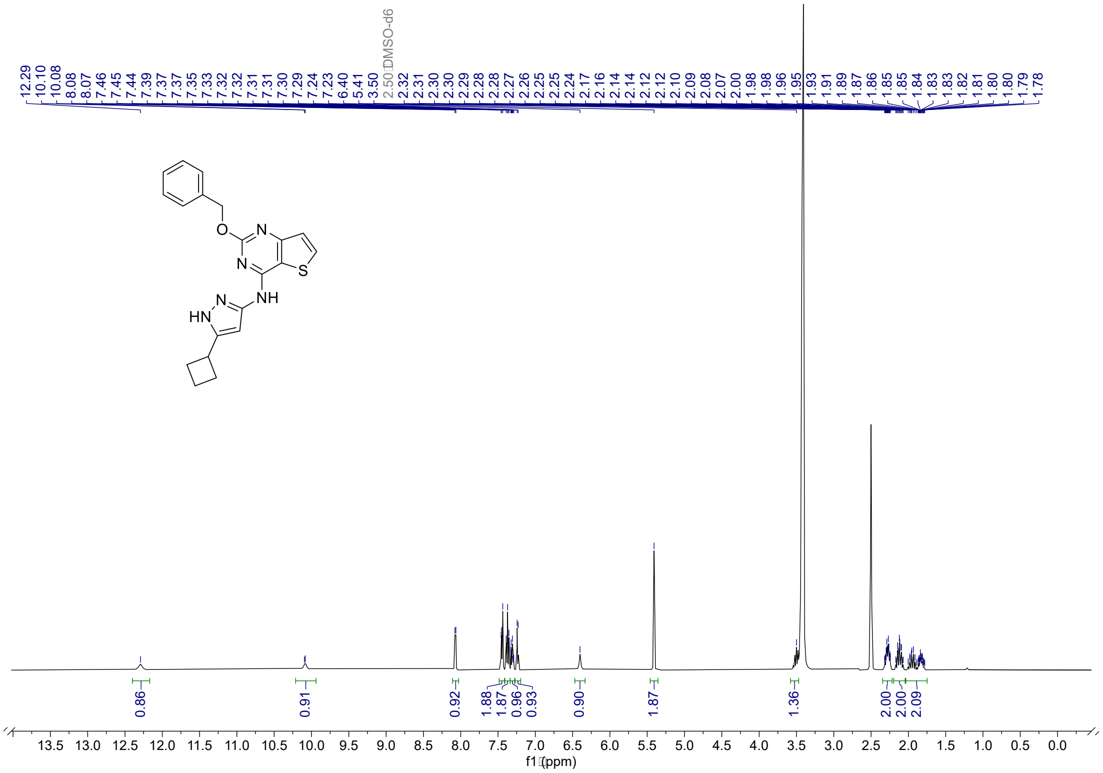

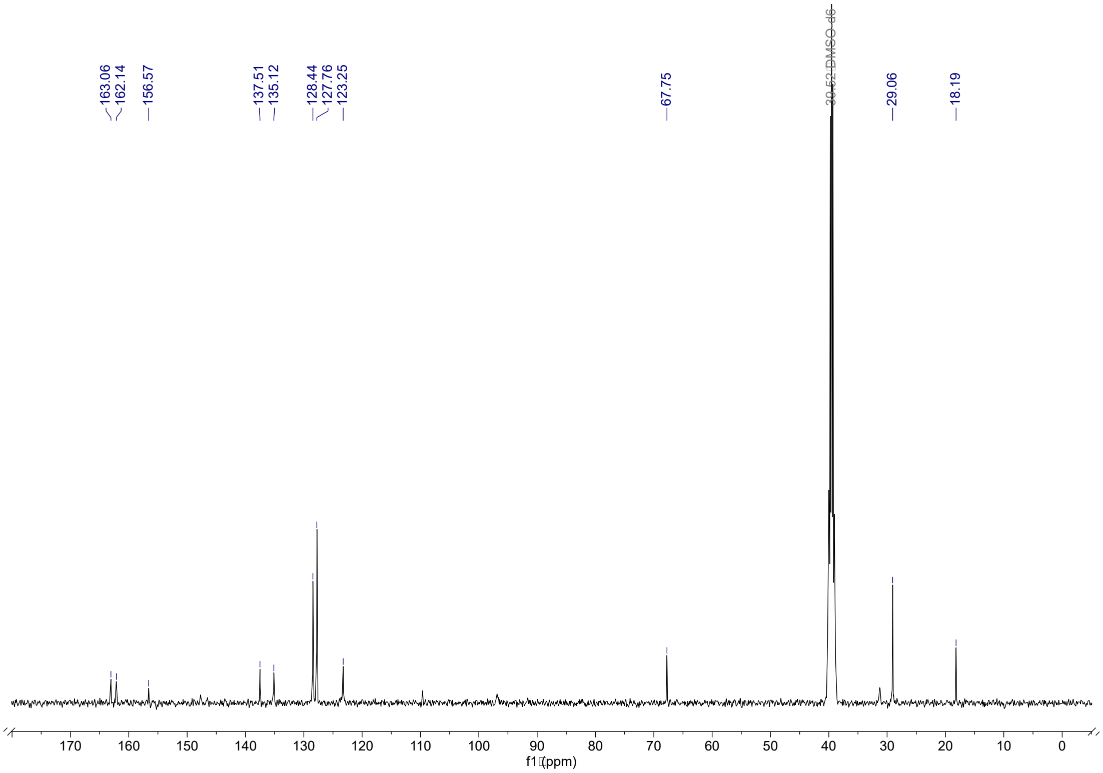

^{1}H NMR (400 MHz, DMSO-d_6) and ^{13}C NMR (101 MHz, DMSO-d_6) of compound 22

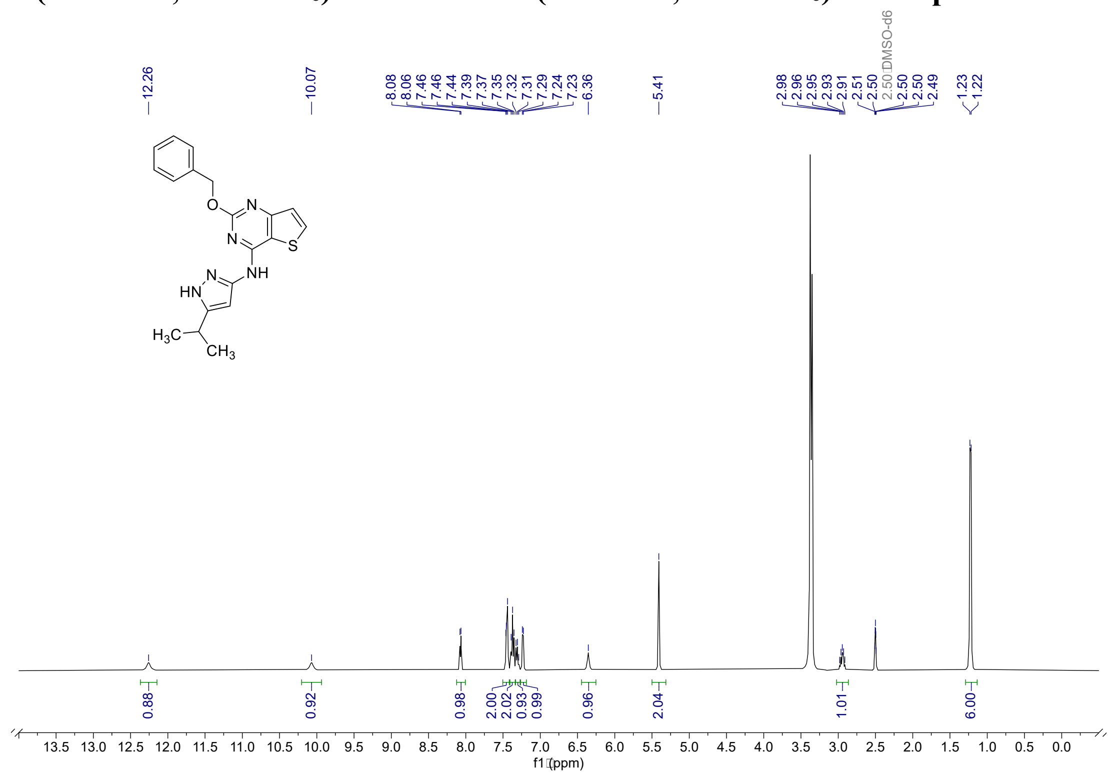

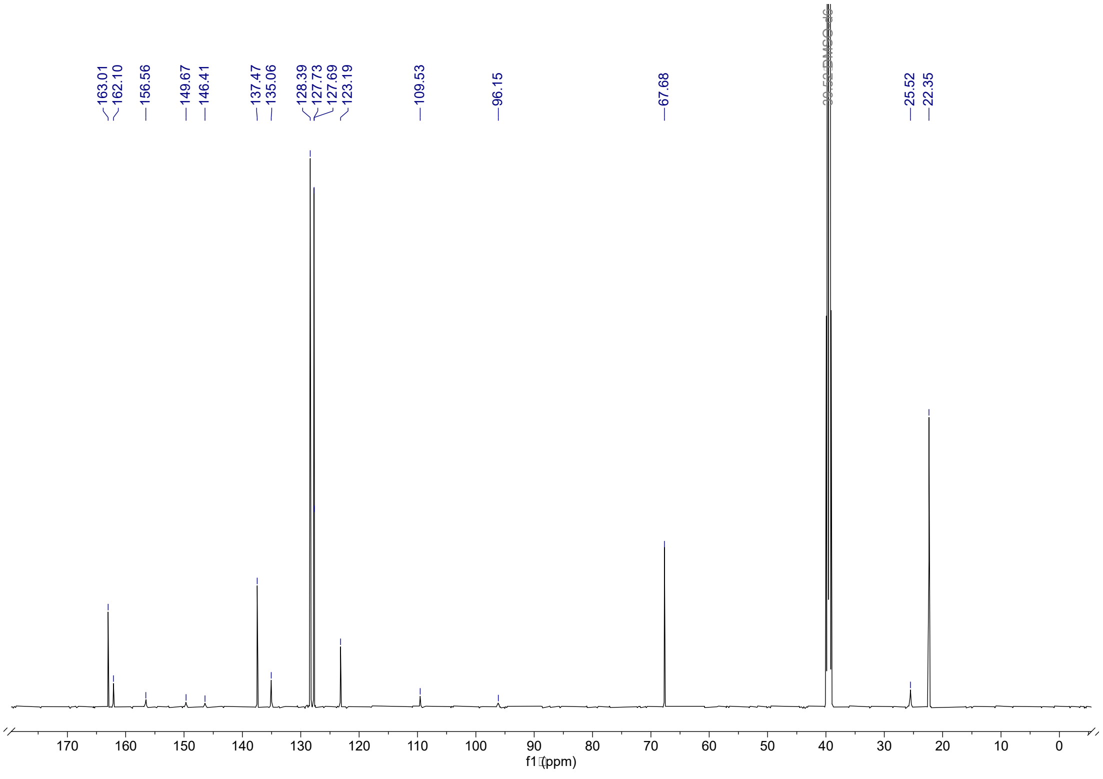

^{1}H NMR (400 MHz, DMSO-*d*$_6$) and ^{13}C NMR (101 MHz, DMSO-*d*$_6$) of compound 23

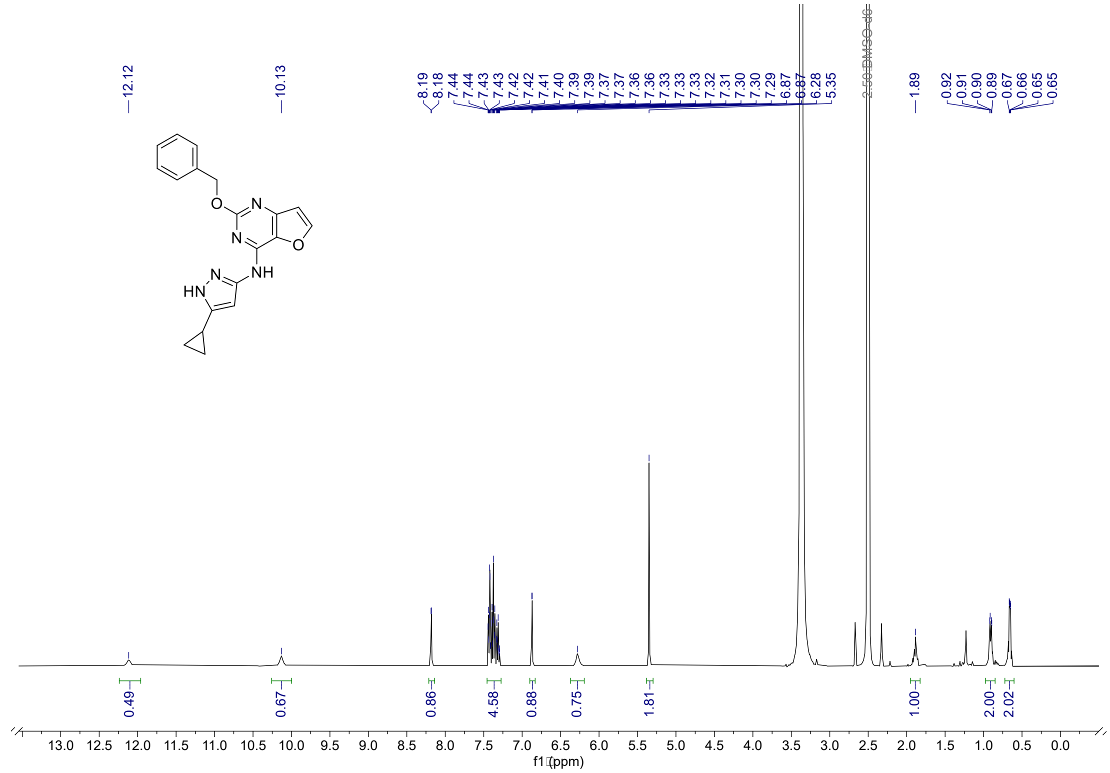

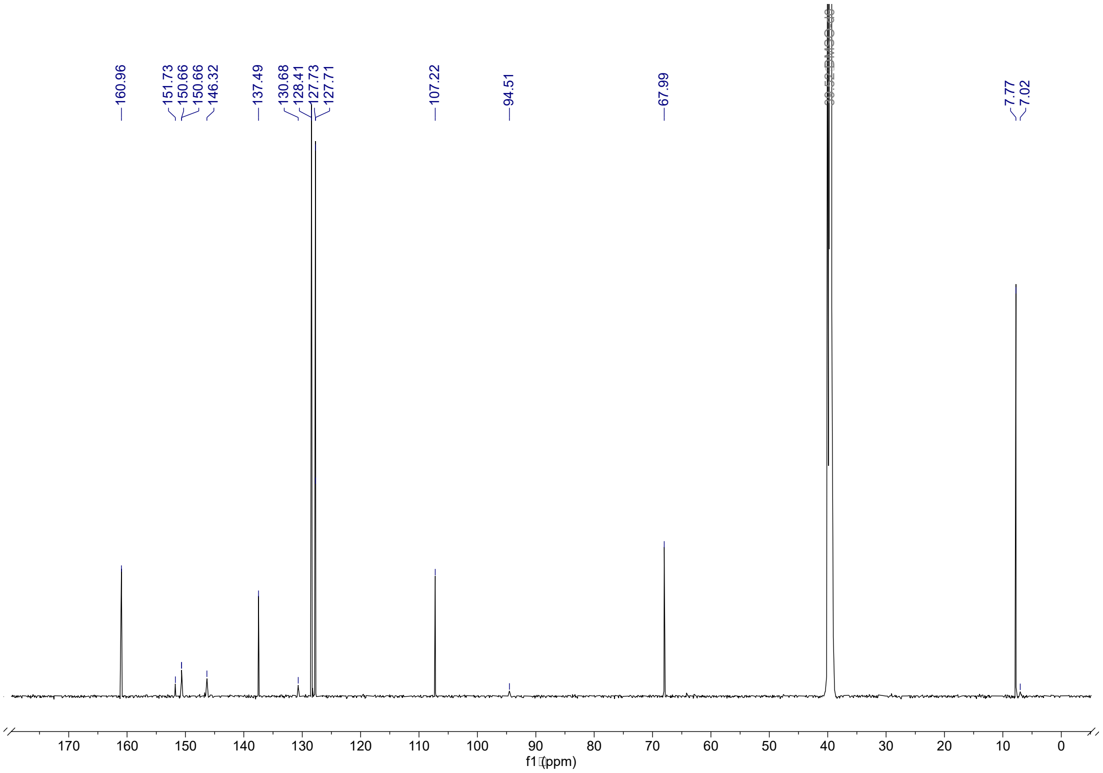

^{1}H NMR (400 MHz, DMSO-*d*$_6$) and ^{13}C NMR (101 MHz, DMSO-*d*$_6$) of compound 24

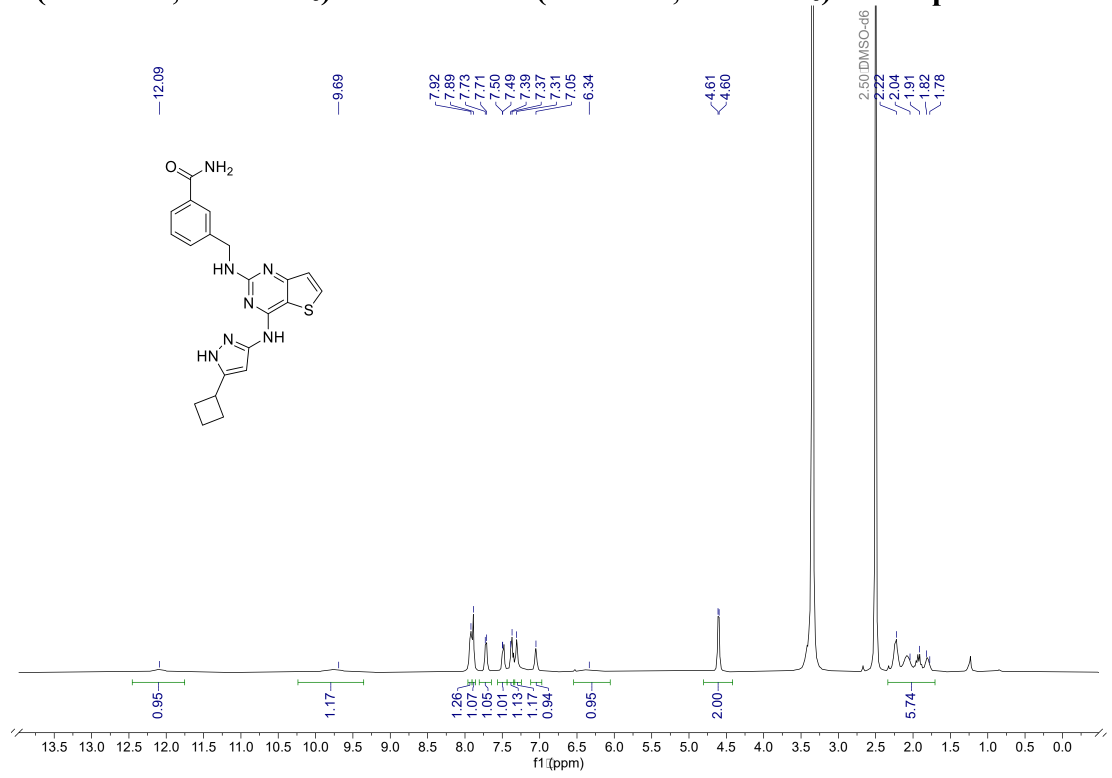

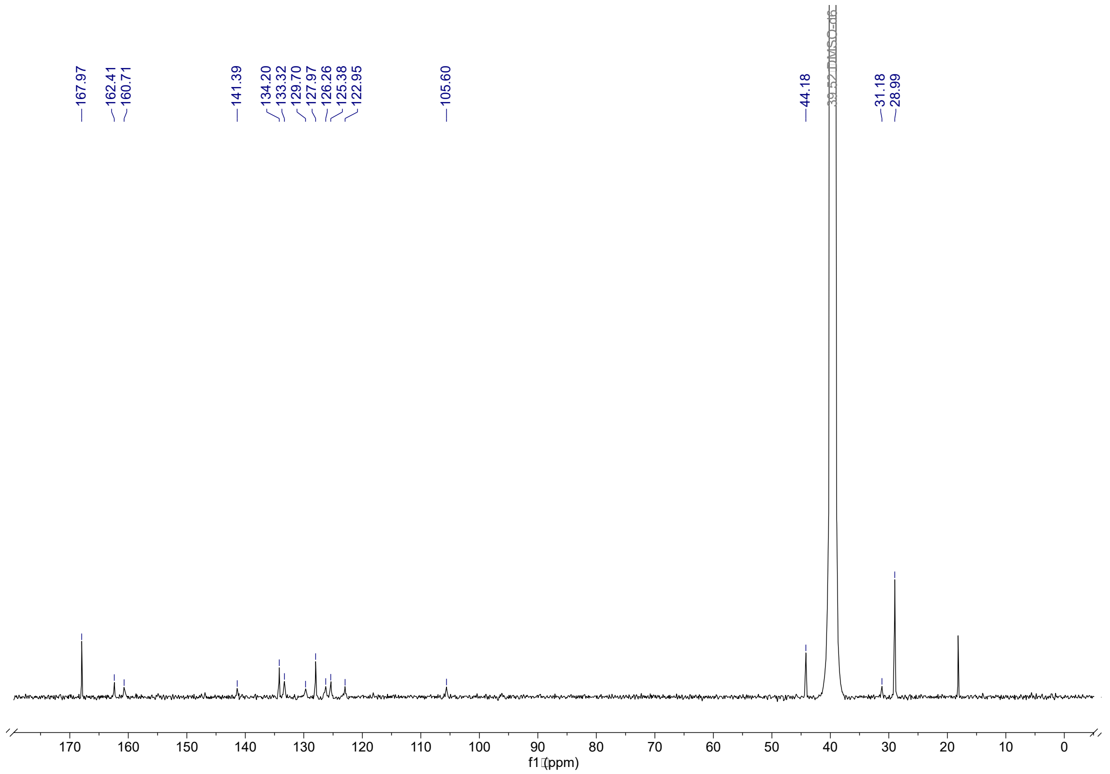

^{1}H NMR (400 MHz, DMSO-d_6) and ^{13}C NMR (101 MHz, DMSO-d_6) of compound 25

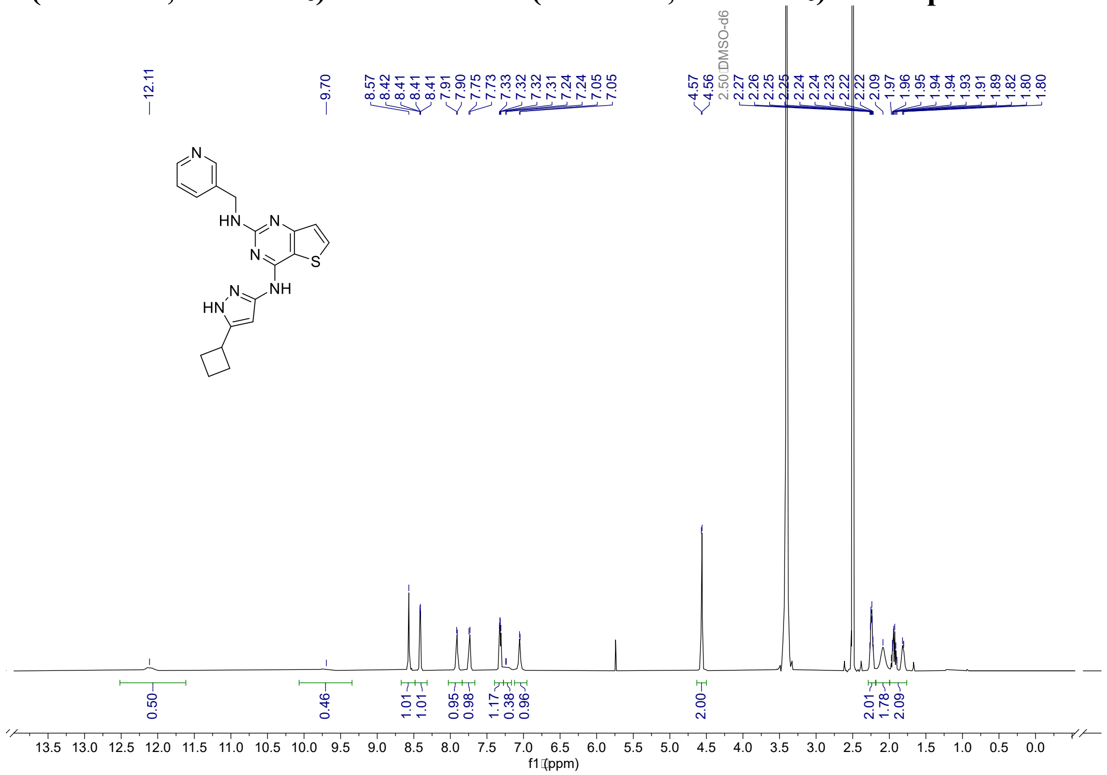

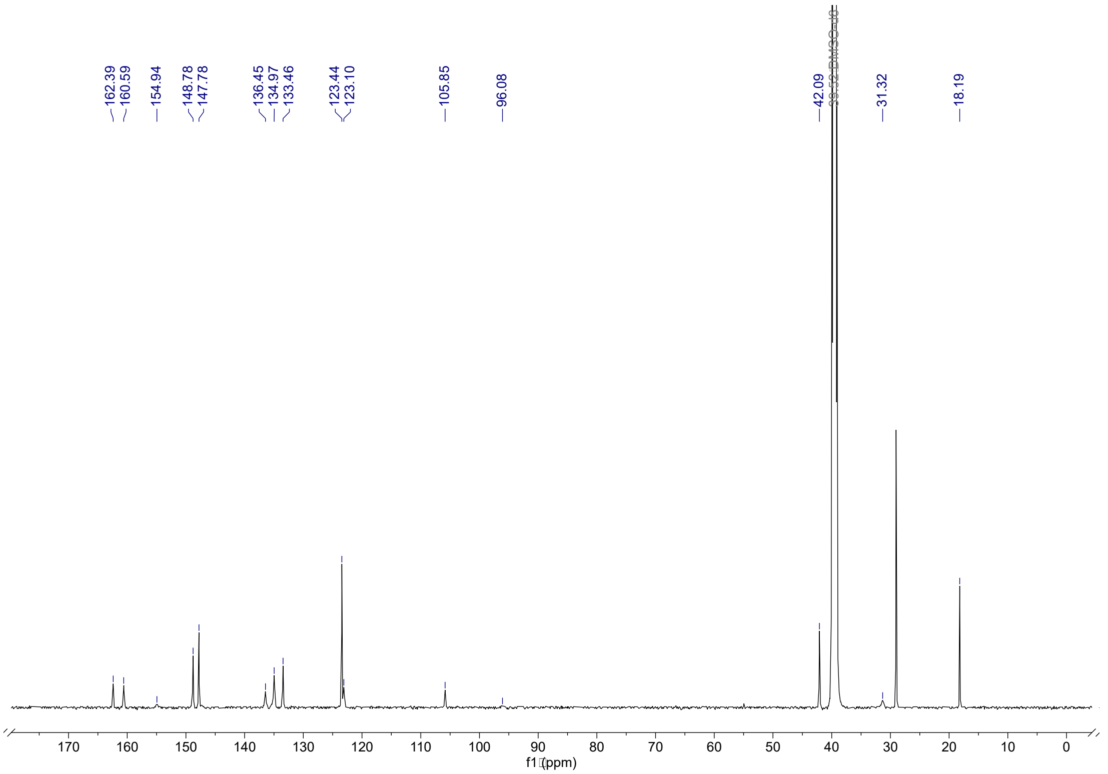

^{1}H NMR (400 MHz, DMSO-d_6) and ^{13}C NMR (101 MHz, DMSO-d_6) of compound 26

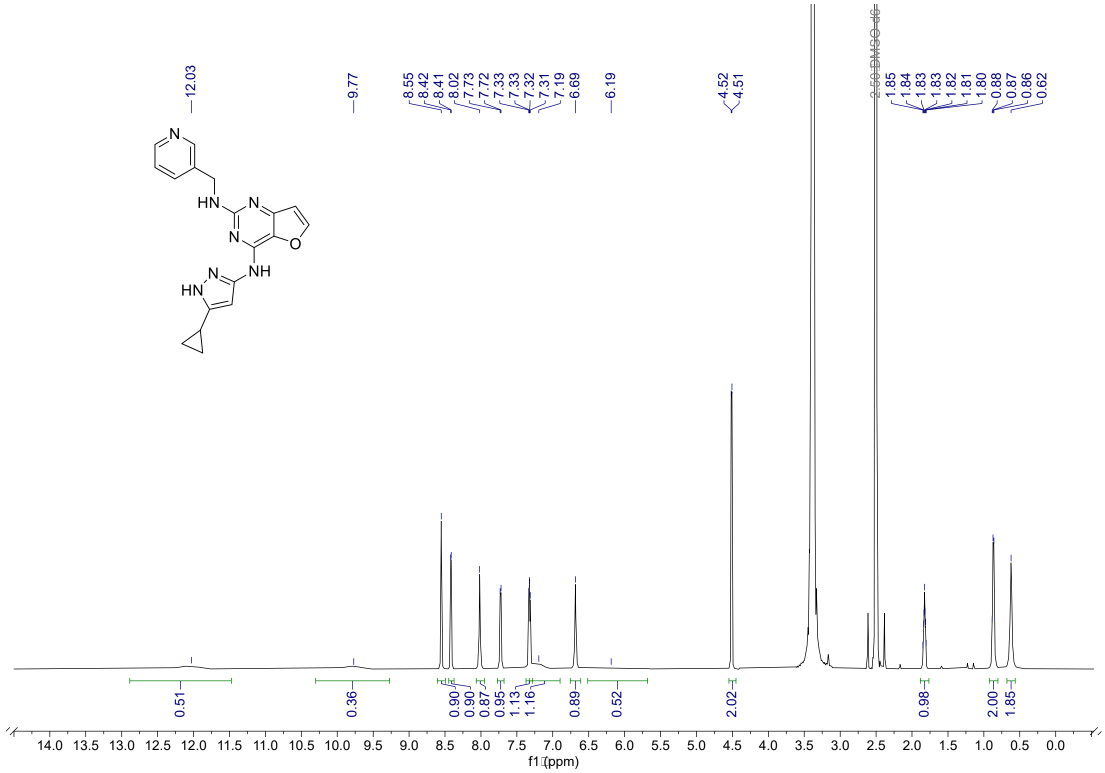

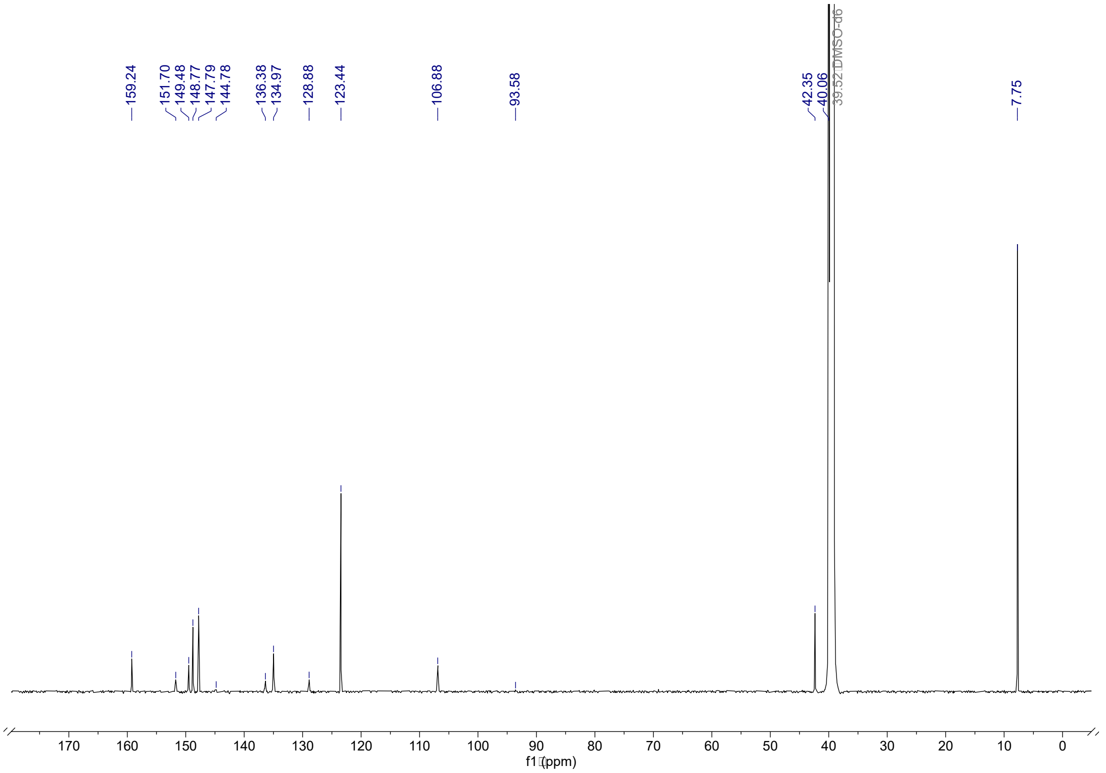

^{1}H NMR (400 MHz, DMSO-d_6) and ^{13}C NMR (101 MHz, DMSO-d_6) of compound 27

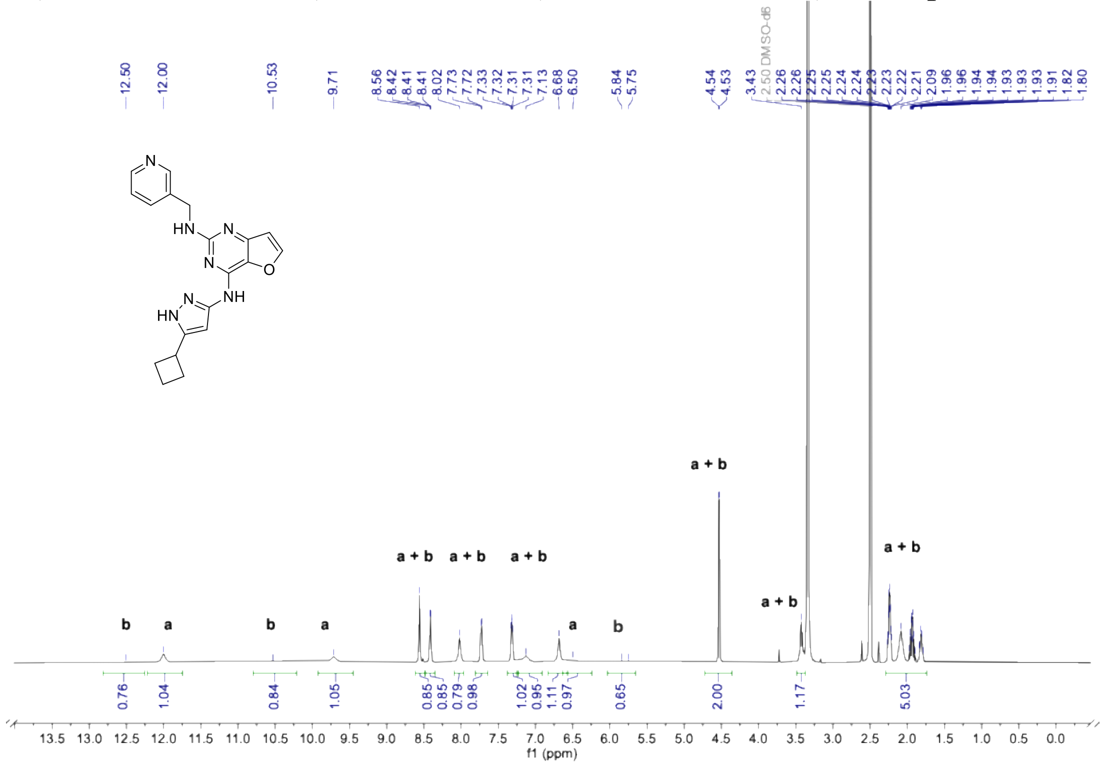

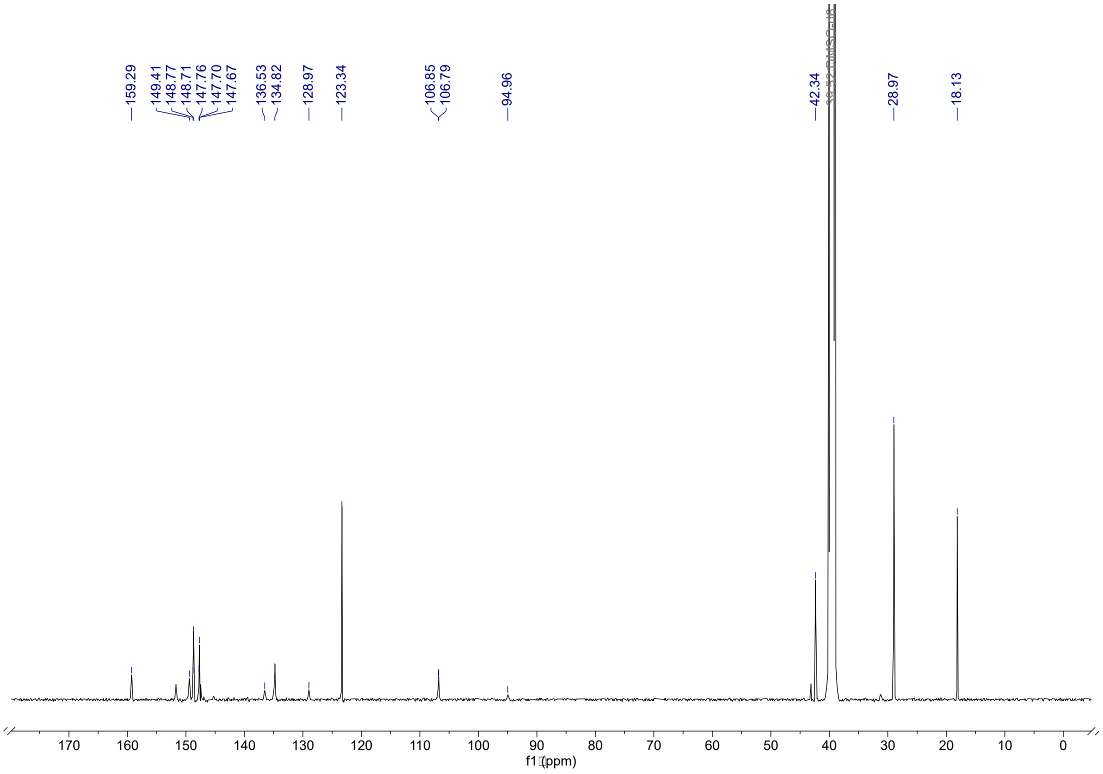

^{1}H NMR (400 MHz, DMSO-d_6) and ^{13}C NMR (101 MHz, DMSO-d_6) of compound 28 (ARN25699)

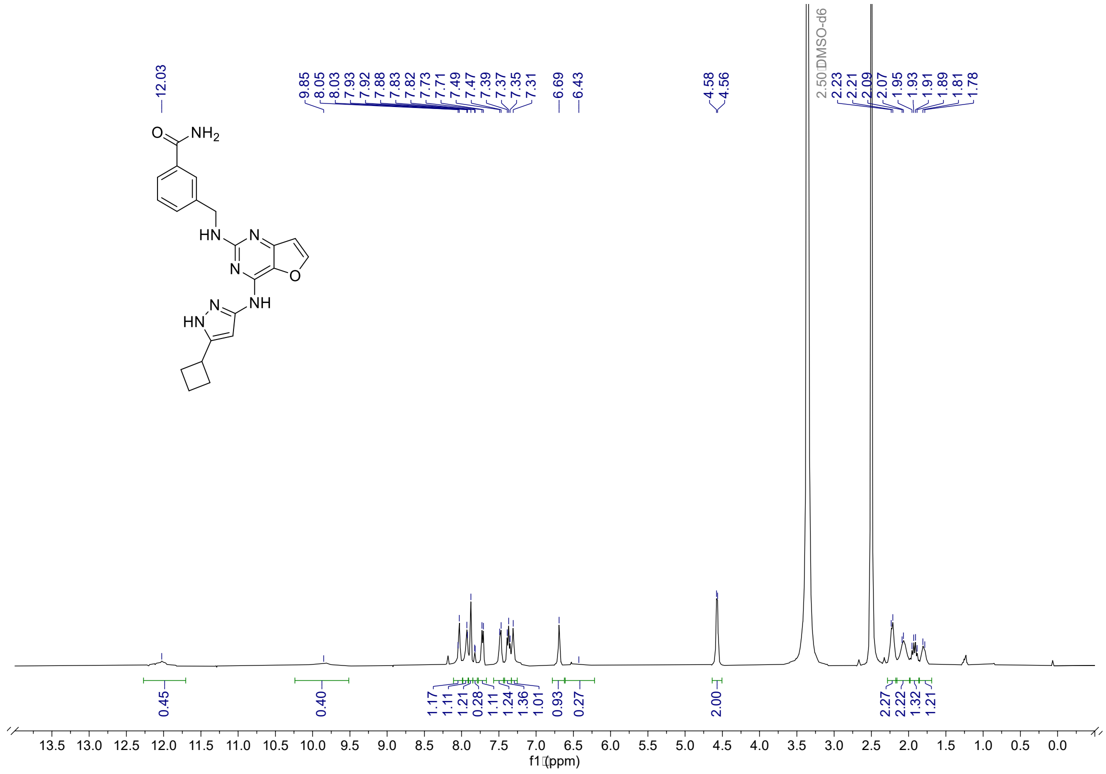

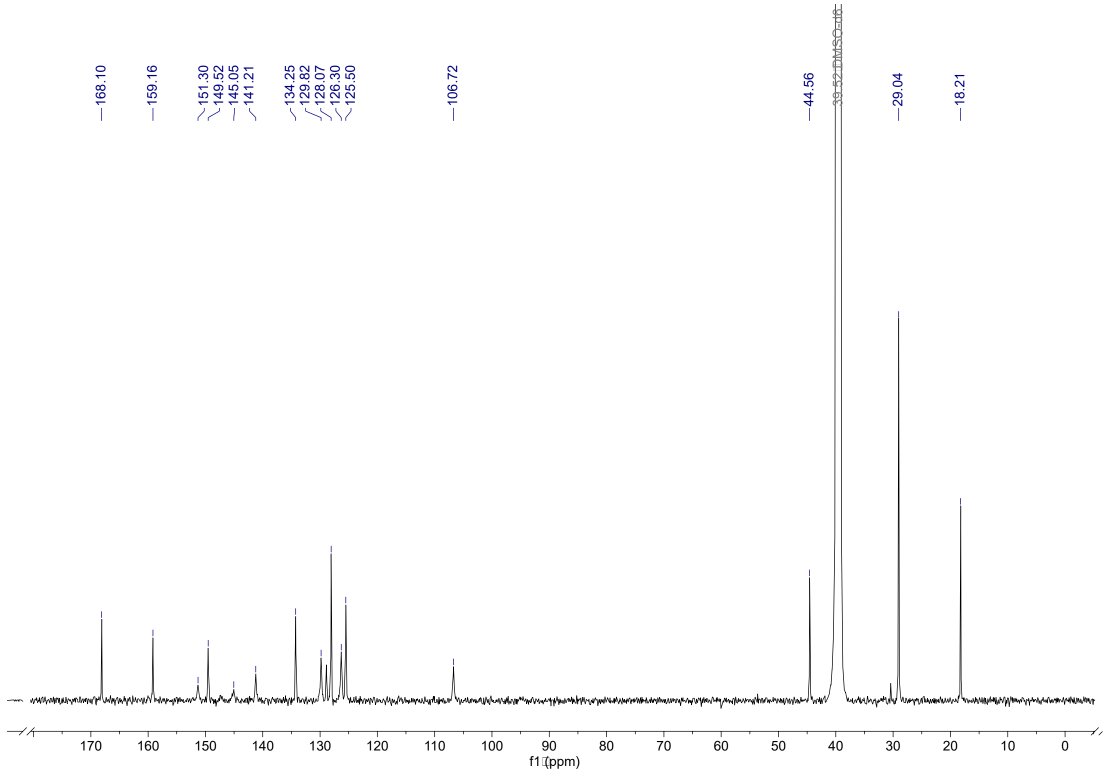

^{1}H NMR (400 MHz, DMSO-d_6) and ^{13}C NMR (101 MHz, DMSO-d_6) of compound 31 (ARN26646)

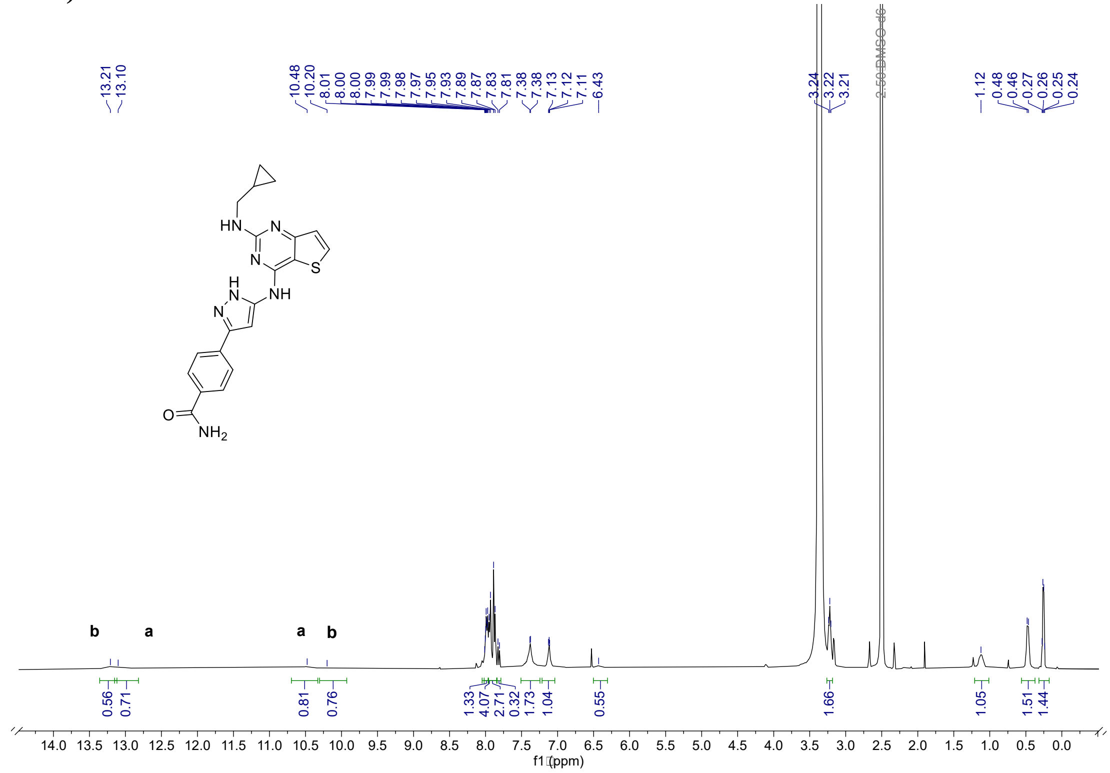

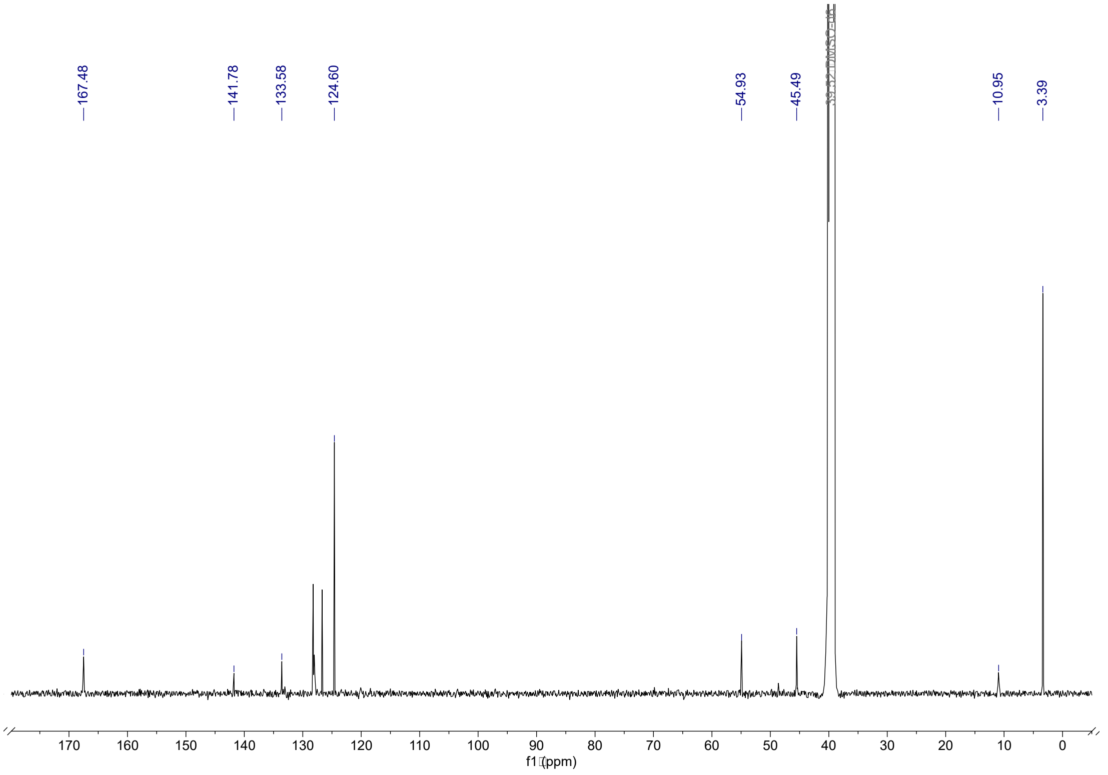

^{1}H NMR (400 MHz, DMSO-d_6) of compound 35

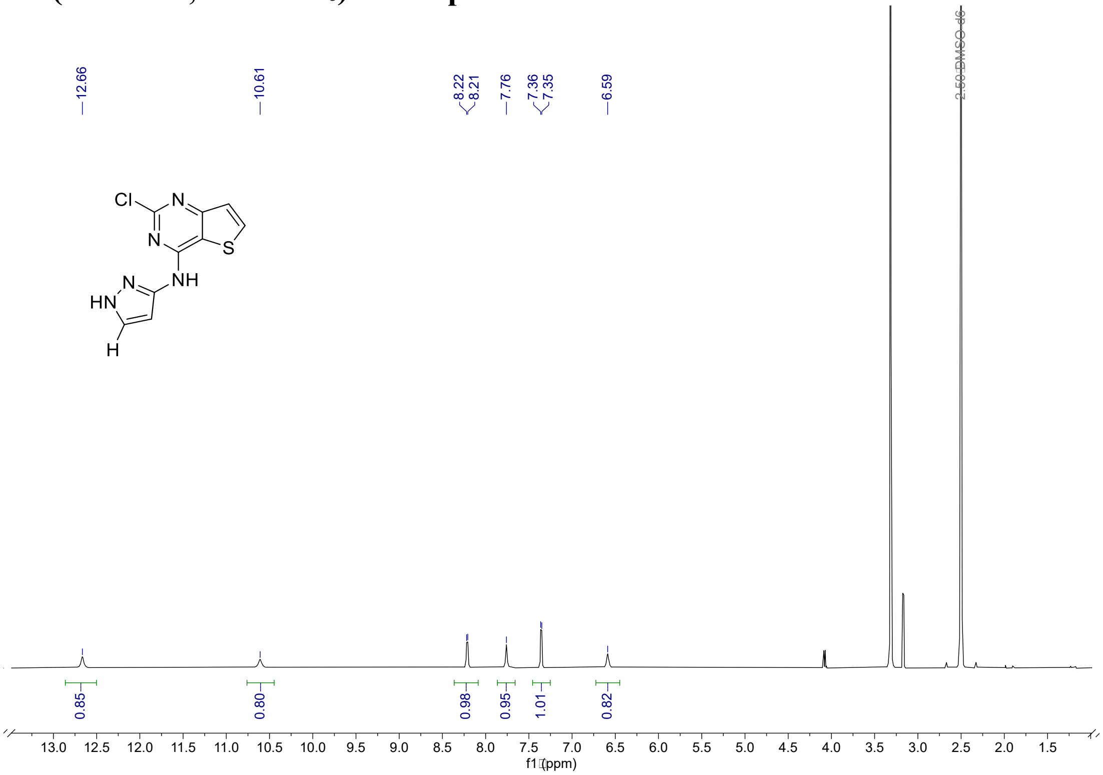

^{1}H NMR (400 MHz, DMSO-d_6) of compound 36

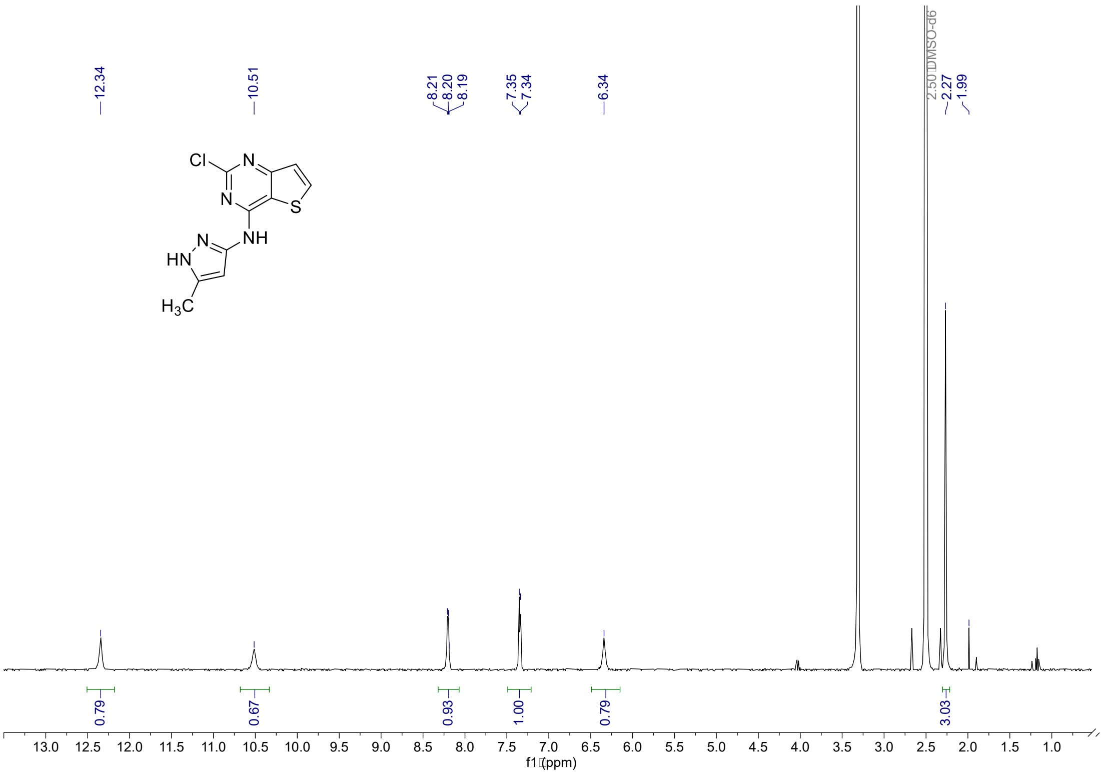

^{1}H NMR (400 MHz, DMSO-d_6) of compound 37

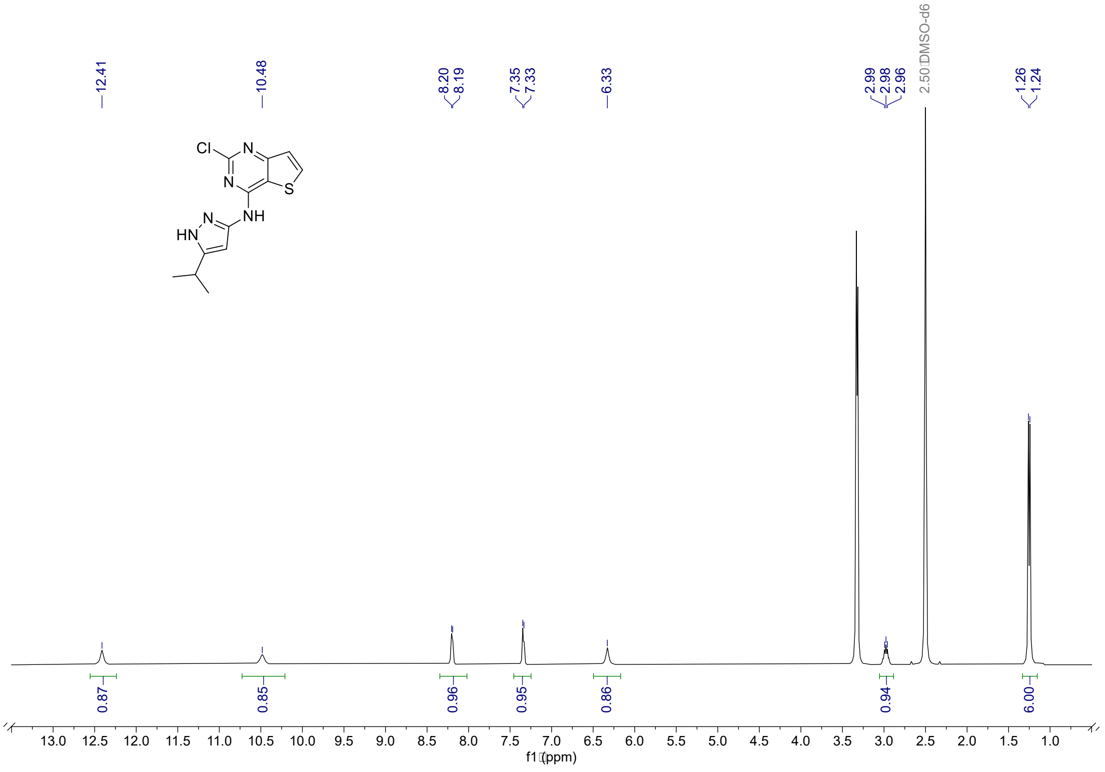

^{1}H NMR (400 MHz, DMSO-d_6) 38

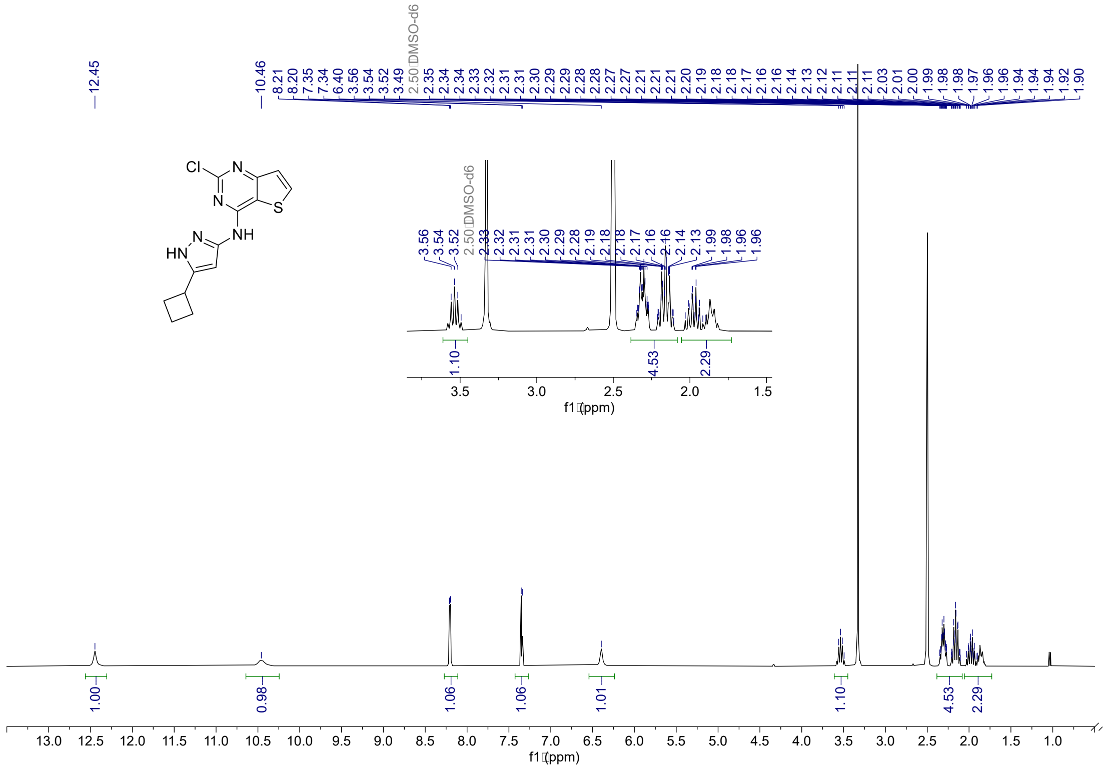

^{1}H NMR (400 MHz, DMSO-d_6) of compound 39

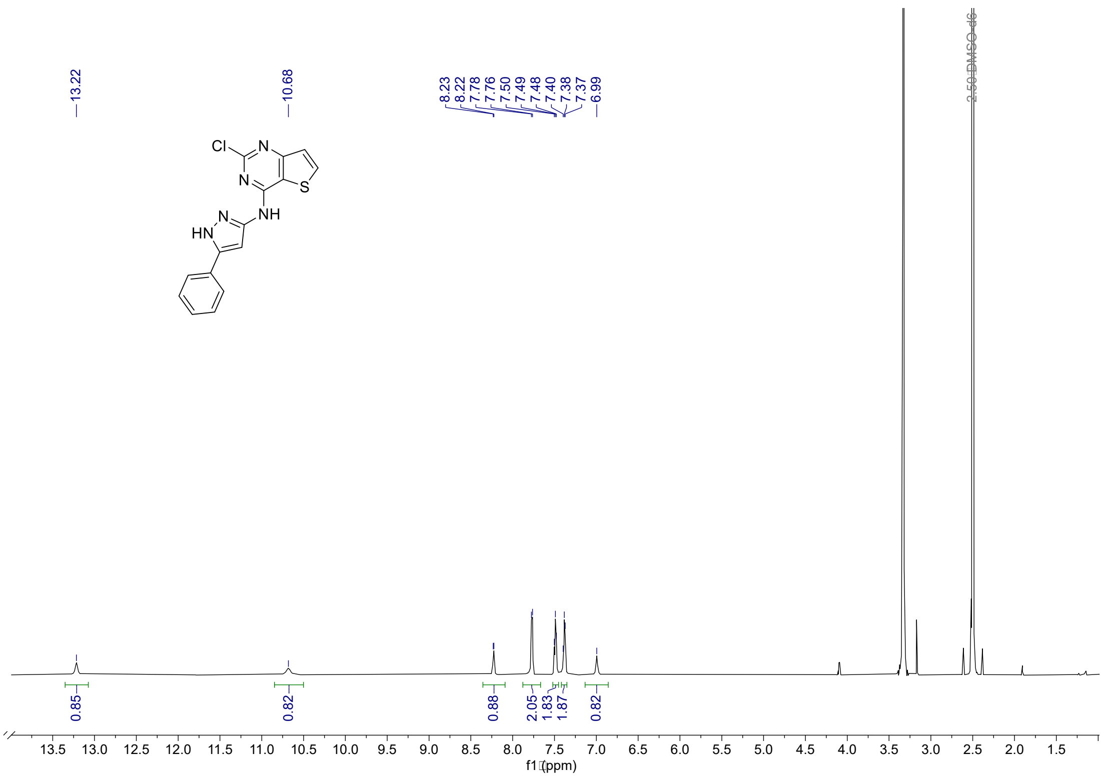

^{1}H NMR (400 MHz, DMSO-d_6) of compound 40

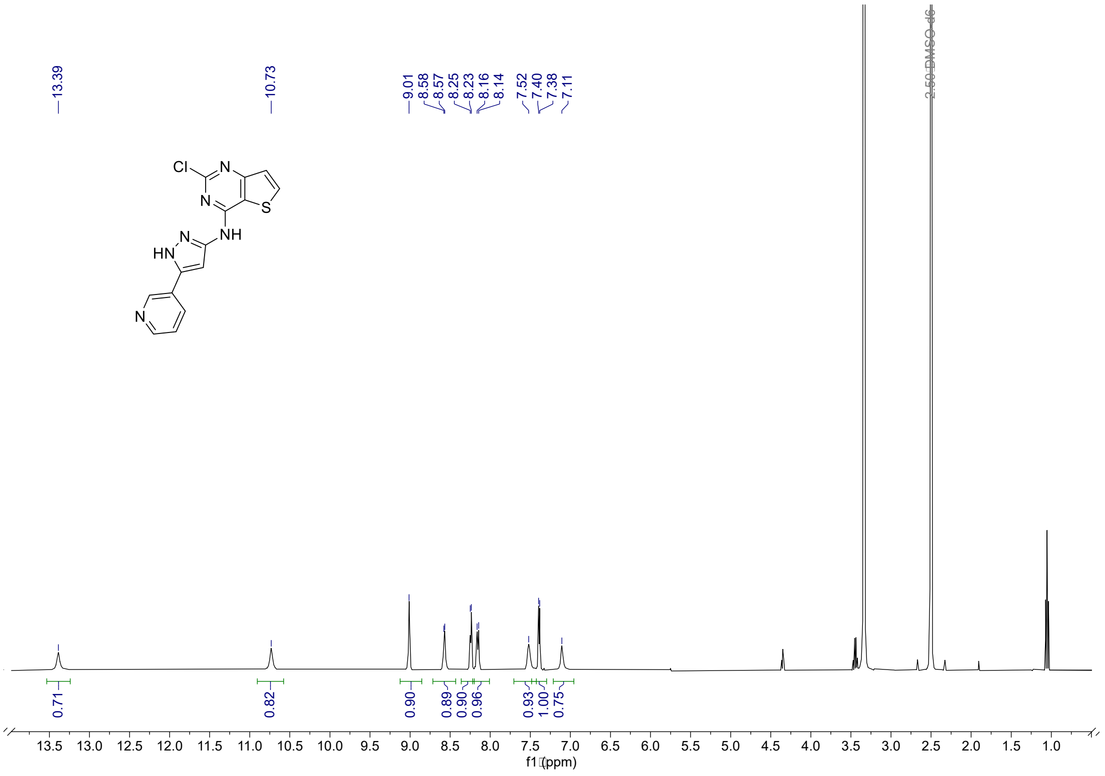

^{1}H NMR (400 MHz, DMSO-d_6) of compound 41

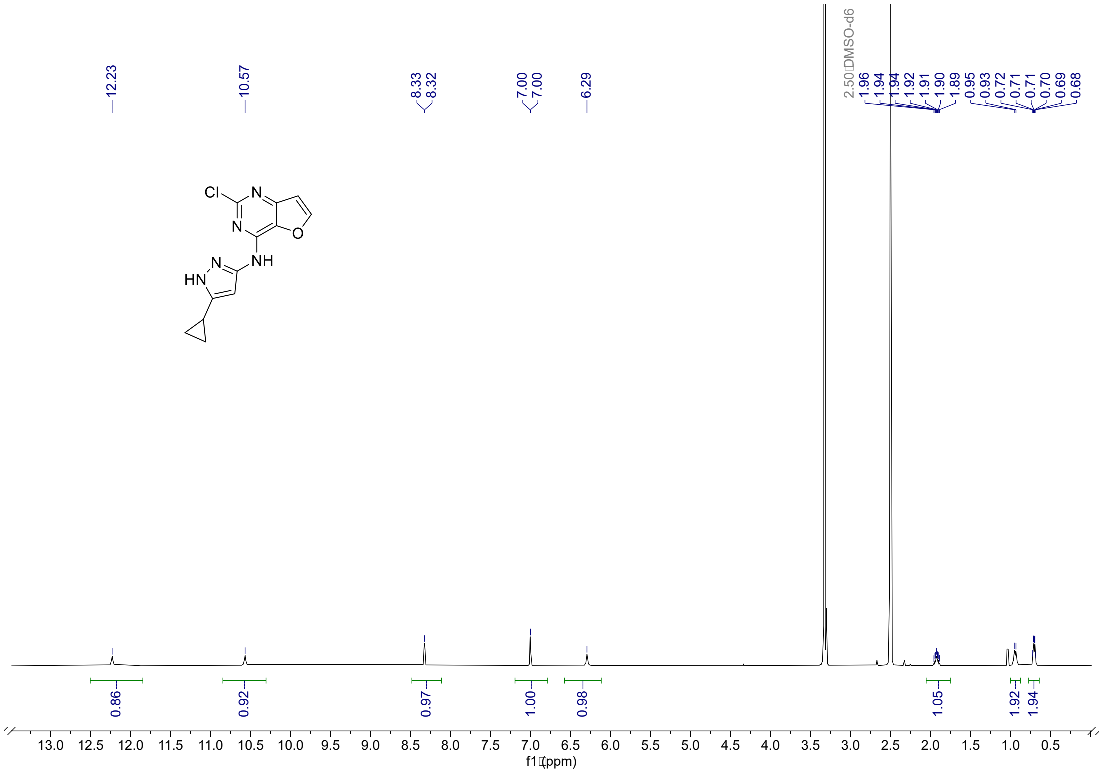

^{1}H NMR (400 MHz, DMSO-d_6) of compound 42

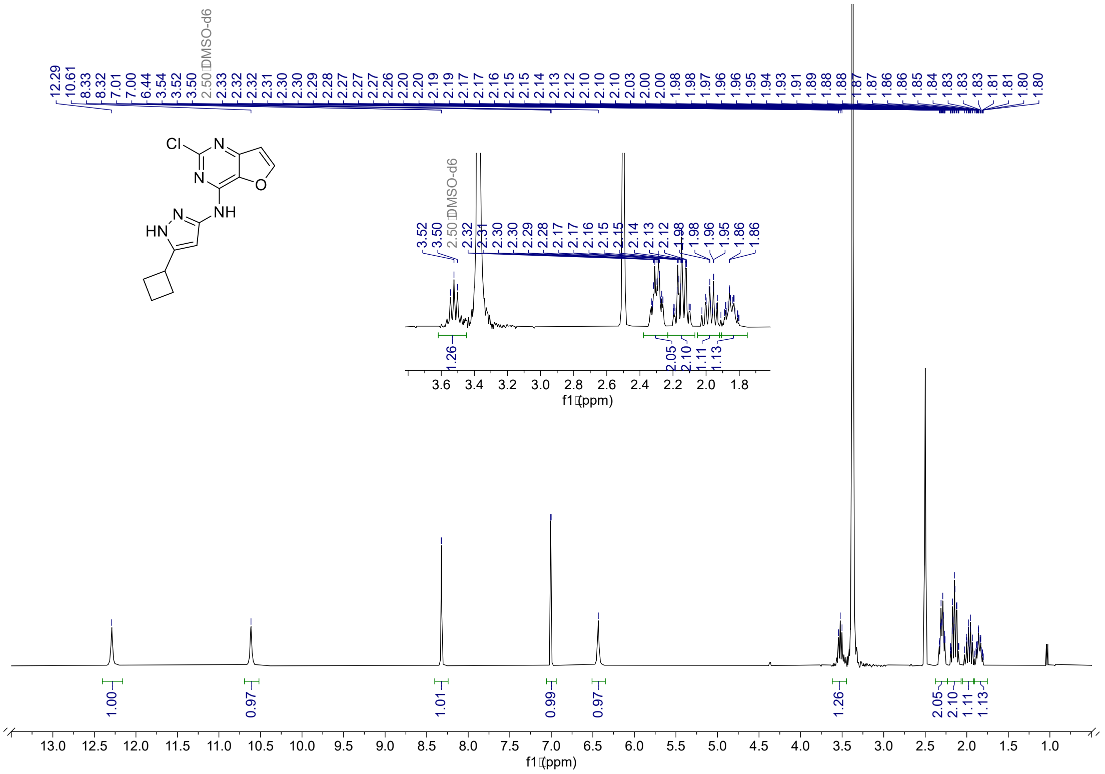

^{1}H NMR (400 MHz, DMSO-d_6) of compound 43

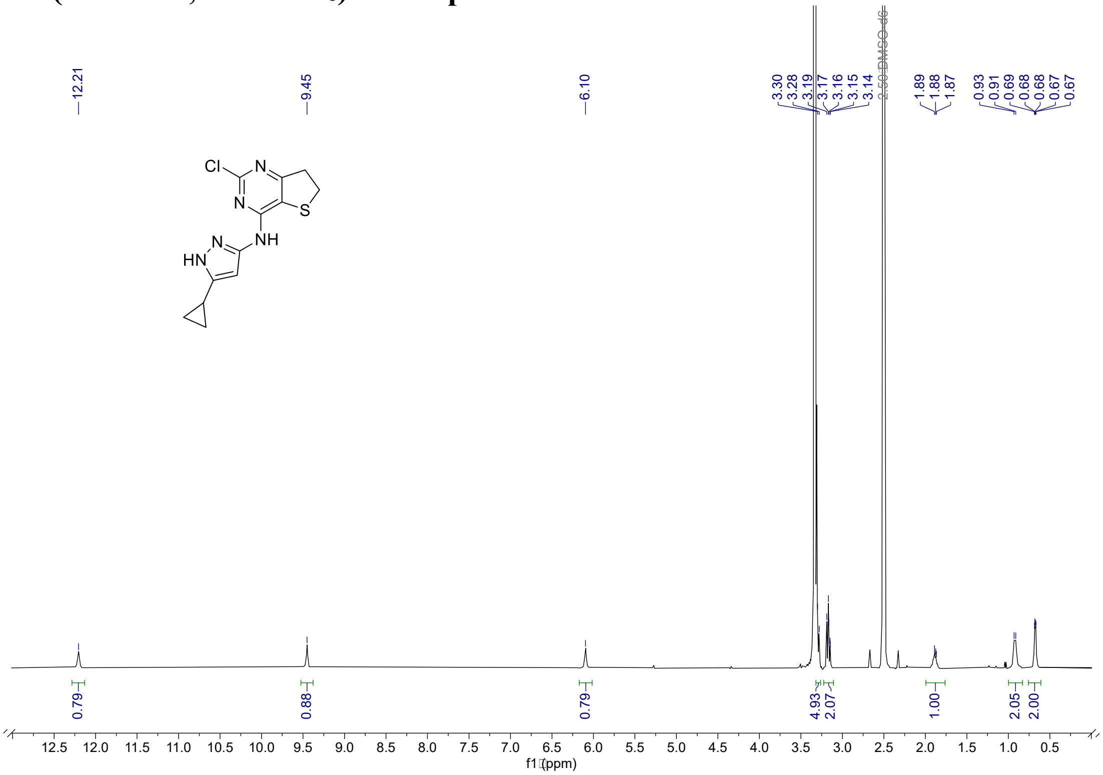

^{1}H NMR (400 MHz, DMSO-d_6) of compound 44

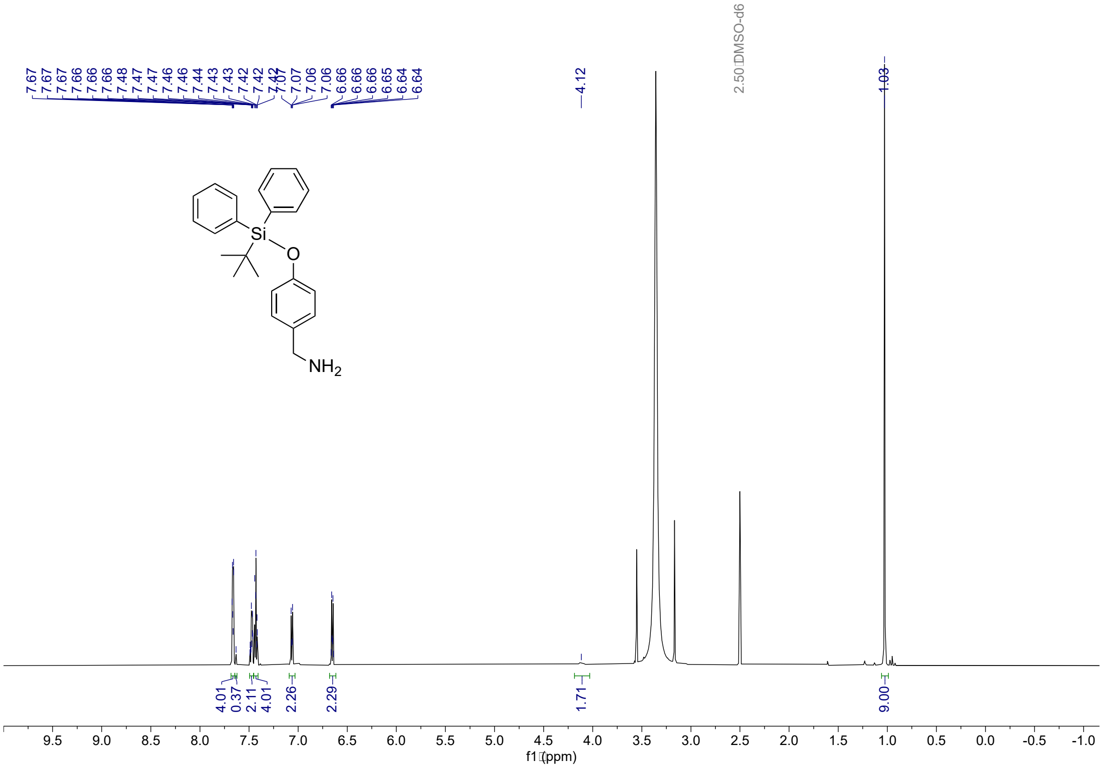

^{1}H NMR (400 MHz, DMSO-d_6) of compound 45

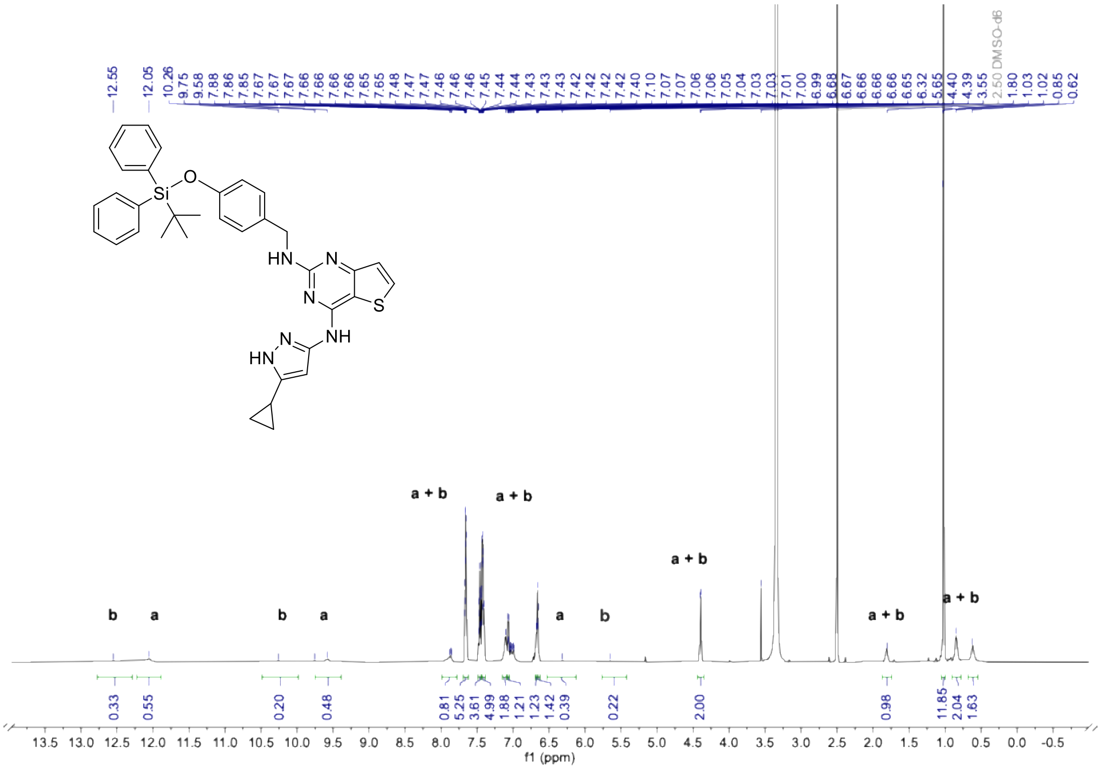

^{1}H NMR (400 MHz, DMSO-d_6) of compound 46

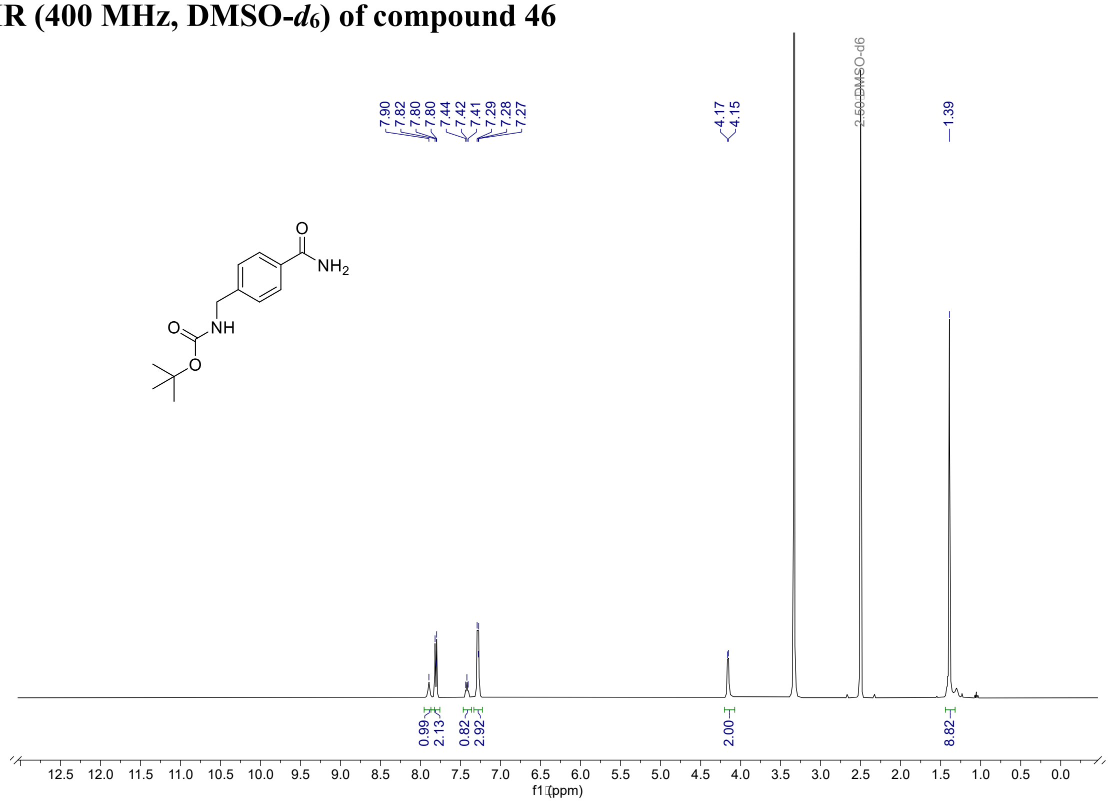

^{1}H NMR (400 MHz, DMSO-d_6) of compound 47

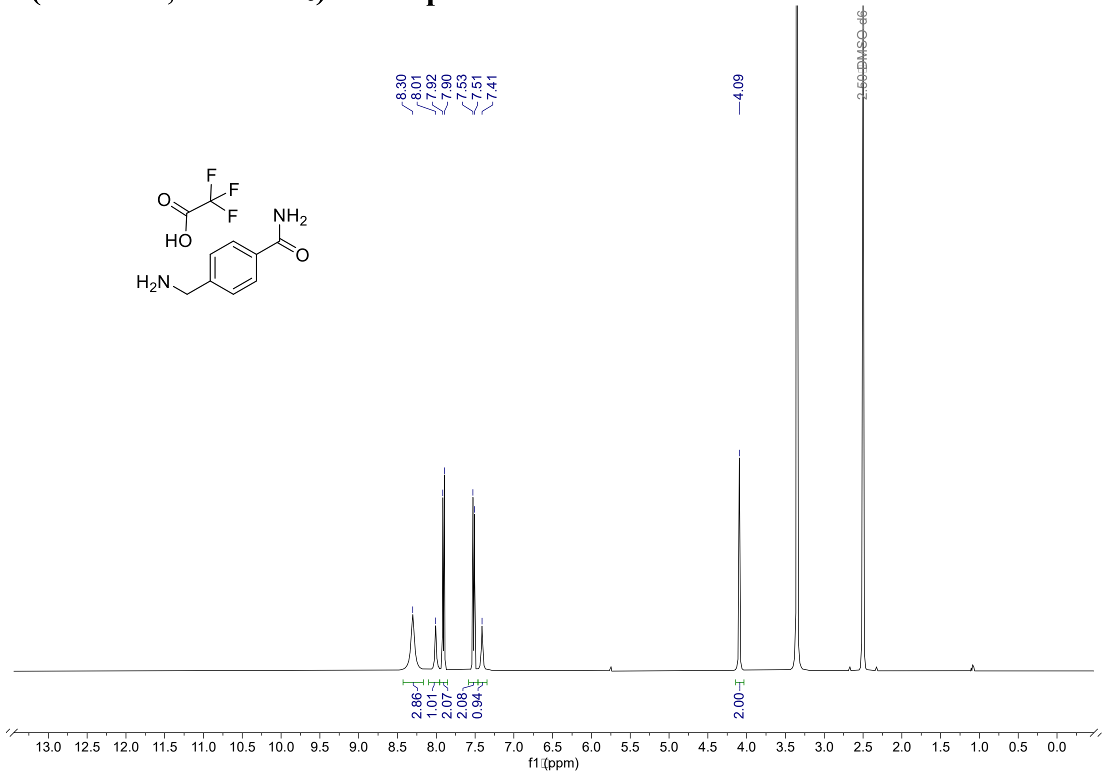

^{1}H NMR (400 MHz, DMSO-d_6) of compound 48

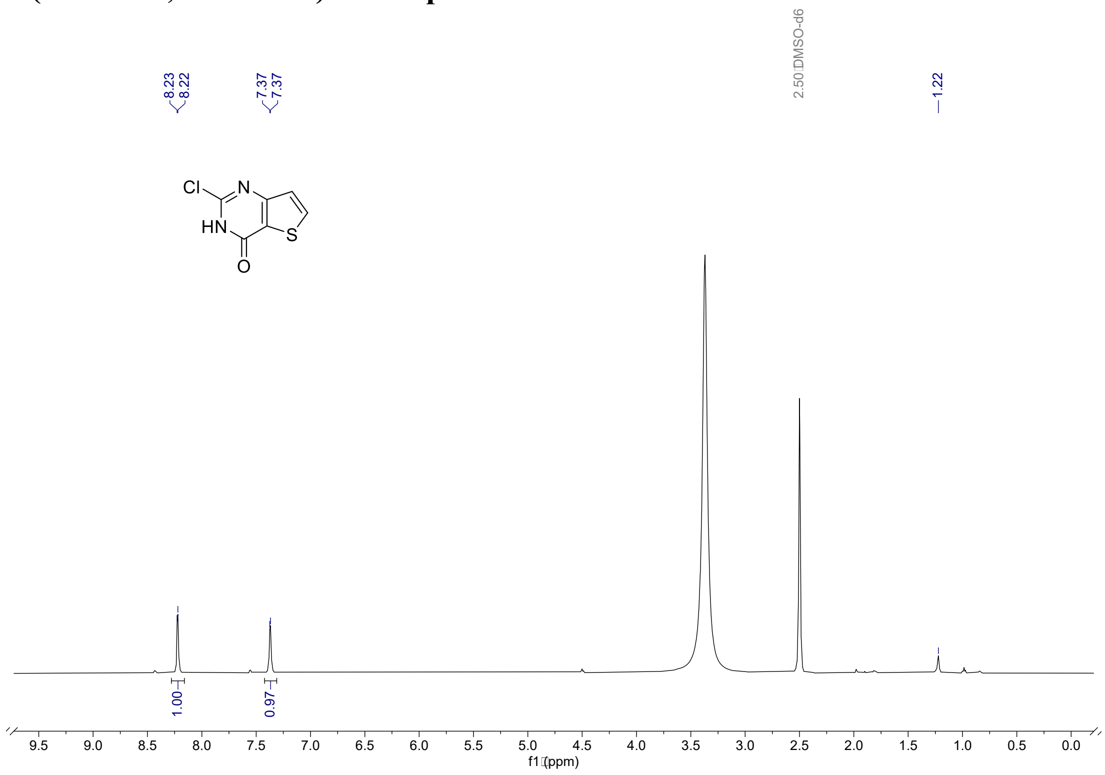

^{1}H NMR (400 MHz, DMSO-d_6) of compound 49

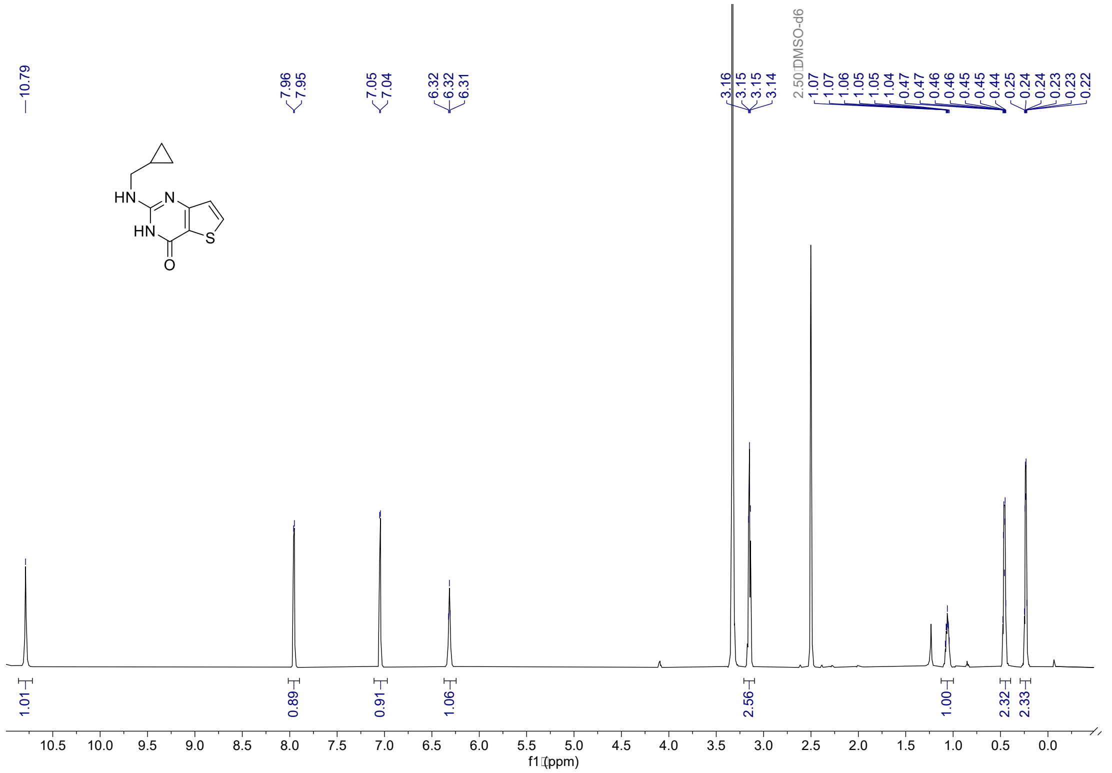

^{1}H NMR (400 MHz, DMSO-d_6) of compound 50

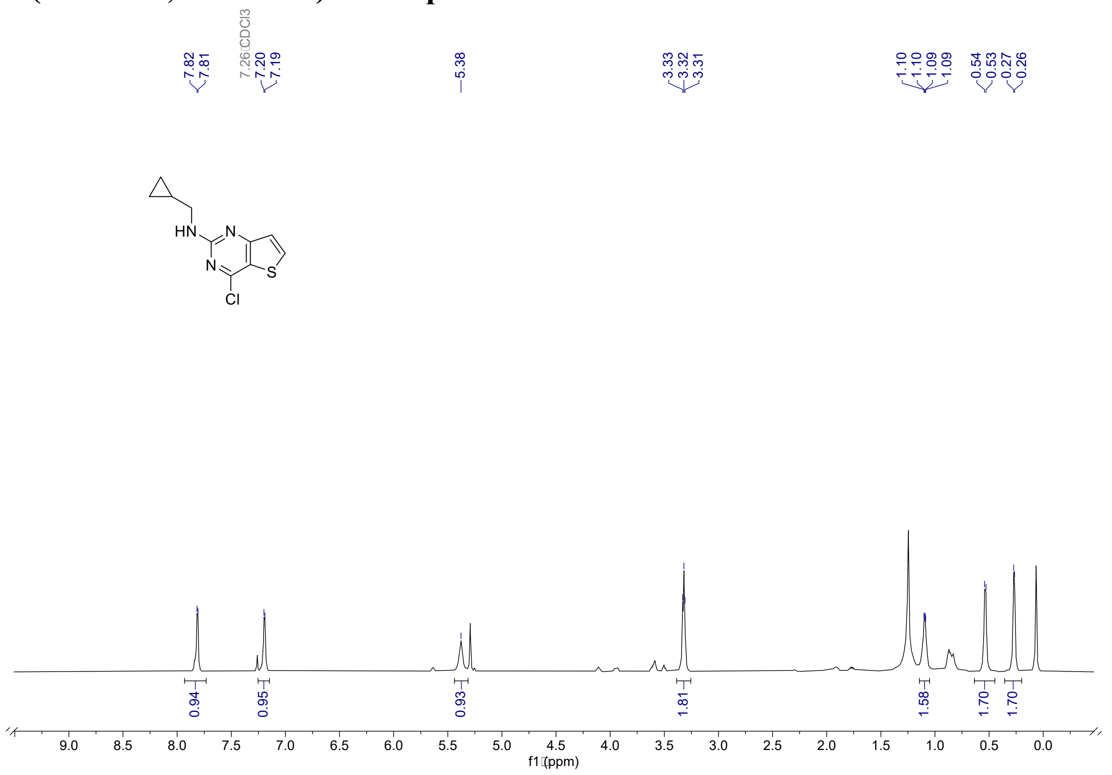

^{1}H NMR (400 MHz, DMSO-d_6) of compound 51

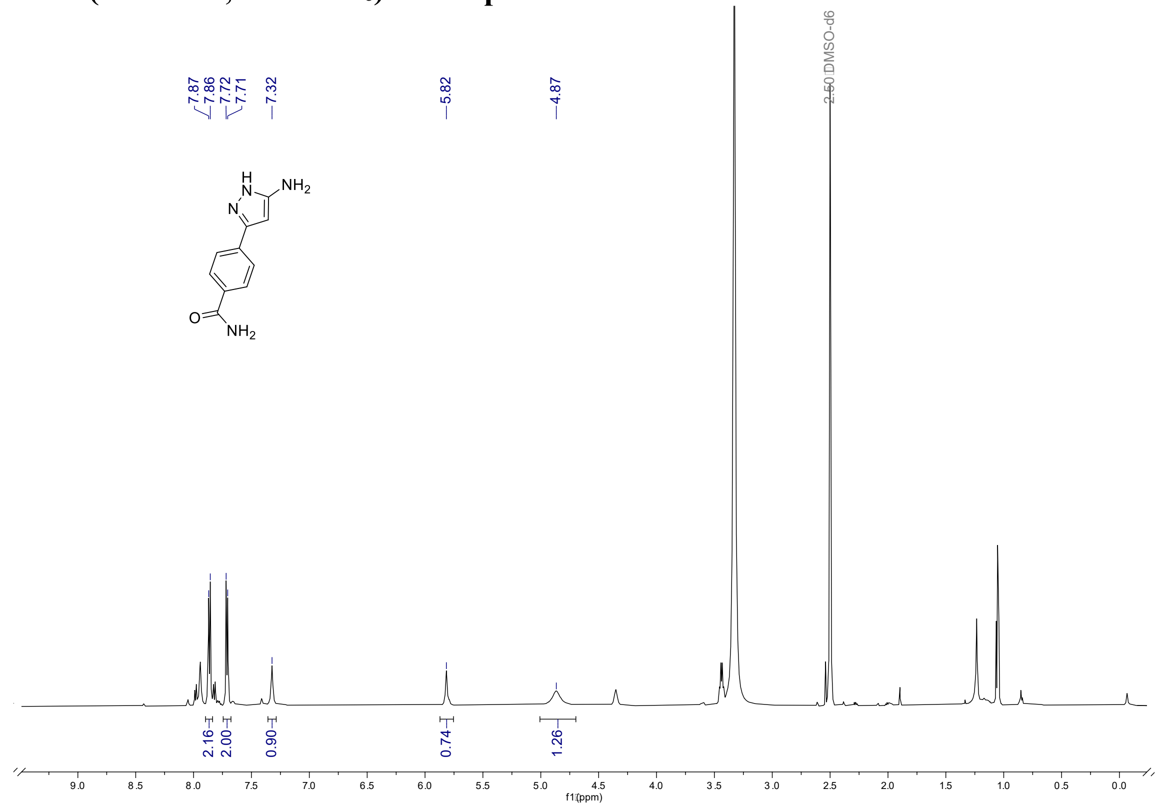

^{1}H NMR (400 MHz, DMSO-d_6) of compound 52

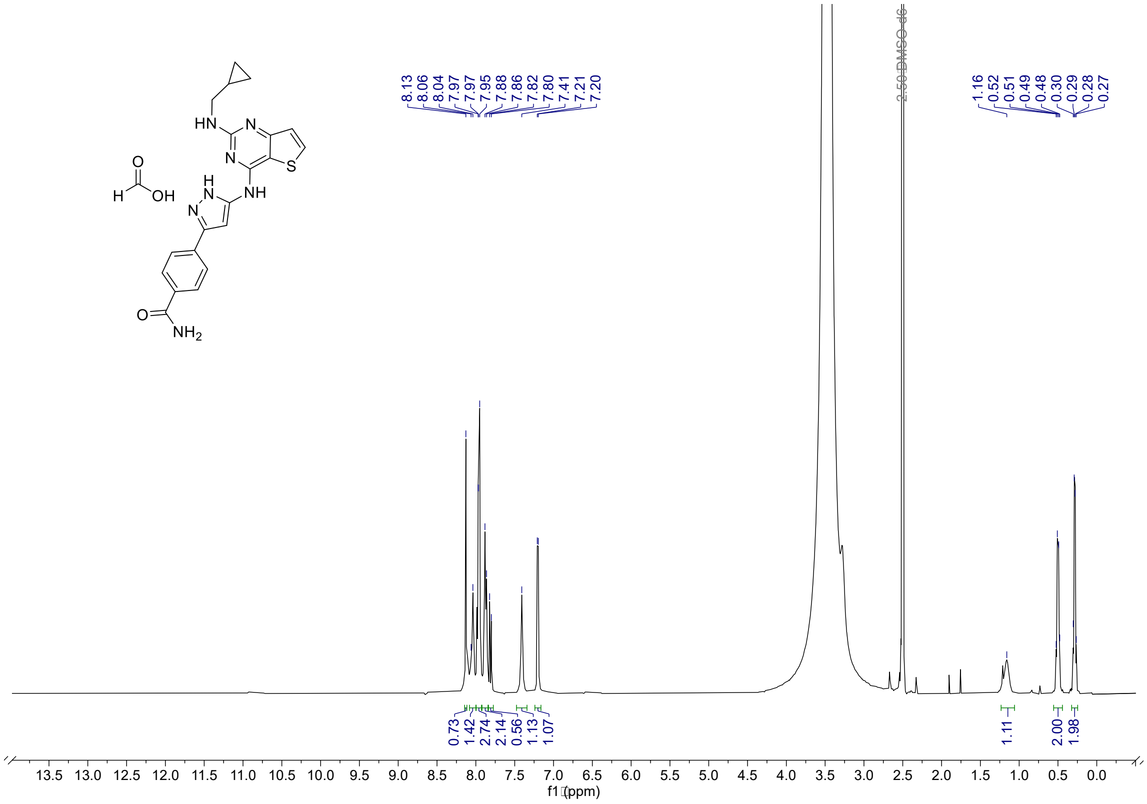

UPLC-MS purity (UV at 215 nm) of compound 2

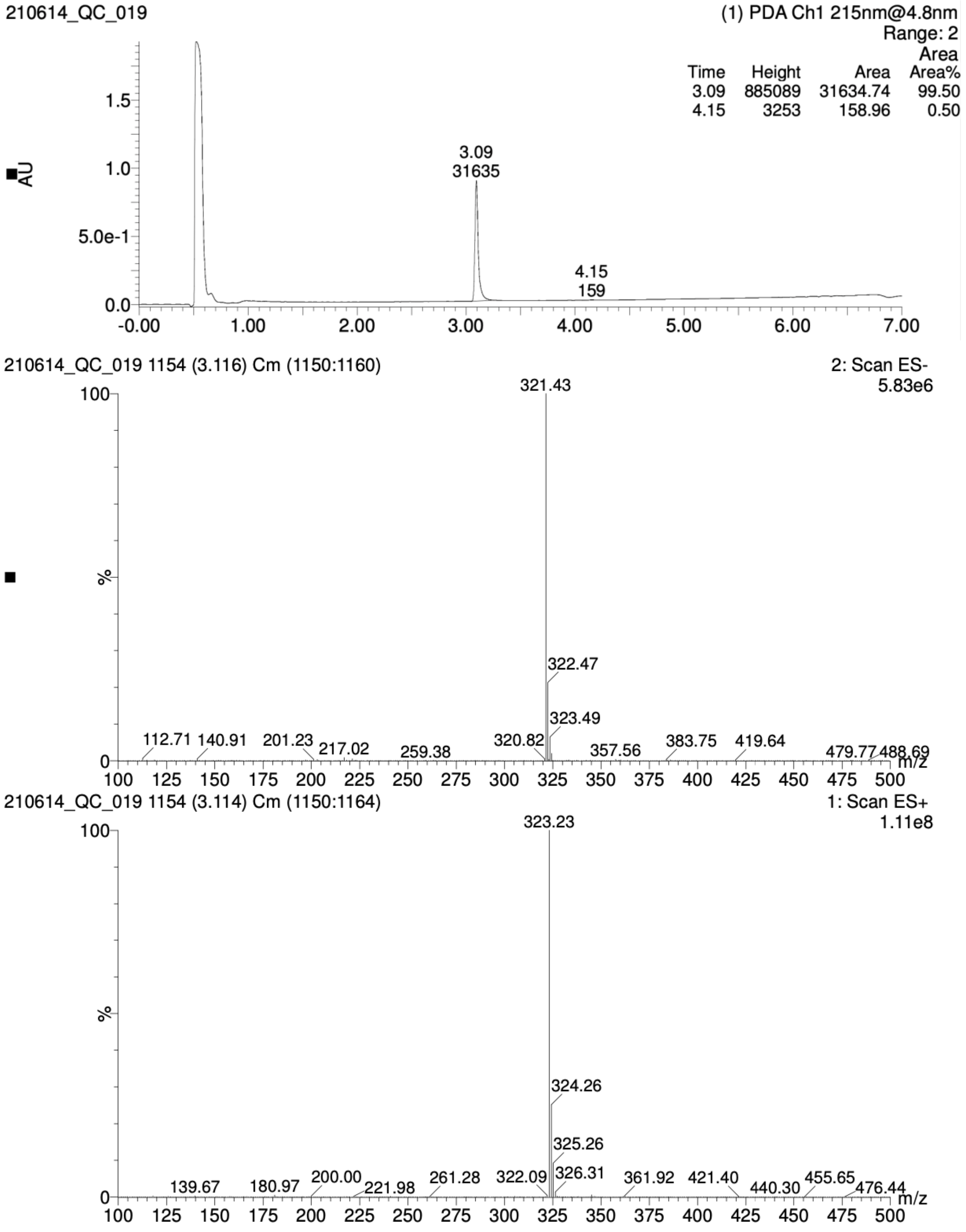

UPLC-MS purity (UV at 215 nm) of compound 3

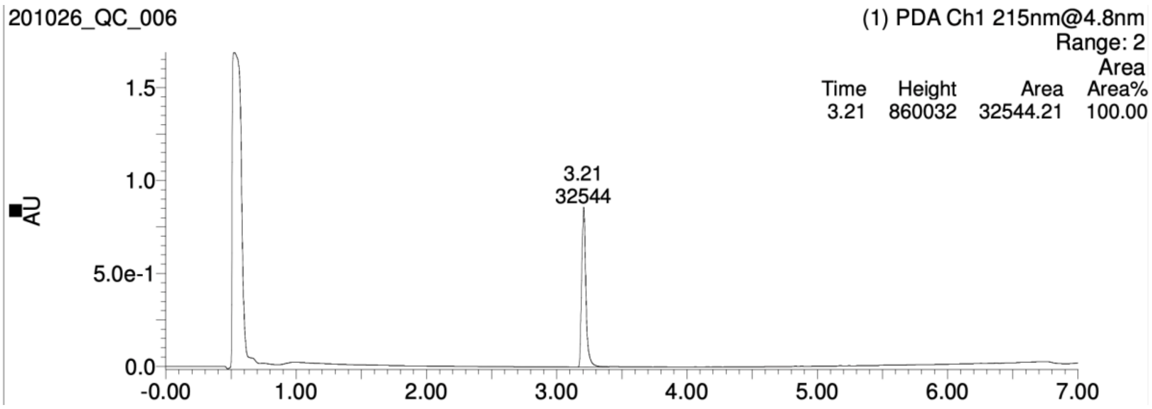

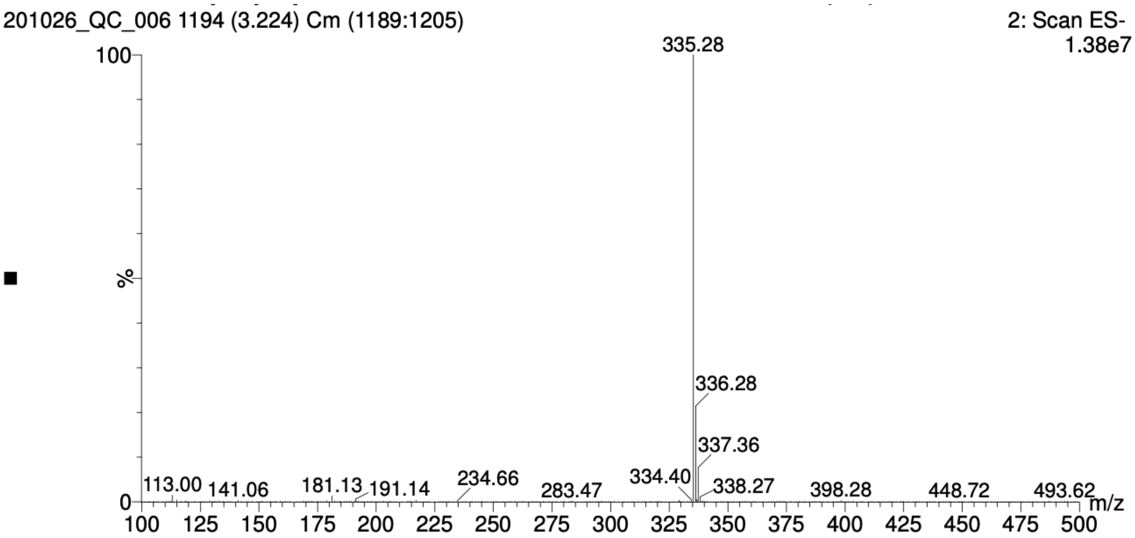

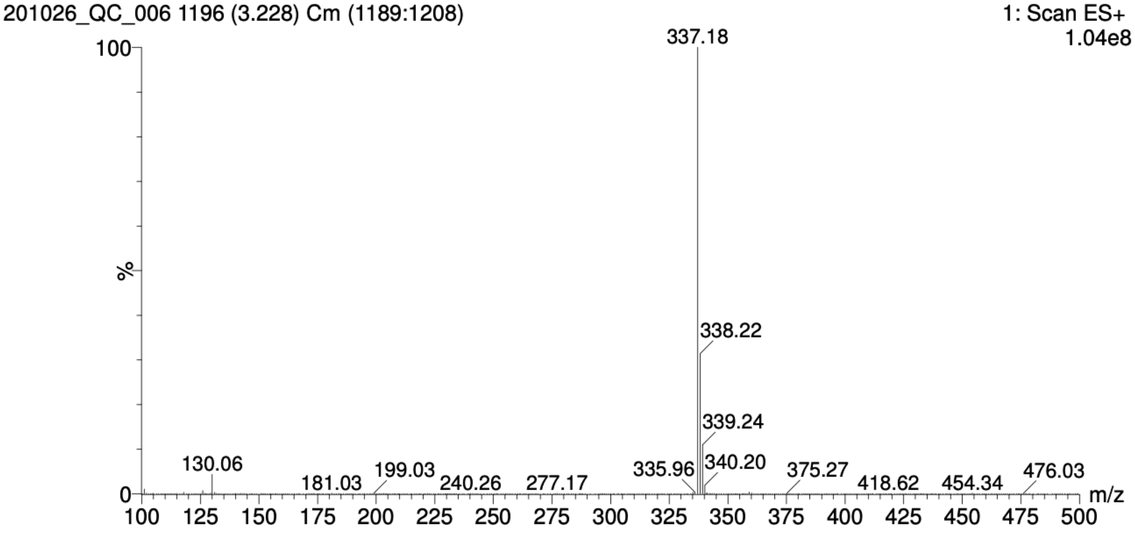

UPLC-MS purity (UV at 215 nm) of compound 4

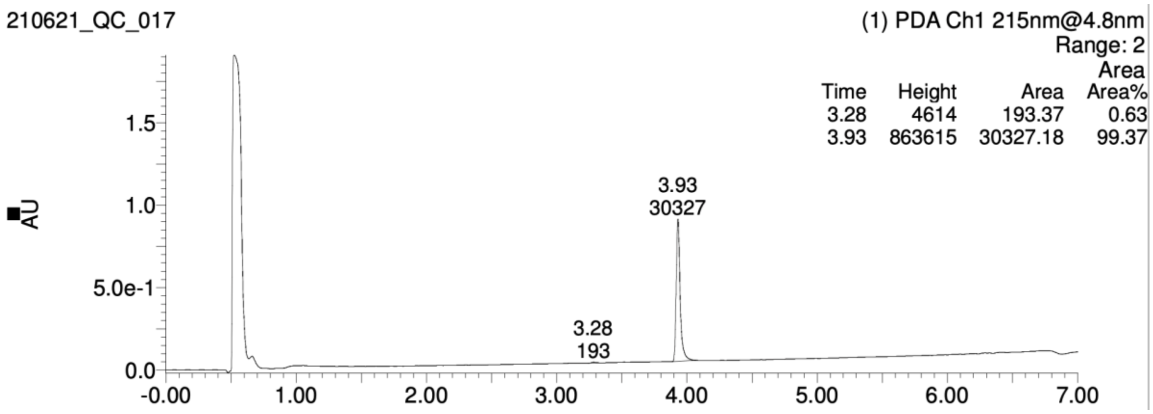

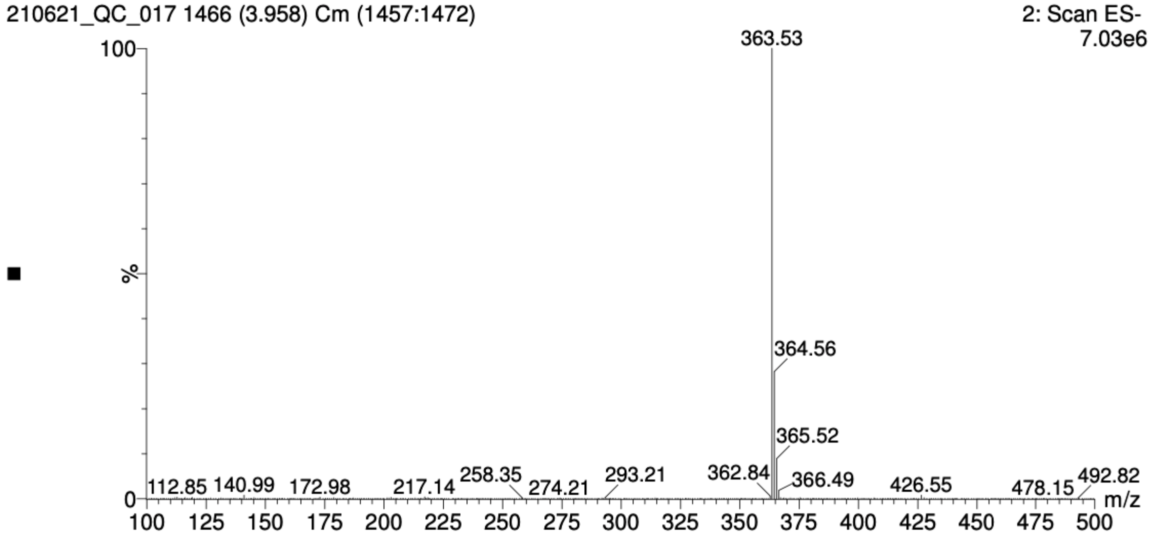

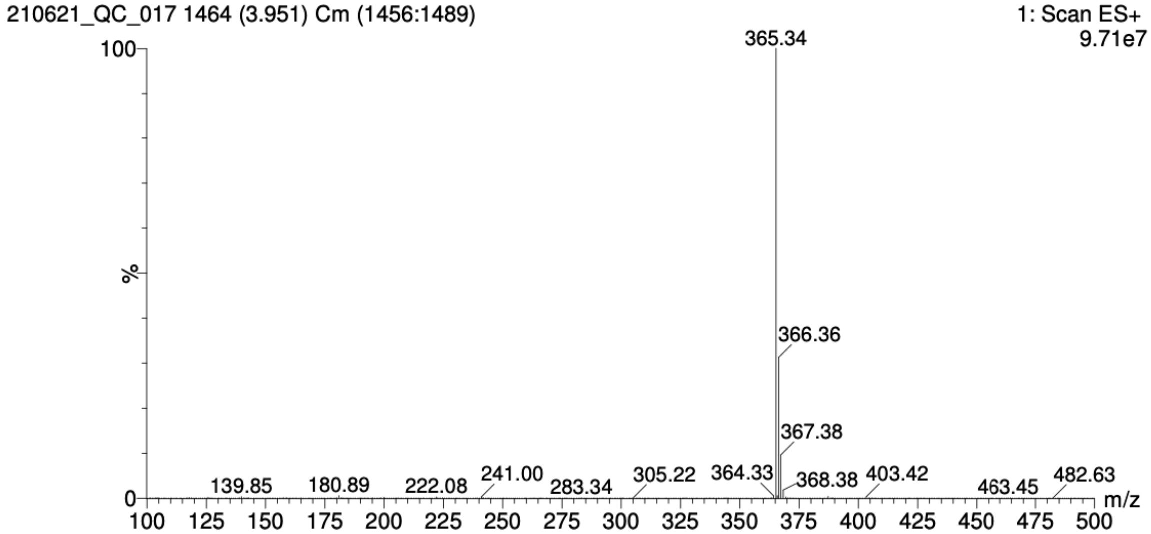

UPLC-MS purity (UV at 215 nm) of compound 5

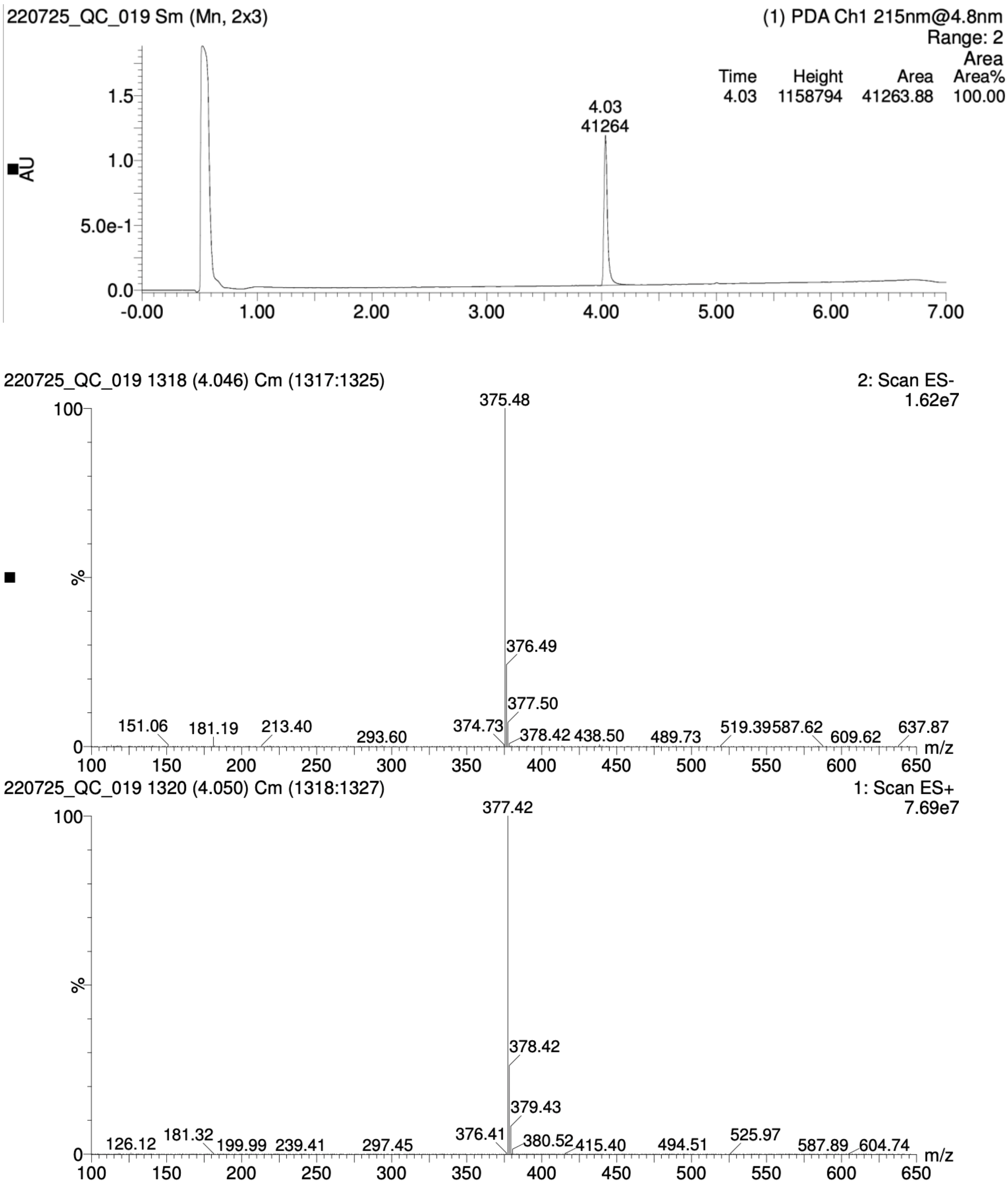

UPLC-MS purity (UV at 215 nm) of compound 6

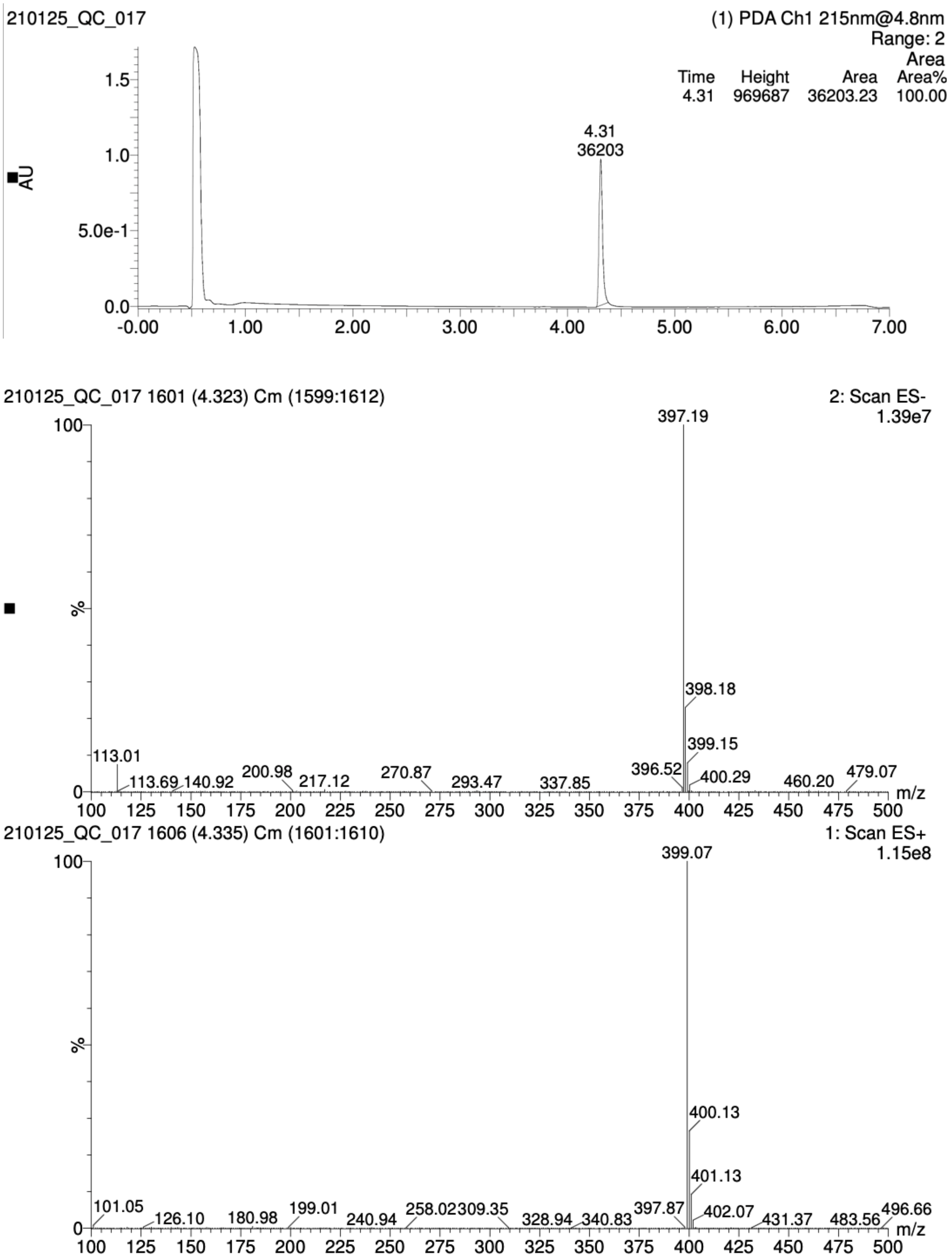

UPLC-MS purity (UV at 215 nm) of compound 7

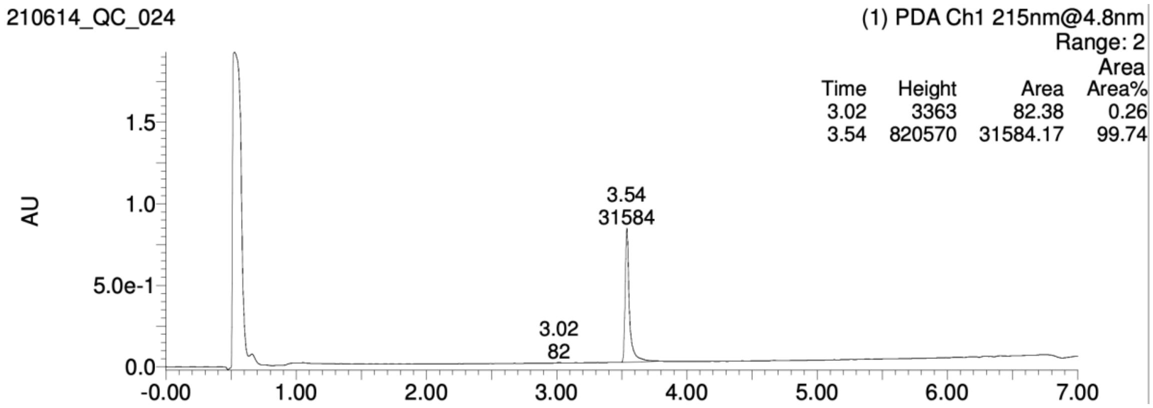

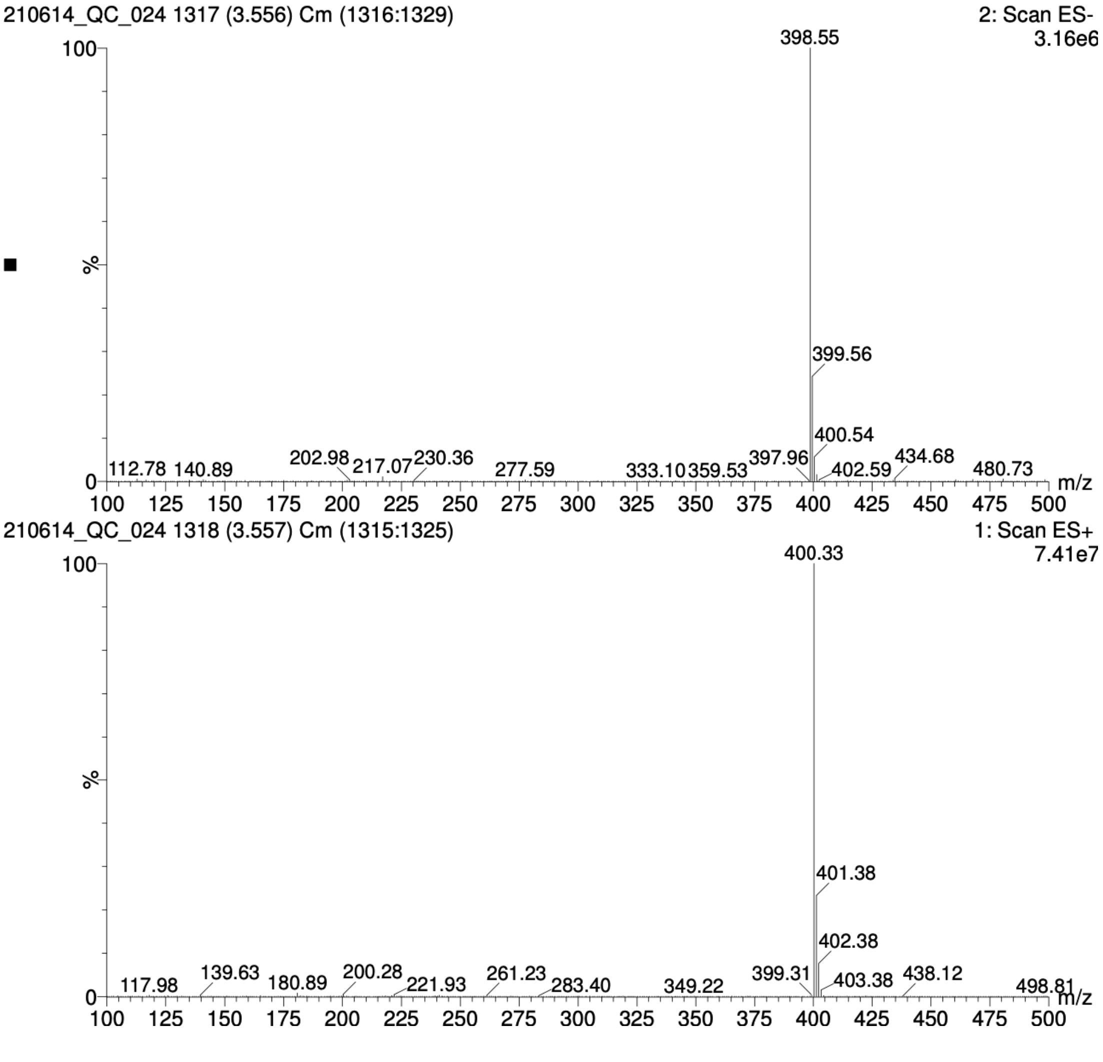

UPLC-MS purity (UV at 215 nm) of compound 8

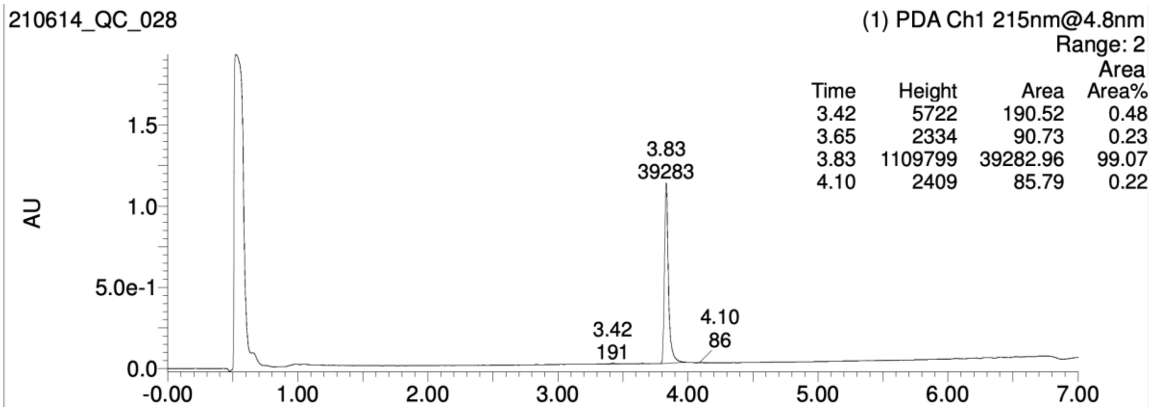

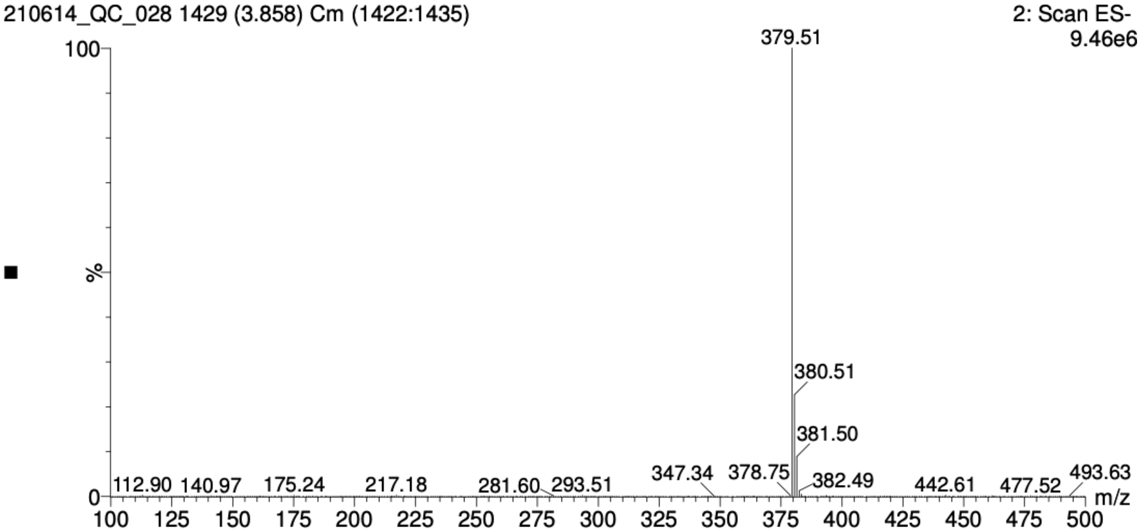

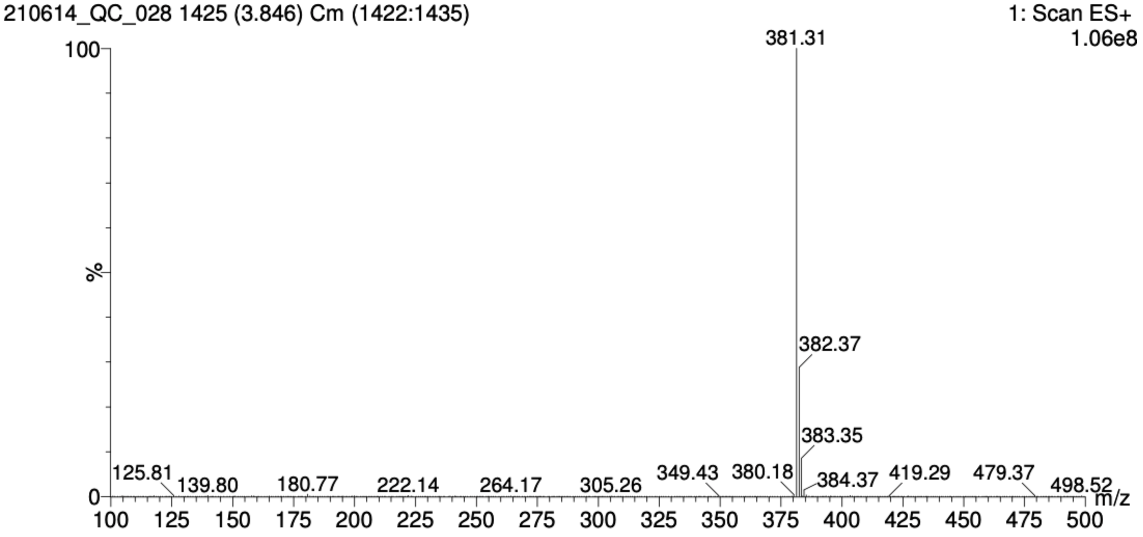

UPLC-MS purity (UV at 215 nm) of compound 9

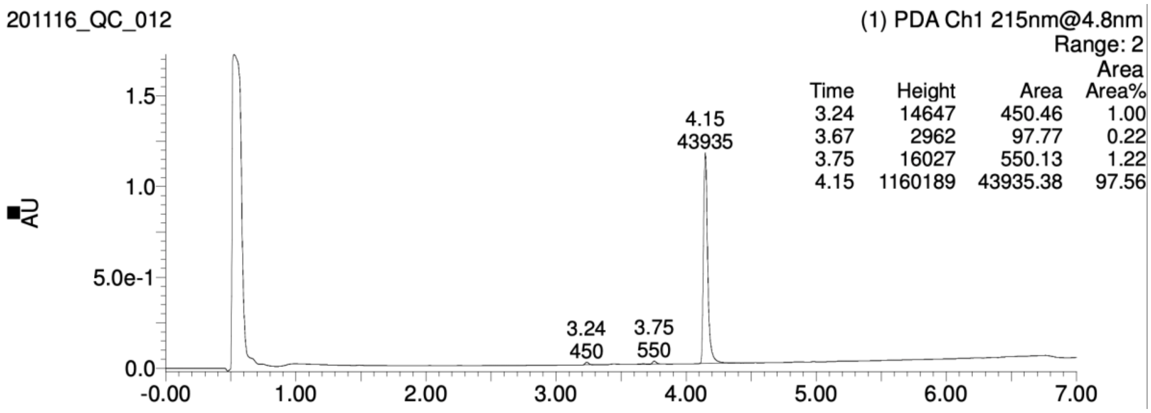

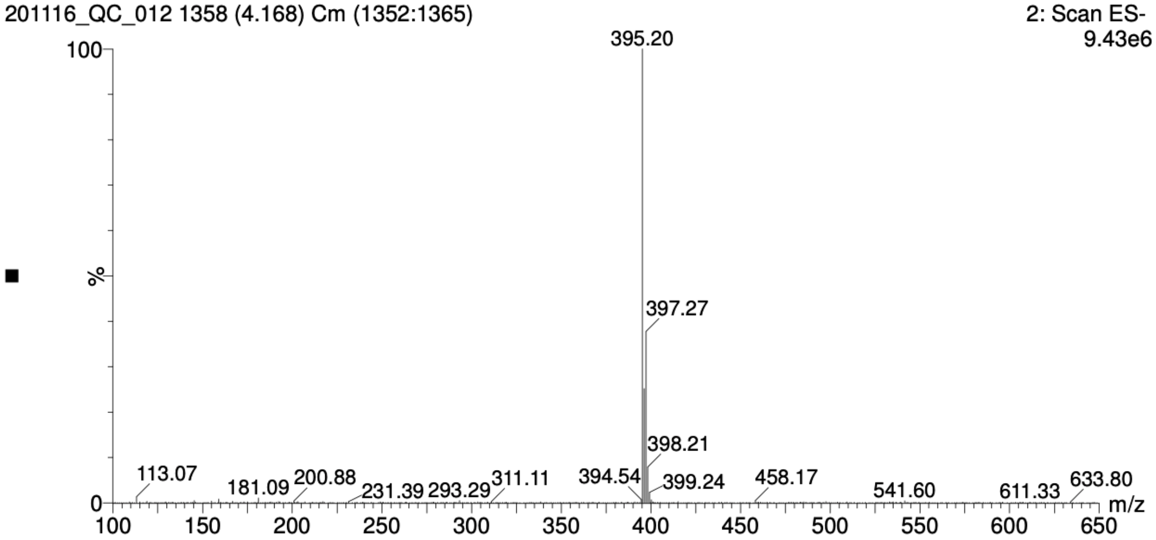

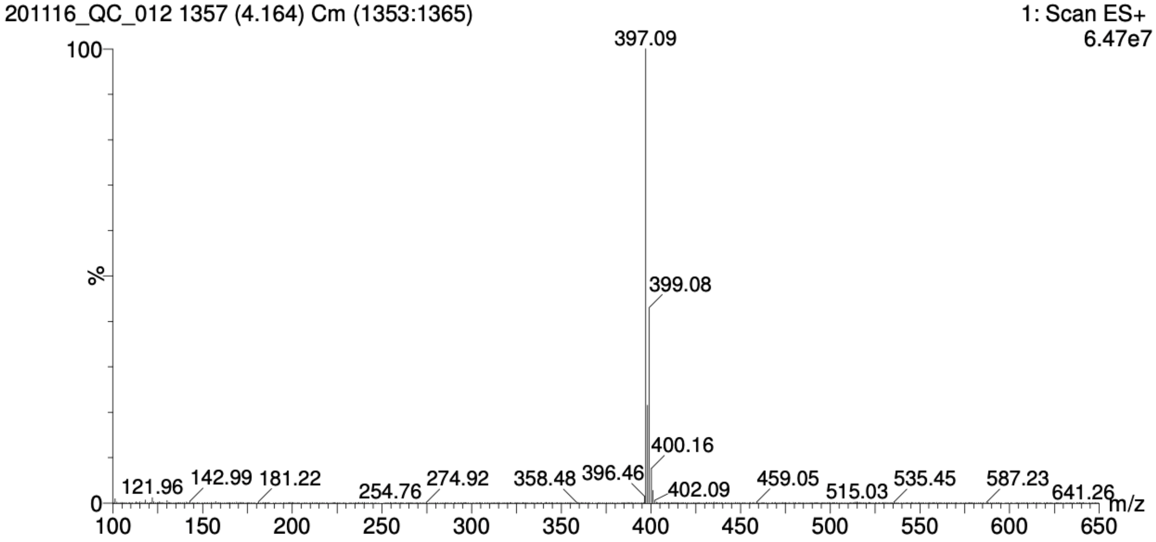

UPLC-MS purity (UV at 215 nm) of compound 10

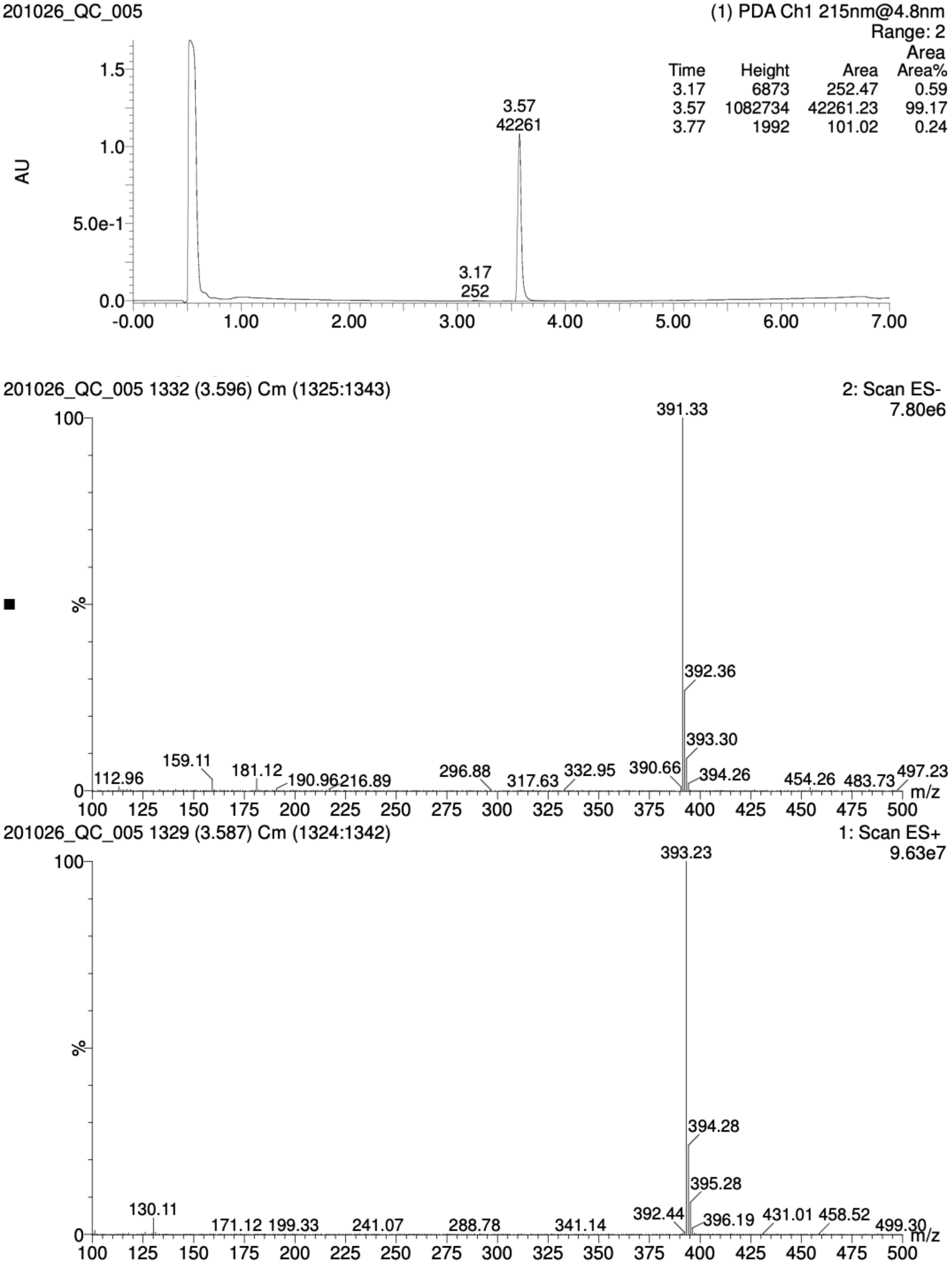

UPLC-MS purity (UV at 215 nm) of compound 11

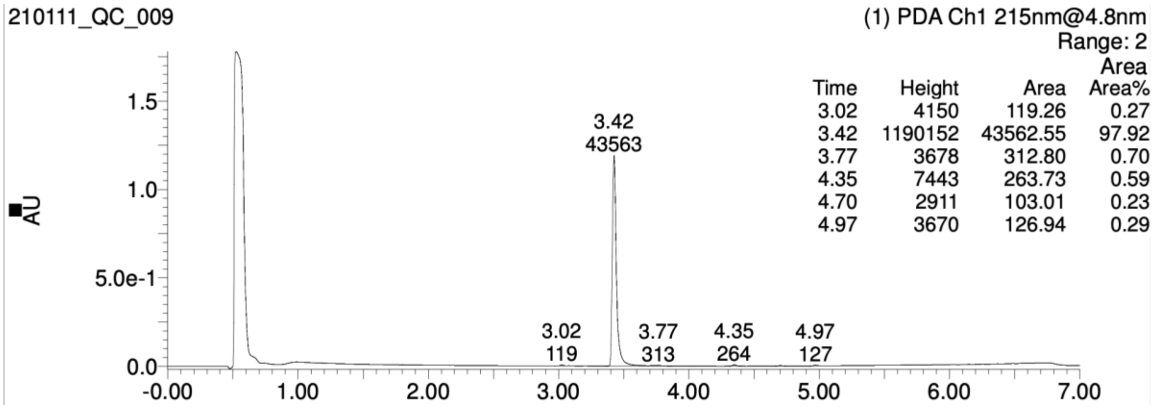

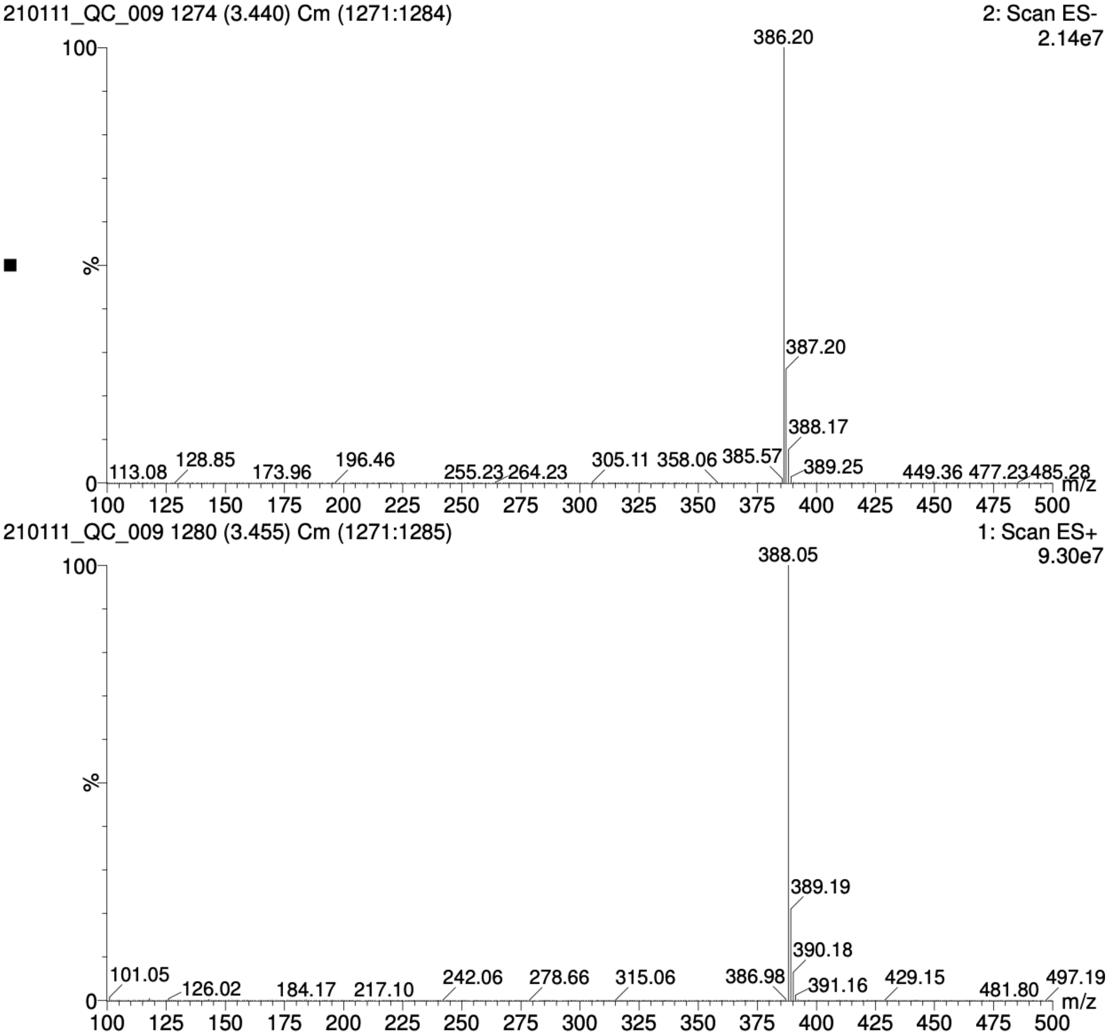

UPLC-MS purity (UV at 215 nm) of compound 12

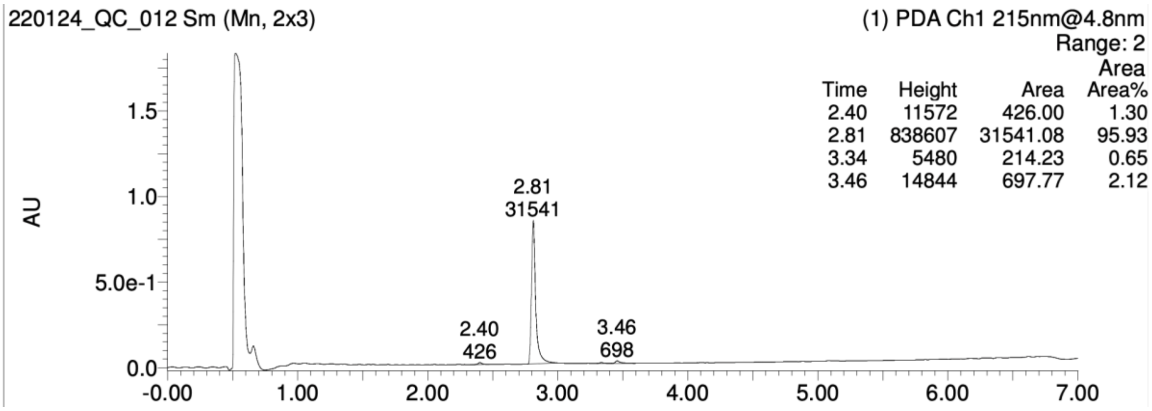

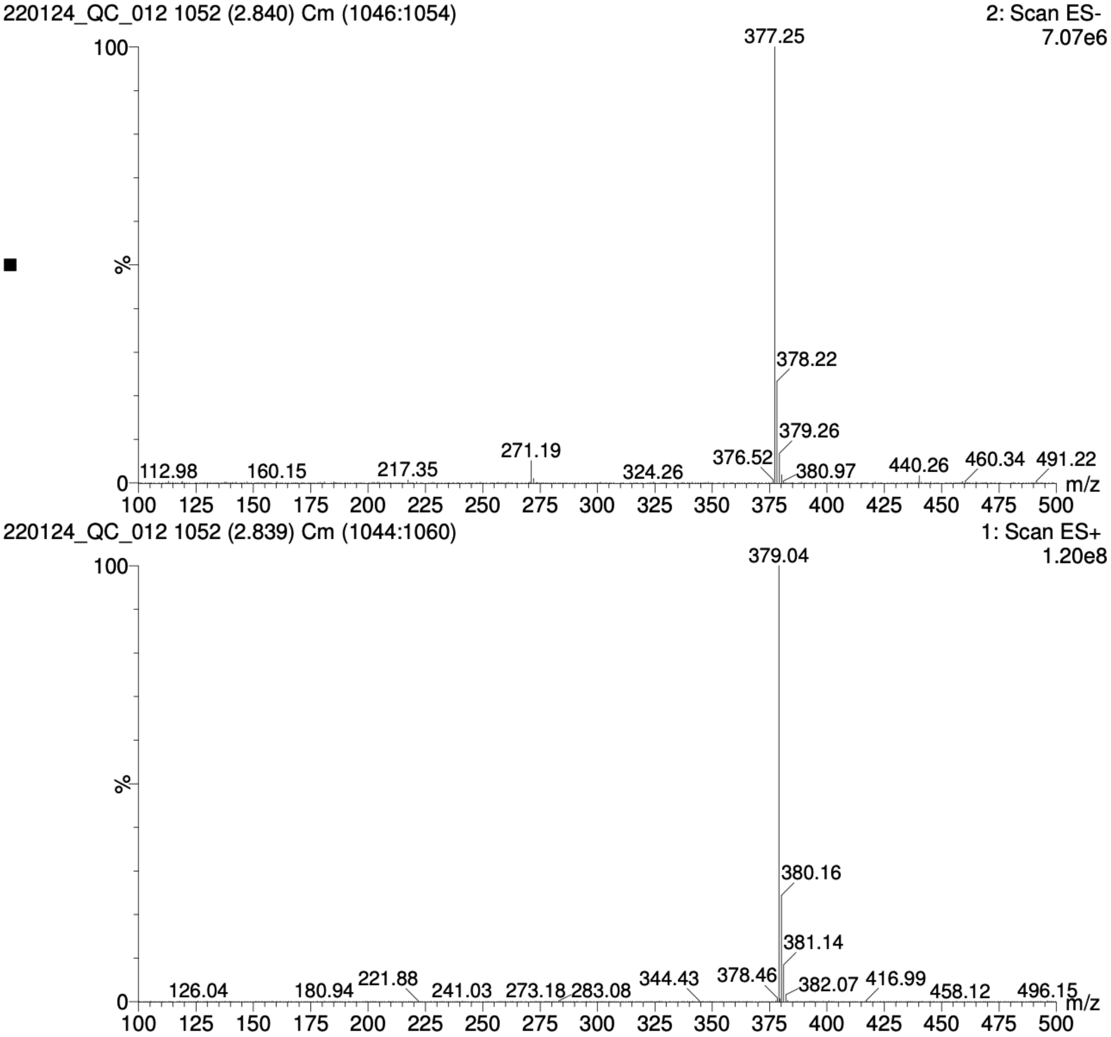

UPLC-MS purity (UV at 215 nm) of compound 13

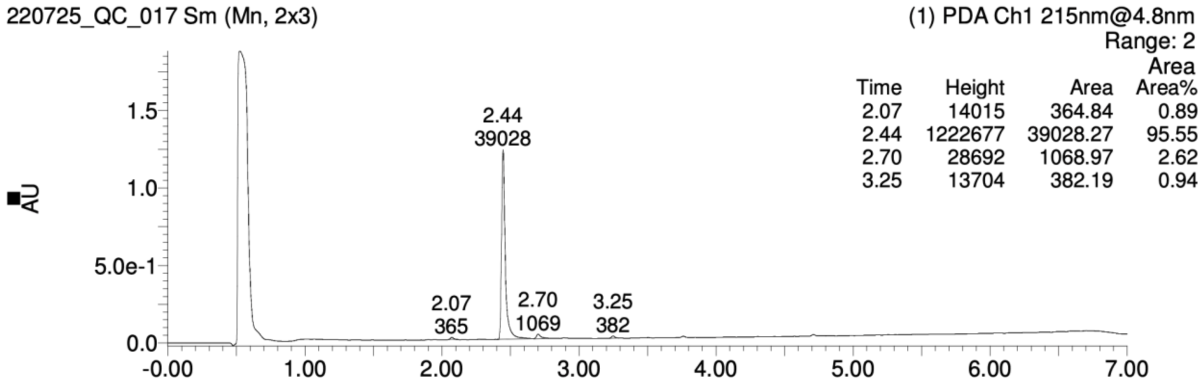

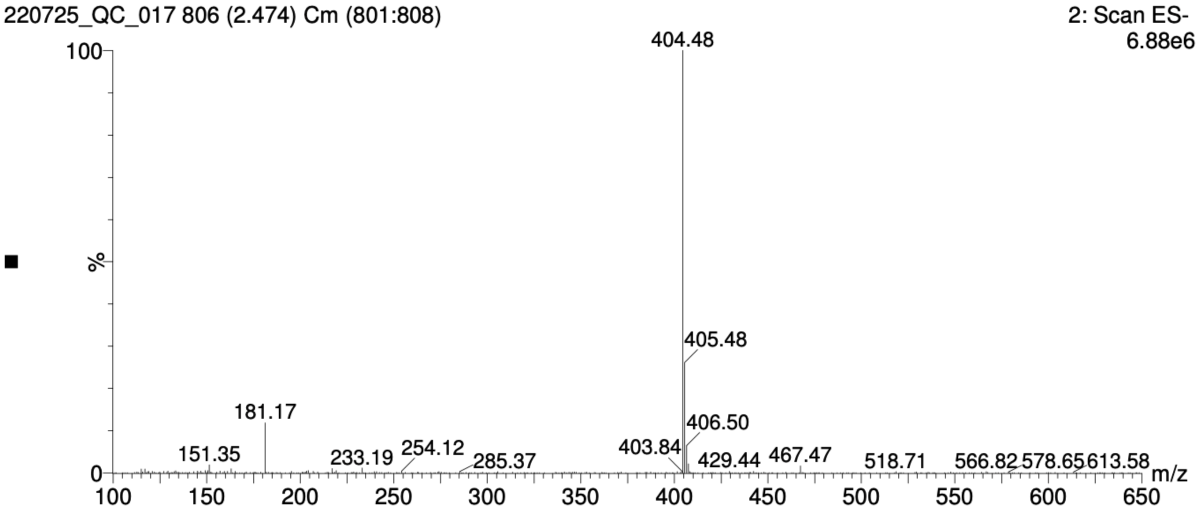

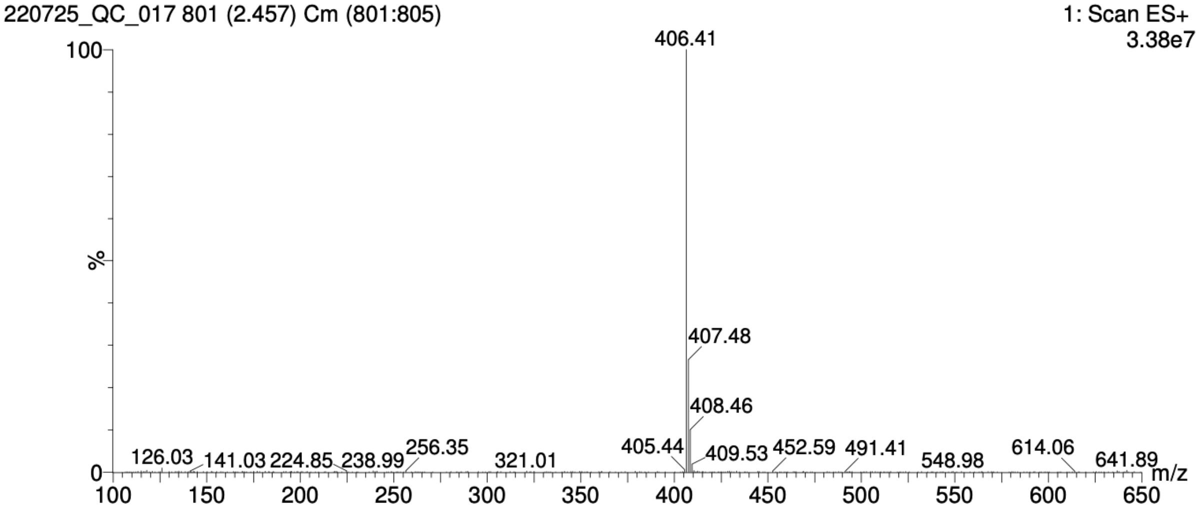

UPLC-MS purity (UV at 215 nm) of compound 14

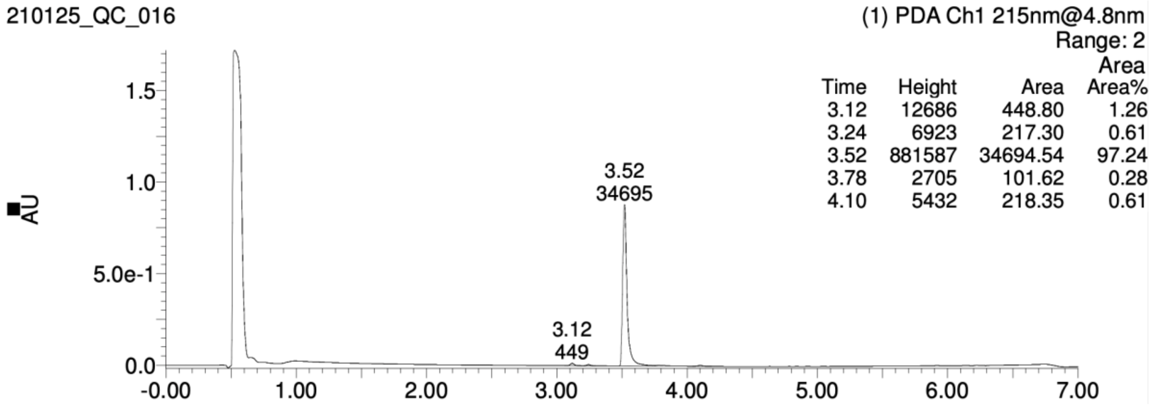

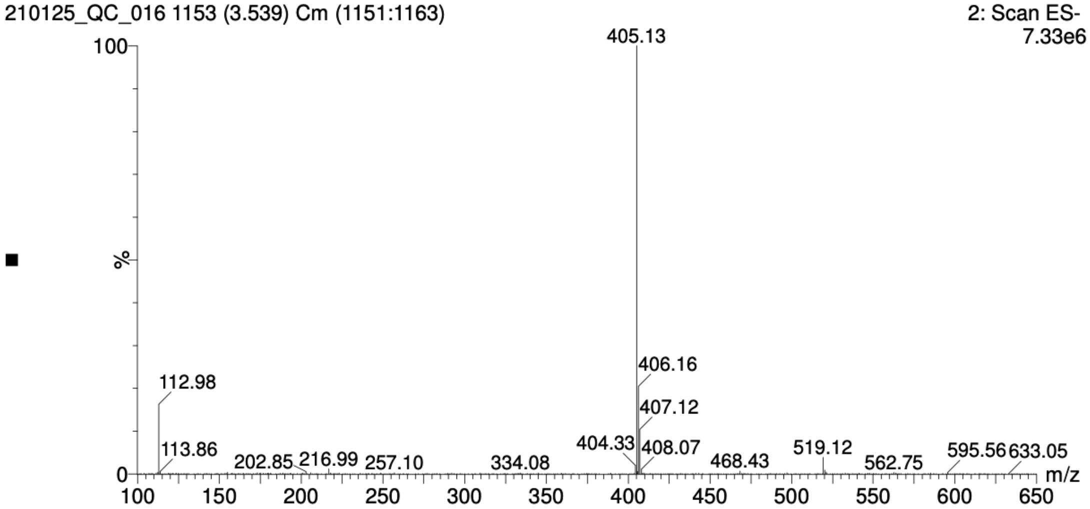

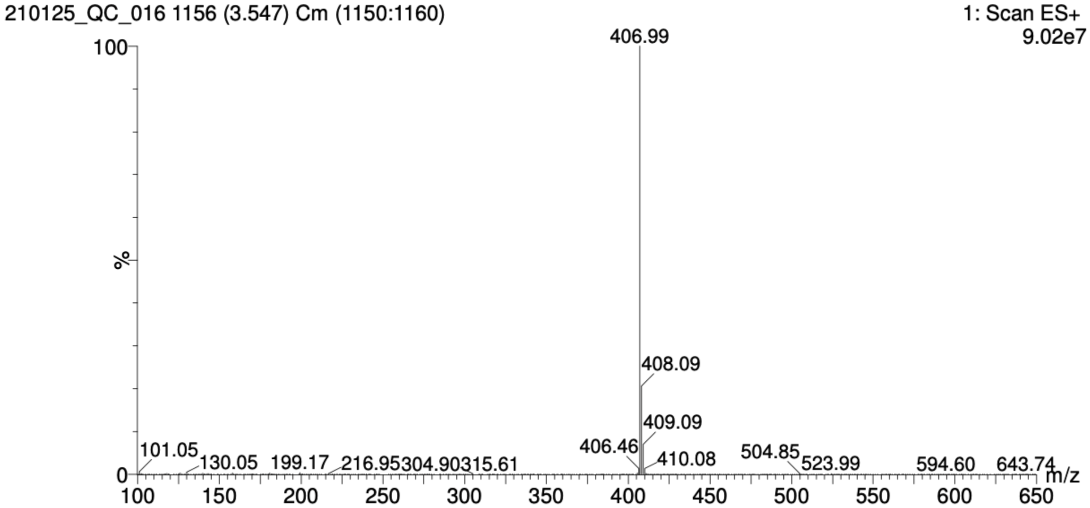

UPLC-MS purity (UV at 215 nm) of compound 15

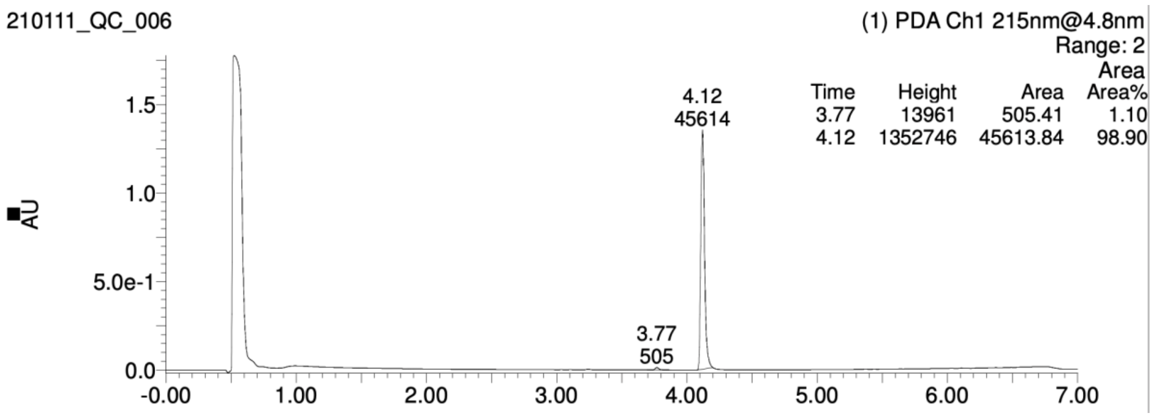

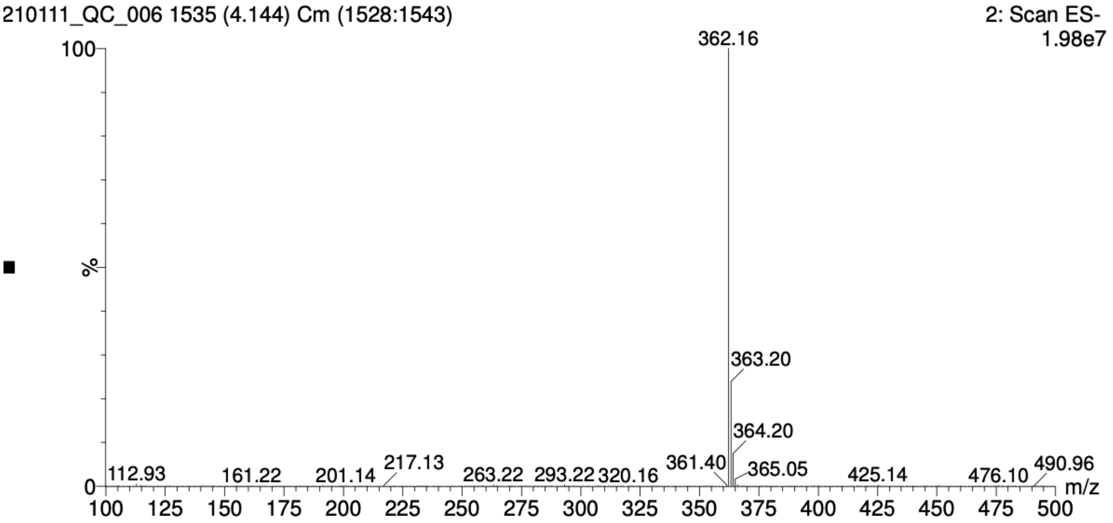

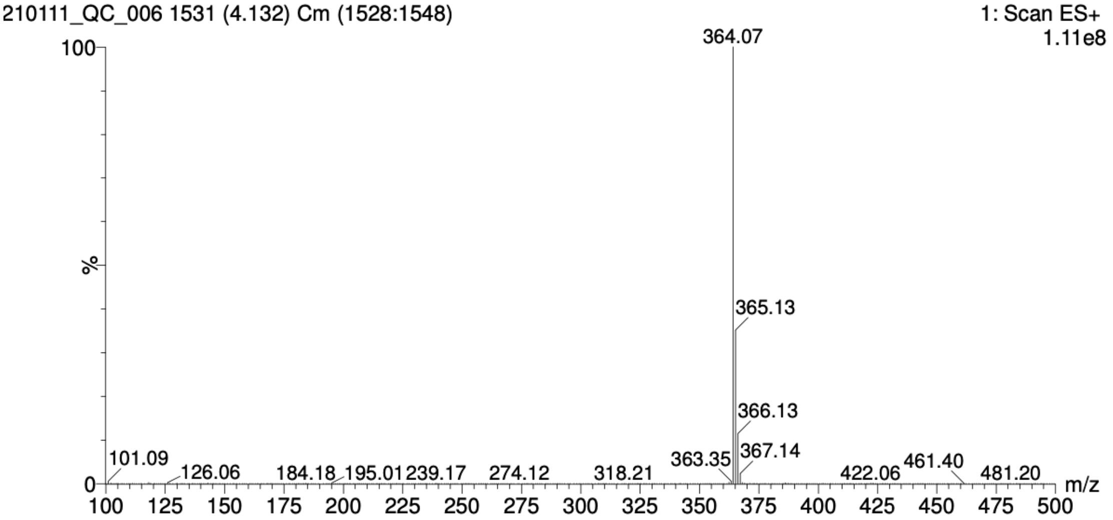

UPLC-MS purity (UV at 215 nm) of compound 16

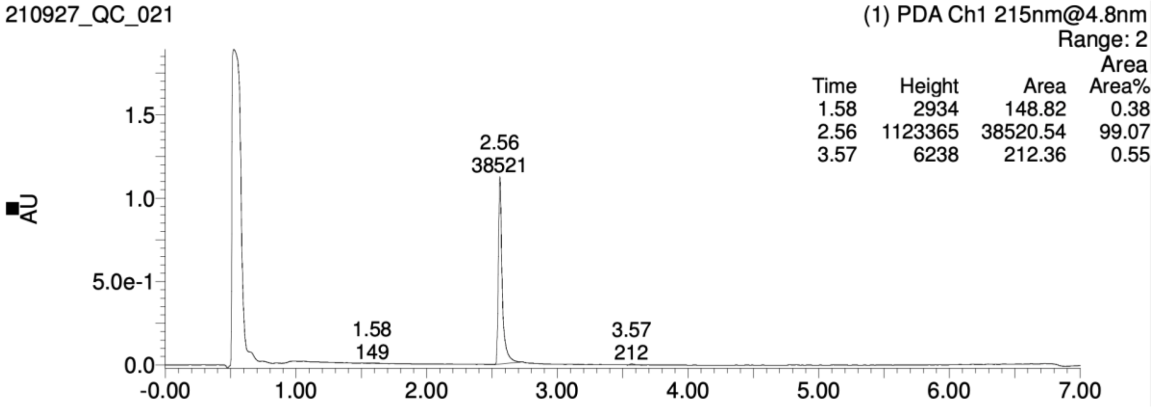

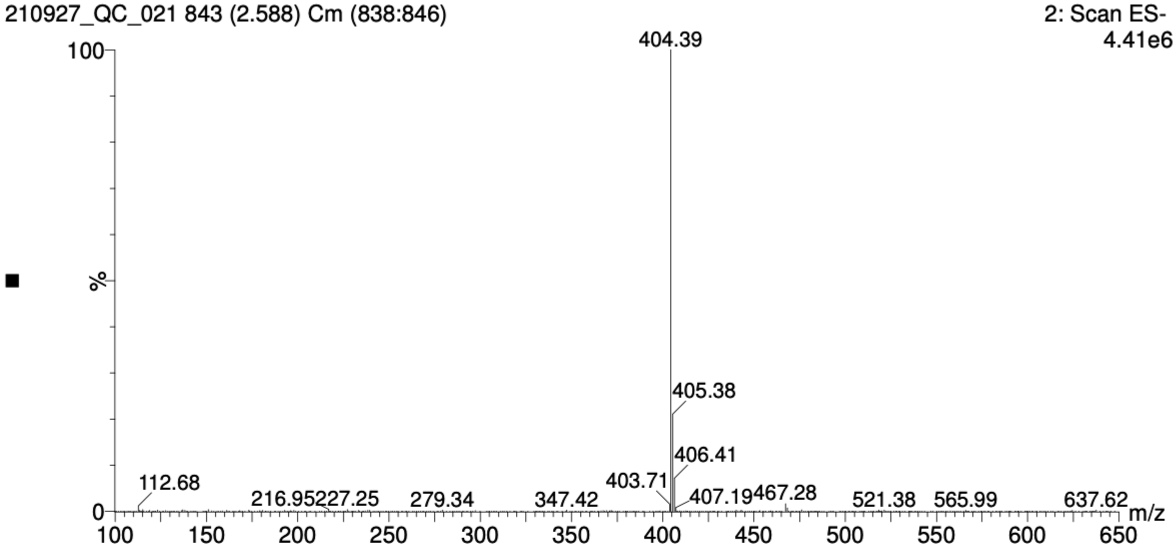

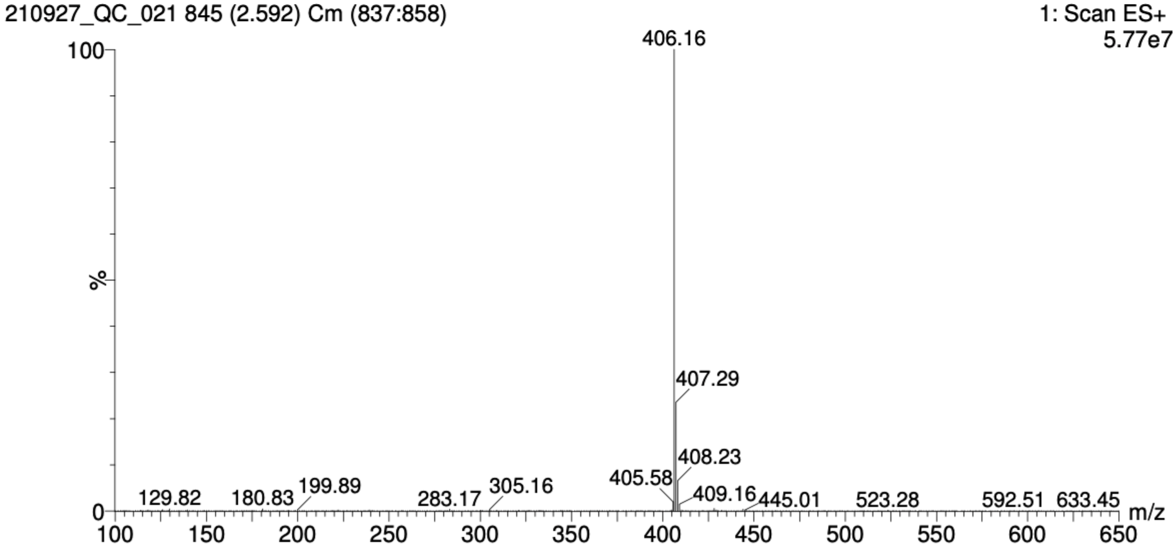

UPLC-MS purity (UV at 215 nm) of compound 17

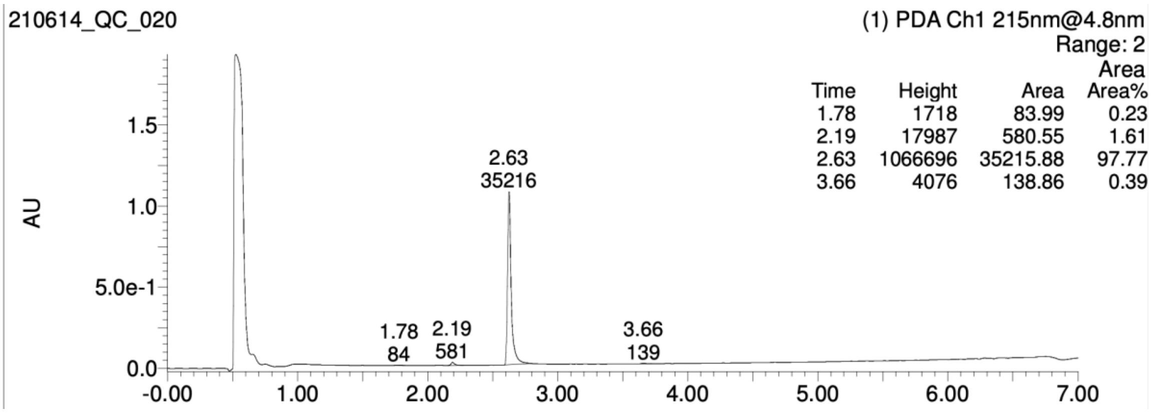

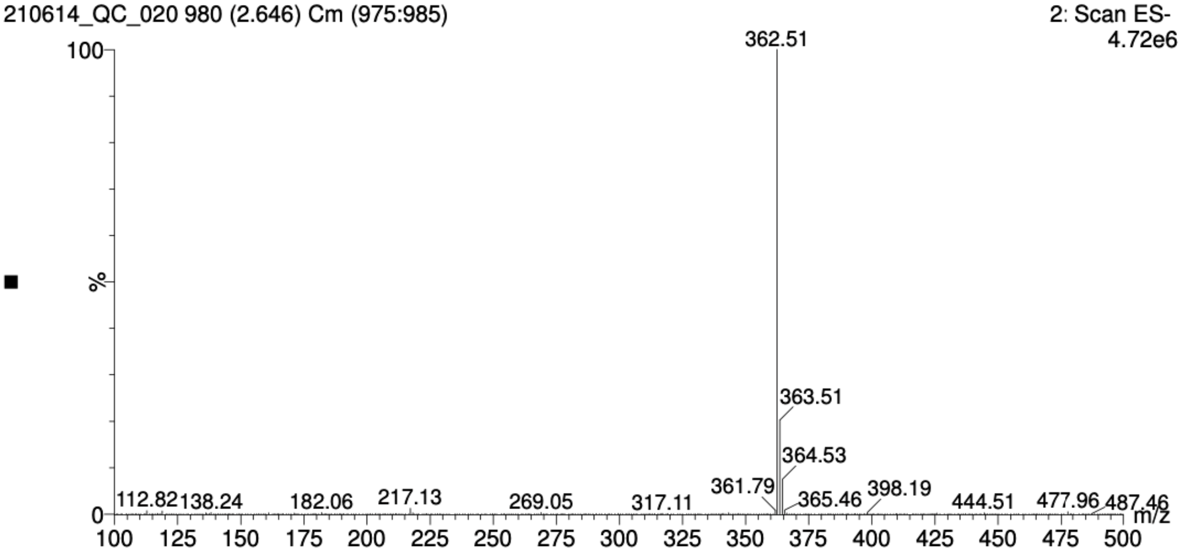

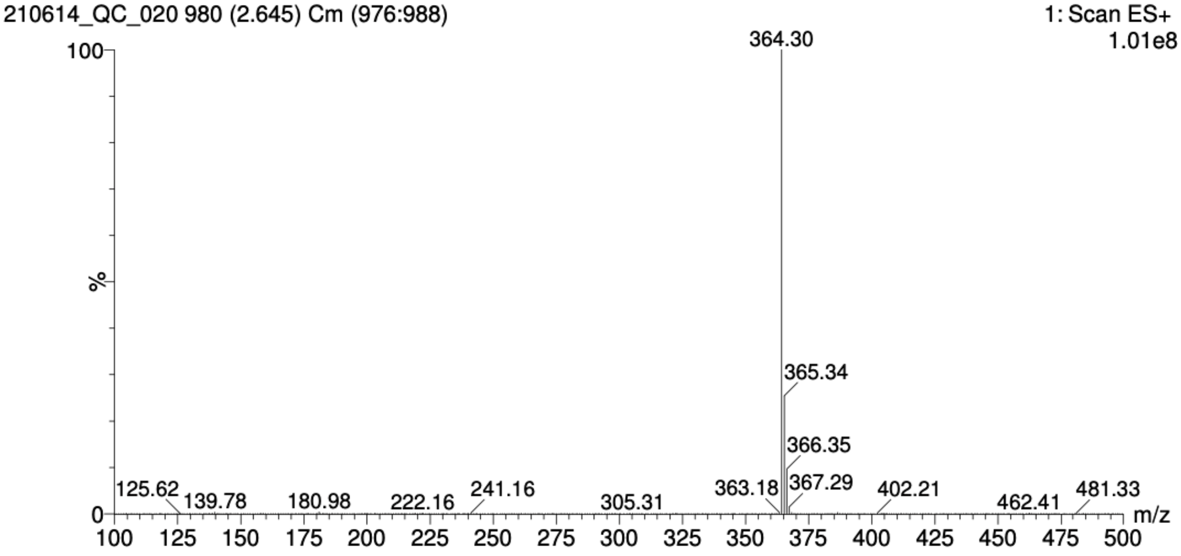

UPLC-MS purity (UV at 215 nm) of compound 18

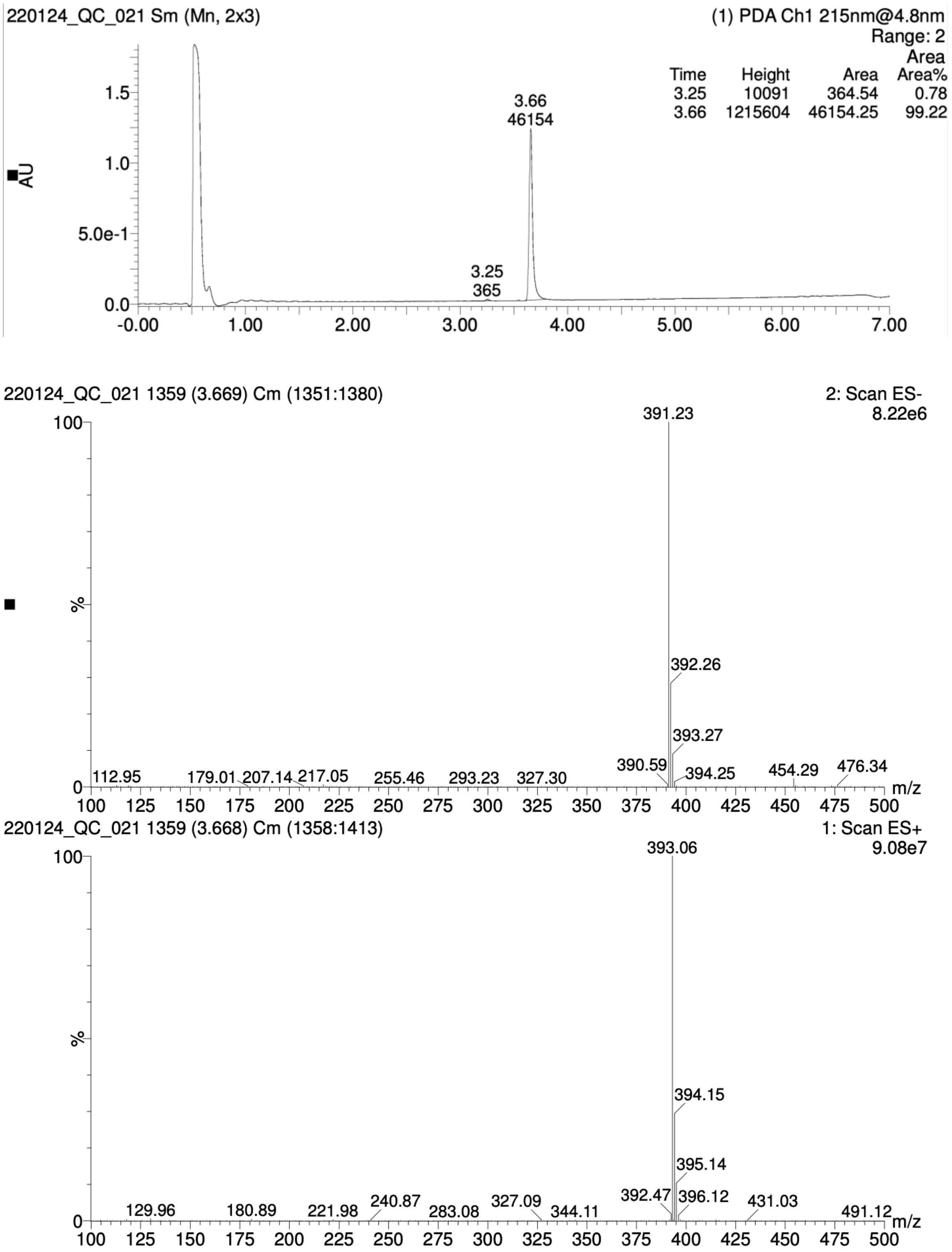

UPLC-MS purity (UV at 215 nm) of compound 19

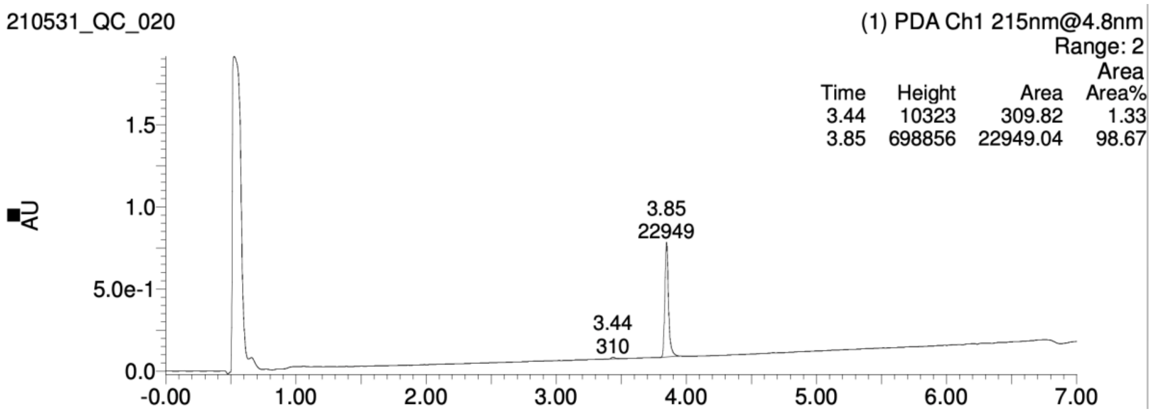

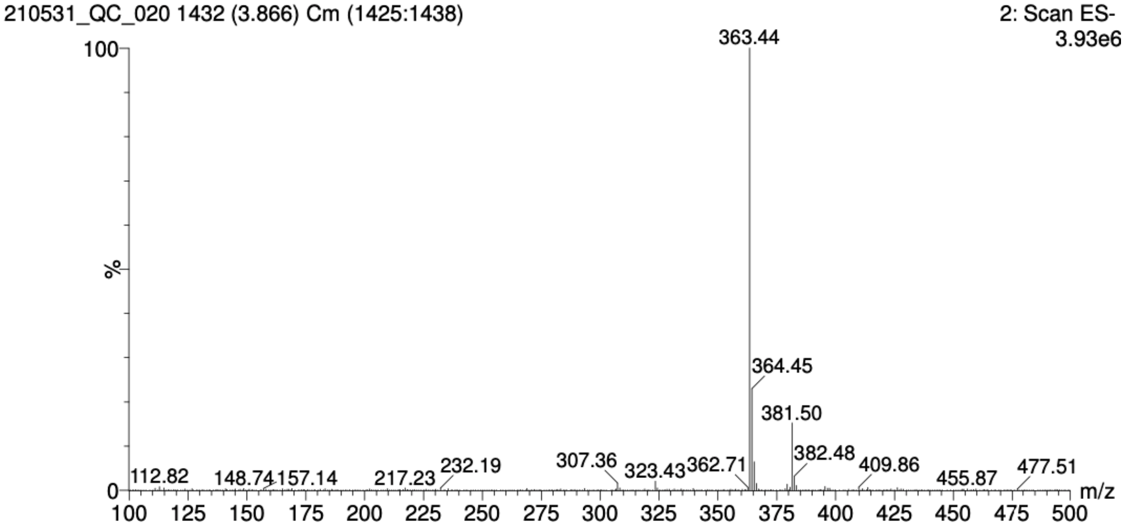

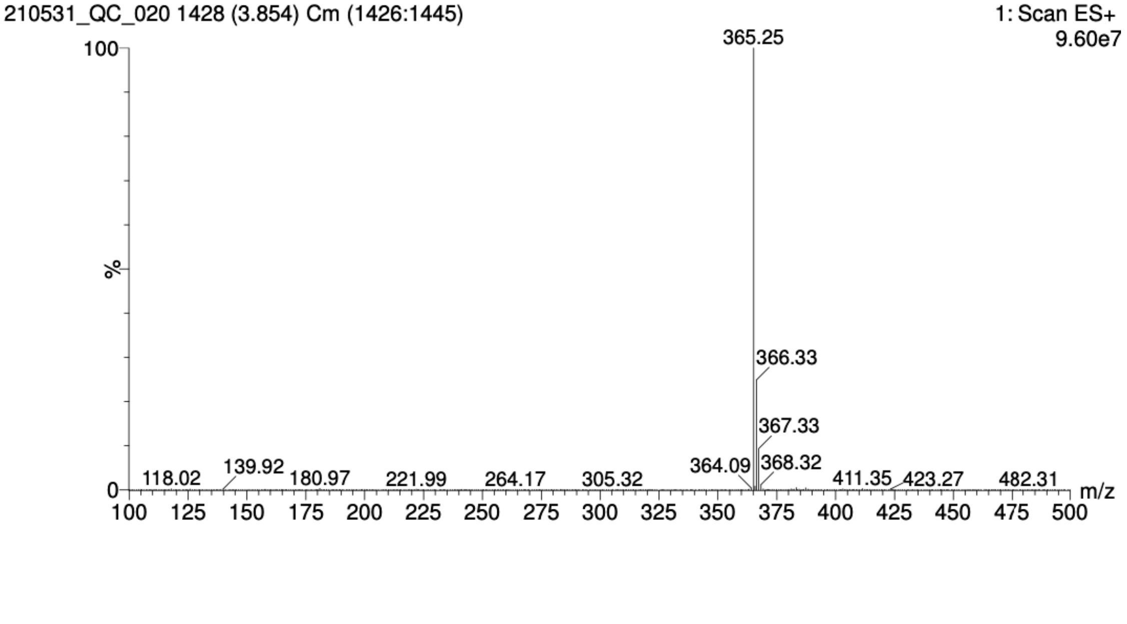

UPLC-MS purity (UV at 215 nm) of compound 20

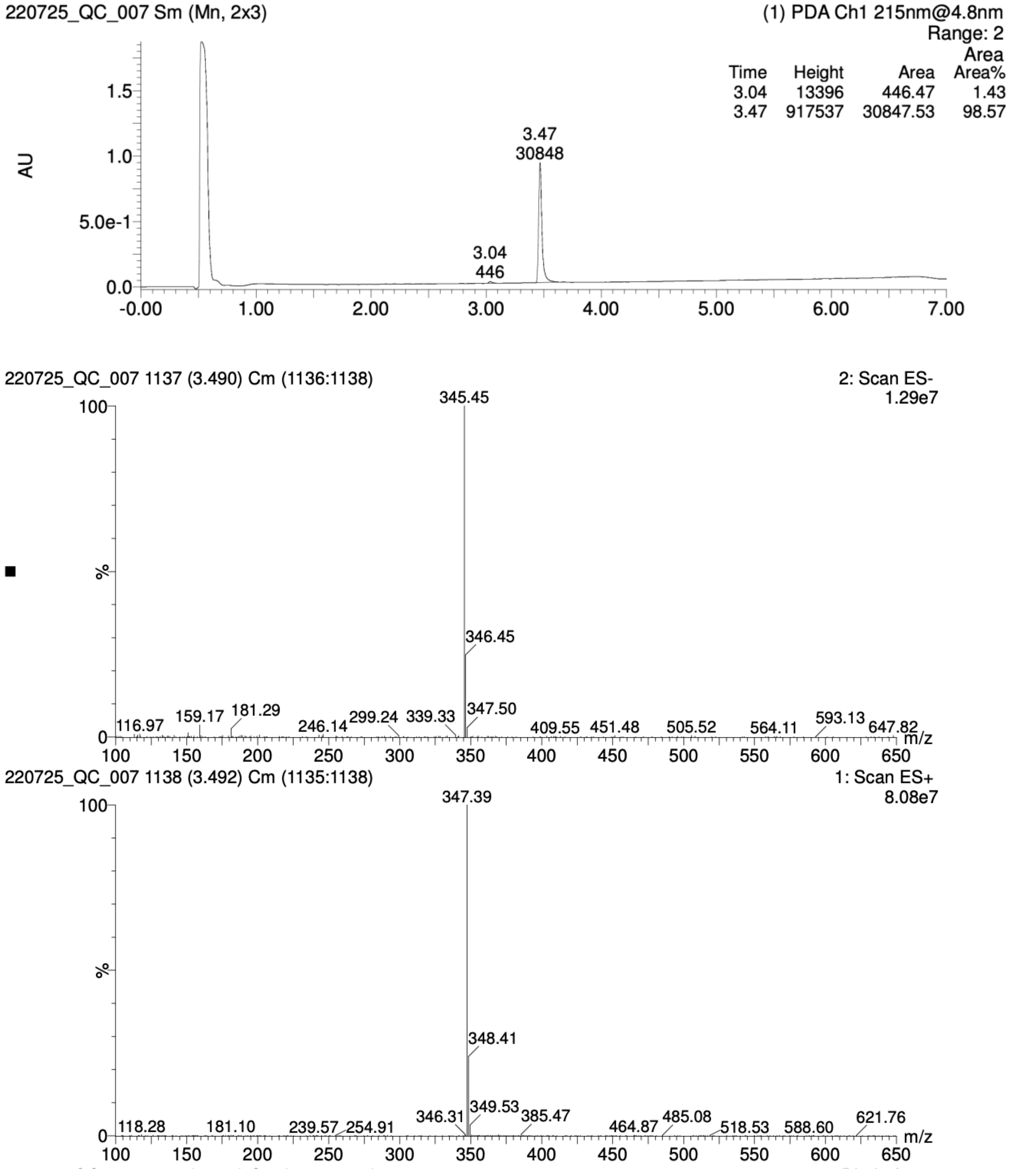

UPLC-MS purity (UV at 215 nm) of compound 21

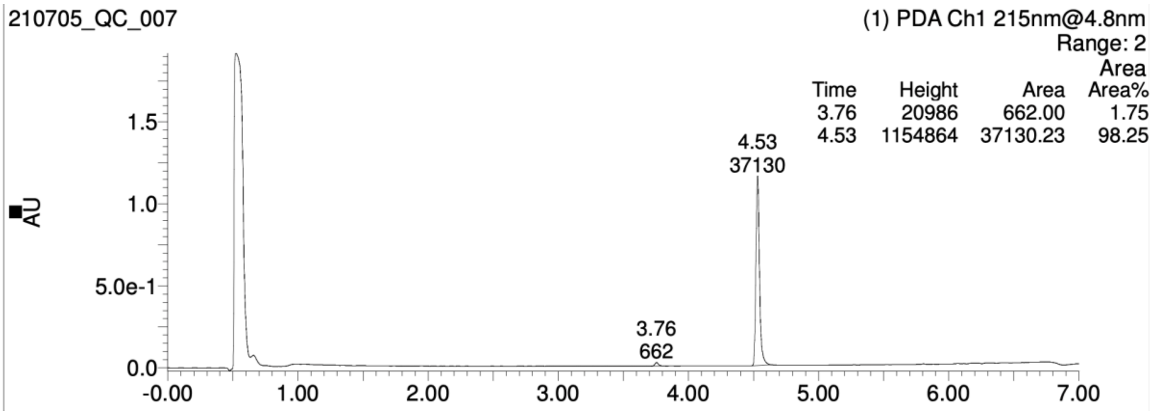

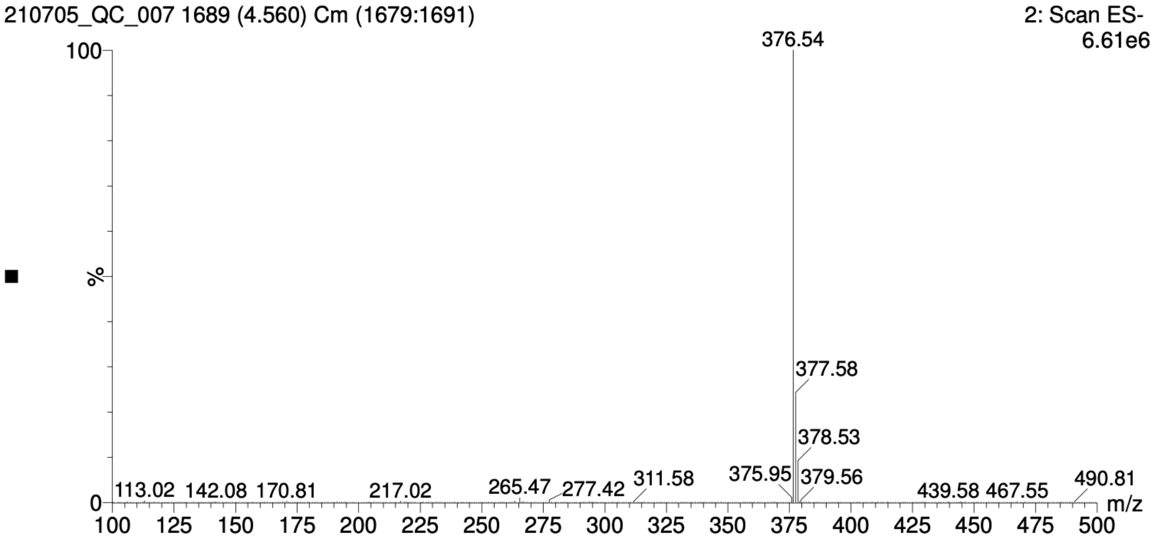

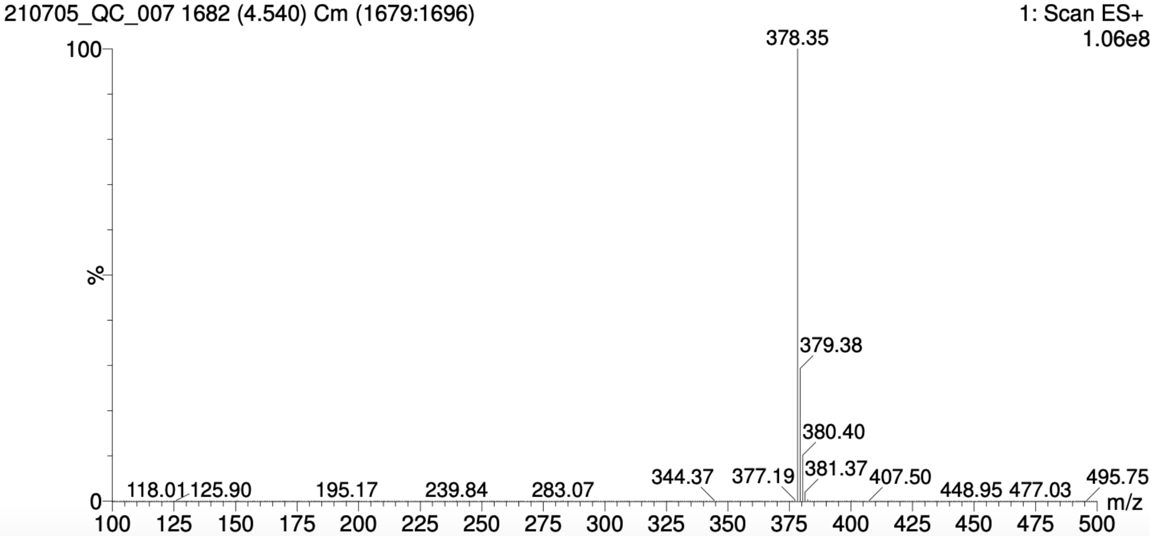

UPLC-MS purity (UV at 215 nm) of compound 22

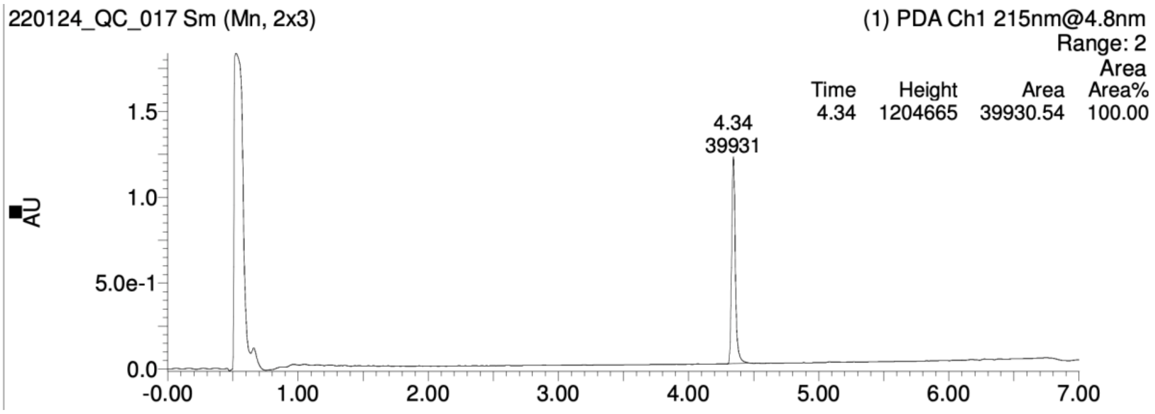

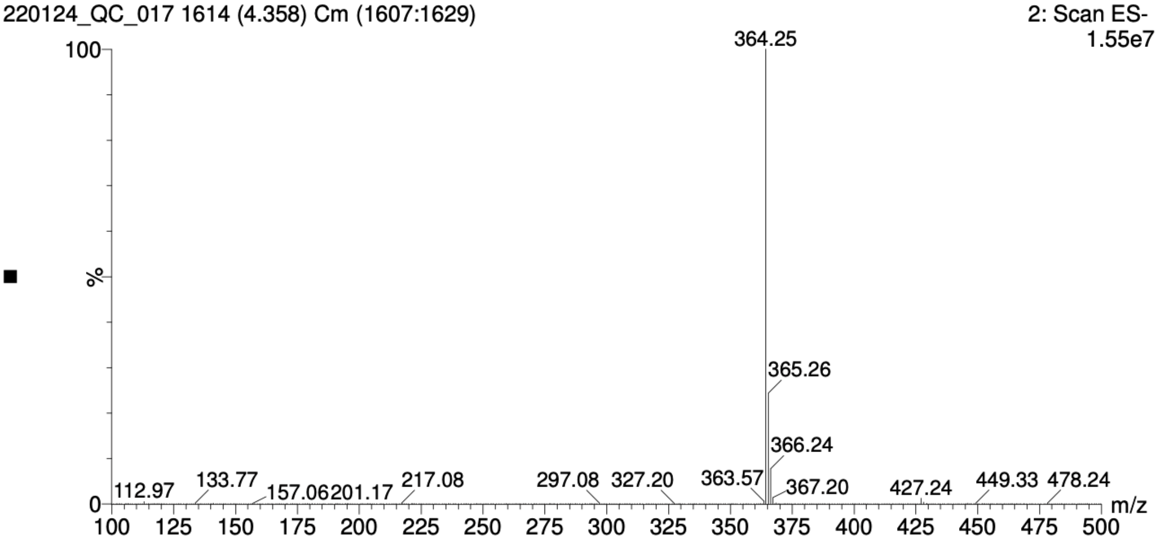

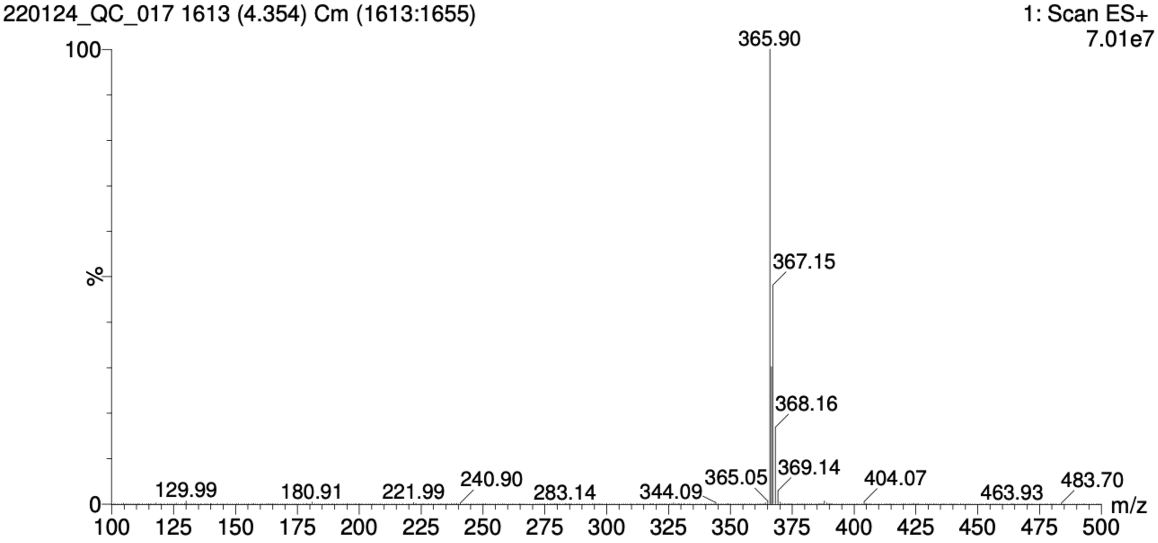

UPLC-MS purity (UV at 215 nm) of compound 23

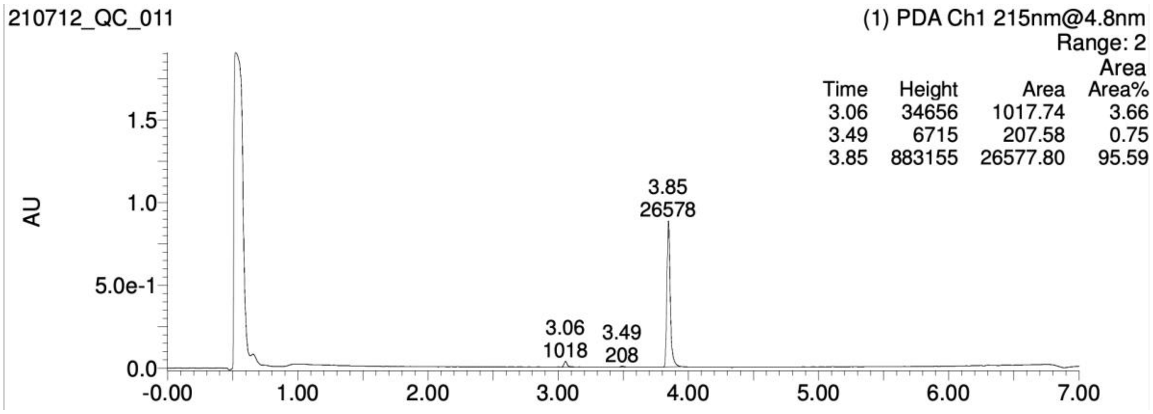

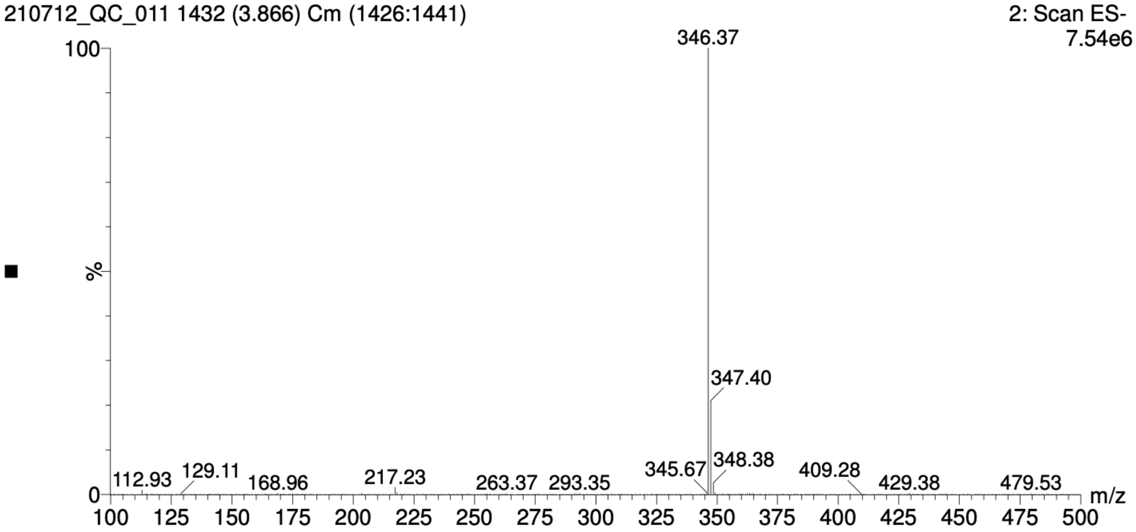

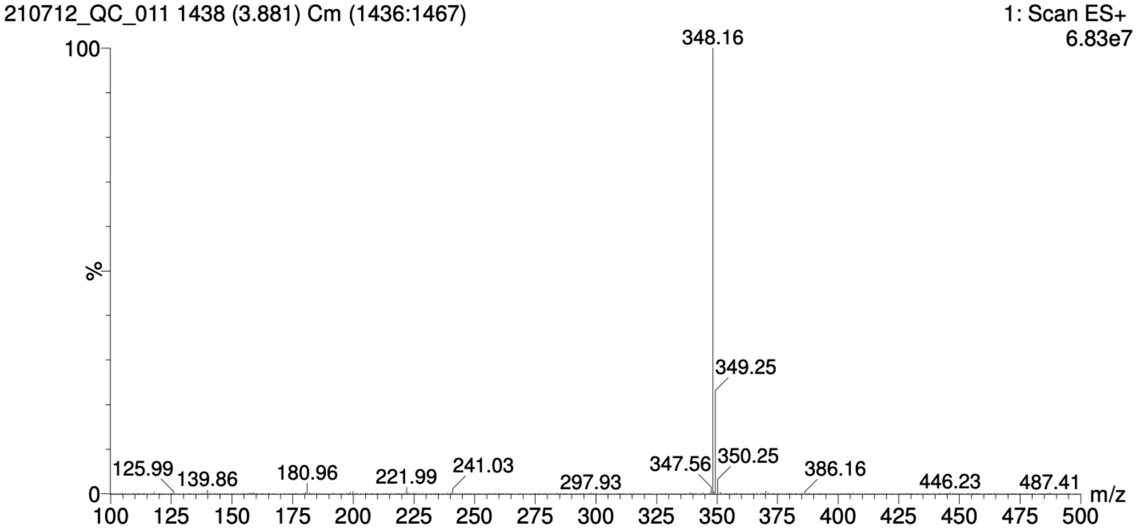

UPLC-MS purity (UV at 215 nm) of compound 24

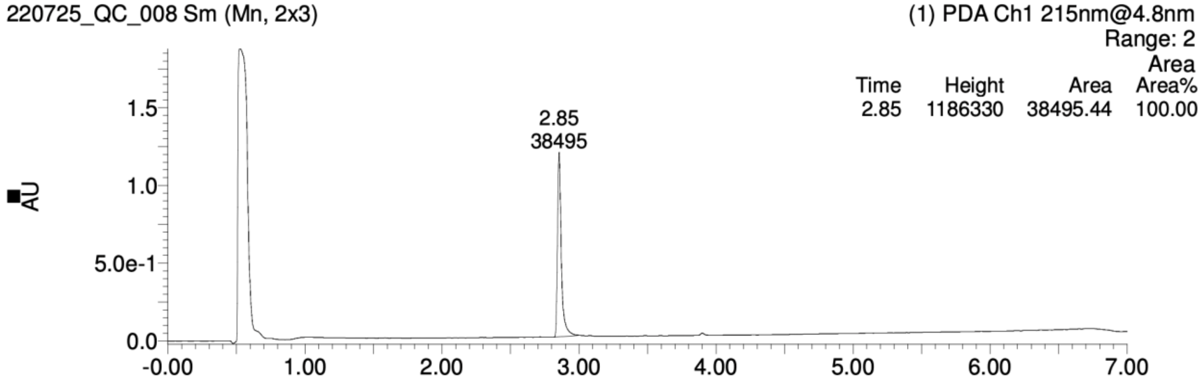

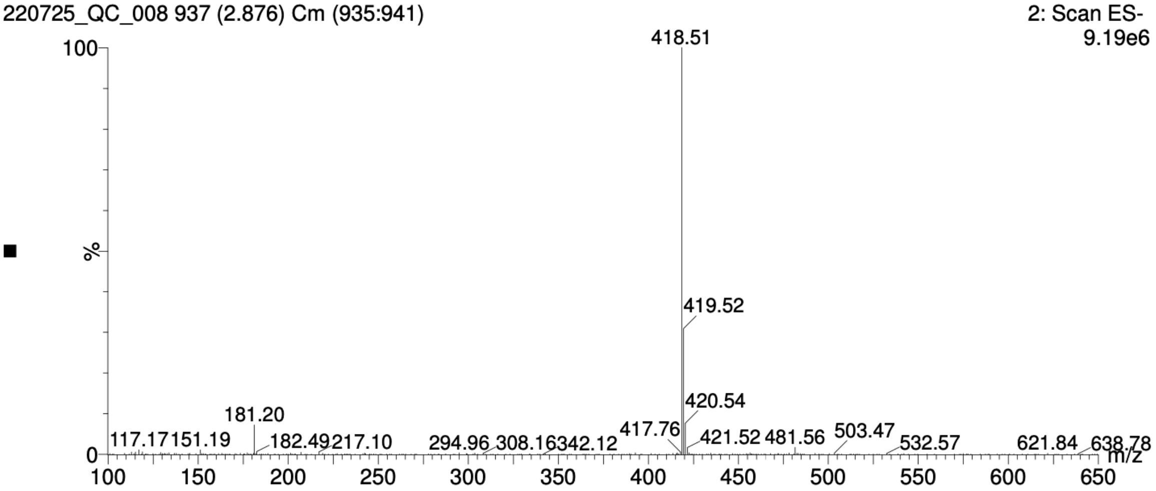

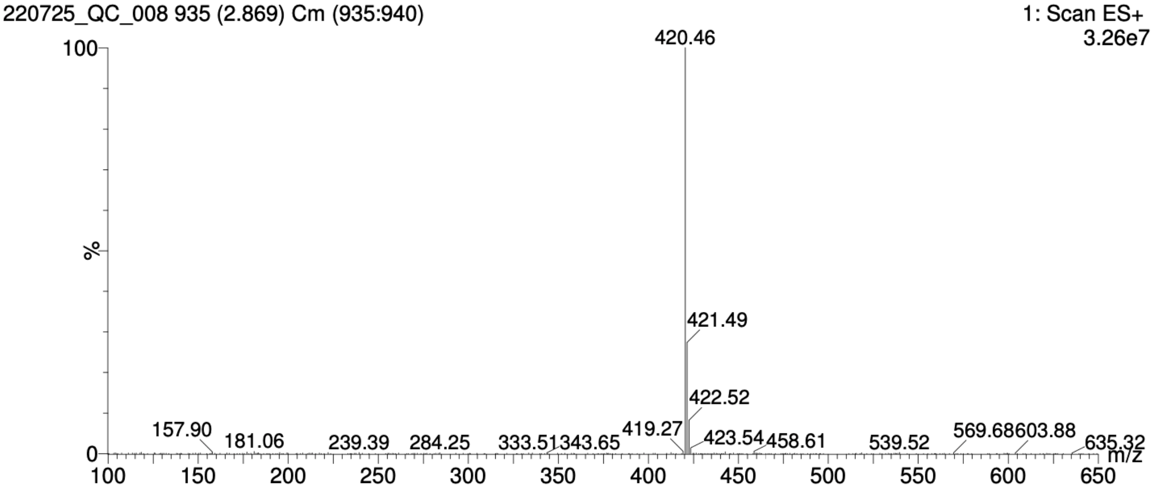

UPLC-MS purity (UV at 215 nm) of compound 25

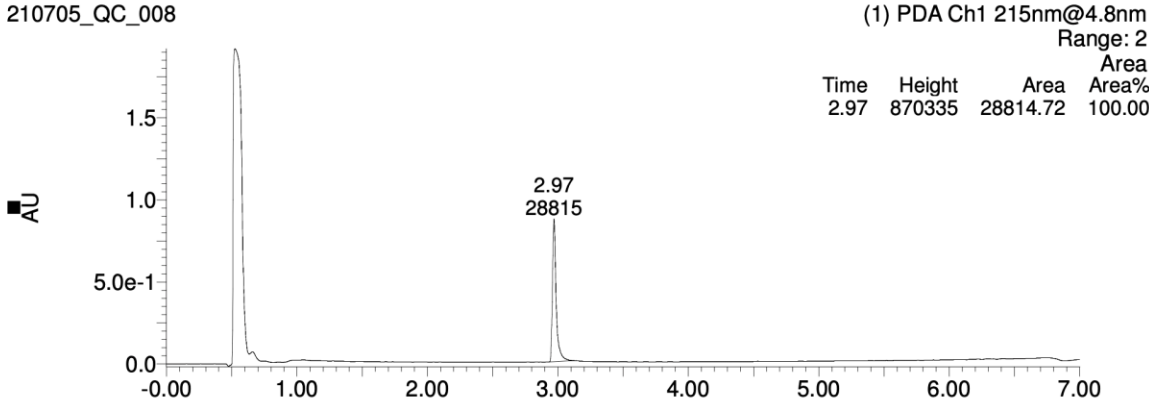

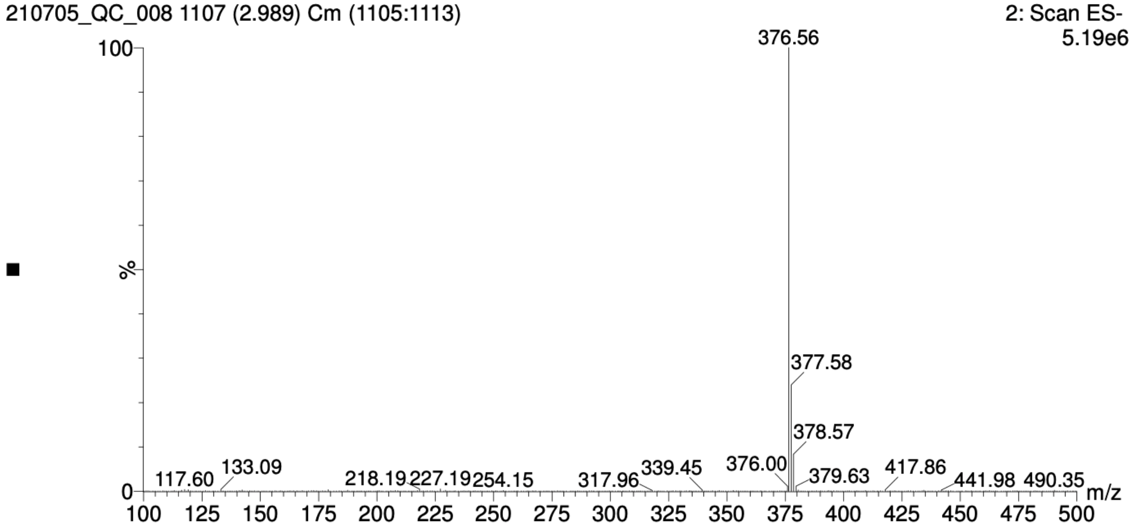

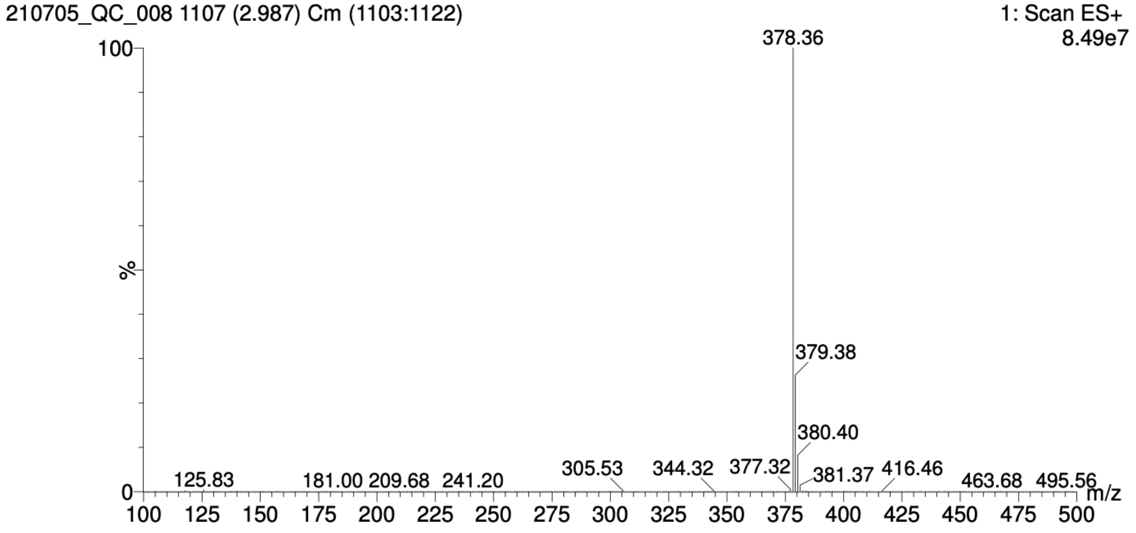

UPLC-MS purity (UV at 215 nm) of compound 26

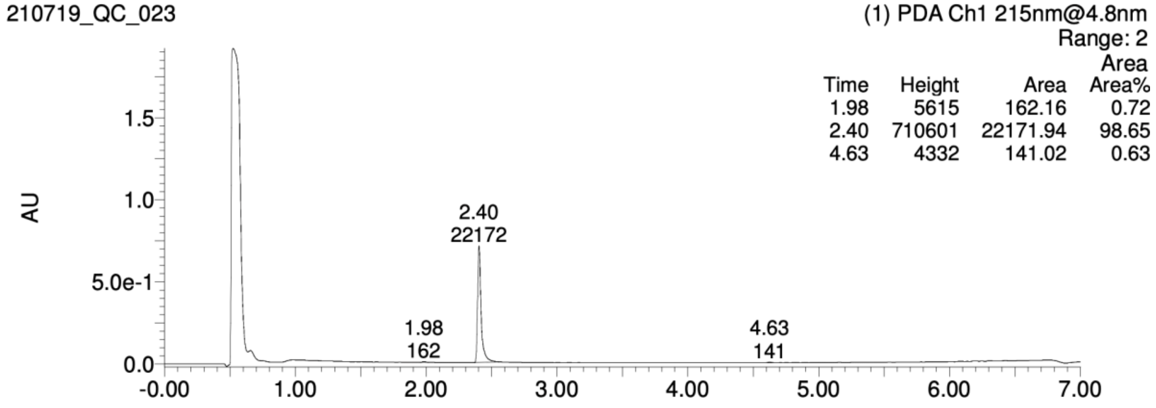

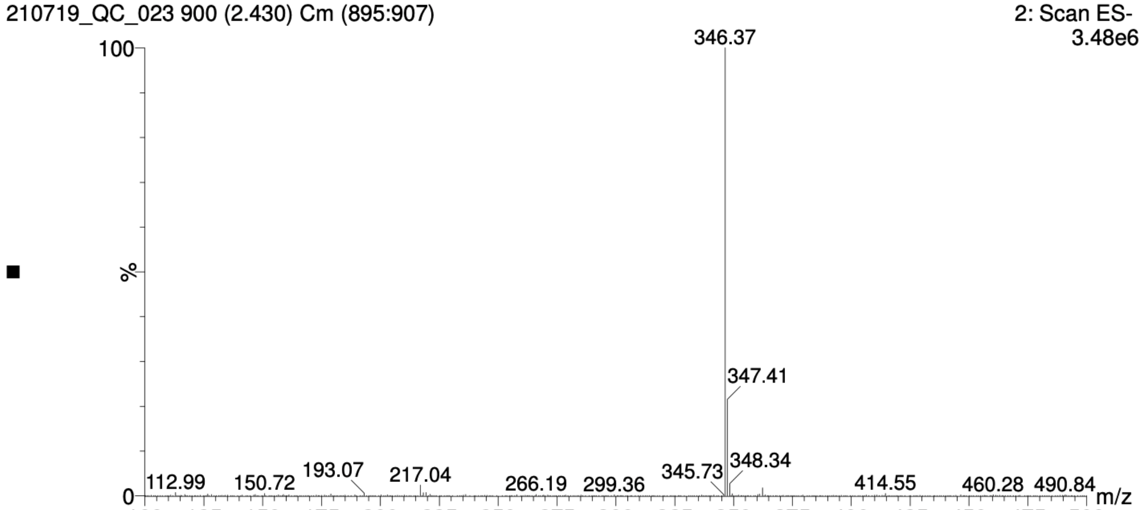

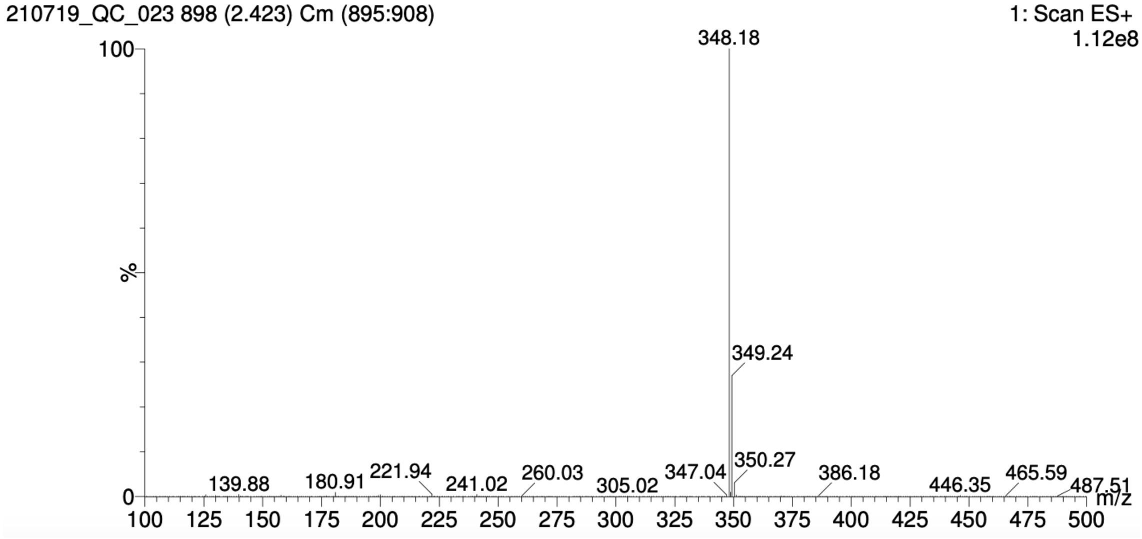

UPLC-MS purity (UV at 215 nm) of compound 27

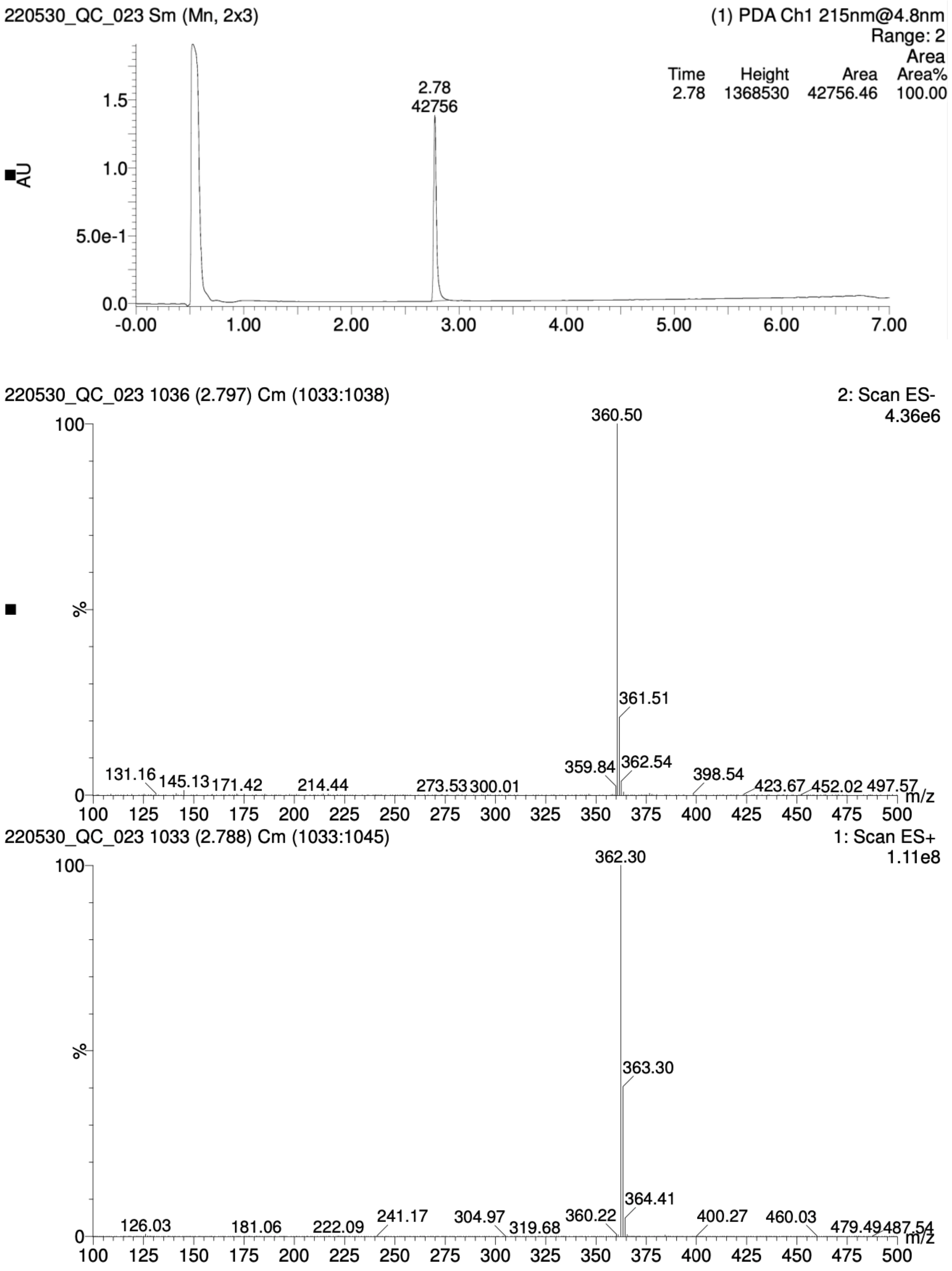

UPLC-MS purity (UV at 215 nm) of compound 28

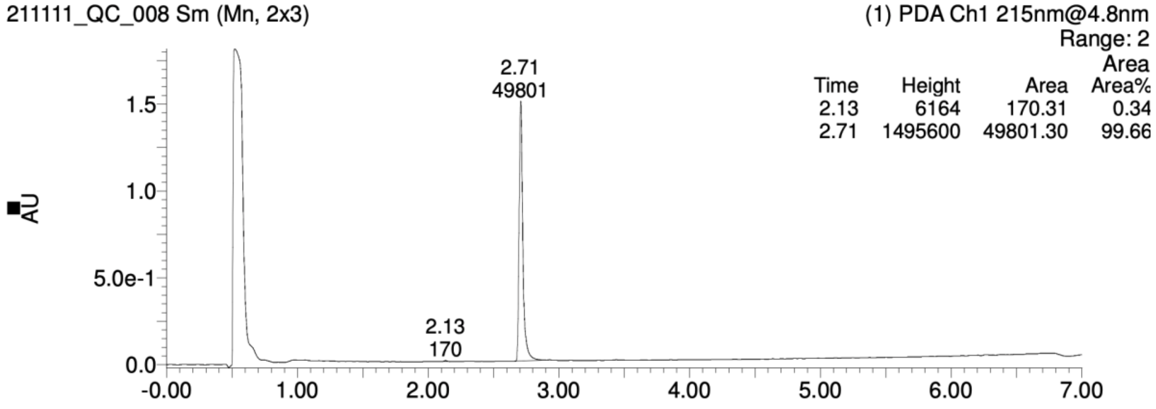

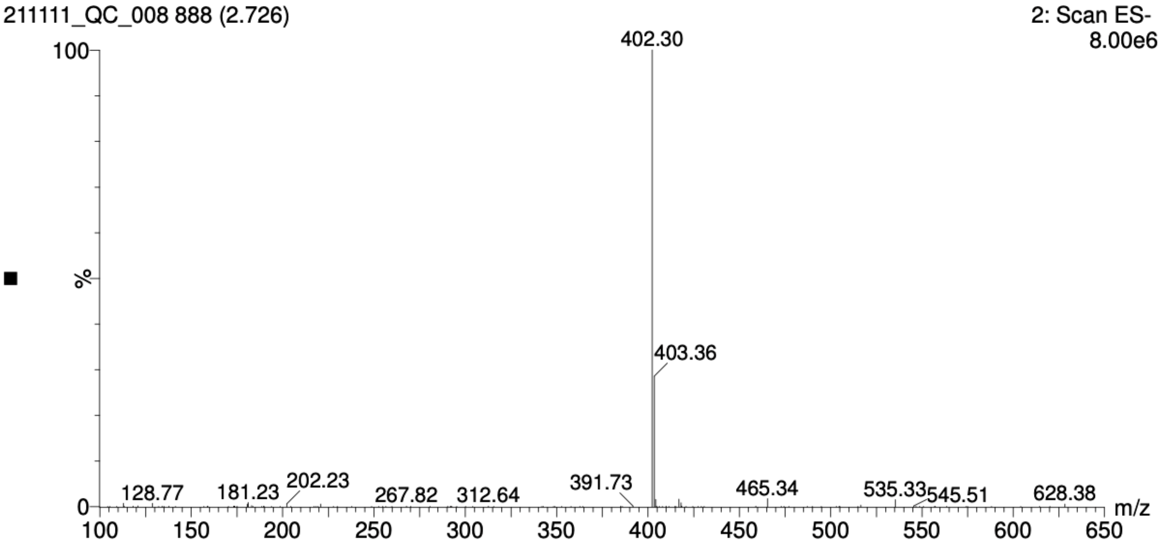

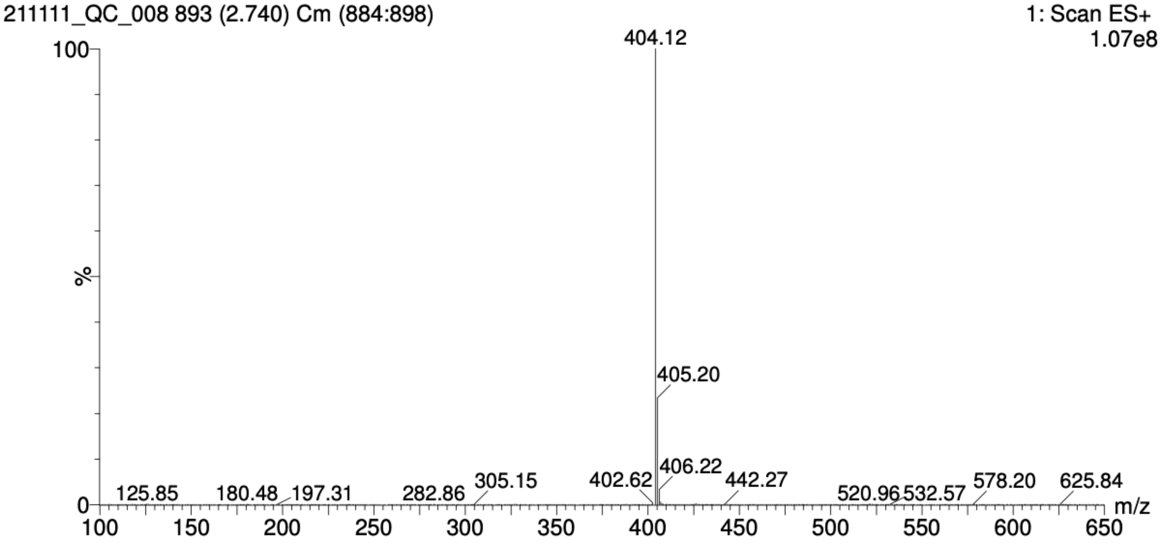

UPLC-MS purity (UV at 215 nm) of compound 31

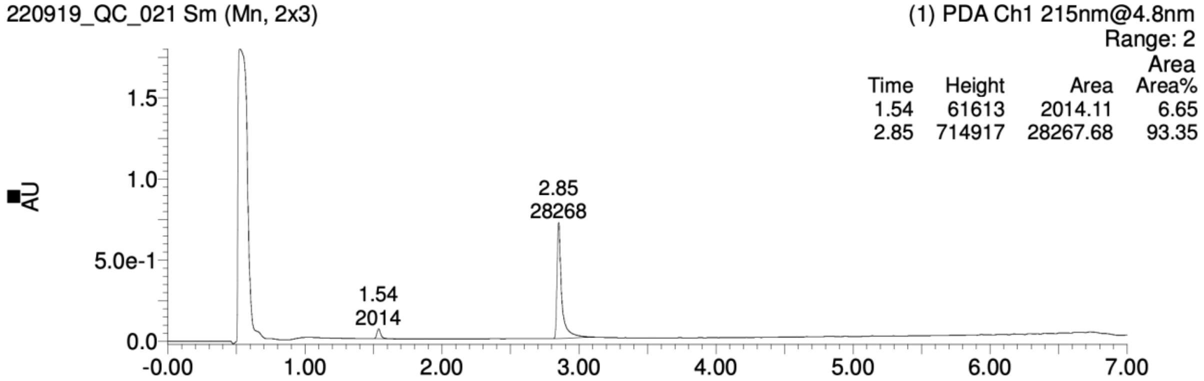

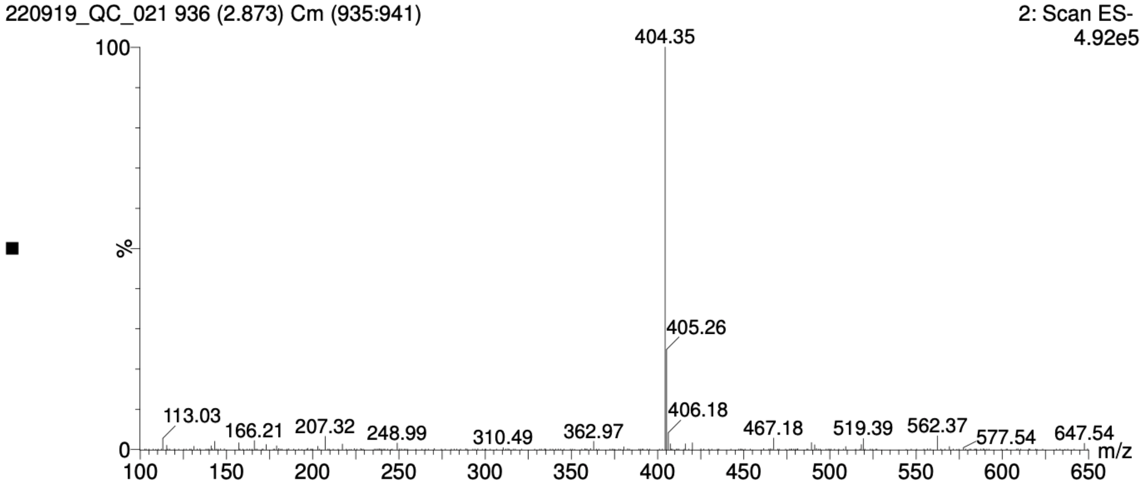

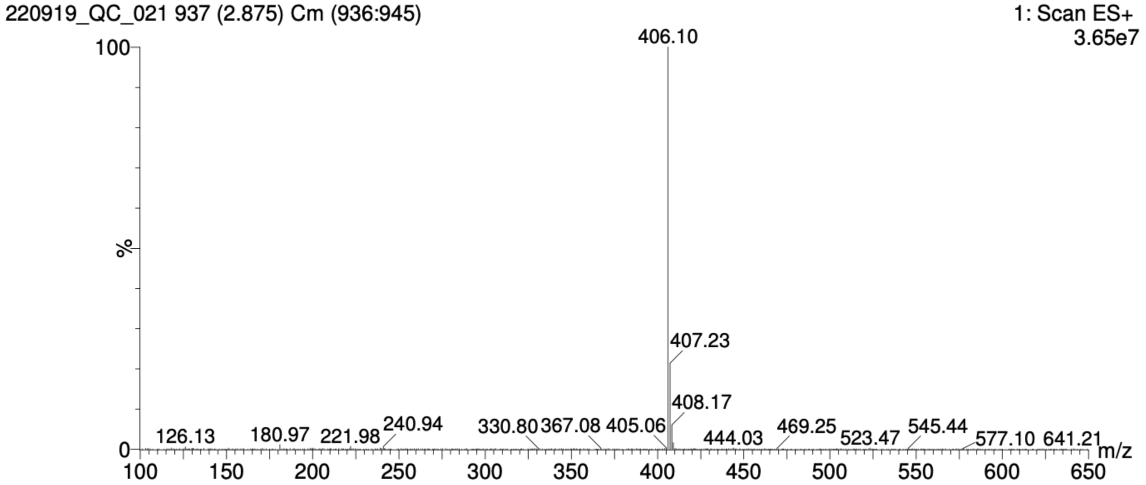

Supplement: Supplementary file 1 [file jm5c01810_si_001.pdf]
